# Supplementary material for: Optimized design parameters for CRISPR Cas9 and Cas12a homology-directed repair
Source: Sci Rep. 2021 Sep 30;11:19482. doi: 10.1038/s41598-021-98965-y (PMC8484621; doi:10.1038/s41598-021-98965-y)
Supplement: Supplementary file 1 — Supplementary Figures. [file 41598_2021_98965_MOESM1_ESM.pdf]

Supplemental Data Set

Optimized design parameters for CRISPR Cas9 and Cas12a homology-directed repair

Mollie S. Schubert[1], Bernice Thommandru[1], Jessica Woodley[1], Rolf Turk[1], Shuqi Yan[1,2], Gavin Kurgan[1], Matthew S. McNeill[1] and Garrett R. Rettig[1,*]

[1] Integrated DNA Technologies, Inc., Coralville, IA, 52241, USA

[2] Present address: BGI Genomics, BGI-Shenzhen, Shenzhen 518083, China.

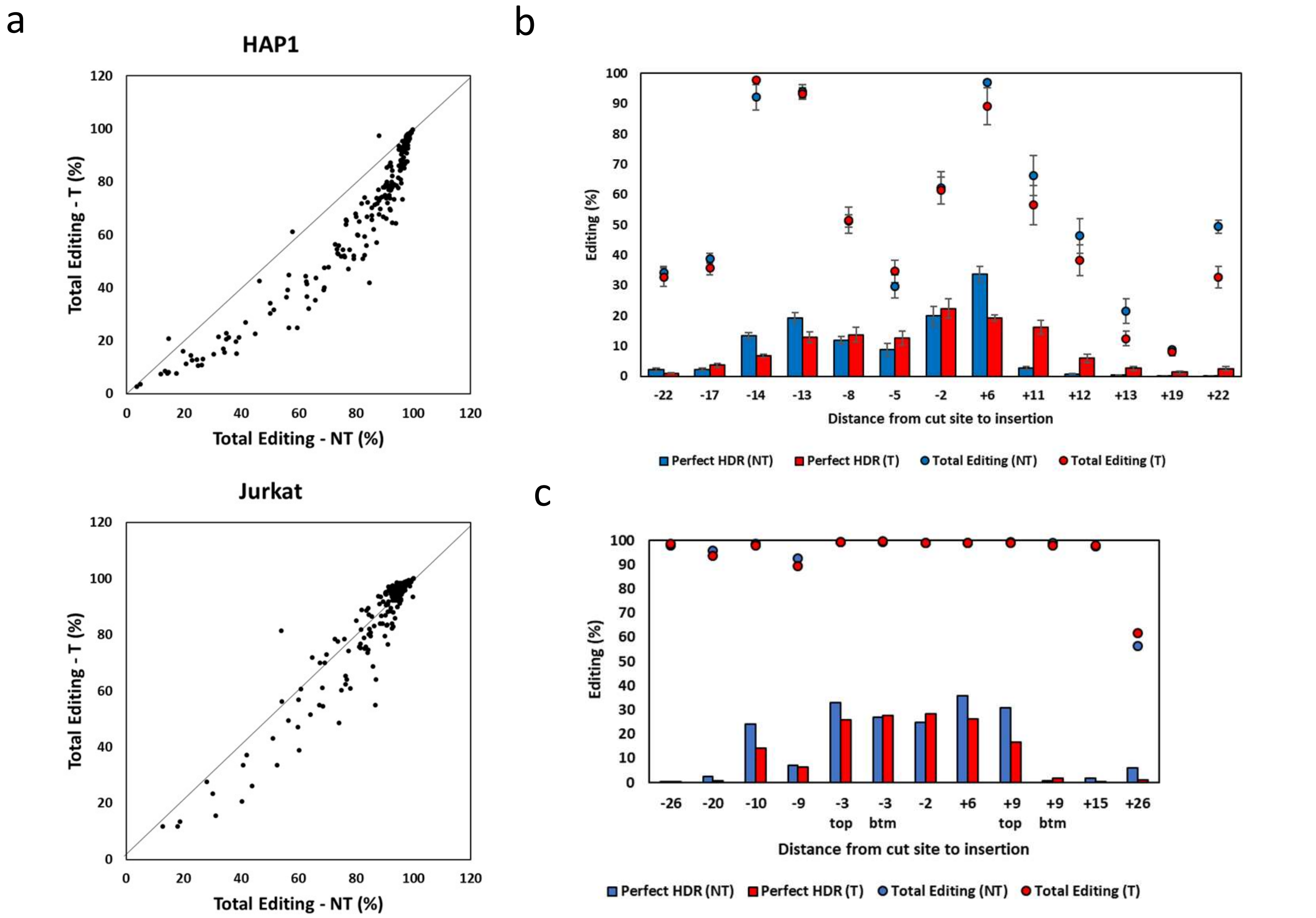

Supplemental Figure 1 (a) EcoRI restriction digest recognition site (GAATTC) was inserted at the Cas9 cleavage site of 254 genomic loci in Jurkat and 239 genomic loci in HAP1 cells using either the targeting (T) or non-targeting (NT) strand as the donor template. RNP complexes (Alt-R *S.p.* Cas9 Nuclease complexed with Alt-R CRISPR-Cas9 crRNA and tracrRNA) were delivered at 4 µM along with 4 µM Alt-R Cas9 Electroporation Enhancer and 3 µM donor template by nucleofection. Total editing was assessed via NGS. (b) Insertion of an EcoRI site before the stop codon of GAPDH in HEK293 cells using guides around the desired HDR insertion location. The cleavage sites and associated distance to the desired insertion location for each guide are indicated above the sequence shown. Both the T and NT strand were tested. RNP complexes (Alt-R *S.p.* Cas9 Nuclease complexed with Alt-R CRISPR-Cas9 crRNA and tracrRNA) were delivered at 2 µM along with 2 µM Alt-R Cas9 Electroporation Enhancer and 2 µM donor template by nucleofection. HDR and total editing were assessed via NGS. Data are represented as means ± S.E.M. of three technical replicates. (c) Insertion of an EcoRI site at the TNPO3 locus in HEK293 cells using guides around the desired HDR insertion location. The distance from each cleavage site to the desired insertion location for each guide are indicated on the x-axis. Two pairs of guides cut at the same location, but on opposite strands. The strand containing the guide is indicated as top or bottom (btm). Both the T and NT strand were tested. RNP complexes (Alt-R *S.p.* Cas9 Nuclease complexed with Alt-R CRISPR-Cas9 crRNA and tracrRNA) were delivered at 2 µM along with 2 µM Alt-R Cas9 Electroporation Enhancer and 2 µM donor template by nucleofection. HDR and total editing were assessed via NGS.

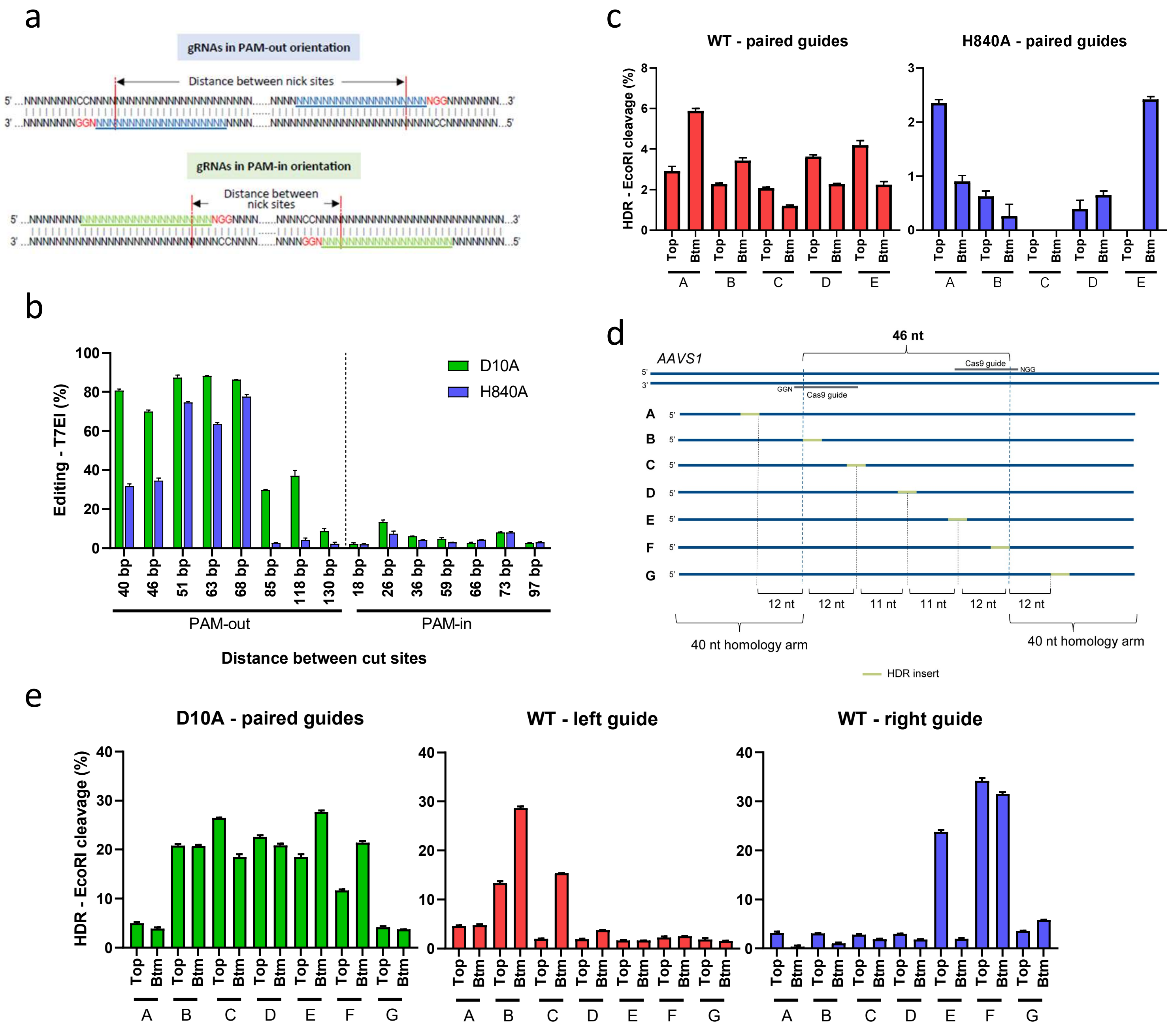

Supplemental Figure 2 HDR using Cas9 D10A nickase compared to WT Cas9 and Cas9 H840A nickase (a) Schematics showing gRNA pairs in PAM-out orientation (top panel) or PAM-in orientation (bottom panel). NGG PAMs are red, protospacers are underlined. Spacing between paired gRNAs is defined by the distance between targeted nick sites as indicated in the diagram. (b) RNP complexes consisting of gRNA pairs in different orientation and spacing targeting the *HPRT1* locus were delivered into HEK293 cells with Cas9 D10A or H840A proteins via lipofection and total editing was measured by T7EI cleavage. (c) HDR mediated by Cas9 WT (left panel) or Cas9 H840A (right panel) with paired gRNAs. Cas9 WT and Cas9 H840A were used in combination with gRNA pairs targeting *HPRT1* 51-nt PAM-out site. RNP complexes (Alt-R *S.p.* Cas9 Nuclease or Alt-R *S.p.* Cas9 H840A nickase complexed with Alt-R CRISPR-Cas9 crRNA and tracrRNA) were delivered at 4 µM (2 µM each RNP for nickase paired guides) along with 4 µM Alt-R Cas9 Electroporation Enhancer and 2 µM donor template by nucleofection. The same set of ssODNs homologous to either the top or bottom (Btm) strand as shown in Figure 2 were used to insert an EcoRI site along the target region. HDR was assessed via EcoRI cleavage. (d) Schematics of HDR donor oligos. HDR donor sequences were designed to insert an EcoRI site at 7 positions along the AAVS1 46-nt PAM-out target region. (e) HDR performance of donor oligos in HEK293 cells. Cas9 D10A with two guides, or Cas9 WT with each of the individual guides were used to induce double strand breaks. Bar charts are showing the HDR rate using indicated oligos homologous to either the top or bottom strand. Data are represented as means ± S.E.M of technical triplicates.

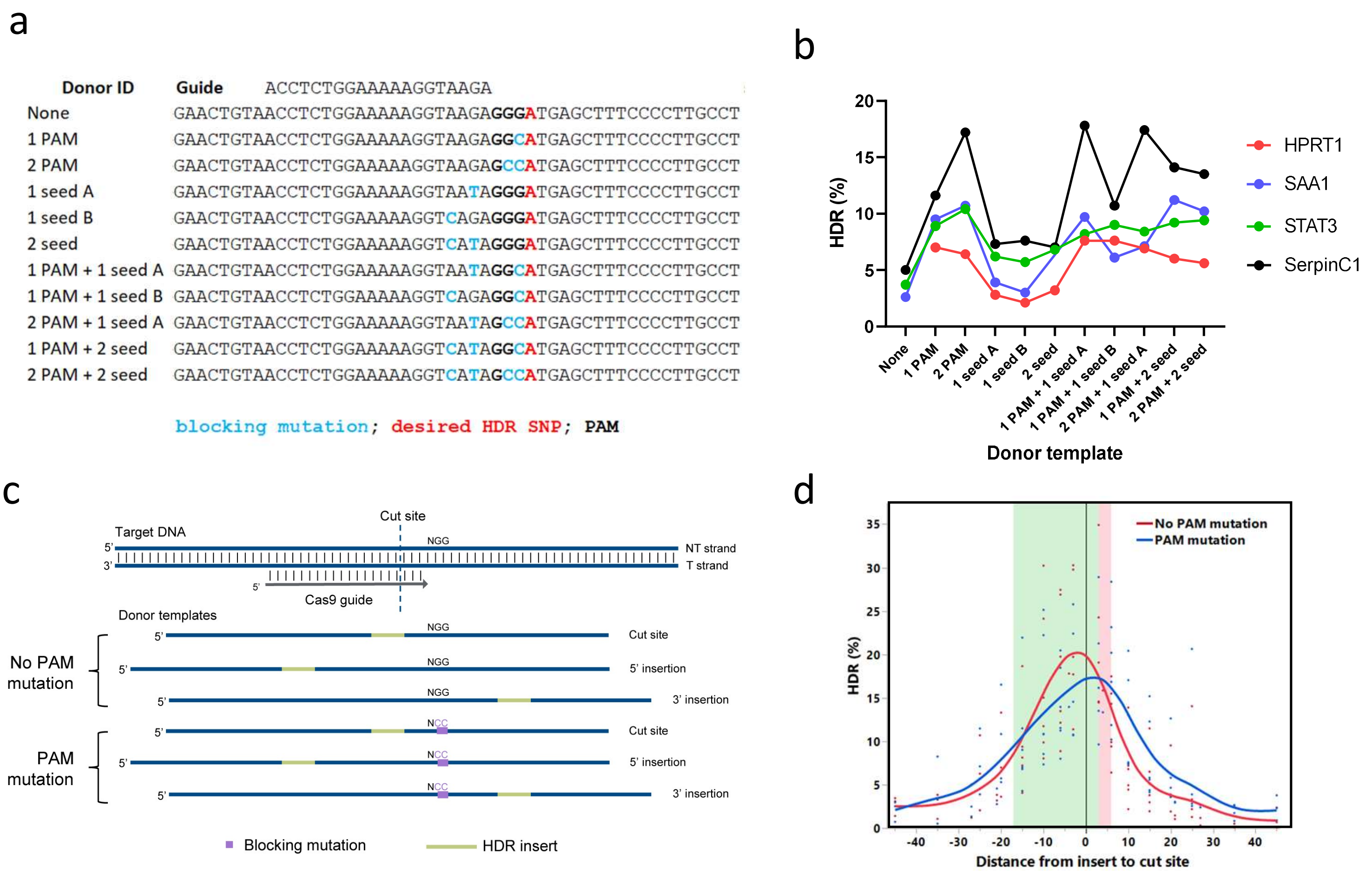

Supplemental Figure 3 (a) Example sequence of site SERPINC1 showing the various blocking mutation(s) tested. The Cas9 guide is shown above the sequence, and the PAM is bolded. The intended HDR mutation is in red and blocking mutations are shown in blue. (b) Donor templates for four target loci following the design strategy shown in panel A were delivered to Jurkat cells at 4 µM along with RNP complexes (Alt-R *S.p.* Cas9 Nuclease complexed with Alt-R CRISPR-Cas9 crRNA and tracrRNA) at 4 µM and with 4 µM Alt-R Cas9 Electroporation Enhancer by nucleofection. SNP conversion of the desired HDR mutation 3' of the PAM was determined by NGS. (c) Schematic representation of donor templates used to an EcoRI insert sequence positioned at varying distances from the Cas9 cleavage site, ranging up to 45 bases in either the 5' or 3' direction. Donor templates were designed with and without a mutation in the PAM ('NGG' to 'NCC') to prevent Cas9 re-cleavage. (d) HDR performance of donor templates for four genomic loci in Jurkat cells and two genomic loci in HEK293 cells. Negative values indicate the insertion was 5' (PAM-distal) of the cut site, whereas positive values indicate the insertion was 3' (PAM-proximal) of the cut site. RNP complexes (Alt-R *S.p.* Cas9 Nuclease complexed with Alt-R CRISPR-Cas9 crRNA and tracrRNA) were delivered at 4 µM along with 4 µM Alt-R Cas9 Electroporation Enhancer and 4 µM donor template by nucleofection. HDR rates were assessed via EcoRI cleavage of targeted amplicons.

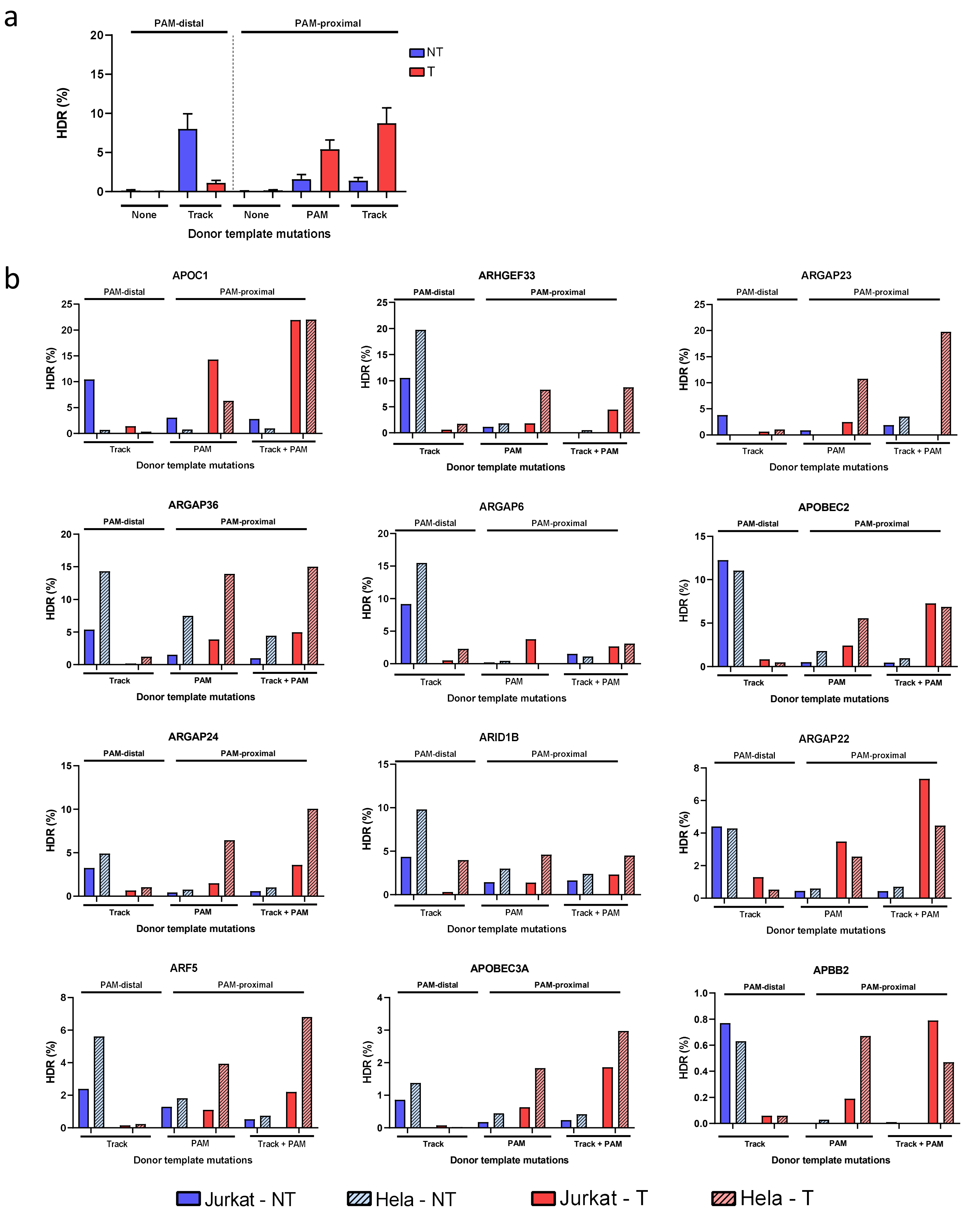

Supplemental Figure 4 (a) Donor templates creating an EcoRI insertion at the cut site or 20 bases PAM-proximal or PAM-distal to the Cas9 cut site for 12 genomic loci were tested in Hela cells as the targeting (T) or non-targeting (NT) strand. Donor templates for the PAM-distal insert contained repair track mutations. Donor templates for the PAM-proximal insert contained either PAM mutation or repair track plus PAM mutations. RNP complexes (Alt-R *S.p.* Cas9 Nuclease complexed with Alt-R CRISPR-Cas9 sgRNA) were delivered at 4 µM along with 4 µM Alt-R Cas9 Electroporation Enhancer and 3 µM donor template by nucleofection. Perfect HDR rates were determined by NGS. Data are represented as means ± S.E.M. (b) Individual plots of the 12 sites tested in HeLa and Jurkat cells.

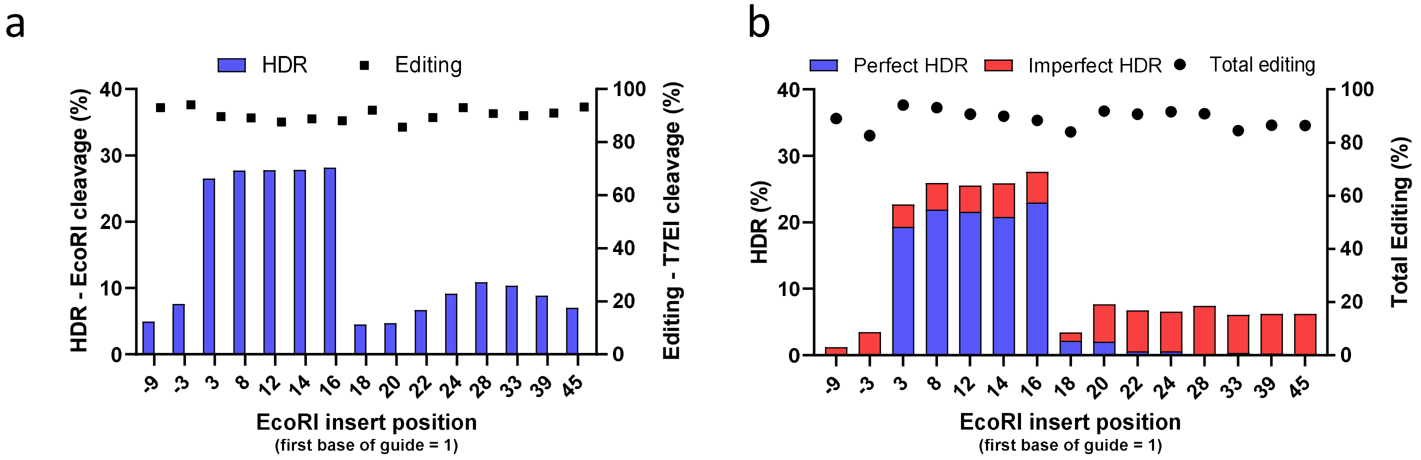

Supplemental Figure 5 Site HPRT 38330 from Figure 5B was delivered as an RNP complex (Alt-R *A.s.* Cas12a *Ultra* nuclease complexed with Alt-R CRISPR-Cas12a crRNA) at 2 µM along with 3 µM Alt-R Cpf1 Electroporation Enhancer and 3 µM Alt-R modified donor templates by nucleofection to Jurkat cells. HDR was measured by EcoRI cleavage (a) and NGS analysis (b) to determine the frequency of perfect HDR (blue) relative to imperfect HDR (red) and total editing (black dots).
