# Supplementary material for: Optimized design parameters for CRISPR Cas9 and Cas12a homology-directed repair
Source: Sci Rep. 2021 Sep 30;11:19482. doi: 10.1038/s41598-021-98965-y (PMC8484621; doi:10.1038/s41598-021-98965-y)
Supplement: Supplementary file 2 — Supplementary Table 1. [file 41598_2021_98965_MOESM2_ESM.pdf]

Supplemental Data Set

Optimized design parameters for CRISPR Cas9 and Cas12a homology-directed repair

Mollie S. Schubert[1], Bernice Thommandru[1], Jessica Woodley[1], Rolf Turk[1], Shuqi Yan[1,2], Gavin Kurgan[1], Matthew S. McNeill[1] and Garrett R. Rettig[1,*]
[1] Integrated DNA Technologies, Inc., Coralville, IA, 52241, USA
[2] Present address: BGI Genomics, BGI-Shenzhen, Shenzhen 518083, China.

Figure	Target	Enzyme	GuideID	Guide protospacer	gRNA format	DonorID	Donor template sequence	Donor modification	T7EI/RFLP Forward primer	T7EI/RFLP Reverse primer	NGS forward primer	NGS reverse primer
1B, S1A	A1CF	Cas9	Hs.Cas9.A1CF.1	TAATCGGCAGTTGTCCACAC	Alt-R Cas9 crRNA:tra	A1CF-NT	G*T*TTTTGGGATGCCCCCAA	PS modified	NA	NA	acactctttccctacacgacgctcttccgatctCACCTTCAGTAACCTTTTTCATCT	gtgactggagttcagacgtgtgctcttccgatctTCAACACTATGAACCCAAACATC
1B, S1A	A3GALT2	Cas9	Hs.Cas9.A3GALT2.1	GCGCTGGCAAGACGTGTCGA	Alt-R Cas9 crRNA:tra	A3GALT2-NT	G*T*GGAGCGCGTGGCGCGC	PS modified	NA	NA	acactctttccctacacgacgctcttccgatctAAAAGTGCCGCTGAAGTG	gtgactggagttcagacgtgtgctcttccgatctTGTACTACGTGTTCACCGAG
1B, S1A	AADACL3	Cas9	Hs.Cas9.AADACL3.1	GGCATCGTGTACTACCACGG	Alt-R Cas9 crRNA:tra	AADACL3-NT	C*A*TCCACCTGCACCCTGAA	PS modified	NA	NA	acactctttccctacacgacgctcttccgatctGCTCAGAATCTGTTCTATGCC	gtgactggagttcagacgtgtgctcttccgatctGGTCTGAGAAAATGGTTCTTACT
1B, S1A	AAGAB	Cas9	Hs.Cas9.AAGAB.1	CAGCTGGTGACTAACGCACA	Alt-R Cas9 crRNA:tra	AAGAB-NT	G*G*TCTCCTGAGAAGACGG	PS modified	NA	NA	acactctttccctacacgacgctcttccgatctCGCCTATCTACTCACGTTG	gtgactggagttcagacgtgtgctcttccgatctGGTCATTCCGAGAACGC
1B, S1A	ABCA12	Cas9	Hs.Cas9.ABCA12.1	CCACGTTTTGCAACTAACGA	Alt-R Cas9 crRNA:tra	ABCA12-NT	T*G*CTGGTTGTGCAGAAGG	PS modified	NA	NA	acactctttccctacacgacgctcttccgatctACAGCAGATGTTTAACAGACTT	gtgactggagttcagacgtgtgctcttccgatctCTGCAAACCTTGAAACAGAAT
1B, S1A	ABCA9	Cas9	Hs.Cas9.ABCA9.1	GCACAAATTGTCGTCCTGAC	Alt-R Cas9 crRNA:tra	ABCA9-NT	T*A*ATGGCTCTTATTGTAAA	PS modified	NA	NA	acactctttccctacacgacgctcttccgatctATTAACCAACTCACCAAAGACAG	gtgactggagttcagacgtgtgctcttccgatctGTTTGATGTATGCTGGCTTCAT
1B, S1A	ABCB11	Cas9	Hs.Cas9.ABCB11.1	CGCATGACCTCGACCATCTG	Alt-R Cas9 crRNA:tra	ABCB11-NT	A*C*CAAATGGCCCTTTTCAT	PS modified	NA	NA	acactctttccctacacgacgctcttccgatctAGAGGGCTGACAGAAATAATAAC	gtgactggagttcagacgtgtgctcttccgatctAAAATCAATGATGCCATAGCTGA
1B, S1A	ABCB9	Cas9	Hs.Cas9.ABCB9.1	ACCCTCGTGTGCCTCTTCGT	Alt-R Cas9 crRNA:tra	ABCB9-NT	T*G*CGGGCCTCGTGGCTGG	PS modified	NA	NA	acactctttccctacacgacgctcttccgatctGCCGAGTGAAATGTACGTC	gtgactggagttcagacgtgtgctcttccgatctCATTGGTGTGGCCAAGAA
1B, S1A	ABCC10	Cas9	Hs.Cas9.ABCC10.1	TGCCAGATAGCACCGTCCAA	Alt-R Cas9 crRNA:tra	ABCC10-NT	C*C*CACCAGCTTCAGCAGTC	PS modified	NA	NA	acactctttccctacacgacgctcttccgatctCACAGACTGCAGCCAAC	gtgactggagttcagacgtgtgctcttccgatctGAATCCCAACATGGTCCC
1B, S1A	ABCD1	Cas9	Hs.Cas9.ABCD1.1	GGCGGGCCACATACACCGAC	Alt-R Cas9 crRNA:tra	ABCD1-NT	G*C*AGCGGGCCAGCCTTCC	PS modified	NA	NA	acactctttccctacacgacgctcttccgatctCAAAGCTGGCATGAACC	gtgactggagttcagacgtgtgctcttccgatctCGATGAGGAGCCACTG
1B, S1A	ABCD3	Cas9	Hs.Cas9.ABCD3.1	ACTCGACTTCGAAGACCCAT	Alt-R Cas9 crRNA:tra	ABCD3-NT	T*G*GTTGTTTCTGGGCTATT	PS modified	NA	NA	acactctttccctacacgacgctcttccgatctCCCAGAACTTTGTGTATCTTTCT	gtgactggagttcagacgtgtgctcttccgatctGTCTTTACCTGTTTGTGATGAGC
1B, S1A	ABCD4	Cas9	Hs.Cas9.ABCD4.1	CTGGTAAGTGTAGTAGACGA	Alt-R Cas9 crRNA:tra	ABCD4-NT	G*G*GAGGCTTCACCTTTGG	PS modified	NA	NA	acactctttccctacacgacgctcttccgatctTAAGCTTCTCTTGGACCTTGA	gtgactggagttcagacgtgtgctcttccgatctTCCAGCTACTCTGTGTCATT
1B, S1A	ABCG2	Cas9	Hs.Cas9.ABCG2.1	ACCTGGTCTCAACGCCATCC	Alt-R Cas9 crRNA:tra	ABCG2-NT	T*G*TGTTTACAGTGGGATCA	PS modified	NA	NA	acactctttccctacacgacgctcttccgatctCACATTAAAAGTGCACAGAAAACG	gtgactggagttcagacgtgtgctcttccgatctCAGTGCCTACCCTAAATTAATAGAA
1B, S1A	ABCG4	Cas9	Hs.Cas9.ABCG4.1	TCGTGTGGGAGCACGACATC	Alt-R Cas9 crRNA:tra	ABCG4-NT	C*C*CGCCAGAGAGCAGGGC	PS modified	NA	NA	acactctttccctacacgacgctcttccgatctAGACTCCGAAGCTGACCT	gtgactggagttcagacgtgtgctcttccgatctTTGTTGACCAGCTCCAGG
1B, S1A	ABCG5	Cas9	Hs.Cas9.ABCG5.1	CATGTGGCAGACCGACTGAT	Alt-R Cas9 crRNA:tra	ABCG5-NT	T*C*ATGGCAGAGCTGAGTCT	PS modified	NA	NA	acactctttccctacacgacgctcttccgatctGCTAGTAACAGTTCTGGGTG	gtgactggagttcagacgtgtgctcttccgatctCCCATGAATTGAAATAGCAGC
1B, S1A	ABHD11	Cas9	Hs.Cas9.ABHD11.1	CGTGCAAAAAGACGACGGCC	Alt-R Cas9 crRNA:tra	ABHD11-NT	G*T*TAGTTTTGCTGCCGAAG	PS modified	NA	NA	acactctttccctacacgacgctcttccgatctCAAGATCTTGGCGATGGA	gtgactggagttcagacgtgtgctcttccgatctTCAGACTCTGACCTCGATC
1B, S1A	ABHD15	Cas9	Hs.Cas9.ABHD15.1	ATACATCCGCTTCCGACACC	Alt-R Cas9 crRNA:tra	ABHD15-NT	T*C*CGACCTCAAGGAGGCG	PS modified	NA	NA	acactctttccctacacgacgctcttccgatctACTCGCCCAGGTAGGA	gtgactggagttcagacgtgtgctcttccgatctGCTACTACCCGGTCATCTT
1B, S1A	ABHD5	Cas9	Hs.Cas9.ABHD5.1	TTGGACGAAGTAGTAGACCC	Alt-R Cas9 crRNA:tra	ABHD5-NT	C*T*ATGCTTTTGACCTATTG	PS modified	NA	NA	acactctttccctacacgacgctcttccgatctGGCACTGAATTTTGGAGATCTTTG	gtgactggagttcagacgtgtgctcttccgatctCACATCTCCACTCTTCAATGGATT
1B, S1A	ABHD6	Cas9	Hs.Cas9.ABHD6.1	GATTGTCAGTTGAGTACTGC	Alt-R Cas9 crRNA:tra	ABHD6-NT	T*T*CTTTGAGCCGTTGTACA	PS modified	NA	NA	acactctttccctacacgacgctcttccgatctTATTAACTCTGGGCTGCTGT	gtgactggagttcagacgtgtgctcttccgatctGACATAGGAGCAGAGCTGAAG
1B, S1A	ABI3	Cas9	Hs.Cas9.ABI3.1	GCCTGGACGACATTGGCCAT	Alt-R Cas9 crRNA:tra	ABI3-NT	C*A*GGAGACCCCTCAACTTT	PS modified	NA	NA	acactctttccctacacgacgctcttccgatctAAGGTCATCGCCCCAGA	gtgactggagttcagacgtgtgctcttccgatctGGGAAAGAGGAGGGCC
1B, S1A	ABL2	Cas9	Hs.Cas9.ABL2.1	AGGGACGTGTGTATCACTAC	Alt-R Cas9 crRNA:tra	ABL2-NT	G*C*TGTCCATCTCGCTCAGG	PS modified	NA	NA	acactctttccctacacgacgctcttccgatctTGACCAGGGAGTCTTACCTT	gtgactggagttcagacgtgtgctcttccgatctCAGCAGTCTAATCAATGGCAG
1B, S1A	ABLIM2	Cas9	Hs.Cas9.ABLIM2.1	TCGACACGCCGGATGCCAGA	Alt-R Cas9 crRNA:tra	ABLIM2-NT	C*T*GTCTTCAGTTCTGGCTG	PS modified	NA	NA	acactctttccctacacgacgctcttccgatctCCACCAACCTTGTTTCTGT	gtgactggagttcagacgtgtgctcttccgatctCCCTGCAACATGGGAAATAA
1B, S1A	ABRAXAS1	Cas9	Hs.Cas9.ABRAXAS1.1	AAGCTGCTCTACTCATCGAC	Alt-R Cas9 crRNA:tra	ABRAXAS1-NT	T*T*AACACCAAGTATAATAA	PS modified	NA	NA	acactctttccctacacgacgctcttccgatctCAGTGACTGGCCAACATTTA	gtgactggagttcagacgtgtgctcttccgatctCTGCTTCACAAAAACTTGCAG
1B, S1A	ACAT1	Cas9	Hs.Cas9.ACAT1.1	TCAAGCTTTACCCCACCATA	Alt-R Cas9 crRNA:tra	ACAT1-NT	G*C*CCGTCTTTTACAATCAA	PS modified	NA	NA	acactctttccctacacgacgctcttccgatctGGGTTTGCAAAATATTTGTATTAACATT	gtgactggagttcagacgtgtgctcttccgatctCAGTATTGGACATTAGATAGCATTTAT
1B, S1A	ACAT2	Cas9	Hs.Cas9.ACAT2.1	GCCGCCGAGACGATGACCAC	Alt-R Cas9 crRNA:tra	ACAT2-NT	G*C*CACTCACCTATGATGGT	PS modified	NA	NA	acactctttccctacacgacgctcttccgatctCGGCAGGAGAAGCAAGAT	gtgactggagttcagacgtgtgctcttccgatctCATACGCCCCTCTCCTACA
1B, S1A	ACCSL	Cas9	Hs.Cas9.ACCSL.1	GGATAGGTCGCGGTTGACAA	Alt-R Cas9 crRNA:tra	ACCSL-NT	A*C*AGAGATGTCAATCCCAC	PS modified	NA	NA	acactctttccctacacgacgctcttccgatctAAGTGCCTCTTCCTTCTGAG	gtgactggagttcagacgtgtgctcttccgatctTAGGCATTGTAGTCCTGGAAG
1B, S1A	ACER2	Cas9	Hs.Cas9.ACER2.1	GCATCTGACCCAAGAAACTA	Alt-R Cas9 crRNA:tra	ACER2-NT	C*C*AAAGGACTGCAAGTTCA	PS modified	NA	NA	acactctttccctacacgacgctcttccgatctGTTGTAATTGATTCTAGGAATTGGAT	gtgactggagttcagacgtgtgctcttccgatctCATTCCGAAAGATCTTTGGTAGATA
1B, S1A	ACKR3	Cas9	Hs.Cas9.ACKR3.1	TTGCACGTGAGCTCGCCCAT	Alt-R Cas9 crRNA:tra	ACKR3-NT	T*G*GAGAAGATGAGGTGTG	PS modified	NA	NA	acactctttccctacacgacgctcttccgatctCTGCTACATCTTGAACCTGG	gtgactggagttcagacgtgtgctcttccgatctTTGGTGAAGTAGGTGATGGA
1B, S1A	ACOT13	Cas9	Hs.Cas9.ACOT13.1	GCAATAGGCACTCTCCACGG	Alt-R Cas9 crRNA:tra	ACOT13-NT	A*A*GTAGAAGAAGAGCATA	PS modified	NA	NA	acactctttccctacacgacgctcttccgatctCTTAGATTACTCTTGTCTCTGCTG	gtgactggagttcagacgtgtgctcttccgatctGCAGAGCCATTGTTGATATGTTAT
1B, S1A	ACOT2	Cas9	Hs.Cas9.ACOT2.1	GAGCGTCCCGGCTGTACCAA	Alt-R Cas9 crRNA:tra	ACOT2-NT	G*G*CCTCATCTCCTGCTGTC	PS modified	NA	NA	acactctttccctacacgacgctcttccgatctCCCCATTCAGTTGTTCTCAG	gtgactggagttcagacgtgtgctcttccgatctAACCCTAATGATCTGACCAAC
1B, S1A	ACP2	Cas9	Hs.Cas9.ACP2.1	GTCAGGATGACCGAATACGT	Alt-R Cas9 crRNA:tra	ACP2-NT	A*C*CTGGAGCCCTGGCTTAT	PS modified	NA	NA	acactctttccctacacgacgctcttccgatctCATTCAACCACTTCCCTGT	gtgactggagttcagacgtgtgctcttccgatctGTAATGAGAGATGGGCTCAC
1B, S1A	ACP5	Cas9	Hs.Cas9.ACP5.1	TTTCCGGCTAGCACGTACCA	Alt-R Cas9 crRNA:tra	ACP5-NT	A*G*ACATTGCCAAGGTGGT	PS modified	NA	NA	acactctttccctacacgacgctcttccgatctTAGAGTATGCAATCTGGGCA	gtgactggagttcagacgtgtgctcttccgatctCTACAGGAGACCTTTGAGGA
1B, S1A	ACR	Cas9	Hs.Cas9.ACR.1	ATGAAGCGCCCACACGAAAT	Alt-R Cas9 crRNA:tra	ACR-NT	A*G*TGGGGCAGGCAGCCCG	PS modified	NA	NA	acactctttccctacacgacgctcttccgatctACAGAGGGAAATGACATTGC	gtgactggagttcagacgtgtgctcttccgatctTTCTATATATCCCCAGCCGG
1B, S1A	ACRBP	Cas9	Hs.Cas9.ACRBP.1	AAGAAGCGTTCGTATTCGGT	Alt-R Cas9 crRNA:tra	ACRBP-NT	T*C*CAGGTTGGAGTCAGCA	PS modified	NA	NA	acactctttccctacacgacgctcttccgatctCAGGTAGTCTCTGCCTTC	gtgactggagttcagacgtgtgctcttccgatctCAGGATTCGACTCAGGC
1B, S1A	ACSL5	Cas9	Hs.Cas9.ACSL5.1	GGCTTGTTACACGTACTCTA	Alt-R Cas9 crRNA:tra	ACSL5-NT	G*T*ACAGTGGATCATCTCCG	PS modified	NA	NA	acactctttccctacacgacgctcttccgatctCCTCCAGTCCTTACTTGAACTT	gtgactggagttcagacgtgtgctcttccgatctTATGTACGATGGCTTCTGGTC
1B, S1A	ACSM4	Cas9	Hs.Cas9.ACSM4.1	AATACAATGGACTCCACCGC	Alt-R Cas9 crRNA:tra	ACSM4-NT	T*T*GTCTTAAGGTCAGGACA	PS modified	NA	NA	acactctttccctacacgacgctcttccgatctCTGACAGCAAAAGACATCCT	gtgactggagttcagacgtgtgctcttccgatctACCCATTCCAGCTTTGTG
1B, S1A	ACSS2	Cas9	Hs.Cas9.ACSS2.1	GTCTCTATGTGAACGGATCT	Alt-R Cas9 crRNA:tra	ACSS2-NT	T*A*GTTTGCAGGCTTCTCTT	PS modified	NA	NA	acactctttccctacacgacgctcttccgatctCCTCAGACTTTCTTCCCTTC	gtgactggagttcagacgtgtgctcttccgatctGTAGCCCCAAATACCAATGA
1B, S1A	ACTA2	Cas9	Hs.Cas9.ACTA2.1	TGGGACGTCCCACAATGGAT	Alt-R Cas9 crRNA:tra	ACTA2-NT	C*A*TAATTTTCCTCACCTGAT	PS modified	NA	NA	acactctttccctacacgacgctcttccgatctCAGAGGAACCTAATCTGTGT	gtgactggagttcagacgtgtgctcttccgatctCTCCAGCTATGTGTGAAGAA
1B, S1A	ACTN1	Cas9	Hs.Cas9.ACTN1.1	GTGCTTTGATCCACCGACAC	Alt-R Cas9 crRNA:tra	ACTN1-NT	C*T*GGAAGGATGGCCTCGG	PS modified	NA	NA	acactctttccctacacgacgctcttccgatctATACCTTCCGCAGCTTCC	gtgactggagttcagacgtgtgctcttccgatctAGCCTCATTTACCAGCCTG
1B, S1A	ACTRT1	Cas9	Hs.Cas9.ACTRT1.1	GAGGGCTCGGTCATAAGTAC	Alt-R Cas9 crRNA:tra	ACTRT1-NT	C*T*CGAATTTCCCTAGGATT	PS modified	NA	NA	acactctttccctacacgacgctcttccgatctCACCGCATGATTAGACAGGTA	gtgactggagttcagacgtgtgctcttccgatctTATGAGGCCCTACATTTGCA
1B, S1A	ACVR1C	Cas9	Hs.Cas9.ACVR1C.1	TGTAGGAGCACTGTCGACCC	Alt-R Cas9 crRNA:tra	ACVR1C-NT	C*A*CATTTGGTCTCTTTTTCT	PS modified	NA	NA	acactctttccctacacgacgctcttccgatctCAGCATTTACCAGATTGCACT	gtgactggagttcagacgtgtgctcttccgatctGCTGGCCATCATTATTACTGTG
1B, S1A	ACVR2A	Cas9	Hs.Cas9.ACVR2A.1	ACTGGTGTTGAACCGTGTTA	Alt-R Cas9 crRNA:tra	ACVR2A-NT	G*G*GAAAAAGACAGAACCA	PS modified	NA	NA	acactctttccctacacgacgctcttccgatctTAGGTGCTATACTTGGTAGATCAGAAA	gtgactggagttcagacgtgtgctcttccgatctCAGAAATATTCTTCCAGGTAGCAAAA
1B, S1A	ACY3	Cas9	Hs.Cas9.ACY3.1	CACCTCATATGGGTCGTCCG	Alt-R Cas9 crRNA:tra	ACY3-NT	T*G*GTTCAGCTCTCGGGCTC	PS modified	NA	NA	acactctttccctacacgacgctcttccgatctGCAGGTCAAGGACAAAGT	gtgactggagttcagacgtgtgctcttccgatctCCCCTTAAACCACTGAAGA
1B, S1A	ADAM30	Cas9	Hs.Cas9.ADAM30.1	TACGAGTCAAACTCCCCTTC	Alt-R Cas9 crRNA:tra	ADAM30-NT	G*C*TTCTCAGGAATGGTGA	PS modified	NA	NA	acactctttccctacacgacgctcttccgatctCTTTTAACTGCAGTAGGTAGGA	gtgactggagttcagacgtgtgctcttccgatctGACAATGCTCCTTAAGTCTCTT
1B, S1A	ADAM33	Cas9	Hs.Cas9.ADAM33.1	CCACGTAGTTGGCGACTTCC	Alt-R Cas9 crRNA:tra	ADAM33-NT	C*T*CCCCGCCGCCCCCAACC	PS modified	NA	NA	acactctttccctacacgacgctcttccgatctTCTTCCCCAAACCCCAC	gtgactggagttcagacgtgtgctcttccgatctTGCCTTCCCAGTTCTTGA
1B, S1A	ADAM7	Cas9	Hs.Cas9.ADAM7.1	GCCAGTATCAGTACGTGTAA	Alt-R Cas9 crRNA:tra	ADAM7-NT	T*A*GTACACGAATATGATTC	PS modified	NA	NA	acactctttccctacacgacgctcttccgatctTCATTGTTTTTACCAAGGATCCAT	gtgactggagttcagacgtgtgctcttccgatctTAAATCATAGGCTACAGCTGAAA
1B, S1A	ADAMTS12	Cas9	Hs.Cas9.ADAMTS12.1	CTCGGACTGGACCCACCACG	Alt-R Cas9 crRNA:tra	ADAMTS12-NT	C*A*AAAAATGCCCACTGGC	PS modified	NA	NA	acactctttccctacacgacgctcttccgatctGAAATTCTGTAGTACACCCAGTC	gtgactggagttcagacgtgtgctcttccgatctGGCTTTGCTTTACTACCAATCTC
1B, S1A	ADCY2	Cas9	Hs.Cas9.ADCY2.1	GACGCTAAGCACGATGGTGT	Alt-R Cas9 crRNA:tra	ADCY2-NT	C*C*TCCCGGTGTTGCAGACA	PS modified	NA	NA	acactctttccctacacgacgctcttccgatctGCCCGTAGGTATCGTTCTTC	gtgactggagttcagacgtgtgctcttccgatctTGGGATGTGGAAAGTCATTCT
1B, S1A	ADGRB3	Cas9	Hs.Cas9.ADGRB3.1	TAACCGAACATGTGCTCCAC	Alt-R Cas9 crRNA:tra	ADGRB3-NT	C*A*CCTGCGACCCTTGACCA	PS modified	NA	NA	acactctttccctacacgacgctcttccgatctCATGTTTATTTGTTGTCTTGCACG	gtgactggagttcagacgtgtgctcttccgatctCGTAAGGTGATACACAAGTTCTG
1B, S1A	ADGRF1	Cas9	Hs.Cas9.ADGRF1.1	TCAAGGTTTTGAGTCGGTTC	Alt-R Cas9 crRNA:tra	ADGRF1-NT	C*T*TAAAAAAGCATATGAAA	PS modified	NA	NA	acactctttccctacacgacgctcttccgatctGCCCTTCTAAGACCATTGTGTTA	gtgactggagttcagacgtgtgctcttccgatctGCTCAAATGTCTTTCTGAAGTACG
1B, S1A	ADGRG1	Cas9	Hs.Cas9.ADGRG1.1	ACCGGATCAACGCCACGGTG	Alt-R Cas9 crRNA:tra	ADGRG1-NT	G*G*ACATGGTGTCCTTCGA	PS modified	NA	NA	acactctttccctacacgacgctcttccgatctTGGAGTCGAAACTGACCT	gtgactggagttcagacgtgtgctcttccgatctGAGTGGATGTGCAGGTC
1B, S1A	ADGRL1	Cas9	Hs.Cas9.ADGRL1.1	CTACGGACGCGCATCAAGAG	Alt-R Cas9 crRNA:tra	ADGRL1-NT	C*G*CGCAACATCGTCAAGTA	PS modified	NA	NA	acactctttccctacacgacgctcttccgatctGAGGTGTCATGGTAGTTGG	gtgactggagttcagacgtgtgctcttccgatctTGCCGTCTTCTACAACAA
1B, S1A	ADGRL2	Cas9	Hs.Cas9.ADGRL2.1	TATTAAAGTATCGGTACGAT	Alt-R Cas9 crRNA:tra	ADGRL2-NT	A*A*ATCTTCTAAAGAAGCAT	PS modified	NA	NA	acactctttccctacacgacgctcttccgatctGACCTTGAAAGCAATTGTGGA	gtgactggagttcagacgtgtgctcttccgatctCCATCATACACCACAAATCCA
1B, S1A	ADGRL3	Cas9	Hs.Cas9.ADGRL3.1	TTTGAGTCCGACCACCAATC	Alt-R Cas9 crRNA:tra	ADGRL3-NT	G*A*GTATACCAGAGTGAAC	PS modified	NA	NA	acactctttccctacacgacgctcttccgatctCCTTCCAACAGTTTTTCTTTGTC	gtgactggagttcagacgtgtgctcttccgatctGATGAATACTCAGTCAGGGTATCA
1B, S1A	ADI1	Cas9	Hs.Cas9.ADI1.1	CTACGAGGAGCATTTGCACT	Alt-R Cas9 crRNA:tra	ADI1-NT	C*T*TCTGCACTAGATTAAGA	PS modified	NA	NA	acactctttccctacacgacgctcttccgatctGAAGTACCCACTGCCATC	gtgactggagttcagacgtgtgctcttccgatctGTGACAGGTCTCTGGTAGA
1B, S1A	ADIRF	Cas9	Hs.Cas9.ADIRF.1	CTTGCAGGACCTGAAGCAAC	Alt-R Cas9 crRNA:tra	ADIRF-NT	C*C*CGAAGCCATGGCAAGC	PS modified	NA	NA	acactctttccctacacgacgctcttccgatctAGCTGCTCTTGACGACT	gtgactggagttcagacgtgtgctcttccgatctTAGGTCGCGCGCTATC
1B, S1A	ADNP	Cas9	Hs.Cas9.ADNP.1	CCTGATAGCCTATACGTTCA	Alt-R Cas9 crRNA:tra	ADNP-NT	T*G*TGTGCCCAATCATGGCA	PS modified	NA	NA	acactctttccctacacgacgctcttccgatctGCAATTAGCATCAAGGGTTTG	gtgactggagttcagacgtgtgctcttccgatctCCTTTTCATGCCAAAGTCCTAT
1B, S1A	ADPGK	Cas9	Hs.Cas9.ADPGK.1	AGCCCAAGGGAAGTCACCGC	Alt-R Cas9 crRNA:tra	ADPGK-NT	G*A*AATAACAGCTCCTGTTC	PS modified	NA	NA	acactctttccctacacgacgctcttccgatctCTTTTACTCCTCCCATGTTCTTT	gtgactggagttcagacgtgtgctcttccgatctGTCATAAGACATCTTAGGAGCCA
1B, S1A	ADRA2C	Cas9	Hs.Cas9.ADRA2C.1	GCGGCCTCAACGACGAGACC	Alt-R Cas9 crRNA:tra	ADRA2C-NT	C*G*ACGGCGCCGCCTACCC	PS modified	NA	NA	acactctttccctacacgacgctcttccgatctTGTGGCTCATCTCGGC	gtgactggagttcagacgtgtgctcttccgatctATGATGAGGCAGGGCG
1B, S1A	ADRB3	Cas9	Hs.Cas9.ADRB3.1	CAACGTGTTCGTGACTTCGC	Alt-R Cas9 crRNA:tra	ADRB3-NT	A*C*TCCGAGACTCCAGACCA	PS modified	NA	NA	acactctttccctacacgacgctcttccgatctACCACCAGGAGTCCCAT	gtgactggagttcagacgtgtgctcttccgatctAACCTGCTGGTCATCGTG
1B, S1A	AEBP1	Cas9	Hs.Cas9.AEBP1.1	GAACTCCTCGATCTCGTCGT	Alt-R Cas9 crRNA:tra	AEBP1-NT	A*G*CTCTGACAGGAAGCCC	PS modified	NA	NA	acactctttccctacacgacgctcttccgatctCTCAGCTGCCTCCTGG	gtgactggagttcagacgtgtgctcttccgatctGGGCTCAGGTTCTAGCT
1B, S1A	AFAP1	Cas9	Hs.Cas9.AFAP1.1	ATAAGAGCTGCTCATCGCAT	Alt-R Cas9 crRNA:tra	AFAP1-NT	T*C*CTCCTCTTCATCATACGA	PS modified	NA	NA	acactctttccctacacgacgctcttccgatctGTTTTCTTCCCCTTCCCATC	gtgactggagttcagacgtgtgctcttccgatctGGTCCCTTTCTCATTCAGTTA
1B, S1A	AFG3L2	Cas9	Hs.Cas9.AFG3L2.1	AAGGCGATGATGAGCACCGT	Alt-R Cas9 crRNA:tra	AFG3L2-NT	C*T*CTTCTGATGGTGTAGAG	PS modified	NA	NA	acactctttccctacacgacgctcttccgatctCCTTGGCAGTGGTTTCTC	gtgactggagttcagacgtgtgctcttccgatctAGTCCTAACCCGTGTTGC
1B, S1A	AGAP3	Cas9	Hs.Cas9.AGAP3.1	TGGTGCACCGCTATCTGACG	Alt-R Cas9 crRNA:tra	AGAP3-NT	C*C*TGTCTAGCGGGAAGTCA	PS modified	NA	NA	acactctttccctacacgacgctcttccgatctTCTGCCCTCTGACCCA	gtgactggagttcagacgtgtgctcttccgatctGGCCTGTGACGCTCAC
1B, S1A	AGAP6	Cas9	Hs.Cas9.AGAP6.1	TGGAATATTGTGCTTGACTC	Alt-R Cas9 crRNA:tra	AGAP6-NT	C*A*CCTTCTGTTTGAGAGTT	PS modified	NA	NA	acactctttccctacacgacgctcttccgatctCTCCCTCTATACATATAGCTTTGGA	gtgactggagttcagacgtgtgctcttccgatctCTCAAAGAAGCCTAAACAAAGTAC
1B, S1A	AGAP9	Cas9	Hs.Cas9.AGAP9.1	TGGTGGTGCTGGAGATACCG	Alt-R Cas9 crRNA:tra	AGAP9-NT	G*G*GGGGCGGGTTGAGCTT	PS modified	NA	NA	acactctttccctacacgacgctcttccgatctTTCAAAGTGCCACGTTTG	gtgactggagttcagacgtgtgctcttccgatctCTAGCCACATCAGCCT
1B, S1A	AGBL5	Cas9	Hs.Cas9.AGBL5.1	ATTCCCATGTTGAACCCCGA	Alt-R Cas9 crRNA:tra	AGBL5-NT	G*T*CGCCTCTTCGTCTTTAA	PS modified	NA	NA	acactctttccctacacgacgctcttccgatctATGGCTTTCTGGACTTCATC	gtgactggagttcagacgtgtgctcttccgatctTATTAGGGTGCAAGAGGACTG
1B, S1A	AGO1	Cas9	Hs.Cas9.AGO1.1	GATCGACGTGTACCACTACG	Alt-R Cas9 crRNA:tra	AGO1-NT	T*A*CTTTGAGGTGGACATCC	PS modified	NA	NA	acactctttccctacacgacgctcttccgatctGAAACCAATCAAGCTCCTGG	gtgactggagttcagacgtgtgctcttccgatctTGTGCATCACTTACCGGTT
1B, S1A	AGO3	Cas9	Hs.Cas9.AGO3.1	GTAGCACCACATCAACGGCA	Alt-R Cas9 crRNA:tra	AGO3-NT	C*C*ACTTCATGGAGGGCAG	PS modified	NA	NA	acactctttccctacacgacgctcttccgatctGCCACTGGAATTAGACAAGC	gtgactggagttcagacgtgtgctcttccgatctAAAGGTTTAACCAAGGGAAGC
1B, S1A	AGPAT1	Cas9	Hs.Cas9.AGPAT1.1	CATCGACCGGAAGCGCACGG	Alt-R Cas9 crRNA:tra	AGPAT1-NT	T*G*CTGGCTGGCAGGAGTC	PS modified	NA	NA	acactctttccctacacgacgctcttccgatctCCATTTCCCCAGGATGA	gtgactggagttcagacgtgtgctcttccgatctGAGCTACTGTGGGCTG
1B, S1A	AGPS	Cas9	Hs.Cas9.AGPS.1	GTACCAATGAGTGCAAAGCG	Alt-R Cas9 crRNA:tra	AGPS-NT	G*G*GCCGGCCCCGGGAGGC	PS modified	NA	NA	acactctttccctacacgacgctcttccgatctGTTCTCTCTGGCCATCTG	gtgactggagttcagacgtgtgctcttccgatctGCTACTCACCGCTTCTTTG
1B, S1A	AHCTF1	Cas9	Hs.Cas9.AHCTF1.1	AAGGATAACATCGTTACCAC	Alt-R Cas9 crRNA:tra	AHCTF1-NT	T*T*AAAAAGCAAAACAGTG	PS modified	NA	NA	acactctttccctacacgacgctcttccgatctGCAGAACCCACTAATACAAAGGA	gtgactggagttcagacgtgtgctcttccgatctCACAGACAAGCCCTCAGATATATT
1B, S1A	AHCYL1	Cas9	Hs.Cas9.AHCYL1.1	CGGATCTTCTTAAACACGTT	Alt-R Cas9 crRNA:tra	AHCYL1-NT	T*C*ACGCTCTCTTCCACAAT	PS modified	NA	NA	acactctttccctacacgacgctcttccgatctGTCTTCTGGCTGTTCTATAGATC	gtgactggagttcagacgtgtgctcttccgatctTCCAGAATCTGTTACCTGTGAA
1B, S1A	AHSG	Cas9	Hs.Cas9.AHSG.1	GGCCCCGCTGAACGACACCA	Alt-R Cas9 crRNA:tra	AHSG-NT	G*T*GTGCCAAGACTGCCCCC	PS modified	NA	NA	acactctttccctacacgacgctcttccgatctTGGTTTCCTCTCTCCGAG	gtgactggagttcagacgtgtgctcttccgatctTCCAGCTGAAAATTGGAGC
1B, S1A	AHSP	Cas9	Hs.Cas9.AHSP.1	TGCGGAAATGAGATCCTTAT	Alt-R Cas9 crRNA:tra	AHSP-NT	A*G*AACGCTGAACTCCTTCA	PS modified	NA	NA	acactctttccctacacgacgctcttccgatctCTACCCTTTTCTCTACCCAGG	gtgactggagttcagacgtgtgctcttccgatctAAATGGAAAGCTTGGACTCAC
1B, S1A	AIDA	Cas9	Hs.Cas9.AIDA.1	CCAAGGTTGCCATCGGAACC	Alt-R Cas9 crRNA:tra	AIDA-NT	C*T*CTGTTGCCAGGTACTTT	PS modified	NA	NA	acactctttccctacacgacgctcttccgatctCCAGCATCTTTCAAACCAATTTT	gtgactggagttcagacgtgtgctcttccgatctACTGTGAAATGATATGGAGCTTTT
1B, S1A	AK1	Cas9	Hs.Cas9.AK1.1	TCCTGATTGATGGCTACCCG	Alt-R Cas9 crRNA:tra	AK1-NT	C*A*AAGTCAATACTTCCAAA	PS modified	NA	NA	acactctttccctacacgacgctcttccgatctTTGGGTCAGTGCCTTAC	gtgactggagttcagacgtgtgctcttccgatctGAGACAGTGTTGGACATG
1B, S1A	AK2	Cas9	Hs.Cas9.AK2.1	GAGTGGCCGTTCCTACCACG	Alt-R Cas9 crRNA:tra	AK2-NT	T*C*TTTCAGGCTGATTCACC	PS modified	NA	NA	acactctttccctacacgacgctcttccgatctTCCTGAGTTTACATACGTCATCTTT	gtgactggagttcagacgtgtgctcttccgatctCGTACTGTCAGTTAACCTAACTCAT
1B, S1A	AKAIN1	Cas9	Hs.Cas9.AKAIN1.1	ATTCTGCACAATCTGTTTGC	Alt-R Cas9 crRNA:tra	AKAIN1-NT	T*G*CTGCACAGCTTGCAGG	PS modified	NA	NA	acactctttccctacacgacgctcttccgatctGTCACTGATTCTCTCTTCTCTG	gtgactggagttcagacgtgtgctcttccgatctTAGGTGAGAAACCTGGAAATGA
1B, S1A	AKAP10	Cas9	Hs.Cas9.AKAP10.1	AGAAGCGGGACTATTTCTAC	Alt-R Cas9 crRNA:tra	AKAP10-NT	T*T*TCCTGACAATTCTTTTAG	PS modified	NA	NA	acactctttccctacacgacgctcttccgatctCACATATGTACACACAAGAAAATCACATA	gtgactggagttcagacgtgtgctcttccgatctAATAATAGAACTAACAGCACTCAGAATCA
1B, S1A	AKAP6	Cas9	Hs.Cas9.AKAP6.1	ACGCCAATGGCAACTACACT	Alt-R Cas9 crRNA:tra	AKAP6-NT	G*C*GCATTCTGCAAGGTCTG	PS modified	NA	NA	acactctttccctacacgacgctcttccgatctCTTGAAACAGAGTTCTCCCTAAAG	gtgactggagttcagacgtgtgctcttccgatctGCTTAACTTCAAGGAAAACACTCA
1B, S1A	AKIRIN1	Cas9	Hs.Cas9.AKIRIN1.1	AAGAATATAGTCGTTATCAG	Alt-R Cas9 crRNA:tra	AKIRIN1-NT	G*C*AAATTTTTCAGAACATA	PS modified	NA	NA	acactctttccctacacgacgctcttccgatctAAGATCTTTGTCTCTTCCCACTT	gtgactggagttcagacgtgtgctcttccgatctCACAAGCTTCACTCTGATTAAGAA
1B, S1A	AKR1B10	Cas9	Hs.Cas9.AKR1B10.1	GAACGTTGCTTTTCCACCGA	Alt-R Cas9 crRNA:tra	AKR1B10-NT	G*A*ACCTACCTCCCAGGCAT	PS modified	NA	NA	acactctttccctacacgacgctcttccgatctAGATGTGGTATTTAGCAAGAGTCA	gtgactggagttcagacgtgtgctcttccgatctCATGGATAACTGAAGATTTCTCTCC
1B, S1A	ALDH3A1	Cas9	Hs.Cas9.ALDH3A1.1	GGACACCCCCATTGATTACT	Alt-R Cas9 crRNA:tra	ALDH3A1-NT	C*T*TGAGCAGCTCCGTGGTC	PS modified	NA	NA	acactctttccctacacgacgctcttccgatctTCCAGGTCACAGTTCTTGT	gtgactggagttcagacgtgtgctcttccgatctTGGTCTTTGCTGAGCCTC
1B, S1A	ALDOA	Cas9	Hs.Cas9.ALDOA.1	ACGGAGCTGACTTCGCCAAG	Alt-R Cas9 crRNA:tra	ALDOA-NT	G*C*GCTGTGCCCAGTACAA	PS modified	NA	NA	acactctttccctacacgacgctcttccgatctTCTTCTCTTAGGGTTGGATGG	gtgactggagttcagacgtgtgctcttccgatctAACATTGGCATTTTCCATGATG
1B, S1A	ALG13	Cas9	Hs.Cas9.ALG13.1	TCGTTTATAACCACTACGAG	Alt-R Cas9 crRNA:tra	ALG13-NT	G*C*TGATGATTGTTCATCAA	PS modified	NA	NA	acactctttccctacacgacgctcttccgatctTATTTGTAGGTGCAGGAAGCT	gtgactggagttcagacgtgtgctcttccgatctTGACCCTCTTTGTGTAGCTG
1B, S1A	ALG14	Cas9	Hs.Cas9.ALG14.1	TTCCATGGACGTTACGCCCC	Alt-R Cas9 crRNA:tra	ALG14-NT	C*T*GCGAATATGGGTAGTG	PS modified	NA	NA	acactctttccctacacgacgctcttccgatctTCGCCTCCTCGATACTTAC	gtgactggagttcagacgtgtgctcttccgatctTGTGCGTTCTCGTTCTAG
1B, S1A	ALG1L	Cas9	Hs.Cas9.ALG1L.1	GTGGCACTCACTCTCTGTTC	Alt-R Cas9 crRNA:tra	ALG1L-NT	G*C*ACCGGCTCTTCATGAAG	PS modified	NA	NA	acactctttccctacacgacgctcttccgatctTGGGTTCCCAGGTCTG	gtgactggagttcagacgtgtgctcttccgatctACCTCTGGACCTGCTG
1B, S1A	ALG1L2	Cas9	Hs.Cas9.ALG1L2.1	ACATCCAGGTCTGCATCCCC	Alt-R Cas9 crRNA:tra	ALG1L2-NT	C*A*TCCACCAGAAGCATTTC	PS modified	NA	NA	acactctttccctacacgacgctcttccgatctAGTATTACAGCCGCCTCAT	gtgactggagttcagacgtgtgctcttccgatctTCAGAAAAGGTACACCCCG
1B, S1A	ALKAL1	Cas9	Hs.Cas9.ALKAL1.1	TGTCCCCGCACGGAGCCCAC	Alt-R Cas9 crRNA:tra	ALKAL1-NT	A*C*TCTTCCTGCTGGCGCTG	PS modified	NA	NA	acactctttccctacacgacgctcttccgatctTTGGGCTCCTTATCCGT	gtgactggagttcagacgtgtgctcttccgatctCTTGCGAACATGCGGC
1B, S1A	ALKBH1	Cas9	Hs.Cas9.ALKBH1.1	ACGGAGACCCCGAAGTTTAC	Alt-R Cas9 crRNA:tra	ALKBH1-NT	T*G*CAGGTATAAAGAAGCG	PS modified	NA	NA	acactctttccctacacgacgctcttccgatctCTGTCCCAGTTATAATGGTAGC	gtgactggagttcagacgtgtgctcttccgatctCCTCTTTTGTCACCAATCTTTG
1B, S1A	ALKBH7	Cas9	Hs.Cas9.ALKBH7.1	CCGCTACGAATACGATCACT	Alt-R Cas9 crRNA:tra	ALKBH7-NT	C*T*GGAGCCCGAGCTGCGC	PS modified	NA	NA	acactctttccctacacgacgctcttccgatctAGACGCTGAGCCGAGAA	gtgactggagttcagacgtgtgctcttccgatctCGCCCCATCTGATGCTC
1B, S1A	ALKBH8	Cas9	Hs.Cas9.ALKBH8.1	GCAAATGAGTACGGCTTGTT	Alt-R Cas9 crRNA:tra	ALKBH8-NT	A*T*TCTTCTGTAGTTCTGTAT	PS modified	NA	NA	acactctttccctacacgacgctcttccgatctCCACTACTTCTTTTCCATTGAGG	gtgactggagttcagacgtgtgctcttccgatctTCCCGGTTTTAGAGAAATGTG
1B, S1A	ALOX5	Cas9	Hs.Cas9.ALOX5.1	GGATTCATACGACGTGACTG	Alt-R Cas9 crRNA:tra	ALOX5-NT	A*T*TGCCTGTTCTCCTTCCCA	PS modified	NA	NA	acactctttccctacacgacgctcttccgatctAACACCTCCAGAACAAAGG	gtgactggagttcagacgtgtgctcttccgatctAGGTACCAGTCGTCATTCAG
1B, S1A	ALPK1	Cas9	Hs.Cas9.ALPK1.1	CGTCGAGCCCATACAGGAAC	Alt-R Cas9 crRNA:tra	ALPK1-NT	G*A*CCTGCAGAAGTTTTCCA	PS modified	NA	NA	acactctttccctacacgacgctcttccgatctGATGTTCCACGCTGTTC	gtgactggagttcagacgtgtgctcttccgatctTTGTGGAGACCTTTGGC
1B, S1A	ALPK2	Cas9	Hs.Cas9.ALPK2.1	GATAACCCTAACCTACACCG	Alt-R Cas9 crRNA:tra	ALPK2-NT	A*G*TGACTATGAACTTTGCC	PS modified	NA	NA	acactctttccctacacgacgctcttccgatctCAGGTTCCTTTGCCAAACT	gtgactggagttcagacgtgtgctcttccgatctAGCTTTCCAGTGAAGACTCT
1B, S1A	ALS2	Cas9	Hs.Cas9.ALS2.1	ACAATGGTGTCGCGTACATG	Alt-R Cas9 crRNA:tra	ALS2-NT	C*T*TCCATAGTGGAGCAGTG	PS modified	NA	NA	acactctttccctacacgacgctcttccgatctCGCCAACTGTAAAATCCTGA	gtgactggagttcagacgtgtgctcttccgatctTAGCCCCATTCTAGAAAATGC
1B, S1A	AMDHD1	Cas9	Hs.Cas9.AMDHD1.1	GAGTGGATATGGCCTCGACC	Alt-R Cas9 crRNA:tra	AMDHD1-NT	G*G*CACCACGCTGGTGGAG	PS modified	NA	NA	acactctttccctacacgacgctcttccgatctGCTCCAGTGCATGATGAG	gtgactggagttcagacgtgtgctcttccgatctGCAGTAGGTAGCCGAGAT
1B, S1A	AMELY	Cas9	Hs.Cas9.AMELY.1	GCACCACCAAATCATCCCCG	Alt-R Cas9 crRNA:tra	AMELY-NT	T*A*CGAGCCCATGGGTGGA	PS modified	NA	NA	acactctttccctacacgacgctcttccgatctTGGTGATGAGACTGCAGG	gtgactggagttcagacgtgtgctcttccgatctAGCCAATGGTAAACCTGCAT
1B, S1A	AMIGO2	Cas9	Hs.Cas9.AMIGO2.1	ACGATTACACCTGTCGCCTG	Alt-R Cas9 crRNA:tra	AMIGO2-NT	T*A*GCTCAGTGATGGATTTT	PS modified	NA	NA	acactctttccctacacgacgctcttccgatctAGGAACCATTGATGATGCTGT	gtgactggagttcagacgtgtgctcttccgatctGCTGGTCTTTTGGTATCGTAG
1B, S1A	AMZ2	Cas9	Hs.Cas9.AMZ2.1	ATCTTTACCCAAGAGACTCG	Alt-R Cas9 crRNA:tra	AMZ2-NT	T*G*TTGTGGGAATAACAATG	PS modified	NA	NA	acactctttccctacacgacgctcttccgatctCATGCAGGTGAATTACACGA	gtgactggagttcagacgtgtgctcttccgatctAATACCATCTGTCAAAGAGGC
1B, S1A	ANAPC11	Cas9	Hs.Cas9.ANAPC11.1	GATTAAGTGCTGGAACGGCG	Alt-R Cas9 crRNA:tra	ANAPC11-NT	G*G*CTCTGCTGCCATGAAGG	PS modified	NA	NA	acactctttccctacacgacgctcttccgatctGTTGGTGCCTAAACGTTCTA	gtgactggagttcagacgtgtgctcttccgatctCAGATGCCACAGTTCTCAT
1B, S1A	ANGPTL4	Cas9	Hs.Cas9.ANGPTL4.1	ACATTGTGAGCCGGGTCAAC	Alt-R Cas9 crRNA:tra	ANGPTL4-NT	G*A*CACTCACGGTGCAGGC	PS modified	NA	NA	acactctttccctacacgacgctcttccgatctGTCCCATCCTAGTTTGGC	gtgactggagttcagacgtgtgctcttccgatctTGGACCTGACAAGGAGAG
1B, S1A	ANGPTL6	Cas9	Hs.Cas9.ANGPTL6.1	ACAAGACGGACCGGAACCAC	Alt-R Cas9 crRNA:tra	ANGPTL6-NT	T*A*CTGGTGTCACTGGTGCT	PS modified	NA	NA	acactctttccctacacgacgctcttccgatctTAATGTCGACTTACCCACAGG	gtgactggagttcagacgtgtgctcttccgatctTGCAATTACACCTGACTTTCTCC
1B, S1A	ANKEF1	Cas9	Hs.Cas9.ANKEF1.1	CCCATTCGGTCTTGCACATC	Alt-R Cas9 crRNA:tra	ANKEF1-NT	C*A*GCCCTCATTGTGGGAGT	PS modified	NA	NA	acactctttccctacacgacgctcttccgatctCTTTGCACTTAGCCTCAGTTT	gtgactggagttcagacgtgtgctcttccgatctTCCATTGACAATTCATGGCC
1B, S1A	ANKIB1	Cas9	Hs.Cas9.ANKIB1.1	GGTATTCTCACGGGATCCCG	Alt-R Cas9 crRNA:tra	ANKIB1-NT	G*C*CCTCAATCTGGAATCTC	PS modified	NA	NA	acactctttccctacacgacgctcttccgatctCAACACCACAAAGATTTGGC	gtgactggagttcagacgtgtgctcttccgatctAAACTGACCTCTCGTTTGTCT
1B, S1A	ANKRD13D	Cas9	Hs.Cas9.ANKRD13D.1	CTTCGACACAATGCCAACGT	Alt-R Cas9 crRNA:tra	ANKRD13D-NT	A*C*CTGGAGTCTGTGAGAG	PS modified	NA	NA	acactctttccctacacgacgctcttccgatctCTCTTCTCTCCTGCCCTTT	gtgactggagttcagacgtgtgctcttccgatctTTGTCCTCTGCAGTACCTG
1B, S1A	ANKRD27	Cas9	Hs.Cas9.ANKRD27.1	TAGACTGGATGCTGCTCGAC	Alt-R Cas9 crRNA:tra	ANKRD27-NT	A*A*TGTAGGACTCAAACTGA	PS modified	NA	NA	acactctttccctacacgacgctcttccgatctGGTCTGAAAATGCTCTTCCA	gtgactggagttcagacgtgtgctcttccgatctTTCTGCTGAGGTGGTAAATGG
1B, S1A	ANKRD35	Cas9	Hs.Cas9.ANKRD35.1	CCATTCGAGTCAAGCTTGGT	Alt-R Cas9 crRNA:tra	ANKRD35-NT	G*A*GGACTGACTTACGGGG	PS modified	NA	NA	acactctttccctacacgacgctcttccgatctCTTCAAAAGGGAGCCACAT	gtgactggagttcagacgtgtgctcttccgatctAACCGCCATGATCAGAAG
1B, S1A	ANKRD45	Cas9	Hs.Cas9.ANKRD45.1	CAAAGTTTCCAAACGACCCC	Alt-R Cas9 crRNA:tra	ANKRD45-NT	A*C*ATCCAGTTCTACCAGTG	PS modified	NA	NA	acactctttccctacacgacgctcttccgatctCTGAGAATATCTAGCAGCAACAT	gtgactggagttcagacgtgtgctcttccgatctGTCTGTTTGATCTCACCATCTT
1B, S1A	ANKRD49	Cas9	Hs.Cas9.ANKRD49.1	GGCCACTCACGTGAACACTA	Alt-R Cas9 crRNA:tra	ANKRD49-NT	G*T*GCGGAGACTCCTTTCTG	PS modified	NA	NA	acactctttccctacacgacgctcttccgatctTTCTTCTCAGCTTACCACAGT	gtgactggagttcagacgtgtgctcttccgatctGCTCCTGAACAATATCTAAGTGT
1B, S1A	ANKRD50	Cas9	Hs.Cas9.ANKRD50.1	TCGGAAGGCATATATCGTCA	Alt-R Cas9 crRNA:tra	ANKRD50-NT	C*G*AAAAATAAGTTTAGATG	PS modified	NA	NA	acactctttccctacacgacgctcttccgatctCGCAAAGCTTCTTCTTGATCTAAAC	gtgactggagttcagacgtgtgctcttccgatctGTTGACATTCTCTTTGAAGATATGGT
1B, S1A	ANKRD52	Cas9	Hs.Cas9.ANKRD52.1	CCACGTTGAGGCTGCTCAAC	Alt-R Cas9 crRNA:tra	ANKRD52-NT	A*G*CACTGCGCCCGCTCCTG	PS modified	NA	NA	acactctttccctacacgacgctcttccgatctCAAGATGCCCACTATGCA	gtgactggagttcagacgtgtgctcttccgatctTTCAGCAGATGTGAATGCC
1B, S1A	ANKRD61	Cas9	Hs.Cas9.ANKRD61.1	AGAAGACTGCACTACGATCG	Alt-R Cas9 crRNA:tra	ANKRD61-NT	A*A*ACTCTATGAAGCCATCA	PS modified	NA	NA	acactctttccctacacgacgctcttccgatctCATCTGCAGCACTTCACT	gtgactggagttcagacgtgtgctcttccgatctCAGAATGGTGATGGGCTG
1B, S1A	ANKZF1	Cas9	Hs.Cas9.ANKZF1.1	GCTATACGGTTCGGGCCAAG	Alt-R Cas9 crRNA:tra	ANKZF1-NT	G*G*TGACACACAAAACTTTT	PS modified	NA	NA	acactctttccctacacgacgctcttccgatctTGGTCTCCAGTACTGAGTCT	gtgactggagttcagacgtgtgctcttccgatctCTTCATTGTAGCGCCTCAG
1B, S1A	ANO6	Cas9	Hs.Cas9.ANO6.1	GGACCGGTTTTTCAGATCAT	Alt-R Cas9 crRNA:tra	ANO6-NT	C*A*GTTGAGTGTACCAAAG	PS modified	NA	NA	acactctttccctacacgacgctcttccgatctTGTACGTATGCTGAGATAATGCA	gtgactggagttcagacgtgtgctcttccgatctCTACACTGAGGACTTTGGTAAAC
1B, S1A	ANTXRL	Cas9	Hs.Cas9.ANTXRL.1	GGGGGCCAACGTTTACACCC	Alt-R Cas9 crRNA:tra	ANTXRL-NT	T*A*GGCTCAAAAGGCTCGG	PS modified	NA	NA	acactctttccctacacgacgctcttccgatctCACATGCATTTCAGGACACT	gtgactggagttcagacgtgtgctcttccgatctGGTTGGCTCGAAAGTGAC
1B, S1A	ANXA2R	Cas9	Hs.Cas9.ANXA2R.1	AGTGAGTACTCTCCTAGTAC	Alt-R Cas9 crRNA:tra	ANXA2R-NT	G*C*AGTCCCAAATCACAGCT	PS modified	NA	NA	acactctttccctacacgacgctcttccgatctAGAGTCCGTTTTGCCAGTA	gtgactggagttcagacgtgtgctcttccgatctAGCCTCCACCTATTGTGA
1B, S1A	AP1S3	Cas9	Hs.Cas9.AP1S3.1	AGAGATTGTGCATCGTTACG	Alt-R Cas9 crRNA:tra	AP1S3-NT	C*A*GGACAATGAGCTCTTGA	PS modified	NA	NA	acactctttccctacacgacgctcttccgatctGGGACTGTAGCTAATCCTAAC	gtgactggagttcagacgtgtgctcttccgatctTTCGTGGGAAAAACTGTCTC
1B, S1A	AP3B1	Cas9	Hs.Cas9.AP3B1.1	AAGCGCTTGCACGAATTAGT	Alt-R Cas9 crRNA:tra	AP3B1-NT	T*C*TAATACTTGACAGAACT	PS modified	NA	NA	acactctttccctacacgacgctcttccgatctAGCATCATGATAGGTACAATAATTGG	gtgactggagttcagacgtgtgctcttccgatctTGCTATTGTCATATTACACCCTTTAAG
1B, S1A	AP3M2	Cas9	Hs.Cas9.AP3M2.1	ACAACCGTTCGAAGGATGGT	Alt-R Cas9 crRNA:tra	AP3M2-NT	C*T*GCCGTACCTGTGATGGT	PS modified	NA	NA	acactctttccctacacgacgctcttccgatctTTTCCATTGGCTACCGAGT	gtgactggagttcagacgtgtgctcttccgatctCTGTAGGATGGCCACTATCT
1B, S1A	AP3S2	Cas9	Hs.Cas9.AP3S2.1	TTTCCATCTAGTCCTCAAGC	Alt-R Cas9 crRNA:tra	AP3S2-NT	C*A*ACAGCAGATTGTTCGAG	PS modified	NA	NA	acactctttccctacacgacgctcttccgatctCTGACAGTTTACCTTCCACC	gtgactggagttcagacgtgtgctcttccgatctGGATGTAGTTTTTCAGGCTTG
1B, S1A	APAF1	Cas9	Hs.Cas9.APAF1.1	TCTTTGCACACGGTTGGATC	Alt-R Cas9 crRNA:tra	APAF1-NT	G*G*GCTTCTGATGAAACTGC	PS modified	NA	NA	acactctttccctacacgacgctcttccgatctGGCCTTAAGTTCATTATTCTTTCC	gtgactggagttcagacgtgtgctcttccgatctTAGCCTCTTCAATATTAAGTGGAA
1B, S1A	APBB2	Cas9	Hs.Cas9.APBB2.1	TTGGGACAACGTTGTCCAGC	Alt-R Cas9 crRNA:tra	APBB2-NT	G*G*GGAGCTGGGTGAGGCA	PS modified	NA	NA	acactctttccctacacgacgctcttccgatctAGGCTCACGTTCCTCTCT	gtgactggagttcagacgtgtgctcttccgatctGGCCAAAACCGACTGTG
1B, S1A	APOBEC2	Cas9	Hs.Cas9.APOBEC2.1	ACCAGGTGACATTGTACCGC	Alt-R Cas9 crRNA:tra	APOBEC2-NT	T*G*CACAGGGGCTGGAGGA	PS modified	NA	NA	acactctttccctacacgacgctcttccgatctTTTCTTCAACACCATCCTGC	gtgactggagttcagacgtgtgctcttccgatctCAGGTTCTTGGTCTTGCTAAG
1B, S1A	APOBEC3A	Cas9	Hs.Cas9.APOBEC3A.1	CGGTCAAGATGGACCAGCAC	Alt-R Cas9 crRNA:tra	APOBEC3A-NT	G*G*AGCGCCTGGACAATGG	PS modified	NA	NA	acactctttccctacacgacgctcttccgatctGGCATAAGACCTACCTGTG	gtgactggagttcagacgtgtgctcttccgatctTGACAAACACTGCAGGAAA
1B, S1A	APOC1	Cas9	Hs.Cas9.APOC1.1	TCCAAGGCACTGGAGACGTC	Alt-R Cas9 crRNA:tra	APOC1-NT	T*G*TTTCCAAACTCCTTCAG	PS modified	NA	NA	acactctttccctacacgacgctcttccgatctATTTTGAACCCCTGCCCAT	gtgactggagttcagacgtgtgctcttccgatctAGCCTTGTCCTCCAGTGT
1B, S1A	APOC3	Cas9	Hs.Cas9.APOC3.1	GCAACAACAAGGAGTACCCG	Alt-R Cas9 crRNA:tra	APOC3-NT	C*A*GAGGCCAGGAGCGCCA	PS modified	NA	NA	acactctttccctacacgacgctcttccgatctACAGGACACTTCCTTGCA	gtgactggagttcagacgtgtgctcttccgatctCCACCAAGTGCTTACGG
1B, S1A	APOC4	Cas9	Hs.Cas9.APOC4.1	GAACCATTGCCACCCGTCTC	Alt-R Cas9 crRNA:tra	APOC4-NT	C*C*CAGCCCAGCACACCCTC	PS modified	NA	NA	acactctttccctacacgacgctcttccgatctTAAAGATGAGTCGCTGGAG	gtgactggagttcagacgtgtgctcttccgatctCTTTCCTCCTCCCTGAGA
1B, S1A	APOL2	Cas9	Hs.Cas9.APOL2.1	ACGAGCCCAAGCCCGCAACT	Alt-R Cas9 crRNA:tra	APOL2-NT	G*A*ACTAGTAAACAAATTGC	PS modified	NA	NA	acactctttccctacacgacgctcttccgatctACTCCTTCATCACCTTTGCTA	gtgactggagttcagacgtgtgctcttccgatctGATTACCTGCAGTGTGGTAGAA
1B, S1A	APOPT1	Cas9	Hs.Cas9.APOPT1.1	TGTAAAAGTGAACAGGTCGA	Alt-R Cas9 crRNA:tra	APOPT1-NT	C*A*ATGGAGATTCATTTTCA	PS modified	NA	NA	acactctttccctacacgacgctcttccgatctAAAGGTCTCAAGATTCTGCC	gtgactggagttcagacgtgtgctcttccgatctGTTTGCCCAGAACTGTTGATT
1B, S1A	APP	Cas9	Hs.Cas9.APP.1	GCGGAATTGACAAGTTCCGA	Alt-R Cas9 crRNA:tra	APP-NT	T*G*ACTACGGCATGTTGCTG	PS modified	NA	NA	acactctttccctacacgacgctcttccgatctCACATTGTCACTTTCTTCAGC	gtgactggagttcagacgtgtgctcttccgatctCCTTACACTTTGTAGACATGCA
1B, S1A	AR	Cas9	Hs.Cas9.AR.1	CTGGGACGCAACCTCTCTCG	Alt-R Cas9 crRNA:tra	AR-NT	G*G*CGGCCACGGCGGCTCC	PS modified	NA	NA	acactctttccctacacgacgctcttccgatctAACAGCAACCTTCACAGC	gtgactggagttcagacgtgtgctcttccgatctAGCTGAGTCATCCTCGTC
1B, S1A	AREL1	Cas9	Hs.Cas9.AREL1.1	GGACTATTTATGACTACGTG	Alt-R Cas9 crRNA:tra	AREL1-NT	G*G*ACCGCGAGCGCCGAGG	PS modified	NA	NA	acactctttccctacacgacgctcttccgatctTCCAATCCCAGGAGACTTTG	gtgactggagttcagacgtgtgctcttccgatctACGTGTAGTCAGCTTCCTC
1B, S1A	ARF3	Cas9	Hs.Cas9.ARF3.1	AGGACAAGATTCGACCCCTC	Alt-R Cas9 crRNA:tra	ARF3-NT	T*A*CAGTGTGGGATGTGGG	PS modified	NA	NA	acactctttccctacacgacgctcttccgatctACTCTCTGCTCATACCCAA	gtgactggagttcagacgtgtgctcttccgatctCAAAGGGTTCAATGTGGAGA
1B, S1A	ARF5	Cas9	Hs.Cas9.ARF5.1	CCCCCAACTTCAGTTTGTAC	Alt-R Cas9 crRNA:tra	ARF5-NT	G*G*TTGGGATGGTGGTGAC	PS modified	NA	NA	acactctttccctacacgacgctcttccgatctATCCCTGTGCCCCTTTC	gtgactggagttcagacgtgtgctcttccgatctCGGGCTCACCTATGGTT
1B, S1A	ARHGAP22	Cas9	Hs.Cas9.ARHGAP22.1	GTCCACCACGAGCGGAAGTA	Alt-R Cas9 crRNA:tra	ARHGAP22-NT	T*T*GGGCAGCGCCTAGAGG	PS modified	NA	NA	acactctttccctacacgacgctcttccgatctATGAAGTCCACACACTGCTC	gtgactggagttcagacgtgtgctcttccgatctAGGGTGTGACTAGCTCCT
1B, S1A	ARHGAP23	Cas9	Hs.Cas9.ARHGAP23.1	CATCGTGTACCCACCCGAGT	Alt-R Cas9 crRNA:tra	ARHGAP23-NT	T*T*TGGCTTCACTCTGCGCC	PS modified	NA	NA	acactctttccctacacgacgctcttccgatctTGCTGCTGTACAAAAGTCC	gtgactggagttcagacgtgtgctcttccgatctCCCTCATGAAAGCTTCCC
1B, S1A	ARHGAP24	Cas9	Hs.Cas9.ARHGAP24.1	TCAAGACTTGGCATACTCGC	Alt-R Cas9 crRNA:tra	ARHGAP24-NT	G*C*TGAGGAAGCAAGGAGG	PS modified	NA	NA	acactctttccctacacgacgctcttccgatctGTTTGCTAACTAGGAAAGTCCAT	gtgactggagttcagacgtgtgctcttccgatctGGCCCTTAATGCTTTACATTTTCT
1B, S1A	ARHGAP36	Cas9	Hs.Cas9.ARHGAP36.1	AAGGTGTTCGGACGGTCAAT	Alt-R Cas9 crRNA:tra	ARHGAP36-NT	T*C*AAAATCAGAAACCACTT	PS modified	NA	NA	acactctttccctacacgacgctcttccgatctCAGATCAGGGCATTGGGAT	gtgactggagttcagacgtgtgctcttccgatctCCTCTGCTGTCCTCTCAA
1B, S1A	ARHGAP6	Cas9	Hs.Cas9.ARHGAP6.1	TCGGATTTCCACACGACGTA	Alt-R Cas9 crRNA:tra	ARHGAP6-NT	G*A*CGCGCAGCGCTGGTCC	PS modified	NA	NA	acactctttccctacacgacgctcttccgatctCTTAGTGGGTGCCTTGCT	gtgactggagttcagacgtgtgctcttccgatctAAGTTCCAGTCCCCACC
1B, S1A	ARHGEF33	Cas9	Hs.Cas9.ARHGEF33.1	GATGGCTTGGATCATCGACA	Alt-R Cas9 crRNA:tra	ARHGEF33-NT	T*G*CATTTCTTCTTGTTTGGA	PS modified	NA	NA	acactctttccctacacgacgctcttccgatctCCCCATGTACCACGTTAAAA	gtgactggagttcagacgtgtgctcttccgatctCTGTTGAAGCTGCTCGATTTT
1B, S1A	ARID1B	Cas9	Hs.Cas9.ARID1B.1	TGCCAATTGGATACCGCTGT	Alt-R Cas9 crRNA:tra	ARID1B-NT	G*G*CCCCGGGAGTCCGACC	PS modified	NA	NA	acactctttccctacacgacgctcttccgatctGCAGTAACCCTCATTCTCA	gtgactggagttcagacgtgtgctcttccgatctGAGGGTACTGCATTCCG
1B, S1A	ARIH2	Cas9	Hs.Cas9.ARIH2.1	GTTCTCGTCAAGGACGGCGT	Alt-R Cas9 crRNA:tra	ARIH2-NT	G*C*TGCTGGGAGCAGCACT	PS modified	NA	NA	acactctttccctacacgacgctcttccgatctAAGGAAAACCTACTCTCTCTGG	gtgactggagttcagacgtgtgctcttccgatctGAGTCAAAGATAAACACTTCATGC
1B, S1A	ARL1	Cas9	Hs.Cas9.ARL1.1	CATGGCAACTAACTCTGATT	Alt-R Cas9 crRNA:tra	ARL1-NT	T*G*GTTACGTTTTCTTACCTC	PS modified	NA	NA	acactctttccctacacgacgctcttccgatctAATGACTGCCCCACATTTTA	gtgactggagttcagacgtgtgctcttccgatctCTCTAGGCCATACTGGAGAT
1B, S1A	ARL15	Cas9	Hs.Cas9.ARL15.1	TGGTCGACACGACGTTATCG	Alt-R Cas9 crRNA:tra	ARL15-NT	A*A*CCCCAGCACAGTACCCA	PS modified	NA	NA	acactctttccctacacgacgctcttccgatctTGCACAGGAAACTAGGACAT	gtgactggagttcagacgtgtgctcttccgatctGTTTGCATAGGCCTCACAG
1B, S1A	ARL16	Cas9	Hs.Cas9.ARL16.1	TGCCACGATGTCAGTAAGAT	Alt-R Cas9 crRNA:tra	ARL16-NT	T*C*CCGGATGGTGATCTTTC	PS modified	NA	NA	acactctttccctacacgacgctcttccgatctGCCCATATAGGATTACAACCC	gtgactggagttcagacgtgtgctcttccgatctCTCTTCAGGAGTTCACAACG
1B, S1A	ARL2	Cas9	Hs.Cas9.ARL2.1	AACTACTTTGAGAGCACCGA	Alt-R Cas9 crRNA:tra	ARL2-NT	A*G*TCCCTGCGGTCCTACTG	PS modified	NA	NA	acactctttccctacacgacgctcttccgatctCTCCTTGCCCAGATTCAA	gtgactggagttcagacgtgtgctcttccgatctCATCTGCTGAGAAGGCAG
1B, S1A	ARL6	Cas9	Hs.Cas9.ARL6.1	TGCCACTATTATCTAGCCCA	Alt-R Cas9 crRNA:tra	ARL6-NT	A*A*GTTTGTTAATGATCGTC	PS modified	NA	NA	acactctttccctacacgacgctcttccgatctCCAATATTTTCCATAACTTAAGGTGC	gtgactggagttcagacgtgtgctcttccgatctGCATCTAACAAAGATACTTACATTTGAA
1B, S1A	ARL6IP5	Cas9	Hs.Cas9.ARL6IP5.1	CTACCCCACGACGTTCGTTA	Alt-R Cas9 crRNA:tra	ARL6IP5-NT	G*T*CCTTCGCCGGATGAAGA	PS modified	NA	NA	acactctttccctacacgacgctcttccgatctGGCAGCCCACAATAAAGAC	gtgactggagttcagacgtgtgctcttccgatctCACAAAGACCATGACTCCTC
1B, S1A	ARMC10	Cas9	Hs.Cas9.ARMC10.1	TTACCTGCTGGAGTCAACGG	Alt-R Cas9 crRNA:tra	ARMC10-NT	G*C*TGAACAACTTCAGAAAC	PS modified	NA	NA	acactctttccctacacgacgctcttccgatctTTCTTATGCAGAAGACTTAACTGATG	gtgactggagttcagacgtgtgctcttccgatctTGCATTGTTACCCAAAGTAATCAAAG
1B, S1A	ARMC3	Cas9	Hs.Cas9.ARMC3.1	ACTACTGAGTAGCCCTGACC	Alt-R Cas9 crRNA:tra	ARMC3-NT	G*G*GGGATTAGAGCCACTC	PS modified	NA	NA	acactctttccctacacgacgctcttccgatctCAAACATGTCTGCAGAGTACAC	gtgactggagttcagacgtgtgctcttccgatctCTTACCTGCACCAAGTTGTAAAT
1B, S1A	ARMC6	Cas9	Hs.Cas9.ARMC6.1	CACACGGATGTCGTCATCGA	Alt-R Cas9 crRNA:tra	ARMC6-NT	T*G*GTTGTGGGCATGGCCA	PS modified	NA	NA	acactctttccctacacgacgctcttccgatctCGCTTGCCTGAAACATGAA	gtgactggagttcagacgtgtgctcttccgatctCCTTTGTTCTCCTGCACAAT
1B, S1A	ARMCX1	Cas9	Hs.Cas9.ARMCX1.1	TTGAACTTGCCTCTCCGGAC	Alt-R Cas9 crRNA:tra	ARMCX1-NT	T*A*TCATCAATTTTATAAGG	PS modified	NA	NA	acactctttccctacacgacgctcttccgatctGGGCAAGTGGAAAATCCAAG	gtgactggagttcagacgtgtgctcttccgatctTTGTTACCCAGAGTGACCAA
1B, S1A	ARNTL2	Cas9	Hs.Cas9.ARNTL2.1	CTCACGCGGCTGGAAACCAC	Alt-R Cas9 crRNA:tra	ARNTL2-NT	T*A*GCTGTTGGTCTTGTCCC	PS modified	NA	NA	acactctttccctacacgacgctcttccgatctGCCCATAGGTAAAGTGTTGA	gtgactggagttcagacgtgtgctcttccgatctGTGGAAACTCTGTCATGTGT
1B, S1A	ARR3	Cas9	Hs.Cas9.ARR3.1	AGTACAATTTGCACCACCGG	Alt-R Cas9 crRNA:tra	ARR3-NT	T*A*TGTGCGGCTGGTTGTCC	PS modified	NA	NA	acactctttccctacacgacgctcttccgatctCTGGCTAATCTCTTGGTCTCT	gtgactggagttcagacgtgtgctcttccgatctATCAGTGAAGAAAGGGCATCA
1B, S1A	ASAP3	Cas9	Hs.Cas9.ASAP3.1	CTAAGGATTCCACGGCCTCT	Alt-R Cas9 crRNA:tra	ASAP3-NT	G*T*TCTGGGACAGGTGGCTC	PS modified	NA	NA	acactctttccctacacgacgctcttccgatctTGAACACGGCCAAGTTTAG	gtgactggagttcagacgtgtgctcttccgatctAGCTAACTCCAAGCTCCC
1B, S1A	ASB7	Cas9	Hs.Cas9.ASB7.1	TGATCACGGCGTATCGCAAC	Alt-R Cas9 crRNA:tra	ASB7-NT	T*C*GGCAATAAGAGTGATTG	PS modified	NA	NA	acactctttccctacacgacgctcttccgatctTCGCCATTATCCGAGAGAG	gtgactggagttcagacgtgtgctcttccgatctCCTCTCTGAAGGAACATTCG
1B, S1A	ASCC3	Cas9	Hs.Cas9.ASCC3.1	ATGGCTTTACCTCGTCTCAC	Alt-R Cas9 crRNA:tra	ASCC3-NT	A*T*GGATCACTGATTGAATA	PS modified	NA	NA	acactctttccctacacgacgctcttccgatctCAAATCTACCTTTAAGTCAGCCA	gtgactggagttcagacgtgtgctcttccgatctGTTTCAGGTTTCTTGCTGTATG
1B, S1A	ASCL5	Cas9	Hs.Cas9.ASCL5.1	GCGAGTCAAGTGCGTCAACG	Alt-R Cas9 crRNA:tra	ASCL5-NT	A*A*GCGCAACGAGCGCGAG	PS modified	NA	NA	acactctttccctacacgacgctcttccgatctTCTCCACCTTGCTGAGTC	gtgactggagttcagacgtgtgctcttccgatctAATACCCCTTCGAGCCAG
1B, S1A	ASIC5	Cas9	Hs.Cas9.ASIC5.1	AGAACCGGAGCAAAATTCGC	Alt-R Cas9 crRNA:tra	ASIC5-NT	T*C*ATGGGATACACAATATT	PS modified	NA	NA	acactctttccctacacgacgctcttccgatctTAGATCTGCCATGTCACAAGT	gtgactggagttcagacgtgtgctcttccgatctAGCGAAAGAAGTTTGACCATGA
1B, S1A	ASL	Cas9	Hs.Cas9.ASL.1	TGCCTACGACCGGCACCTTT	Alt-R Cas9 crRNA:tra	ASL-NT	A*T*GGAGAAGTTCAACGCG	PS modified	NA	NA	acactctttccctacacgacgctcttccgatctAATTGTTCTTGCTCTCCTGG	gtgactggagttcagacgtgtgctcttccgatctTCTAGGCCATGGAGTATCTG
1B, S1A	ASPHD2	Cas9	Hs.Cas9.ASPHD2.1	CGTGTGGTACTGTTATCACG	Alt-R Cas9 crRNA:tra	ASPHD2-NT	G*T*GGCCTGCCTCCTGGTCC	PS modified	NA	NA	acactctttccctacacgacgctcttccgatctGCTGTCATCACTGTGG	gtgactggagttcagacgtgtgctcttccgatctCGTAGCCATTCTGCAG
1B, S1A	ATF5	Cas9	Hs.Cas9.ATF5.1	TGGGATGGCTCGTAGACTAT	Alt-R Cas9 crRNA:tra	ATF5-NT	G*G*CCCTGCTCCCAGCTAGT	PS modified	NA	NA	acactctttccctacacgacgctcttccgatctTGTGCTACAGCCATGTCA	gtgactggagttcagacgtgtgctcttccgatctAAGGACCTCATAGGGAGC
1B, S1A	ATG4D	Cas9	Hs.Cas9.ATG4D.1	CGGCTTTTAACCACCCAACC	Alt-R Cas9 crRNA:tra	ATG4D-NT	T*G*GAGATCTTGCTAAAGCT	PS modified	NA	NA	acactctttccctacacgacgctcttccgatctGCTGTCATGCAAGTGCTT	gtgactggagttcagacgtgtgctcttccgatctCACAGAGGTGGATGCTG
1B, S1A	ATP10D	Cas9	Hs.Cas9.ATP10D.1	GAGACCCACGCGTTCTTTGT	Alt-R Cas9 crRNA:tra	ATP10D-NT	C*T*AAGCAACAAATTTTCTT	PS modified	NA	NA	acactctttccctacacgacgctcttccgatctCAAGTCAAAAACATTCAAGGGC	gtgactggagttcagacgtgtgctcttccgatctGTGTTTCTAATGGTGCATCCT
1B, S1A	ATP1B4	Cas9	Hs.Cas9.ATP1B4.1	GACGGTGGTGCCCAAATCGG	Alt-R Cas9 crRNA:tra	ATP1B4-NT	G*A*AGCAGAAGAAGAGGCT	PS modified	NA	NA	acactctttccctacacgacgctcttccgatctCACTGTTCCAGGATGATCC	gtgactggagttcagacgtgtgctcttccgatctCTCTGGATCCCACAGGTATT
1B, S1A	ATP2A2	Cas9	Hs.Cas9.ATP2A2.1	TTCGGCGTCAACGAGAGTAC	Alt-R Cas9 crRNA:tra	ATP2A2-NT	C*G*GTGGAGGAGGTGCTGG	PS modified	NA	NA	acactctttccctacacgacgctcttccgatctAAGCCATGGAGAACGCG	gtgactggagttcagacgtgtgctcttccgatctCACCTACCGTTGGAGCC
1B, S1A	ATP5E	Cas9	Hs.Cas9.ATP5E.1	TTGCACAGATCTGGGAGTAT	Alt-R Cas9 crRNA:tra	ATP5E-NT	C*T*TCAGTGCATCTCTCACTG	PS modified	NA	NA	acactctttccctacacgacgctcttccgatctCCAGAAGTCTTCTCAGCATTT	gtgactggagttcagacgtgtgctcttccgatctCACAGTAACAGCTGTCTGG
1B, S1A	ATP5J2	Cas9	Hs.Cas9.ATP5J2.1	GCCAAGCTGGATCTTGATGC	Alt-R Cas9 crRNA:tra	ATP5J2-NT	C*T*GGAGGTCAAACTGGGG	PS modified	NA	NA	acactctttccctacacgacgctcttccgatctCTGACCTCTTTGAAACGCTC	gtgactggagttcagacgtgtgctcttccgatctCCGTTTCAGTACCAGTGAAG
1B, S1A	ATP5L2	Cas9	Hs.Cas9.ATP5L2.1	GTACTACACCACGGTTGAGC	Alt-R Cas9 crRNA:tra	ATP5L2-NT	A*A*GCCTCGATTGGCCGCAT	PS modified	NA	NA	acactctttccctacacgacgctcttccgatctCTACCAGTCTGAGCACTACT	gtgactggagttcagacgtgtgctcttccgatctTGCTGTGACTTACTTGAAGC
1B, S1A	ATP6AP2	Cas9	Hs.Cas9.ATP6AP2.1	GAAACAGGCGATTACGGAGC	Alt-R Cas9 crRNA:tra	ATP6AP2-NT	T*G*AACTGAGAACAGAGTTT	PS modified	NA	NA	acactctttccctacacgacgctcttccgatctTCTTCTTACAGAGAGTGTATATGGTA	gtgactggagttcagacgtgtgctcttccgatctCTTACTTCATTGTTCCTACTCAGAG
1B, S1A	ATP6V0A2	Cas9	Hs.Cas9.ATP6V0A2.1	TGTTTGGATAGGGGTACACG	Alt-R Cas9 crRNA:tra	ATP6V0A2-NT	G*A*TCTCCCTCCGCTCCTCG	PS modified	NA	NA	acactctttccctacacgacgctcttccgatctAGTTCTAGTGCTGACAGATGT	gtgactggagttcagacgtgtgctcttccgatctCACTTCCAGCTTACTCACAGT
1B, S1A	ATP6V1G1	Cas9	Hs.Cas9.ATP6V1G1.1	TTCAGTGCTGCAACTGCCAC	Alt-R Cas9 crRNA:tra	ATP6V1G1-NT	T*T*CTCCTGGGTCTCCTTCTC	PS modified	NA	NA	acactctttccctacacgacgctcttccgatctGTTCTGATAATCCCTCCGTGA	gtgactggagttcagacgtgtgctcttccgatctGGTTGTCCAAGACTTCATCC
1B, S1A	ATP8B3	Cas9	Hs.Cas9.ATP8B3.1	GCCAACAACCGTGCCTACAA	Alt-R Cas9 crRNA:tra	ATP8B3-NT	C*A*GCATTCACCTGGAAGG	PS modified	NA	NA	acactctttccctacacgacgctcttccgatctCCGCCCACCTTGTATTT	gtgactggagttcagacgtgtgctcttccgatctGGTGTTTGTCCTGGGC
1B, S1A	ATXN7	Cas9	Hs.Cas9.ATXN7.1	TAATCAGGTTGTCAAACCGC	Alt-R Cas9 crRNA:tra	ATXN7-NT	T*A*CTTGGTGGTGTGTAACG	PS modified	NA	NA	acactctttccctacacgacgctcttccgatctGTCCAGCCCATGATGATTT	gtgactggagttcagacgtgtgctcttccgatctACATTATGTCCCATGCATGC
1B, S1A	AVEN	Cas9	Hs.Cas9.AVEN.1	GTTCGGCAGCAACGTTGAGT	Alt-R Cas9 crRNA:tra	AVEN-NT	C*T*TCGGGATTTTTACCTGG	PS modified	NA	NA	acactctttccctacacgacgctcttccgatctTTTCTCCTCCTGCCCTAAT	gtgactggagttcagacgtgtgctcttccgatctGTGAGTTATTGGTTCGAGCC
1B, S1A	AXL	Cas9	Hs.Cas9.AXL.1	TGCGAAGCCCATAACGCCAA	Alt-R Cas9 crRNA:tra	AXL-NT	T*G*AACAAGACATCCTCTTT	PS modified	NA	NA	acactctttccctacacgacgctcttccgatctACCCTTCCCTCATATGACT	gtgactggagttcagacgtgtgctcttccgatctCTCCCAACTCTGTCACCT
1B, S1A	B3GNT4	Cas9	Hs.Cas9.B3GNT4.1	ACTCTAACACGTTGGGGACG	Alt-R Cas9 crRNA:tra	B3GNT4-NT	G*G*CTGGGTCCCAGCCATCC	PS modified	NA	NA	acactctttccctacacgacgctcttccgatctAGGCCCATTTCATGCTAAA	gtgactggagttcagacgtgtgctcttccgatctTGGCTCTGGACATGACATAT
1B, S1A	B4GALNT1	Cas9	Hs.Cas9.B4GALNT1.1	CCCACGGCGCAAGAGGTAGC	Alt-R Cas9 crRNA:tra	B4GALNT1-NT	C*C*TGCGGGGGCTTTGCGG	PS modified	NA	NA	acactctttccctacacgacgctcttccgatctAGGAGCAAGATCTGGCAG	gtgactggagttcagacgtgtgctcttccgatctGTGCGCTCTGGTCCTT
1B, S1A	BAHD1	Cas9	Hs.Cas9.BAHD1.1	ACCAATGGGCGCTTACGAAG	Alt-R Cas9 crRNA:tra	BAHD1-NT	T*G*CAGGCCTTGGGCTTCTC	PS modified	NA	NA	acactctttccctacacgacgctcttccgatctGAGAAAGTCCCTTCCCATG	gtgactggagttcagacgtgtgctcttccgatctATTCTCCAGGCGAGTCAG
1B, S1A	BAK1	Cas9	Hs.Cas9.BAK1.1	GGAACTCTGAGTCATAGCGT	Alt-R Cas9 crRNA:tra	BAK1-NT	C*T*GCAGGTGCTGCAACATG	PS modified	NA	NA	acactctttccctacacgacgctcttccgatctGTGGCAATCTTGGTGAAGTA	gtgactggagttcagacgtgtgctcttccgatctTGGTTACTGGCTCACCTG
1B, S1A	BANF1	Cas9	Hs.Cas9.BANF1.1	CCAAAAGCACCGAGACTTCG	Alt-R Cas9 crRNA:tra	BANF1-NT	A*G*CCTGATCAAGATGACA	PS modified	NA	NA	acactctttccctacacgacgctcttccgatctTCTTACTATGAGATTGCTCGTGG	gtgactggagttcagacgtgtgctcttccgatctACACCTTGTCAAAACCCCTTTC
1B, S1A	BATF2	Cas9	Hs.Cas9.BATF2.1	GGTTTGGGGGACACACGGGT	Alt-R Cas9 crRNA:tra	BATF2-NT	C*T*CCTGTGGGCAAGAGAA	PS modified	NA	NA	acactctttccctacacgacgctcttccgatctGCCCTTCATAACCACCTAC	gtgactggagttcagacgtgtgctcttccgatctATGACATGGTTGCTGAATGG
1B, S1A	BBOX1	Cas9	Hs.Cas9.BBOX1.1	ACCGGAGCATCTGACAAACC	Alt-R Cas9 crRNA:tra	BBOX1-NT	A*G*AAAGTAGGCATAGTAA	PS modified	NA	NA	acactctttccctacacgacgctcttccgatctCAGCTACCCACTTTGGATTTT	gtgactggagttcagacgtgtgctcttccgatctGTCCTCAGTTGGGAACTATTT
1B, S1A	BBS2	Cas9	Hs.Cas9.BBS2.1	GCCACCAATAATCGCAAGAG	Alt-R Cas9 crRNA:tra	BBS2-NT	T*T*GAAACCTTGCAGAGCAC	PS modified	NA	NA	acactctttccctacacgacgctcttccgatctATACCGTCCAAAAGAGATCACTT	gtgactggagttcagacgtgtgctcttccgatctCTTTTCTTAGGTAGCAGATGGG
1B, S1A	BCAR3	Cas9	Hs.Cas9.BCAR3.1	GGTGAATCACCAGTTCCCCC	Alt-R Cas9 crRNA:tra	BCAR3-NT	G*C*AAGCCTTCCCAGAAACA	PS modified	NA	NA	acactctttccctacacgacgctcttccgatctTGTATAGACACATCTTGATAGGCAT	gtgactggagttcagacgtgtgctcttccgatctGAATTATGGCTGCAGGAAAATTTG
1B, S1A	BCCIP	Cas9	Hs.Cas9.BCCIP.1	CTGCGGAACCCCACTTTCCA	Alt-R Cas9 crRNA:tra	BCCIP-NT	C*G*CTGGACTGGGGGATCC	PS modified	NA	NA	acactctttccctacacgacgctcttccgatctGCTACTATGGGCTGGTC	gtgactggagttcagacgtgtgctcttccgatctTTTCCTCTTCCTCGTCG
1B, S1A	BCHE	Cas9	Hs.Cas9.BCHE.1	TACCGCCCAAGGAGCATTAA	Alt-R Cas9 crRNA:tra	BCHE-NT	T*T*CCTAGCTTCATAAAGAG	PS modified	NA	NA	acactctttccctacacgacgctcttccgatctAGCTAAGTTCAACGTTCTGTTC	gtgactggagttcagacgtgtgctcttccgatctTAGCCTGCATTTGCTTTCTC

1B, S1A	BCO2	Cas9	Hs.Cas9.BCO2.1	GTCAACTATGTGCGGTACAA	Alt-R Cas9 crRNA:tra	BCO2-NT	C*A*GCCATGACTGACAATAC	PS modified	NA	NA	acactctttccctacacgacgctcttccgatctAAATAACTCTAGGGTTTGGTTTCA	gtgactggagttcagacgtgtgctcttccgatctCTTTATTCATAAAGTTGGTCTCAGT
1B, S1A	BECN2	Cas9	Hs.Cas9.BECN2.1	TCACCGCCACGTTTGAGATC	Alt-R Cas9 crRNA:tra	BECN2-NT	C*C*GGCTGAAGGAAATCAA	PS modified	NA	NA	acactctttccctacacgacgctcttccgatctAGAACCAGCTGCAGTATG	gtgactggagttcagacgtgtgctcttccgatctGGCCCAACCTGAAGTTATT
1B, S1A	BEND7	Cas9	Hs.Cas9.BEND7.1	GAATGGCCTGCAACGTTGAC	Alt-R Cas9 crRNA:tra	BEND7-NT	C*C*TCATGGTCTTGAGTTCT	PS modified	NA	NA	acactctttccctacacgacgctcttccgatctGATCCTTGTACCTGCTTGAATTT	gtgactggagttcagacgtgtgctcttccgatctGTGAATTCATCAGCTGGATCAAA
1B, S1A	BEX1	Cas9	Hs.Cas9.BEX1.1	GTGTGCCTAGAGGAAATCGT	Alt-R Cas9 crRNA:tra	BEX1-NT	C*C*CTTTGGATGCTGGTGAA	PS modified	NA	NA	acactctttccctacacgacgctcttccgatctTCCAAGCCTATGCATCATATC	gtgactggagttcagacgtgtgctcttccgatctGCAGTAAACAGTCTCAGCAT
1B, S1A	BEX5	Cas9	Hs.Cas9.BEX5.1	ATGTTATCGACAAGCCTATT	Alt-R Cas9 crRNA:tra	BEX5-NT	C*T*CCATCTCCATCTATCATA	PS modified	NA	NA	acactctttccctacacgacgctcttccgatctCCTTAGCTCTCTCATCTCCT	gtgactggagttcagacgtgtgctcttccgatctCAAAGTTGTGGAGAAGGCC
1B, S1A	BICDL1	Cas9	Hs.Cas9.BICDL1.1	CGACCCCGGGGAACACCCTC	Alt-R Cas9 crRNA:tra	BICDL1-NT	C*T*GGCGGCCGGGGAGCGG	PS modified	NA	NA	acactctttccctacacgacgctcttccgatctGGAGCTAGAACTGGCGTTA	gtgactggagttcagacgtgtgctcttccgatctGCTCATGTCCTGGTTCCT
1B, S1A	BICDL2	Cas9	Hs.Cas9.BICDL2.1	CAACGAGGCAGCCGACACGT	Alt-R Cas9 crRNA:tra	BICDL2-NT	A*G*GCTGTGGGCCAGTTCTG	PS modified	NA	NA	acactctttccctacacgacgctcttccgatctTGCAACCCTCTCGATGG	gtgactggagttcagacgtgtgctcttccgatctTCCGAGCTGGAGGAGG
1B, S1A	BLOC1S1	Cas9	Hs.Cas9.BLOC1S1.1	GATCCACCAAAGCTTCTGTC	Alt-R Cas9 crRNA:tra	BLOC1S1-NT	A*G*GTCCATACCCCACATTG	PS modified	NA	NA	acactctttccctacacgacgctcttccgatctCAACTAGCAGAATAGTAATGGATGG	gtgactggagttcagacgtgtgctcttccgatctGAGGAAACTGATGTTGATAAGAGGT
1B, S1A	BLOC1S6	Cas9	Hs.Cas9.BLOC1S6.1	GTGTGTCTAACAATACAACT	Alt-R Cas9 crRNA:tra	BLOC1S6-NT	A*A*ATTTTGAAATCTCTTGTT	PS modified	NA	NA	acactctttccctacacgacgctcttccgatctCACTTTAAATATGTAGAGTTTGTCTTGG	gtgactggagttcagacgtgtgctcttccgatctCCAAAGCATTAATATCCAACATAGAATGA
1B, S1A	BLVRA	Cas9	Hs.Cas9.BLVRA.1	ACACGAAGCCAATCAGGTTC	Alt-R Cas9 crRNA:tra	BLVRA-NT	C*A*AAGACATTGTGAGCCAC	PS modified	NA	NA	acactctttccctacacgacgctcttccgatctCCTACAGTGTTTTCAGACTCCA	gtgactggagttcagacgtgtgctcttccgatctCTGGGCTTTCCATGAATTATGAA
1B, S1A	BMF	Cas9	Hs.Cas9.BMF.1	AGAGAGCAAGCTCCCGGGTT	Alt-R Cas9 crRNA:tra	BMF-NT	A*G*GCTCTGGGCAAACAGG	PS modified	NA	NA	acactctttccctacacgacgctcttccgatctAGAGTCTGGGTAGCTTTGT	gtgactggagttcagacgtgtgctcttccgatctAGGATGATGTGTTCCAACC
1B, S1A	BMP6	Cas9	Hs.Cas9.BMP6.1	CTTCCTCAACGACGCGGACA	Alt-R Cas9 crRNA:tra	BMP6-NT	C*T*GACCAGCGCGCAGGACA	PS modified	NA	NA	acactctttccctacacgacgctcttccgatctTCAACCGCAAGAGCCTT	gtgactggagttcagacgtgtgctcttccgatctCGTTACCCCAAATCCTTACC
1B, S1A	BMT2	Cas9	Hs.Cas9.BMT2.1	TGGTGAAGAGCGTCCACCGG	Alt-R Cas9 crRNA:tra	BMT2-NT	G*G*AGCAGGAGAAGCTCTC	PS modified	NA	NA	acactctttccctacacgacgctcttccgatctTTCCTCCCTCACTCAGC	gtgactggagttcagacgtgtgctcttccgatctGCCGAAATACTGCTCGT
1B, S1A	BMX	Cas9	Hs.Cas9.BMX.1	CGTCCACGAAGAACCCACTA	Alt-R Cas9 crRNA:tra	BMX-NT	C*T*GCTGGCAACACAGGAAC	PS modified	NA	NA	acactctttccctacacgacgctcttccgatctCTTGTTTTCTTCCTGTCTGCT	gtgactggagttcagacgtgtgctcttccgatctCACATTACATGCTTCCCAGAG
1B, S1A	BNIPL	Cas9	Hs.Cas9.BNIPL.1	GGTGTTCCGAATGGGACCAC	Alt-R Cas9 crRNA:tra	BNIPL-NT	G*G*AGAAGATGGACATCAC	PS modified	NA	NA	acactctttccctacacgacgctcttccgatctGTTGTATGTGGGATGTGACT	gtgactggagttcagacgtgtgctcttccgatctATGCTAGCCATTACCTCCAT
1B, S1A	BOLL	Cas9	Hs.Cas9.BOLL.1	GGTGGGACCGAAGTTACCTC	Alt-R Cas9 crRNA:tra	BOLL-NT	T*G*AAATTACTTACAGGCCA	PS modified	NA	NA	acactctttccctacacgacgctcttccgatctGGTTGATGTGTGTTATTATTTGTAATTAT	gtgactggagttcagacgtgtgctcttccgatctCTAACAACTTCAACTGGATATCCTTATA
1B, S1A	BOP1	Cas9	Hs.Cas9.BOP1.1	GTACGATGACTTCCCCCACG	Alt-R Cas9 crRNA:tra	BOP1-NT	G*T*GGGCAACGTGCCCTTGC	PS modified	NA	NA	acactctttccctacacgacgctcttccgatctAACTGGTCCAGCTCATCC	gtgactggagttcagacgtgtgctcttccgatctTAGGATCAGATGCCGACAT
1B, S1A	BRAF	Cas9	Hs.Cas9.BRAF.1	GAACTTCTGTACTACAACGC	Alt-R Cas9 crRNA:tra	BRAF-NT	G*T*CATAATTAACACACATCA	PS modified	NA	NA	acactctttccctacacgacgctcttccgatctCCTCACAGACTTTTAGACATCGTAG	gtgactggagttcagacgtgtgctcttccgatctTCACCTTAGCATTTTGTGACTTTT
1B, S1A	BRD8	Cas9	Hs.Cas9.BRD8.1	CCGGAGGTTACCCACTGTGA	Alt-R Cas9 crRNA:tra	BRD8-NT	C*G*TCAAGCAGTAAAAACAC	PS modified	NA	NA	acactctttccctacacgacgctcttccgatctCTCTTCCATAGTGGTTGGAGT	gtgactggagttcagacgtgtgctcttccgatctCTGCTTTAGTAATGCAACATACCT
1B, S1A	BRDT	Cas9	Hs.Cas9.BRDT.1	TTCTCCCTTGAACGTGGTAC	Alt-R Cas9 crRNA:tra	BRDT-NT	T*C*TGTATTTCCTAAGACATC	PS modified	NA	NA	acactctttccctacacgacgctcttccgatctCGCAACAGAAAAAGTATTTAAGCAG	gtgactggagttcagacgtgtgctcttccgatctTTGCTAGACGCTGAAGACTAATTTT
1B, S1A	BRIX1	Cas9	Hs.Cas9.BRIX1.1	GCAACCAAGAGGAAACGGCG	Alt-R Cas9 crRNA:tra	BRIX1-NT	G*C*GGGCGGCGAGGCAAGA	PS modified	NA	NA	acactctttccctacacgacgctcttccgatctGAGCCGCCGAACCATA	gtgactggagttcagacgtgtgctcttccgatctCGGCTCCGCATCTATTTC
1B, S1A	BSPRY	Cas9	Hs.Cas9.BSPRY.1	CAGGACGTCCGCCACATACT	Alt-R Cas9 crRNA:tra	BSPRY-NT	A*C*TGCTCTTTGATTCTTCCC	PS modified	NA	NA	acactctttccctacacgacgctcttccgatctGAACAAGATTGTGGACCAGT	gtgactggagttcagacgtgtgctcttccgatctTCCCTAAGTCTAAGGCCTTAT
1B, S1A	BTBD3	Cas9	Hs.Cas9.BTBD3.1	TCAACGCCAGATCTTGTCGT	Alt-R Cas9 crRNA:tra	BTBD3-NT	A*A*CCTTGCGTTTATTTTCAA	PS modified	NA	NA	acactctttccctacacgacgctcttccgatctGAAACTCTGAATGCCAAAGAAATT	gtgactggagttcagacgtgtgctcttccgatctCAAGTAAAGTGCCTTTCCTAGAA
1B, S1A	BTF3L4	Cas9	Hs.Cas9.BTF3L4.1	AAGCTTTTTGTCATCAGCTG	Alt-R Cas9 crRNA:tra	BTF3L4-NT	G*C*CAGTTTTTTTAGAGAAC	PS modified	NA	NA	acactctttccctacacgacgctcttccgatctTGTTTGGTTATTTTTCAGGGTACA	gtgactggagttcagacgtgtgctcttccgatctGCAAAGTGATCATACCTCTTCAATA
1B, S1A	BTN3A1	Cas9	Hs.Cas9.BTN3A1.1	GAGGGAACTTCTGATGGTAC	Alt-R Cas9 crRNA:tra	BTN3A1-NT	C*T*GGCTGTCTTTTCCAGGC	PS modified	NA	NA	acactctttccctacacgacgctcttccgatctAGTAGCAGCATCTGTGATCAT	gtgactggagttcagacgtgtgctcttccgatctAGGTTGACTGCAGACACTAA
1B, S1A	BTN3A2	Cas9	Hs.Cas9.BTN3A2.1	TAATCTTCACGTCGAAGTGA	Alt-R Cas9 crRNA:tra	BTN3A2-NT	T*A*TCCTTCCACAGCACTGG	PS modified	NA	NA	acactctttccctacacgacgctcttccgatctCAGGAGCTATCCAGAATTTAGGC	gtgactggagttcagacgtgtgctcttccgatctAGATGCTGCTACTTCATATAGGC
1B, S1A	BUD23	Cas9	Hs.Cas9.BUD23.1	GTAGTCTACCACCATGCCAC	Alt-R Cas9 crRNA:tra	BUD23-NT	T*T*CTTTGCTTTGGCACTGTT	PS modified	NA	NA	acactctttccctacacgacgctcttccgatctGCTGCCTTTCTTTCCTCA	gtgactggagttcagacgtgtgctcttccgatctGCTATGCCACTACCCTCC
1B, S1A	BZW1	Cas9	Hs.Cas9.BZW1.1	AACTGAAACCGGTACTGATT	Alt-R Cas9 crRNA:tra	BZW1-NT	C*A*AGACTGTATTATTCAAG	PS modified	NA	NA	acactctttccctacacgacgctcttccgatctAGGTTTGACCCTACTCAGTTT	gtgactggagttcagacgtgtgctcttccgatctCGACGGTAATCAAGTTTTGCT
1B, S1A	C10orf111	Cas9	Hs.Cas9.C10orf111.1	TCGGAGTCGCTGCAAAGTCG	Alt-R Cas9 crRNA:tra	C10orf111-NT	A*G*CAATGGAGACTCAGGC	PS modified	NA	NA	acactctttccctacacgacgctcttccgatctGTCTTCTAAGTTCTGGCCAA	gtgactggagttcagacgtgtgctcttccgatctGAATATCCCGTCCAGTGTC
1B, S1A	C10orf67	Cas9	Hs.Cas9.C10orf67.1	GGGGGACCTTTGGCACACGC	Alt-R Cas9 crRNA:tra	C10orf67-NT	T*C*ACTGCTTTTCCTCCTCCT	PS modified	NA	NA	acactctttccctacacgacgctcttccgatctTGAATTCCCGAGCTTCTCG	gtgactggagttcagacgtgtgctcttccgatctGATCGCAGGGCTCATTATGT
1B, S1A	C10orf90	Cas9	Hs.Cas9.C10orf90.1	ACCATCACGGCCAGACGCGT	Alt-R Cas9 crRNA:tra	C10orf90-NT	C*C*CGCAGAGGGTTTGCATC	PS modified	NA	NA	acactctttccctacacgacgctcttccgatctAACAGTGTGTCCTCAGC	gtgactggagttcagacgtgtgctcttccgatctGATTAGGAATCCCGGCAC
1B, S1A	C11orf57	Cas9	Hs.Cas9.C11orf57.1	ATGCTTACCGGGGGACCAAA	Alt-R Cas9 crRNA:tra	C11orf57-NT	A*G*AACTGGAAAAACAGAT	PS modified	NA	NA	acactctttccctacacgacgctcttccgatctAGGAATCAGATATGTGGAAAATAAGAG	gtgactggagttcagacgtgtgctcttccgatctGAATTATTTTAGTTCTCAGAGCTGCAA
1B, S1A	C11orf74	Cas9	Hs.Cas9.C11orf74.1	CTGGGCCACAAAAGGGATAC	Alt-R Cas9 crRNA:tra	C11orf74-NT	G*G*CTTCACTTCACAGGTAG	PS modified	NA	NA	acactctttccctacacgacgctcttccgatctTTCCAGGAGAAGTGGAGCA	gtgactggagttcagacgtgtgctcttccgatctCTGTGGAGTACCTCTTCCGT
1B, S1A	C12orf54	Cas9	Hs.Cas9.C12orf54.1	GTCCTGGGTGCTGATGTCAT	Alt-R Cas9 crRNA:tra	C12orf54-NT	G*T*CAGAATAGGGACACTTA	PS modified	NA	NA	acactctttccctacacgacgctcttccgatctCAGAATGACTCTTCTCTGTGT	gtgactggagttcagacgtgtgctcttccgatctCCATCTCCCTTGAACATTGT
1B, S1A	C12orf77	Cas9	Hs.Cas9.C12orf77.1	AGACGCTGGACAGCATACGA	Alt-R Cas9 crRNA:tra	C12orf77-NT	T*G*GGAGCAAACAAGGGAA	PS modified	NA	NA	acactctttccctacacgacgctcttccgatctTGTTTCAGTAGAGATGGCATATTT	gtgactggagttcagacgtgtgctcttccgatctCCTGACAGTTCTAATAAGGTACC
1B, S1A	C14orf132	Cas9	Hs.Cas9.C14orf132.1	TCGGTGCTGAAGTCCTCGTT	Alt-R Cas9 crRNA:tra	C14orf132-NT	C*A*GAGGAGTTAAACAGGG	PS modified	NA	NA	acactctttccctacacgacgctcttccgatctCATGGCCCTGGATAATTCT	gtgactggagttcagacgtgtgctcttccgatctAATCCATAGCAAGACGGC
1B, S1A	C15orf38-AP3S2	Cas9	Hs.Cas9.C15orf38-AP3S2.1	GGGGTACGGCTGAAGACTCG	Alt-R Cas9 crRNA:tra	C15orf38-AP3S2-NT	T*G*ATGGAACTCGAGCTGG	PS modified	NA	NA	acactctttccctacacgacgctcttccgatctGGTTGCATTGCTCACC	gtgactggagttcagacgtgtgctcttccgatctACACAGTGGCGTTCTG
1B, S1A	C15orf53	Cas9	Hs.Cas9.C15orf53.1	CTGGTTTCATGGTTCCTCCG	Alt-R Cas9 crRNA:tra	C15orf53-NT	T*G*CCCTTAGCCTTGCTTCCT	PS modified	NA	NA	acactctttccctacacgacgctcttccgatctGGGACTTCAGTTAGTGACA	gtgactggagttcagacgtgtgctcttccgatctGAAACTGAGAACCCTGCTA
1B, S1A	C16orf58	Cas9	Hs.Cas9.C16orf58.1	ACGGAATCCCACAGCTGGTA	Alt-R Cas9 crRNA:tra	C16orf58-NT	C*C*CAGACGACCGAGCTGA	PS modified	NA	NA	acactctttccctacacgacgctcttccgatctGCACTGTCCTCTGCCC	gtgactggagttcagacgtgtgctcttccgatctCGTGTTCCTGCCTCAGG
1B, S1A	C16orf82	Cas9	Hs.Cas9.C16orf82.1	ATCCTCTGTCCAGAATGAGC	Alt-R Cas9 crRNA:tra	C16orf82-NT	T*T*TCTCGAGGGAGAAAAC	PS modified	NA	NA	acactctttccctacacgacgctcttccgatctTCACAGCCAACATTCAGAG	gtgactggagttcagacgtgtgctcttccgatctGCTCTAAGCTGGGTGACT
1B, S1A	C16orf90	Cas9	Hs.Cas9.C16orf90.1	CGCACCCCCCAACATCTACG	Alt-R Cas9 crRNA:tra	C16orf90-NT	C*A*AGGACGCCCCGGCCACC	PS modified	NA	NA	acactctttccctacacgacgctcttccgatctAAGTTCTTGGGCTTGCTT	gtgactggagttcagacgtgtgctcttccgatctCTGTCTCCTCTGCAGATG
1B, S1A	C17orf102	Cas9	Hs.Cas9.C17orf102.1	AAGGATGCACGCTCCCCACC	Alt-R Cas9 crRNA:tra	C17orf102-NT	G*C*AGTCACTGATGTCCCTT	PS modified	NA	NA	acactctttccctacacgacgctcttccgatctTGATTCCCAGCCAGTG	gtgactggagttcagacgtgtgctcttccgatctCGTTTCTCCAGGGTAGA
1B, S1A	C17orf58	Cas9	Hs.Cas9.C17orf58.1	CCGAGTCCACATGTTAGCCC	Alt-R Cas9 crRNA:tra	C17orf58-NT	C*T*CACCCCCGACGGCTTCT	PS modified	NA	NA	acactctttccctacacgacgctcttccgatctCAGGTTTAAACTCTGGACACG	gtgactggagttcagacgtgtgctcttccgatctGGGTAATGCTCTTCTCCAAA
1B, S1A	C17orf99	Cas9	Hs.Cas9.C17orf99.1	GCGGGCCAACTTCACTCTGC	Alt-R Cas9 crRNA:tra	C17orf99-NT	C*T*GCCAGAGCCAGTGTCTG	PS modified	NA	NA	acactctttccctacacgacgctcttccgatctCTACCTTCTCCCACCCTG	gtgactggagttcagacgtgtgctcttccgatctCTTCCCGATCAGGCTGTT
1B, S1A	C19orf12	Cas9	Hs.Cas9.C19orf12.1	GCCCACCAAACCCCCGACGA	Alt-R Cas9 crRNA:tra	C19orf12-NT	C*C*AACGGCGAGTCCCGGTC	PS modified	NA	NA	acactctttccctacacgacgctcttccgatctTGTGAGACACCTGCACTTA	gtgactggagttcagacgtgtgctcttccgatctATCATGAAGCTGCTGTGCT
1B, S1A	C19orf33	Cas9	Hs.Cas9.C19orf33.1	GTCCCCACAAAGTTCAGGGC	Alt-R Cas9 crRNA:tra	C19orf33-NT	C*C*ACGGGGGAGATCCCAA	PS modified	NA	NA	acactctttccctacacgacgctcttccgatctCAACCACCCAACTTCTCTC	gtgactggagttcagacgtgtgctcttccgatctGACTTACCTTTGGACCCG
1B, S1A	C19orf60	Cas9	Hs.Cas9.C19orf60.1	GCTACCGGAGCACAGTGCAC	Alt-R Cas9 crRNA:tra	C19orf60-NT	C*G*GCCCTGACTACGACTTC	PS modified	NA	NA	acactctttccctacacgacgctcttccgatctTCAGGGTCCCCACATG	gtgactggagttcagacgtgtgctcttccgatctAGCTCTGCTTCCACCG
1B, S1A	C19orf84	Cas9	Hs.Cas9.C19orf84.1	GGGGGCCTACACCTTCCAAC	Alt-R Cas9 crRNA:tra	C19orf84-NT	T*G*CCTCCTGCACAGCGCCC	PS modified	NA	NA	acactctttccctacacgacgctcttccgatctTGTGCCTGACTTCCCAG	gtgactggagttcagacgtgtgctcttccgatctATACGCCTGGACACCCT
1B, S1A	C1D	Cas9	Hs.Cas9.C1D.1	AACCCAAGGAGTTAATCCTA	Alt-R Cas9 crRNA:tra	C1D-NT	T*T*TTCTCTTGTAGTTTATTT	PS modified	NA	NA	acactctttccctacacgacgctcttccgatctGCTCACTTTCATAATTTCAACTCGAATT	gtgactggagttcagacgtgtgctcttccgatctCATTCACCATTTTATTCCATGAAATTTTT
1B, S1A	C1orf112	Cas9	Hs.Cas9.C1orf112.1	TTATTATGGACTGGTGCTTA	Alt-R Cas9 crRNA:tra	C1orf112-NT	A*T*CTTTGTGTTTCAACTGGC	PS modified	NA	NA	acactctttccctacacgacgctcttccgatctGAGGTCCTAAGTTACTTGATGTGTTA	gtgactggagttcagacgtgtgctcttccgatctAGGAGAAAAGAATGTCTTCACACAA
1B, S1A	C1orf116	Cas9	Hs.Cas9.C1orf116.1	CTCAGCAAGGACGAACGCCA	Alt-R Cas9 crRNA:tra	C1orf116-NT	A*G*GAGGTCCAGAGGAGAC	PS modified	NA	NA	acactctttccctacacgacgctcttccgatctCTTCTGGCAATGTGGATATTC	gtgactggagttcagacgtgtgctcttccgatctTCAGGAAGTCATTGCTTTCC
1B, S1A	C1orf210	Cas9	Hs.Cas9.C1orf210.1	TGTGCTGGTAGGATTTGTGC	Alt-R Cas9 crRNA:tra	C1orf210-NT	G*G*CACTGGGGCTCGGGCA	PS modified	NA	NA	acactctttccctacacgacgctcttccgatctTTTGGCAGCAAGTGCAAT	gtgactggagttcagacgtgtgctcttccgatctGTCCCTTTCTCTGCAGCA
1B, S1A	C1orf228	Cas9	Hs.Cas9.C1orf228.1	CAGGTCTTTGATCAACTCGA	Alt-R Cas9 crRNA:tra	C1orf228-NT	G*C*CTGGCGCACATCGTAAC	PS modified	NA	NA	acactctttccctacacgacgctcttccgatctCTGCACAGTGCTGATCAGTA	gtgactggagttcagacgtgtgctcttccgatctGAGATCTCCTTGACCGACG
1B, S1A	C1orf27	Cas9	Hs.Cas9.C1orf27.1	CACTAGAACGCCACCCAAAG	Alt-R Cas9 crRNA:tra	C1orf27-NT	C*A*AAAGGATTATGTGATTC	PS modified	NA	NA	acactctttccctacacgacgctcttccgatctGCTTTTTAATTTGTTGTTGAAGTGTT	gtgactggagttcagacgtgtgctcttccgatctCATTCTTCATCCAAGTTATCCAACTTA
1B, S1A	C1orf61	Cas9	Hs.Cas9.C1orf61.1	TCTATCGTCCACACGGAGGA	Alt-R Cas9 crRNA:tra	C1orf61-NT	C*T*GTCCGTGGTGCTGGATG	PS modified	NA	NA	acactctttccctacacgacgctcttccgatctCAAAGCTGACTGCAAACAATT	gtgactggagttcagacgtgtgctcttccgatctTCTCTTCCCTTTAGCTTCTCAC
1B, S1A	C1orf68	Cas9	Hs.Cas9.C1orf68.1	CACCCTGGACATAGCACGTC	Alt-R Cas9 crRNA:tra	C1orf68-NT	A*T*AATAGCTCTGGCAAGGA	PS modified	NA	NA	acactctttccctacacgacgctcttccgatctCAACCTATGTAAAATGCCCAA	gtgactggagttcagacgtgtgctcttccgatctCTTGCAGGAGCTTGAACATA
1B, S1A	C1QTNF8	Cas9	Hs.Cas9.C1QTNF8.1	GTTCACCAGCTCCGTGTCGA	Alt-R Cas9 crRNA:tra	C1QTNF8-NT	G*C*CAGGTCGAAGGCGCCG	PS modified	NA	NA	acactctttccctacacgacgctcttccgatctCAGGTGTGCACGTTGAG	gtgactggagttcagacgtgtgctcttccgatctCTACGCCGCCTTCTCC
1B, S1A	C20orf173	Cas9	Hs.Cas9.C20orf173.1	GCCACCACACAGCCGACGAA	Alt-R Cas9 crRNA:tra	C20orf173-NT	T*G*AGACCCTCAACTGCTCC	PS modified	NA	NA	acactctttccctacacgacgctcttccgatctGGTACCCCATAGTCTTCCTG	gtgactggagttcagacgtgtgctcttccgatctTGTACTTGGTACCACAGCATT
1B, S1A	C20orf203	Cas9	Hs.Cas9.C20orf203.1	ACCTCTGGGACCTTGGCGGT	Alt-R Cas9 crRNA:tra	C20orf203-NT	A*G*TCTTGGCTGGACTCACT	PS modified	NA	NA	acactctttccctacacgacgctcttccgatctCCACCAACCCAAATCCTTTC	gtgactggagttcagacgtgtgctcttccgatctCTTGCGAGTCTCAGGTACTAA
1B, S1A	C2CD2	Cas9	Hs.Cas9.C2CD2.1	CGACGCACACTGCATTAATG	Alt-R Cas9 crRNA:tra	C2CD2-NT	C*T*GAACAGGATCGTTCAGC	PS modified	NA	NA	acactctttccctacacgacgctcttccgatctAAGGTGAATTCCTCTTCCCA	gtgactggagttcagacgtgtgctcttccgatctAAAATAAACGCCAACACGATG
1B, S1A	C2orf15	Cas9	Hs.Cas9.C2orf15.1	TGGATGATCACTTAATACGA	Alt-R Cas9 crRNA:tra	C2orf15-NT	T*G*CTATACATATGGATTCA	PS modified	NA	NA	acactctttccctacacgacgctcttccgatctTTCACATTTGTTCAGCTATCCT	gtgactggagttcagacgtgtgctcttccgatctGAAATAACTGAGTCGCTGGTT
1B, S1A	C2orf16	Cas9	Hs.Cas9.C2orf16.1	GGGTTGACCCCAAAGCTAAC	Alt-R Cas9 crRNA:tra	C2orf16-NT	T*A*GATACTGTAGAGAAATC	PS modified	NA	NA	acactctttccctacacgacgctcttccgatctCAGAATCTTCAGAAATGGCACAA	gtgactggagttcagacgtgtgctcttccgatctATTTTGGTACCTGAAGATCTGG
1B, S1A	C2orf70	Cas9	Hs.Cas9.C2orf70.1	CGGCATTGAACTCGGTCAGT	Alt-R Cas9 crRNA:tra	C2orf70-NT	C*A*TGAGTCCAGGGGGCAC	PS modified	NA	NA	acactctttccctacacgacgctcttccgatctAGGCTCGCTGTACTCG	gtgactggagttcagacgtgtgctcttccgatctGAGGAGGGCGCTAGTG
1B, S1A	C2orf74	Cas9	Hs.Cas9.C2orf74.1	CCTTGTACAGATGCAAACGG	Alt-R Cas9 crRNA:tra	C2orf74-NT	A*A*GGTAAAGAGACAAAGA	PS modified	NA	NA	acactctttccctacacgacgctcttccgatctCACCTTAAAATCAGGGCCATT	gtgactggagttcagacgtgtgctcttccgatctATTGCTTGTCACCACTTTGG
1B, S1A	ATAD3A	Cas9	Hs.Cas9.ATAD3A.1	AAGAATGCCACGCTTGTCGC	Alt-R Cas9 crRNA:tra	ATAD3A-NT	T*G*GCTGTTGGGGTCTACTC	PS modified	NA	NA	acactctttccctacacgacgctcttccgatctCAGTGCGGTGTCTCTG	gtgactggagttcagacgtgtgctcttccgatctCGCTGCTACCTGGATG
1B, S1A	ATAD3C	Cas9	Hs.Cas9.ATAD3C.1	GTCAAAGGACGCCCTGAATC	Alt-R Cas9 crRNA:tra	ATAD3C-NT	G*T*GTCCCTGCATCTGCAGG	PS modified	NA	NA	acactctttccctacacgacgctcttccgatctTATCCTAACACCTGCCCTC	gtgactggagttcagacgtgtgctcttccgatctAATCTCCACGCCCGAAC
1B, S1A	A1CF	Cas9	Hs.Cas9.A1CF.1	TAATCGGCAGTTGTCCACAC	Alt-R Cas9 crRNA:tra	A1CF-T	T*T*TTGCAGAAATGGGCGCC	PS modified	NA	NA	acactctttccctacacgacgctcttccgatctCACCTTCAGTAACCTTTTTCATCT	gtgactggagttcagacgtgtgctcttccgatctTCAACACTATGAACCCAAACATC
1B, S1A	A3GALT2	Cas9	Hs.Cas9.A3GALT2.1	GCGCTGGCAAGACGTGTCGA	Alt-R Cas9 crRNA:tra	A3GALT2-T	C*C*GCCCAGCGCCGCGTGCA	PS modified	NA	NA	acactctttccctacacgacgctcttccgatctAAAAGTGCCGCTGAAGTG	gtgactggagttcagacgtgtgctcttccgatctTGTACTACGTGTTCACCGAG
1B, S1A	AADACL3	Cas9	Hs.Cas9.AADACL3.1	GGCATCGTGTACTACCACGG	Alt-R Cas9 crRNA:tra	AADACL3-T	A*T*GGTTCTTACTCAAACTCC	PS modified	NA	NA	acactctttccctacacgacgctcttccgatctGCTCAGAATCTGTTCTATGCC	gtgactggagttcagacgtgtgctcttccgatctGGTCTGAGAAAATGGTTCTTACT
1B, S1A	AAGAB	Cas9	Hs.Cas9.AAGAB.1	CAGCTGGTGACTAACGCACA	Alt-R Cas9 crRNA:tra	AAGAB-T	C*C*GGCTCGCGAGCGCAGC	PS modified	NA	NA	acactctttccctacacgacgctcttccgatctCGCCTATCTACTCACGTTG	gtgactggagttcagacgtgtgctcttccgatctGGTCATTCCGAGAACGC
1B, S1A	ABCA12	Cas9	Hs.Cas9.ABCA12.1	CCACGTTTTGCAACTAACGA	Alt-R Cas9 crRNA:tra	ABCA12-T	C*A*GATGTTTAACAGACTTC	PS modified	NA	NA	acactctttccctacacgacgctcttccgatctACAGCAGATGTTTAACAGACTT	gtgactggagttcagacgtgtgctcttccgatctCTGCAAACCTTGAAACAGAAT
1B, S1A	ABCA9	Cas9	Hs.Cas9.ABCA9.1	GCACAAATTGTCGTCCTGAC	Alt-R Cas9 crRNA:tra	ABCA9-T	A*T*AGAGGAGAAAGAGGGT	PS modified	NA	NA	acactctttccctacacgacgctcttccgatctATTAACCAACTCACCAAAGACAG	gtgactggagttcagacgtgtgctcttccgatctGTTTGATGTATGCTGGCTTCAT
1B, S1A	ABCB11	Cas9	Hs.Cas9.ABCB11.1	CGCATGACCTCGACCATCTG	Alt-R Cas9 crRNA:tra	ABCB11-T	C*A*GTTTCCAACCCCTGAAA	PS modified	NA	NA	acactctttccctacacgacgctcttccgatctAGAGGGCTGACAGAAATAATAAC	gtgactggagttcagacgtgtgctcttccgatctAAAATCAATGATGCCATAGCTGA
1B, S1A	ABCB9	Cas9	Hs.Cas9.ABCB9.1	ACCCTCGTGTGCCTCTTCGT	Alt-R Cas9 crRNA:tra	ABCB9-T	T*G*AGAAGAGCAGCAGCTTC	PS modified	NA	NA	acactctttccctacacgacgctcttccgatctGCCGAGTGAAATGTACGTC	gtgactggagttcagacgtgtgctcttccgatctCATTGGTGTGGCCAAGAA
1B, S1A	ABCC10	Cas9	Hs.Cas9.ABCC10.1	TGCCAGATAGCACCGTCCAA	Alt-R Cas9 crRNA:tra	ABCC10-T	G*A*GGGGGCACGGCTGTGG	PS modified	NA	NA	acactctttccctacacgacgctcttccgatctCACAGACTGCAGCCAAC	gtgactggagttcagacgtgtgctcttccgatctGAATCCCAACATGGTCCC
1B, S1A	ABCD1	Cas9	Hs.Cas9.ABCD1.1	GGCGGGCCACATACACCGAC	Alt-R Cas9 crRNA:tra	ABCD1-T	C*C*CTGCACTCGGCCGCCTT	PS modified	NA	NA	acactctttccctacacgacgctcttccgatctCAAAGCTGGCATGAACC	gtgactggagttcagacgtgtgctcttccgatctCGATGAGGAGCCACTG
1B, S1A	ABCD3	Cas9	Hs.Cas9.ABCD3.1	ACTCGACTTCGAAGACCCAT	Alt-R Cas9 crRNA:tra	ABCD3-T	T*C*CTTCATACTTTTGCTCAG	PS modified	NA	NA	acactctttccctacacgacgctcttccgatctCCCAGAACTTTGTGTATCTTTCT	gtgactggagttcagacgtgtgctcttccgatctGTCTTTACCTGTTTGTGATGAGC
1B, S1A	ABCD4	Cas9	Hs.Cas9.ABCD4.1	CTGGTAAGTGTAGTAGACGA	Alt-R Cas9 crRNA:tra	ABCD4-T	A*G*CATGGCCAGCAAGCTCA	PS modified	NA	NA	acactctttccctacacgacgctcttccgatctTAAGCTTCTCTTGGACCTTGA	gtgactggagttcagacgtgtgctcttccgatctTCCAGCTACTCTGTGTCATT
1B, S1A	ABCG2	Cas9	Hs.Cas9.ABCG2.1	ACCTGGTCTCAACGCCATCC	Alt-R Cas9 crRNA:tra	ABCG2-T	T*A*TACTCACGAAGATTTGC	PS modified	NA	NA	acactctttccctacacgacgctcttccgatctCACATTAAAAGTGCACAGAAAACG	gtgactggagttcagacgtgtgctcttccgatctCAGTGCCTACCCTAAATTAATAGAA
1B, S1A	ABCG4	Cas9	Hs.Cas9.ABCG4.1	TCGTGTGGGAGCACGACATC	Alt-R Cas9 crRNA:tra	ABCG4-T	G*G*GGCCAGGTGACAGAGA	PS modified	NA	NA	acactctttccctacacgacgctcttccgatctAGACTCCGAAGCTGACCT	gtgactggagttcagacgtgtgctcttccgatctTTGTTGACCAGCTCCAGG
1B, S1A	ABCG5	Cas9	Hs.Cas9.ABCG5.1	CATGTGGCAGACCGACTGAT	Alt-R Cas9 crRNA:tra	ABCG5-T	C*T*CACCCGTGGAAATGCCC	PS modified	NA	NA	acactctttccctacacgacgctcttccgatctGCTAGTAACAGTTCTGGGTG	gtgactggagttcagacgtgtgctcttccgatctCCCATGAATTGAAATAGCAGC
1B, S1A	ABHD11	Cas9	Hs.Cas9.ABHD11.1	CGTGCAAAAAGACGACGGCC	Alt-R Cas9 crRNA:tra	ABHD11-T	T*T*TCCTACAGGCTTCTGGA	PS modified	NA	NA	acactctttccctacacgacgctcttccgatctCAAGATCTTGGCGATGGA	gtgactggagttcagacgtgtgctcttccgatctTCAGACTCTGACCTCGATC
1B, S1A	ABHD15	Cas9	Hs.Cas9.ABHD15.1	ATACATCCGCTTCCGACACC	Alt-R Cas9 crRNA:tra	ABHD15-T	C*C*CGAGCCTTCGCTCACCG	PS modified	NA	NA	acactctttccctacacgacgctcttccgatctACTCGCCCAGGTAGGA	gtgactggagttcagacgtgtgctcttccgatctGCTACTACCCGGTCATCTT
1B, S1A	ABHD5	Cas9	Hs.Cas9.ABHD5.1	TTGGACGAAGTAGTAGACCC	Alt-R Cas9 crRNA:tra	ABHD5-T	A*C*TGATTCTCCACTTCTTCT	PS modified	NA	NA	acactctttccctacacgacgctcttccgatctGGCACTGAATTTTGGAGATCTTTG	gtgactggagttcagacgtgtgctcttccgatctCACATCTCCACTCTTCAATGGATT
1B, S1A	ABHD6	Cas9	Hs.Cas9.ABHD6.1	GATTGTCAGTTGAGTACTGC	Alt-R Cas9 crRNA:tra	ABHD6-T	T*A*TCATCAAGCCATTTGGG	PS modified	NA	NA	acactctttccctacacgacgctcttccgatctTATTAACTCTGGGCTGCTGT	gtgactggagttcagacgtgtgctcttccgatctGACATAGGAGCAGAGCTGAAG
1B, S1A	ABI3	Cas9	Hs.Cas9.ABI3.1	GCCTGGACGACATTGGCCAT	Alt-R Cas9 crRNA:tra	ABI3-T	A*G*GGAAAGAGGAGGGCCC	PS modified	NA	NA	acactctttccctacacgacgctcttccgatctAAGGTCATCGCCCCAGA	gtgactggagttcagacgtgtgctcttccgatctGGGAAAGAGGAGGGCC
1B, S1A	ABL2	Cas9	Hs.Cas9.ABL2.1	AGGGACGTGTGTATCACTAC	Alt-R Cas9 crRNA:tra	ABL2-T	G*G*AGTCTTACCTTGCCATC	PS modified	NA	NA	acactctttccctacacgacgctcttccgatctTGACCAGGGAGTCTTACCTT	gtgactggagttcagacgtgtgctcttccgatctCAGCAGTCTAATCAATGGCAG
1B, S1A	ABLIM2	Cas9	Hs.Cas9.ABLIM2.1	TCGACACGCCGGATGCCAGA	Alt-R Cas9 crRNA:tra	ABLIM2-T	C*A*TTTCCCTCTTGGGAATTT	PS modified	NA	NA	acactctttccctacacgacgctcttccgatctCCACCAACCTTGTTTCTGT	gtgactggagttcagacgtgtgctcttccgatctCCCTGCAACATGGGAAATAA
1B, S1A	ABRAXAS1	Cas9	Hs.Cas9.ABRAXAS1.1	AAGCTGCTCTACTCATCGAC	Alt-R Cas9 crRNA:tra	ABRAXAS1-T	A*T*TTACCCTTTTTGAGGTTT	PS modified	NA	NA	acactctttccctacacgacgctcttccgatctCAGTGACTGGCCAACATTTA	gtgactggagttcagacgtgtgctcttccgatctCTGCTTCACAAAAACTTGCAG
1B, S1A	ACAT1	Cas9	Hs.Cas9.ACAT1.1	TCAAGCTTTACCCCACCATA	Alt-R Cas9 crRNA:tra	ACAT1-T	C*A*ATGTTCCATATGTAATGA	PS modified	NA	NA	acactctttccctacacgacgctcttccgatctGGGTTTGCAAAATATTTGTATTAACATT	gtgactggagttcagacgtgtgctcttccgatctCAGTATTGGACATTAGATAGCATTTAT
1B, S1A	ACAT2	Cas9	Hs.Cas9.ACAT2.1	GCCGCCGAGACGATGACCAC	Alt-R Cas9 crRNA:tra	ACAT2-T	C*G*GCAGGAGAAGCAAGAT	PS modified	NA	NA	acactctttccctacacgacgctcttccgatctCGGCAGGAGAAGCAAGAT	gtgactggagttcagacgtgtgctcttccgatctCATACGCCCCTCTCCTACA
1B, S1A	ACCSL	Cas9	Hs.Cas9.ACCSL.1	GGATAGGTCGCGGTTGACAA	Alt-R Cas9 crRNA:tra	ACCSL-T	G*A*TCCAGTTCCCCAACTGA	PS modified	NA	NA	acactctttccctacacgacgctcttccgatctAAGTGCCTCTTCCTTCTGAG	gtgactggagttcagacgtgtgctcttccgatctTAGGCATTGTAGTCCTGGAAG
1B, S1A	ACER2	Cas9	Hs.Cas9.ACER2.1	GCATCTGACCCAAGAAACTA	Alt-R Cas9 crRNA:tra	ACER2-T	T*T*CTAGGAATTGGATCCGT	PS modified	NA	NA	acactctttccctacacgacgctcttccgatctGTTGTAATTGATTCTAGGAATTGGAT	gtgactggagttcagacgtgtgctcttccgatctCATTCCGAAAGATCTTTGGTAGATA
1B, S1A	ACKR3	Cas9	Hs.Cas9.ACKR3.1	TTGCACGTGAGCTCGCCCAT	Alt-R Cas9 crRNA:tra	ACKR3-T	C*T*GGGTGGTCAGTCTCGTG	PS modified	NA	NA	acactctttccctacacgacgctcttccgatctCTGCTACATCTTGAACCTGG	gtgactggagttcagacgtgtgctcttccgatctTTGGTGAAGTAGGTGATGGA
1B, S1A	ACOT13	Cas9	Hs.Cas9.ACOT13.1	GCAATAGGCACTCTCCACGG	Alt-R Cas9 crRNA:tra	ACOT13-T	T*G*TTGATATGTTATCTACTA	PS modified	NA	NA	acactctttccctacacgacgctcttccgatctCTTAGATTACTCTTGTCTCTGCTG	gtgactggagttcagacgtgtgctcttccgatctGCAGAGCCATTGTTGATATGTTAT
1B, S1A	ACOT2	Cas9	Hs.Cas9.ACOT2.1	GAGCGTCCCGGCTGTACCAA	Alt-R Cas9 crRNA:tra	ACOT2-T	G*A*GACCCCAGGAACTGCGC	PS modified	NA	NA	acactctttccctacacgacgctcttccgatctCCCCATTCAGTTGTTCTCAG	gtgactggagttcagacgtgtgctcttccgatctAACCCTAATGATCTGACCAAC
1B, S1A	ACP2	Cas9	Hs.Cas9.ACP2.1	GTCAGGATGACCGAATACGT	Alt-R Cas9 crRNA:tra	ACP2-T	T*T*CAACCACTTCCCTGTCTC	PS modified	NA	NA	acactctttccctacacgacgctcttccgatctCATTCAACCACTTCCCTGT	gtgactggagttcagacgtgtgctcttccgatctGTAATGAGAGATGGGCTCAC
1B, S1A	ACP5	Cas9	Hs.Cas9.ACP5.1	TTTCCGGCTAGCACGTACCA	Alt-R Cas9 crRNA:tra	ACP5-T	G*G*ACGTATTCTCTGACCGC	PS modified	NA	NA	acactctttccctacacgacgctcttccgatctTAGAGTATGCAATCTGGGCA	gtgactggagttcagacgtgtgctcttccgatctCTACAGGAGACCTTTGAGGA
1B, S1A	ACR	Cas9	Hs.Cas9.ACR.1	ATGAAGCGCCCACACGAAAT	Alt-R Cas9 crRNA:tra	ACR-T	G*G*GAAATGACATTGCCCTC	PS modified	NA	NA	acactctttccctacacgacgctcttccgatctACAGAGGGAAATGACATTGC	gtgactggagttcagacgtgtgctcttccgatctTTCTATATATCCCCAGCCGG
1B, S1A	ACRBP	Cas9	Hs.Cas9.ACRBP.1	AAGAAGCGTTCGTATTCGGT	Alt-R Cas9 crRNA:tra	ACRBP-T	G*A*CTCAGGCCTCCACTCCA	PS modified	NA	NA	acactctttccctacacgacgctcttccgatctCAGGTAGTCTCTGCCTTC	gtgactggagttcagacgtgtgctcttccgatctCAGGATTCGACTCAGGC
1B, S1A	ACSL5	Cas9	Hs.Cas9.ACSL5.1	GGCTTGTTACACGTACTCTA	Alt-R Cas9 crRNA:tra	ACSL5-T	T*C*TGGTCCCAAGGTGTCAT	PS modified	NA	NA	acactctttccctacacgacgctcttccgatctCCTCCAGTCCTTACTTGAACTT	gtgactggagttcagacgtgtgctcttccgatctTATGTACGATGGCTTCTGGTC
1B, S1A	ACSM4	Cas9	Hs.Cas9.ACSM4.1	AATACAATGGACTCCACCGC	Alt-R Cas9 crRNA:tra	ACSM4-T	G*G*CCAAGTGCATTGTGGCC	PS modified	NA	NA	acactctttccctacacgacgctcttccgatctCTGACAGCAAAAGACATCCT	gtgactggagttcagacgtgtgctcttccgatctACCCATTCCAGCTTTGTG
1B, S1A	ACSS2	Cas9	Hs.Cas9.ACSS2.1	GTCTCTATGTGAACGGATCT	Alt-R Cas9 crRNA:tra	ACSS2-T	T*C*ACCTGTAGTGATGAGAA	PS modified	NA	NA	acactctttccctacacgacgctcttccgatctCCTCAGACTTTCTTCCCTTC	gtgactggagttcagacgtgtgctcttccgatctGTAGCCCCAAATACCAATGA
1B, S1A	ACTA2	Cas9	Hs.Cas9.ACTA2.1	TGGGACGTCCCACAATGGAT	Alt-R Cas9 crRNA:tra	ACTA2-T	G*C*TTTGCTGGGGACGATGC	PS modified	NA	NA	acactctttccctacacgacgctcttccgatctCAGAGGAACCTAATCTGTGT	gtgactggagttcagacgtgtgctcttccgatctCTCCAGCTATGTGTGAAGAA
1B, S1A	ACTN1	Cas9	Hs.Cas9.ACTN1.1	GTGCTTTGATCCACCGACAC	Alt-R Cas9 crRNA:tra	ACTN1-T	C*C*TTCCGCAGCTTCCCGTAC	PS modified	NA	NA	acactctttccctacacgacgctcttccgatctATACCTTCCGCAGCTTCC	gtgactggagttcagacgtgtgctcttccgatctAGCCTCATTTACCAGCCTG
1B, S1A	ACTRT1	Cas9	Hs.Cas9.ACTRT1.1	GAGGGCTCGGTCATAAGTAC	Alt-R Cas9 crRNA:tra	ACTRT1-T	T*G*AGCGGGAGCTTGGAGT	PS modified	NA	NA	acactctttccctacacgacgctcttccgatctCACCGCATGATTAGACAGGTA	gtgactggagttcagacgtgtgctcttccgatctTATGAGGCCCTACATTTGCA
1B, S1A	ACVR1C	Cas9	Hs.Cas9.ACVR1C.1	TGTAGGAGCACTGTCGACCC	Alt-R Cas9 crRNA:tra	ACVR1C-T	T*G*TCCATAGCTGCGATGCT	PS modified	NA	NA	acactctttccctacacgacgctcttccgatctCAGCATTTACCAGATTGCACT	gtgactggagttcagacgtgtgctcttccgatctGCTGGCCATCATTATTACTGTG
1B, S1A	ACVR2A	Cas9	Hs.Cas9.ACVR2A.1	ACTGGTGTTGAACCGTGTTA	Alt-R Cas9 crRNA:tra	ACVR2A-T	G*G*TAGCAAAACAATGCCGC	PS modified	NA	NA	acactctttccctacacgacgctcttccgatctTAGGTGCTATACTTGGTAGATCAGAAA	gtgactggagttcagacgtgtgctcttccgatctCAGAAATATTCTTCCAGGTAGCAAAA
1B, S1A	ACY3	Cas9	Hs.Cas9.ACY3.1	CACCTCATATGGGTCGTCCG	Alt-R Cas9 crRNA:tra	ACY3-T	G*T*CCCCCATTCCCGTCTCCA	PS modified	NA	NA	acactctttccctacacgacgctcttccgatctGCAGGTCAAGGACAAAGT	gtgactggagttcagacgtgtgctcttccgatctCCCCTTAAACCACTGAAGA
1B, S1A	ADAM30	Cas9	Hs.Cas9.ADAM30.1	TACGAGTCAAACTCCCCTTC	Alt-R Cas9 crRNA:tra	ADAM30-T	C*C*TTAAGTCTCTTGGCGAA	PS modified	NA	NA	acactctttccctacacgacgctcttccgatctCTTTTAACTGCAGTAGGTAGGA	gtgactggagttcagacgtgtgctcttccgatctGACAATGCTCCTTAAGTCTCTT
1B, S1A	ADAM33	Cas9	Hs.Cas9.ADAM33.1	CCACGTAGTTGGCGACTTCC	Alt-R Cas9 crRNA:tra	ADAM33-T	G*G*CACCGAAACTTGAACCA	PS modified	NA	NA	acactctttccctacacgacgctcttccgatctTCTTCCCCAAACCCCAC	gtgactggagttcagacgtgtgctcttccgatctTGCCTTCCCAGTTCTTGA
1B, S1A	ADAM7	Cas9	Hs.Cas9.ADAM7.1	GCCAGTATCAGTACGTGTAA	Alt-R Cas9 crRNA:tra	ADAM7-T	A*A*GGAAAAAGAAAGATAT	PS modified	NA	NA	acactctttccctacacgacgctcttccgatctTCATTGTTTTTACCAAGGATCCAT	gtgactggagttcagacgtgtgctcttccgatctTAAATCATAGGCTACAGCTGAAA
1B, S1A	ADAMTS12	Cas9	Hs.Cas9.ADAMTS12.1	CTCGGACTGGACCCACCACG	Alt-R Cas9 crRNA:tra	ADAMTS12-T	T*T*TCAGAGCATTTTATCAAC	PS modified	NA	NA	acactctttccctacacgacgctcttccgatctGAAATTCTGTAGTACACCCAGTC	gtgactggagttcagacgtgtgctcttccgatctGGCTTTGCTTTACTACCAATCTC
1B, S1A	ADCY2	Cas9	Hs.Cas9.ADCY2.1	GACGCTAAGCACGATGGTGT	Alt-R Cas9 crRNA:tra	ADCY2-T	G*A*CGCCATCATTGCCAGCG	PS modified	NA	NA	acactctttccctacacgacgctcttccgatctGCCCGTAGGTATCGTTCTTC	gtgactggagttcagacgtgtgctcttccgatctTGGGATGTGGAAAGTCATTCT
1B, S1A	ADGRB3	Cas9	Hs.Cas9.ADGRB3.1	TAACCGAACATGTGCTCCAC	Alt-R Cas9 crRNA:tra	ADGRB3-T	T*G*CACGCAGGTGAATCTGC	PS modified	NA	NA	acactctttccctacacgacgctcttccgatctCATGTTTATTTGTTGTCTTGCACG	gtgactggagttcagacgtgtgctcttccgatctCGTAAGGTGATACACAAGTTCTG
1B, S1A	ADGRF1	Cas9	Hs.Cas9.ADGRF1.1	TCAAGGTTTTGAGTCGGTTC	Alt-R Cas9 crRNA:tra	ADGRF1-T	T*A*AGACCATTGTGTTACTTA	PS modified	NA	NA	acactctttccctacacgacgctcttccgatctGCCCTTCTAAGACCATTGTGTTA	gtgactggagttcagacgtgtgctcttccgatctGCTCAAATGTCTTTCTGAAGTACG
1B, S1A	ADGRG1	Cas9	Hs.Cas9.ADGRG1.1	ACCGGATCAACGCCACGGTG	Alt-R Cas9 crRNA:tra	ADGRG1-T	G*C*AGGTCCTGGAGGCCGG	PS modified	NA	NA	acactctttccctacacgacgctcttccgatctTGGAGTCGAAACTGACCT	gtgactggagttcagacgtgtgctcttccgatctGAGTGGATGTGCAGGTC
1B, S1A	ADGRL1	Cas9	Hs.Cas9.ADGRL1.1	CTACGGACGCGCATCAAGAG	Alt-R Cas9 crRNA:tra	ADGRL1-T	G*T*CATGGTAGTTGGCGGTA	PS modified	NA	NA	acactctttccctacacgacgctcttccgatctGAGGTGTCATGGTAGTTGG	gtgactggagttcagacgtgtgctcttccgatctTGCCGTCTTCTACAACAA
1B, S1A	ADGRL2	Cas9	Hs.Cas9.ADGRL2.1	TATTAAAGTATCGGTACGAT	Alt-R Cas9 crRNA:tra	ADGRL2-T	G*C*TGCAGATAAAATTTATT	PS modified	NA	NA	acactctttccctacacgacgctcttccgatctGACCTTGAAAGCAATTGTGGA	gtgactggagttcagacgtgtgctcttccgatctCCATCATACACCACAAATCCA
1B, S1A	ADGRL3	Cas9	Hs.Cas9.ADGRL3.1	TTTGAGTCCGACCACCAATC	Alt-R Cas9 crRNA:tra	ADGRL3-T	G*T*CAGATGCCTGCAGAGGC	PS modified	NA	NA	acactctttccctacacgacgctcttccgatctCCTTCCAACAGTTTTTCTTTGTC	gtgactggagttcagacgtgtgctcttccgatctGATGAATACTCAGTCAGGGTATCA
1B, S1A	ADI1	Cas9	Hs.Cas9.ADI1.1	CTACGAGGAGCATTTGCACT	Alt-R Cas9 crRNA:tra	ADI1-T	C*C*ACTGCCATCCAGGATGT	PS modified	NA	NA	acactctttccctacacgacgctcttccgatctGAAGTACCCACTGCCATC	gtgactggagttcagacgtgtgctcttccgatctGTGACAGGTCTCTGGTAGA
1B, S1A	ADIRF	Cas9	Hs.Cas9.ADIRF.1	CTTGCAGGACCTGAAGCAAC	Alt-R Cas9 crRNA:tra	ADIRF-T	C*T*ATCCTCACCGGCTTCCTG	PS modified	NA	NA	acactctttccctacacgacgctcttccgatctAGCTGCTCTTGACGACT	gtgactggagttcagacgtgtgctcttccgatctTAGGTCGCGCGCTATC
1B, S1A	ADNP	Cas9	Hs.Cas9.ADNP.1	CCTGATAGCCTATACGTTCA	Alt-R Cas9 crRNA:tra	ADNP-T	C*C*TATGAAGCTTTGGTACA	PS modified	NA	NA	acactctttccctacacgacgctcttccgatctGCAATTAGCATCAAGGGTTTG	gtgactggagttcagacgtgtgctcttccgatctCCTTTTCATGCCAAAGTCCTAT
1B, S1A	ADPGK	Cas9	Hs.Cas9.ADPGK.1	AGCCCAAGGGAAGTCACCGC	Alt-R Cas9 crRNA:tra	ADPGK-T	A*G*ATACCTGTTGTTGTTTTT	PS modified	NA	NA	acactctttccctacacgacgctcttccgatctCTTTTACTCCTCCCATGTTCTTT	gtgactggagttcagacgtgtgctcttccgatctGTCATAAGACATCTTAGGAGCCA
1B, S1A	ADRA2C	Cas9	Hs.Cas9.ADRA2C.1	GCGGCCTCAACGACGAGACC	Alt-R Cas9 crRNA:tra	ADRA2C-T	C*G*AAGAAGGAGCCGATGC	PS modified	NA	NA	acactctttccctacacgacgctcttccgatctTGTGGCTCATCTCGGC	gtgactggagttcagacgtgtgctcttccgatctATGATGAGGCAGGGCG
1B, S1A	ADRB3	Cas9	Hs.Cas9.ADRB3.1	CAACGTGTTCGTGACTTCGC	Alt-R Cas9 crRNA:tra	ADRB3-T	A*C*CACCAGGAGTCCCATCA	PS modified	NA	NA	acactctttccctacacgacgctcttccgatctACCACCAGGAGTCCCAT	gtgactggagttcagacgtgtgctcttccgatctAACCTGCTGGTCATCGTG
1B, S1A	AEBP1	Cas9	Hs.Cas9.AEBP1.1	GAACTCCTCGATCTCGTCGT	Alt-R Cas9 crRNA:tra	AEBP1-T	C*T*GTGCCCTGGAGGGCGCC	PS modified	NA	NA	acactctttccctacacgacgctcttccgatctCTCAGCTGCCTCCTGG	gtgactggagttcagacgtgtgctcttccgatctGGGCTCAGGTTCTAGCT
1B, S1A	AFAP1	Cas9	Hs.Cas9.AFAP1.1	ATAAGAGCTGCTCATCGCAT	Alt-R Cas9 crRNA:tra	AFAP1-T	A*C*CAGTGTATGTTTATTTTT	PS modified	NA	NA	acactctttccctacacgacgctcttccgatctGTTTTCTTCCCCTTCCCATC	gtgactggagttcagacgtgtgctcttccgatctGGTCCCTTTCTCATTCAGTTA
1B, S1A	AFG3L2	Cas9	Hs.Cas9.AFG3L2.1	AAGGCGATGATGAGCACCGT	Alt-R Cas9 crRNA:tra	AFG3L2-T	G*T*GTGTCCCCAGCTCTTTTC	PS modified	NA	NA	acactctttccctacacgacgctcttccgatctCCTTGGCAGTGGTTTCTC	gtgactggagttcagacgtgtgctcttccgatctAGTCCTAACCCGTGTTGC
1B, S1A	AGAP3	Cas9	Hs.Cas9.AGAP3.1	TGGTGCACCGCTATCTGACG	Alt-R Cas9 crRNA:tra	AGAP3-T	G*C*TCACCTTCAGGGGACTC	PS modified	NA	NA	acactctttccctacacgacgctcttccgatctTCTGCCCTCTGACCCA	gtgactggagttcagacgtgtgctcttccgatctGGCCTGTGACGCTCAC
1B, S1A	AGAP6	Cas9	Hs.Cas9.AGAP6.1	TGGAATATTGTGCTTGACTC	Alt-R Cas9 crRNA:tra	AGAP6-T	A*C*ATATAGCTTTGGAGTTTA	PS modified	NA	NA	acactctttccctacacgacgctcttccgatctCTCCCTCTATACATATAGCTTTGGA	gtgactggagttcagacgtgtgctcttccgatctCTCAAAGAAGCCTAAACAAAGTAC
1B, S1A	AGAP9	Cas9	Hs.Cas9.AGAP9.1	TGGTGGTGCTGGAGATACCG	Alt-R Cas9 crRNA:tra	AGAP9-T	T*G*GACACCGGGCTGGGTGA	PS modified	NA	NA	acactctttccctacacgacgctcttccgatctTTCAAAGTGCCACGTTTG	gtgactggagttcagacgtgtgctcttccgatctCTAGCCACATCAGCCT
1B, S1A	AGBL5	Cas9	Hs.Cas9.AGBL5.1	ATTCCCATGTTGAACCCCGA	Alt-R Cas9 crRNA:tra	AGBL5-T	G*G*AAGCCACTTACCGGTAC	PS modified	NA	NA	acactctttccctacacgacgctcttccgatctATGGCTTTCTGGACTTCATC	gtgactggagttcagacgtgtgctcttccgatctTATTAGGGTGCAAGAGGACTG
1B, S1A	AGO1	Cas9	Hs.Cas9.AGO1.1	GATCGACGTGTACCACTACG	Alt-R Cas9 crRNA:tra	AGO1-T	A*C*TCTACGGGGACACTTAT	PS modified	NA	NA	acactctttccctacacgacgctcttccgatctGAAACCAATCAAGCTCCTGG	gtgactggagttcagacgtgtgctcttccgatctTGTGCATCACTTACCGGTT
1B, S1A	AGO3	Cas9	Hs.Cas9.AGO3.1	GTAGCACCACATCAACGGCA	Alt-R Cas9 crRNA:tra	AGO3-T	T*G*GAATTAGACAAGCCAAT	PS modified	NA	NA	acactctttccctacacgacgctcttccgatctGCCACTGGAATTAGACAAGC	gtgactggagttcagacgtgtgctcttccgatctAAAGGTTTAACCAAGGGAAGC
1B, S1A	AGPAT1	Cas9	Hs.Cas9.AGPAT1.1	CATCGACCGGAAGCGCACGG	Alt-R Cas9 crRNA:tra	AGPAT1-T	G*T*CTGGGCGACCTCAGACA	PS modified	NA	NA	acactctttccctacacgacgctcttccgatctCCATTTCCCCAGGATGA	gtgactggagttcagacgtgtgctcttccgatctGAGCTACTGTGGGCTG
1B, S1A	AGPS	Cas9	Hs.Cas9.AGPS.1	GTACCAATGAGTGCAAAGCG	Alt-R Cas9 crRNA:tra	AGPS-T	C*C*GTGGGCGCTGCCGTGGC	PS modified	NA	NA	acactctttccctacacgacgctcttccgatctGTTCTCTCTGGCCATCTG	gtgactggagttcagacgtgtgctcttccgatctGCTACTCACCGCTTCTTTG
1B, S1A	AHCTF1	Cas9	Hs.Cas9.AHCTF1.1	AAGGATAACATCGTTACCAC	Alt-R Cas9 crRNA:tra	AHCTF1-T	C*T*CAGATATATTCAGACAAT	PS modified	NA	NA	acactctttccctacacgacgctcttccgatctGCAGAACCCACTAATACAAAGGA	gtgactggagttcagacgtgtgctcttccgatctCACAGACAAGCCCTCAGATATATT
1B, S1A	AHCYL1	Cas9	Hs.Cas9.AHCYL1.1	CGGATCTTCTTAAACACGTT	Alt-R Cas9 crRNA:tra	AHCYL1-T	G*G*GAGACTTAACCCACTGC	PS modified	NA	NA	acactctttccctacacgacgctcttccgatctGTCTTCTGGCTGTTCTATAGATC	gtgactggagttcagacgtgtgctcttccgatctTCCAGAATCTGTTACCTGTGAA
1B, S1A	AHSG	Cas9	Hs.Cas9.AHSG.1	GGCCCCGCTGAACGACACCA	Alt-R Cas9 crRNA:tra	AHSG-T	A*A*GGCGGCCAGGGCAGCT	PS modified	NA	NA	acactctttccctacacgacgctcttccgatctTGGTTTCCTCTCTCCGAG	gtgactggagttcagacgtgtgctcttccgatctTCCAGCTGAAAATTGGAGC
1B, S1A	AHSP	Cas9	Hs.Cas9.AHSP.1	TGCGGAAATGAGATCCTTAT	Alt-R Cas9 crRNA:tra	AHSP-T	T*T*TTCTCTACCCAGGCAGA	PS modified	NA	NA	acactctttccctacacgacgctcttccgatctCTACCCTTTTCTCTACCCAGG	gtgactggagttcagacgtgtgctcttccgatctAAATGGAAAGCTTGGACTCAC
1B, S1A	AIDA	Cas9	Hs.Cas9.AIDA.1	CCAAGGTTGCCATCGGAACC	Alt-R Cas9 crRNA:tra	AIDA-T	A*A*TTTTCTCAATTCTGATAG	PS modified	NA	NA	acactctttccctacacgacgctcttccgatctCCAGCATCTTTCAAACCAATTTT	gtgactggagttcagacgtgtgctcttccgatctACTGTGAAATGATATGGAGCTTTT
1B, S1A	AK1	Cas9	Hs.Cas9.AK1.1	TCCTGATTGATGGCTACCCG	Alt-R Cas9 crRNA:tra	AK1-T	C*C*CGTCGCTCAAACTCTTCT	PS modified	NA	NA	acactctttccctacacgacgctcttccgatctTTGGGTCAGTGCCTTAC	gtgactggagttcagacgtgtgctcttccgatctGAGACAGTGTTGGACATG
1B, S1A	AK2	Cas9	Hs.Cas9.AK2.1	GAGTGGCCGTTCCTACCACG	Alt-R Cas9 crRNA:tra	AK2-T	T*C*ATCTTTCATGGGCTCTTT	PS modified	NA	NA	acactctttccctacacgacgctcttccgatctTCCTGAGTTTACATACGTCATCTTT	gtgactggagttcagacgtgtgctcttccgatctCGTACTGTCAGTTAACCTAACTCAT
1B, S1A	AKAIN1	Cas9	Hs.Cas9.AKAIN1.1	ATTCTGCACAATCTGTTTGC	Alt-R Cas9 crRNA:tra	AKAIN1-T	G*G*AAATGAGCCTGAAGAG	PS modified	NA	NA	acactctttccctacacgacgctcttccgatctGTCACTGATTCTCTCTTCTCTG	gtgactggagttcagacgtgtgctcttccgatctTAGGTGAGAAACCTGGAAATGA
1B, S1A	AKAP10	Cas9	Hs.Cas9.AKAP10.1	AGAAGCGGGACTATTTCTAC	Alt-R Cas9 crRNA:tra	AKAP10-T	G*A*AACCCAAGAATCTTCCT	PS modified	NA	NA	acactctttccctacacgacgctcttccgatctCACATATGTACACACAAGAAAATCACATA	gtgactggagttcagacgtgtgctcttccgatctAATAATAGAACTAACAGCACTCAGAATCA
1B, S1A	AKAP6	Cas9	Hs.Cas9.AKAP6.1	ACGCCAATGGCAACTACACT	Alt-R Cas9 crRNA:tra	AKAP6-T	T*C*TCTTCAGAGAAAGCTTG	PS modified	NA	NA	acactctttccctacacgacgctcttccgatctCTTGAAACAGAGTTCTCCCTAAAG	gtgactggagttcagacgtgtgctcttccgatctGCTTAACTTCAAGGAAAACACTCA
1B, S1A	AKIRIN1	Cas9	Hs.Cas9.AKIRIN1.1	AAGAATATAGTCGTTATCAG	Alt-R Cas9 crRNA:tra	AKIRIN1-T	C*A*CTCTGATTAAGAACAAC	PS modified	NA	NA	acactctttccctacacgacgctcttccgatctAAGATCTTTGTCTCTTCCCACTT	gtgactggagttcagacgtgtgctcttccgatctCACAAGCTTCACTCTGATTAAGAA
1B, S1A	AKR1B10	Cas9	Hs.Cas9.AKR1B10.1	GAACGTTGCTTTTCCACCGA	Alt-R Cas9 crRNA:tra	AKR1B10-T	G*A*TGACCTTTTCCCCAAAGA	PS modified	NA	NA	acactctttccctacacgacgctcttccgatctAGATGTGGTATTTAGCAAGAGTCA	gtgactggagttcagacgtgtgctcttccgatctCATGGATAACTGAAGATTTCTCTCC
1B, S1A	ALDH3A1	Cas9	Hs.Cas9.ALDH3A1.1	GGACACCCCCATTGATTACT	Alt-R Cas9 crRNA:tra	ALDH3A1-T	G*C*CTCCCCTCTCTCCTTGCA	PS modified	NA	NA	acactctttccctacacgacgctcttccgatctTCCAGGTCACAGTTCTTGT	gtgactggagttcagacgtgtgctcttccgatctTGGTCTTTGCTGAGCCTC
1B, S1A	ALDOA	Cas9	Hs.Cas9.ALDOA.1	ACGGAGCTGACTTCGCCAAG	Alt-R Cas9 crRNA:tra	ALDOA-T	A*G*GGGGTGTGTTCCCCAAT	PS modified	NA	NA	acactctttccctacacgacgctcttccgatctTCTTCTCTTAGGGTTGGATGG	gtgactggagttcagacgtgtgctcttccgatctAACATTGGCATTTTCCATGATG
1B, S1A	ALG13	Cas9	Hs.Cas9.ALG13.1	TCGTTTATAACCACTACGAG	Alt-R Cas9 crRNA:tra	ALG13-T	A*G*GAAGCTGTTTGGAGACT	PS modified	NA	NA	acactctttccctacacgacgctcttccgatctTATTTGTAGGTGCAGGAAGCT	gtgactggagttcagacgtgtgctcttccgatctTGACCCTCTTTGTGTAGCTG
1B, S1A	ALG14	Cas9	Hs.Cas9.ALG14.1	TTCCATGGACGTTACGCCCC	Alt-R Cas9 crRNA:tra	ALG14-T	C*C*GGACCCAGCCACTACCA	PS modified	NA	NA	acactctttccctacacgacgctcttccgatctTCGCCTCCTCGATACTTAC	gtgactggagttcagacgtgtgctcttccgatctTGTGCGTTCTCGTTCTAG
1B, S1A	ALG1L	Cas9	Hs.Cas9.ALG1L.1	GTGGCACTCACTCTCTGTTC	Alt-R Cas9 crRNA:tra	ALG1L-T	G*A*GAAGGCAGCTGAGGAG	PS modified	NA	NA	acactctttccctacacgacgctcttccgatctTGGGTTCCCAGGTCTG	gtgactggagttcagacgtgtgctcttccgatctACCTCTGGACCTGCTG
1B, S1A	ALG1L2	Cas9	Hs.Cas9.ALG1L2.1	ACATCCAGGTCTGCATCCCC	Alt-R Cas9 crRNA:tra	ALG1L2-T	C*A*CCTAGAAAGCGGGGGTA	PS modified	NA	NA	acactctttccctacacgacgctcttccgatctAGTATTACAGCCGCCTCAT	gtgactggagttcagacgtgtgctcttccgatctTCAGAAAAGGTACACCCCG
1B, S1A	ALKAL1	Cas9	Hs.Cas9.ALKAL1.1	TGTCCCCGCACGGAGCCCAC	Alt-R Cas9 crRNA:tra	ALKAL1-T	C*C*GTGACGCGCGCTCCCCT	PS modified	NA	NA	acactctttccctacacgacgctcttccgatctTTGGGCTCCTTATCCGT	gtgactggagttcagacgtgtgctcttccgatctCTTGCGAACATGCGGC
1B, S1A	ALKBH1	Cas9	Hs.Cas9.ALKBH1.1	ACGGAGACCCCGAAGTTTAC	Alt-R Cas9 crRNA:tra	ALKBH1-T	T*A*ATGGTAGCCTACGGTCA	PS modified	NA	NA	acactctttccctacacgacgctcttccgatctCTGTCCCAGTTATAATGGTAGC	gtgactggagttcagacgtgtgctcttccgatctCCTCTTTTGTCACCAATCTTTG
1B, S1A	ALKBH7	Cas9	Hs.Cas9.ALKBH7.1	CCGCTACGAATACGATCACT	Alt-R Cas9 crRNA:tra	ALKBH7-T	T*C*CCTCGCCCCCGGCGCTG	PS modified	NA	NA	acactctttccctacacgacgctcttccgatctAGACGCTGAGCCGAGAA	gtgactggagttcagacgtgtgctcttccgatctCGCCCCATCTGATGCTC
1B, S1A	ALKBH8	Cas9	Hs.Cas9.ALKBH8.1	GCAAATGAGTACGGCTTGTT	Alt-R Cas9 crRNA:tra	ALKBH8-T	G*A*AATGTGGACTGGTGGA	PS modified	NA	NA	acactctttccctacacgacgctcttccgatctCCACTACTTCTTTTCCATTGAGG	gtgactggagttcagacgtgtgctcttccgatctTCCCGGTTTTAGAGAAATGTG
1B, S1A	ALOX5	Cas9	Hs.Cas9.ALOX5.1	GGATTCATACGACGTGACTG	Alt-R Cas9 crRNA:tra	ALOX5-T	A*T*TCTGACCAGCTGGATCT	PS modified	NA	NA	acactctttccctacacgacgctcttccgatctAACACCTCCAGAACAAAGG	gtgactggagttcagacgtgtgctcttccgatctAGGTACCAGTCGTCATTCAG
1B, S1A	ALPK1	Cas9	Hs.Cas9.ALPK1.1	CGTCGAGCCCATACAGGAAC	Alt-R Cas9 crRNA:tra	ALPK1-T	G*T*GCGGCTGCGGCGGCTA	PS modified	NA	NA	acactctttccctacacgacgctcttccgatctGATGTTCCACGCTGTTC	gtgactggagttcagacgtgtgctcttccgatctTTGTGGAGACCTTTGGC
1B, S1A	ALPK2	Cas9	Hs.Cas9.ALPK2.1	GATAACCCTAACCTACACCG	Alt-R Cas9 crRNA:tra	ALPK2-T	C*A*TTCCAGATACTCCAGGT	PS modified	NA	NA	acactctttccctacacgacgctcttccgatctCAGGTTCCTTTGCCAAACT	gtgactggagttcagacgtgtgctcttccgatctAGCTTTCCAGTGAAGACTCT
1B, S1A	ALS2	Cas9	Hs.Cas9.ALS2.1	ACAATGGTGTCGCGTACATG	Alt-R Cas9 crRNA:tra	ALS2-T	T*G*GCTACTGCACACTGGCC	PS modified	NA	NA	acactctttccctacacgacgctcttccgatctCGCCAACTGTAAAATCCTGA	gtgactggagttcagacgtgtgctcttccgatctTAGCCCCATTCTAGAAAATGC
1B, S1A	AMDHD1	Cas9	Hs.Cas9.AMDHD1.1	GAGTGGATATGGCCTCGACC	Alt-R Cas9 crRNA:tra	AMDHD1-T	C*G*CTCAATCACGCGCAGCA	PS modified	NA	NA	acactctttccctacacgacgctcttccgatctGCTCCAGTGCATGATGAG	gtgactggagttcagacgtgtgctcttccgatctGCAGTAGGTAGCCGAGAT
1B, S1A	AMELY	Cas9	Hs.Cas9.AMELY.1	GCACCACCAAATCATCCCCG	Alt-R Cas9 crRNA:tra	AMELY-T	T*G*CAGGGTGTGAGTCAGG	PS modified	NA	NA	acactctttccctacacgacgctcttccgatctTGGTGATGAGACTGCAGG	gtgactggagttcagacgtgtgctcttccgatctAGCCAATGGTAAACCTGCAT
1B, S1A	AMIGO2	Cas9	Hs.Cas9.AMIGO2.1	ACGATTACACCTGTCGCCTG	Alt-R Cas9 crRNA:tra	AMIGO2-T	G*C*AGAAGTACCTGACGCGA	PS modified	NA	NA	acactctttccctacacgacgctcttccgatctAGGAACCATTGATGATGCTGT	gtgactggagttcagacgtgtgctcttccgatctGCTGGTCTTTTGGTATCGTAG
1B, S1A	AMZ2	Cas9	Hs.Cas9.AMZ2.1	ATCTTTACCCAAGAGACTCG	Alt-R Cas9 crRNA:tra	AMZ2-T	C*A*TCTGTCAAAGAGGCCTG	PS modified	NA	NA	acactctttccctacacgacgctcttccgatctCATGCAGGTGAATTACACGA	gtgactggagttcagacgtgtgctcttccgatctAATACCATCTGTCAAAGAGGC
1B, S1A	ANAPC11	Cas9	Hs.Cas9.ANAPC11.1	GATTAAGTGCTGGAACGGCG	Alt-R Cas9 crRNA:tra	ANAPC11-T	C*A*GTTCTCATCGTTGGCCAC	PS modified	NA	NA	acactctttccctacacgacgctcttccgatctGTTGGTGCCTAAACGTTCTA	gtgactggagttcagacgtgtgctcttccgatctCAGATGCCACAGTTCTCAT
1B, S1A	ANGPTL4	Cas9	Hs.Cas9.ANGPTL4.1	ACATTGTGAGCCGGGTCAAC	Alt-R Cas9 crRNA:tra	ANGPTL4-T	T*G*CCCGAAGAAAGAGGCTC	PS modified	NA	NA	acactctttccctacacgacgctcttccgatctGTCCCATCCTAGTTTGGC	gtgactggagttcagacgtgtgctcttccgatctTGGACCTGACAAGGAGAG
1B, S1A	ANGPTL6	Cas9	Hs.Cas9.ANGPTL6.1	ACAAGACGGACCGGAACCAC	Alt-R Cas9 crRNA:tra	ANGPTL6-T	G*T*CACCCCAGGTCCTGCCG	PS modified	NA	NA	acactctttccctacacgacgctcttccgatctTAATGTCGACTTACCCACAGG	gtgactggagttcagacgtgtgctcttccgatctTGCAATTACACCTGACTTTCTCC
1B, S1A	ANKEF1	Cas9	Hs.Cas9.ANKEF1.1	CCCATTCGGTCTTGCACATC	Alt-R Cas9 crRNA:tra	ANKEF1-T	T*A*TGGTCAGCTTTCTCCTTG	PS modified	NA	NA	acactctttccctacacgacgctcttccgatctCTTTGCACTTAGCCTCAGTTT	gtgactggagttcagacgtgtgctcttccgatctTCCATTGACAATTCATGGCC
1B, S1A	ANKIB1	Cas9	Hs.Cas9.ANKIB1.1	GGTATTCTCACGGGATCCCG	Alt-R Cas9 crRNA:tra	ANKIB1-T	T*C*TAATGCAGCATATTCAG	PS modified	NA	NA	acactctttccctacacgacgctcttccgatctCAACACCACAAAGATTTGGC	gtgactggagttcagacgtgtgctcttccgatctAAACTGACCTCTCGTTTGTCT
1B, S1A	ANKRD13D	Cas9	Hs.Cas9.ANKRD13D.1	CTTCGACACAATGCCAACGT	Alt-R Cas9 crRNA:tra	ANKRD13D-T	T*G*CAGTACCTGCCCAGCCC	PS modified	NA	NA	acactctttccctacacgacgctcttccgatctCTCTTCTCTCCTGCCCTTT	gtgactggagttcagacgtgtgctcttccgatctTTGTCCTCTGCAGTACCTG
1B, S1A	ANKRD27	Cas9	Hs.Cas9.ANKRD27.1	TAGACTGGATGCTGCTCGAC	Alt-R Cas9 crRNA:tra	ANKRD27-T	G*T*TTTTTCCAGGTCTTAGTA	PS modified	NA	NA	acactctttccctacacgacgctcttccgatctGGTCTGAAAATGCTCTTCCA	gtgactggagttcagacgtgtgctcttccgatctTTCTGCTGAGGTGGTAAATGG
1B, S1A	ANKRD35	Cas9	Hs.Cas9.ANKRD35.1	CCATTCGAGTCAAGCTTGGT	Alt-R Cas9 crRNA:tra	ANKRD35-T	C*G*TGGCTGCCCTGGCCTCC	PS modified	NA	NA	acactctttccctacacgacgctcttccgatctCTTCAAAAGGGAGCCACAT	gtgactggagttcagacgtgtgctcttccgatctAACCGCCATGATCAGAAG
1B, S1A	ANKRD45	Cas9	Hs.Cas9.ANKRD45.1	CAAAGTTTCCAAACGACCCC	Alt-R Cas9 crRNA:tra	ANKRD45-T	T*T*GTTAGGGTACACACTCT	PS modified	NA	NA	acactctttccctacacgacgctcttccgatctCTGAGAATATCTAGCAGCAACAT	gtgactggagttcagacgtgtgctcttccgatctGTCTGTTTGATCTCACCATCTT
1B, S1A	ANKRD49	Cas9	Hs.Cas9.ANKRD49.1	GGCCACTCACGTGAACACTA	Alt-R Cas9 crRNA:tra	ANKRD49-T	G*C*TGCTCGATGAAGAGGG	PS modified	NA	NA	acactctttccctacacgacgctcttccgatctTTCTTCTCAGCTTACCACAGT	gtgactggagttcagacgtgtgctcttccgatctGCTCCTGAACAATATCTAAGTGT
1B, S1A	ANKRD50	Cas9	Hs.Cas9.ANKRD50.1	TCGGAAGGCATATATCGTCA	Alt-R Cas9 crRNA:tra	ANKRD50-T	T*G*ATCTAAACGATGAAGAA	PS modified	NA	NA	acactctttccctacacgacgctcttccgatctCGCAAAGCTTCTTCTTGATCTAAAC	gtgactggagttcagacgtgtgctcttccgatctGTTGACATTCTCTTTGAAGATATGGT
1B, S1A	ANKRD52	Cas9	Hs.Cas9.ANKRD52.1	CCACGTTGAGGCTGCTCAAC	Alt-R Cas9 crRNA:tra	ANKRD52-T	A*C*CGGGCCACCAAGTGTGC	PS modified	NA	NA	acactctttccctacacgacgctcttccgatctCAAGATGCCCACTATGCA	gtgactggagttcagacgtgtgctcttccgatctTTCAGCAGATGTGAATGCC
1B, S1A	ANKRD61	Cas9	Hs.Cas9.ANKRD61.1	AGAAGACTGCACTACGATCG	Alt-R Cas9 crRNA:tra	ANKRD61-T	A*T*GGGCTGGTTGACAGGG	PS modified	NA	NA	acactctttccctacacgacgctcttccgatctCATCTGCAGCACTTCACT	gtgactggagttcagacgtgtgctcttccgatctCAGAATGGTGATGGGCTG
1B, S1A	ANKZF1	Cas9	Hs.Cas9.ANKZF1.1	GCTATACGGTTCGGGCCAAG	Alt-R Cas9 crRNA:tra	ANKZF1-T	C*A*CCTCGGGCATCCCGAAG	PS modified	NA	NA	acactctttccctacacgacgctcttccgatctTGGTCTCCAGTACTGAGTCT	gtgactggagttcagacgtgtgctcttccgatctCTTCATTGTAGCGCCTCAG
1B, S1A	ANO6	Cas9	Hs.Cas9.ANO6.1	GGACCGGTTTTTCAGATCAT	Alt-R Cas9 crRNA:tra	ANO6-T	G*C*TGAGATAATGCACATCA	PS modified	NA	NA	acactctttccctacacgacgctcttccgatctTGTACGTATGCTGAGATAATGCA	gtgactggagttcagacgtgtgctcttccgatctCTACACTGAGGACTTTGGTAAAC
1B, S1A	ANTXRL	Cas9	Hs.Cas9.ANTXRL.1	GGGGGCCAACGTTTACACCC	Alt-R Cas9 crRNA:tra	ATXRL-T	G*G*AATTACCTGATCCAGAT	PS modified	NA	NA	acactctttccctacacgacgctcttccgatctCACATGCATTTCAGGACACT	gtgactggagttcagacgtgtgctcttccgatctGGTTGGCTCGAAAGTGAC
1B, S1A	ANXA2R	Cas9	Hs.Cas9.ANXA2R.1	AGTGAGTACTCTCCTAGTAC	Alt-R Cas9 crRNA:tra	ANXA2R-T	A*G*AAGATCGTGGGCCGTG	PS modified	NA	NA	acactctttccctacacgacgctcttccgatctAGAGTCCGTTTTGCCAGTA	gtgactggagttcagacgtgtgctcttccgatctAGCCTCCACCTATTGTGA
1B, S1A	AP1S3	Cas9	Hs.Cas9.AP1S3.1	AGAGATTGTGCATCGTTACG	Alt-R Cas9 crRNA:tra	AP1S3-T	A*C*ACTTACATTTCCAAAATA	PS modified	NA	NA	acactctttccctacacgacgctcttccgatctGGGACTGTAGCTAATCCTAAC	gtgactggagttcagacgtgtgctcttccgatctTTCGTGGGAAAAACTGTCTC
1B, S1A	AP3B1	Cas9	Hs.Cas9.AP3B1.1	AAGCGCTTGCACGAATTAGT	Alt-R Cas9 crRNA:tra	AP3B1-T	G*G*TGAATATTTTTGCACTA	PS modified	NA	NA	acactctttccctacacgacgctcttccgatctAGCATCATGATAGGTACAATAATTGG	gtgactggagttcagacgtgtgctcttccgatctTGCTATTGTCATATTACACCCTTTAAG
1B, S1A	AP3M2	Cas9	Hs.Cas9.AP3M2.1	ACAACCGTTCGAAGGATGGT	Alt-R Cas9 crRNA:tra	AP3M2-T	C*G*AGTCGAACATTCTTAAA	PS modified	NA	NA	acactctttccctacacgacgctcttccgatctTTTCCATTGGCTACCGAGT	gtgactggagttcagacgtgtgctcttccgatctCTGTAGGATGGCCACTATCT
1B, S1A	AP3S2	Cas9	Hs.Cas9.AP3S2.1	TTTCCATCTAGTCCTCAAGC	Alt-R Cas9 crRNA:tra	AP3S2-T	C*T*TCCACCCTCCAAGAAGT	PS modified	NA	NA	acactctttccctacacgacgctcttccgatctCTGACAGTTTACCTTCCACC	gtgactggagttcagacgtgtgctcttccgatctGGATGTAGTTTTTCAGGCTTG
1B, S1A	APAF1	Cas9	Hs.Cas9.APAF1.1	TCTTTGCACACGGTTGGATC	Alt-R Cas9 crRNA:tra	APAF1-T	A*T*ATTAAGTGGAAGCCTCT	PS modified	NA	NA	acactctttccctacacgacgctcttccgatctGGCCTTAAGTTCATTATTCTTTCC	gtgactggagttcagacgtgtgctcttccgatctTAGCCTCTTCAATATTAAGTGGAA
1B, S1A	APBB2	Cas9	Hs.Cas9.APBB2.1	TTGGGACAACGTTGTCCAGC	Alt-R Cas9 crRNA:tra	APBB2-T	C*G*GATCCAGAACCTGGCAC	PS modified	NA	NA	acactctttccctacacgacgctcttccgatctAGGCTCACGTTCCTCTCT	gtgactggagttcagacgtgtgctcttccgatctGGCCAAAACCGACTGTG
1B, S1A	APOBEC2	Cas9	Hs.Cas9.APOBEC2.1	ACCAGGTGACATTGTACCGC	Alt-R Cas9 crRNA:tra	APOBEC2-T	T*C*TTCAACACCATCCTGCCA	PS modified	NA	NA	acactctttccctacacgacgctcttccgatctTTTCTTCAACACCATCCTGC	gtgactggagttcagacgtgtgctcttccgatctCAGGTTCTTGGTCTTGCTAAG
1B, S1A	APOBEC3A	Cas9	Hs.Cas9.APOBEC3A.1	CGGTCAAGATGGACCAGCAC	Alt-R Cas9 crRNA:tra	APOBEC3A-T	T*G*GCTGGGTCGGTCACCTG	PS modified	NA	NA	acactctttccctacacgacgctcttccgatctGGCATAAGACCTACCTGTG	gtgactggagttcagacgtgtgctcttccgatctTGACAAACACTGCAGGAAA
1B, S1A	APOC1	Cas9	Hs.Cas9.APOC1.1	TCCAAGGCACTGGAGACGTC	Alt-R Cas9 crRNA:tra	APOC1-T	C*T*TCCTGGCAGGCCCAGCC	PS modified	NA	NA	acactctttccctacacgacgctcttccgatctATTTTGAACCCCTGCCCAT	gtgactggagttcagacgtgtgctcttccgatctAGCCTTGTCCTCCAGTGT
1B, S1A	APOC3	Cas9	Hs.Cas9.APOC3.1	GCAACAACAAGGAGTACCCG	Alt-R Cas9 crRNA:tra	APOC3-T	G*A*CACTTCCTTGCAGGAAC	PS modified	NA	NA	acactctttccctacacgacgctcttccgatctACAGGACACTTCCTTGCA	gtgactggagttcagacgtgtgctcttccgatctCCACCAAGTGCTTACGG
1B, S1A	APOC4	Cas9	Hs.Cas9.APOC4.1	GAACCATTGCCACCCGTCTC	Alt-R Cas9 crRNA:tra	APOC4-T	A*T*GAAGGAGCTGCTGGAG	PS modified	NA	NA	acactctttccctacacgacgctcttccgatctTAAAGATGAGTCGCTGGAG	gtgactggagttcagacgtgtgctcttccgatctCTTTCCTCCTCCCTGAGA
1B, S1A	APOL2	Cas9	Hs.Cas9.APOL2.1	ACGAGCCCAAGCCCGCAACT	Alt-R Cas9 crRNA:tra	APOL2-T	T*T*CATCACCTTTGCTACATT	PS modified	NA	NA	acactctttccctacacgacgctcttccgatctACTCCTTCATCACCTTTGCTA	gtgactggagttcagacgtgtgctcttccgatctGATTACCTGCAGTGTGGTAGAA
1B, S1A	APOPT1	Cas9	Hs.Cas9.APOPT1.1	TGTAAAAGTGAACAGGTCGA	Alt-R Cas9 crRNA:tra	APOPT1-T	A*T*GATTGGATAGGACCCCC	PS modified	NA	NA	acactctttccctacacgacgctcttccgatctAAAGGTCTCAAGATTCTGCC	gtgactggagttcagacgtgtgctcttccgatctGTTTGCCCAGAACTGTTGATT
1B, S1A	APP	Cas9	Hs.Cas9.APP.1	GCGGAATTGACAAGTTCCGA	Alt-R Cas9 crRNA:tra	APP-T	T*T*TCTTCAGCCAGTGGGCA	PS modified	NA	NA	acactctttccctacacgacgctcttccgatctCACATTGTCACTTTCTTCAGC	gtgactggagttcagacgtgtgctcttccgatctCCTTACACTTTGTAGACATGCA
1B, S1A	AR	Cas9	Hs.Cas9.AR.1	CTGGGACGCAACCTCTCTCG	Alt-R Cas9 crRNA:tra	AR-T	A*A*CCTTCACAGCCGCAGTC	PS modified	NA	NA	acactctttccctacacgacgctcttccgatctAACAGCAACCTTCACAGC	gtgactggagttcagacgtgtgctcttccgatctAGCTGAGTCATCCTCGTC
1B, S1A	AREL1	Cas9	Hs.Cas9.AREL1.1	GGACTATTTATGACTACGTG	Alt-R Cas9 crRNA:tra	AREL1-T	A*G*ACTTTGCAAGACCGGGC	PS modified	NA	NA	acactctttccctacacgacgctcttccgatctTCCAATCCCAGGAGACTTTG	gtgactggagttcagacgtgtgctcttccgatctACGTGTAGTCAGCTTCCTC
1B, S1A	ARF3	Cas9	Hs.Cas9.ARF3.1	AGGACAAGATTCGACCCCTC	Alt-R Cas9 crRNA:tra	ARF3-T	T*G*AGCATACCTTGGGTGTT	PS modified	NA	NA	acactctttccctacacgacgctcttccgatctACTCTCTGCTCATACCCAA	gtgactggagttcagacgtgtgctcttccgatctCAAAGGGTTCAATGTGGAGA
1B, S1A	ARF5	Cas9	Hs.Cas9.ARF5.1	CCCCCAACTTCAGTTTGTAC	Alt-R Cas9 crRNA:tra	ARF5-T	C*A*GTTGGCTTGGATGCGGC	PS modified	NA	NA	acactctttccctacacgacgctcttccgatctATCCCTGTGCCCCTTTC	gtgactggagttcagacgtgtgctcttccgatctCGGGCTCACCTATGGTT
1B, S1A	ARHGAP22	Cas9	Hs.Cas9.ARHGAP22.1	GTCCACCACGAGCGGAAGTA	Alt-R Cas9 crRNA:tra	ARHGAP22-T	A*C*ACTGCTCCACCAGCAGG	PS modified	NA	NA	acactctttccctacacgacgctcttccgatctATGAAGTCCACACACTGCTC	gtgactggagttcagacgtgtgctcttccgatctAGGGTGTGACTAGCTCCT

1B, S1A	ARHGAP23	Cas9	Hs.Cas9.ARHGAP23.1	CATCGTGTACCCACCCGAGT	Alt-R Cas9 crRNA:tra	ARHGAP23-T	C*G*AGCCGGGCATACCTTCA	PS modified	NA	NA	acactctttccctacacgacgctcttccgatctTGCTGCTGTACAAAAGTCC	gtgactggagttcagacgtgtgctcttccgatctCCCTCATGAAAGCTTCCC
1B, S1A	ARHGAP24	Cas9	Hs.Cas9.ARHGAP24.1	TCAAGACTTGGCATACTCGC	Alt-R Cas9 crRNA:tra	ARHGAP24-T	T*G*AAATAATAGAGCTGATC	PS modified	NA	NA	acactctttccctacacgacgctcttccgatctGTTTGCTAACTAGGAAAGTCCAT	gtgactggagttcagacgtgtgctcttccgatctGGCCCTTAATGCTTTACATTTTCT
1B, S1A	ARHGAP36	Cas9	Hs.Cas9.ARHGAP36.1	AAGGTGTTCGGACGGTCAAT	Alt-R Cas9 crRNA:tra	ARHGAP36-T	T*G*TATCTTTTCTTTTTTCTCT	PS modified	NA	NA	acactctttccctacacgacgctcttccgatctCAGATCAGGGCATTGGGAT	gtgactggagttcagacgtgtgctcttccgatctCCTCTGCTGTCCTCTCAA
1B, S1A	ARHGAP6	Cas9	Hs.Cas9.ARHGAP6.1	TCGGATTTCCACACGACGTA	Alt-R Cas9 crRNA:tra	ARHGAP6-T	G*A*AGTTCCAGTCCCCACCC	PS modified	NA	NA	acactctttccctacacgacgctcttccgatctCTTAGTGGGTGCCTTGCT	gtgactggagttcagacgtgtgctcttccgatctAAGTTCCAGTCCCCACC
1B, S1A	ARHGEF33	Cas9	Hs.Cas9.ARHGEF33.1	GATGGCTTGGATCATCGACA	Alt-R Cas9 crRNA:tra	ARHGEF33-T	G*C*AATTCTTATTTTTTAGGA	PS modified	NA	NA	acactctttccctacacgacgctcttccgatctCCCCATGTACCACGTTAAAA	gtgactggagttcagacgtgtgctcttccgatctCTGTTGAAGCTGCTCGATTTT
1B, S1A	ARID1B	Cas9	Hs.Cas9.ARID1B.1	TGCCAATTGGATACCGCTGT	Alt-R Cas9 crRNA:tra	ARID1B-T	G*T*CCGTACCCAGGAGGTTC	PS modified	NA	NA	acactctttccctacacgacgctcttccgatctGCAGTAACCCTCATTCTCA	gtgactggagttcagacgtgtgctcttccgatctGAGGGTACTGCATTCCG
1B, S1A	ARIH2	Cas9	Hs.Cas9.ARIH2.1	GTTCTCGTCAAGGACGGCGT	Alt-R Cas9 crRNA:tra	ARIH2-T	G*C*TTTTTATAAGTTTTGTGC	PS modified	NA	NA	acactctttccctacacgacgctcttccgatctAAGGAAAACCTACTCTCTCTGG	gtgactggagttcagacgtgtgctcttccgatctGAGTCAAAGATAAACACTTCATGC
1B, S1A	ARL1	Cas9	Hs.Cas9.ARL1.1	CATGGCAACTAACTCTGATT	Alt-R Cas9 crRNA:tra	ARL1-T	G*T*AGACAGTTGTGACCGAC	PS modified	NA	NA	acactctttccctacacgacgctcttccgatctAATGACTGCCCCACATTTTA	gtgactggagttcagacgtgtgctcttccgatctCTCTAGGCCATACTGGAGAT
1B, S1A	ARL15	Cas9	Hs.Cas9.ARL15.1	TGGTCGACACGACGTTATCG	Alt-R Cas9 crRNA:tra	ARL15-T	A*A*ACCAGTCTGTTGTCCAA	PS modified	NA	NA	acactctttccctacacgacgctcttccgatctTGCACAGGAAACTAGGACAT	gtgactggagttcagacgtgtgctcttccgatctGTTTGCATAGGCCTCACAG
1B, S1A	ARL16	Cas9	Hs.Cas9.ARL16.1	TGCCACGATGTCAGTAAGAT	Alt-R Cas9 crRNA:tra	ARL16-T	C*T*TTTTGCATCGTGGTTTTT	PS modified	NA	NA	acactctttccctacacgacgctcttccgatctGCCCATATAGGATTACAACCC	gtgactggagttcagacgtgtgctcttccgatctCTCTTCAGGAGTTCACAACG
1B, S1A	ARL2	Cas9	Hs.Cas9.ARL2.1	AACTACTTTGAGAGCACCGA	Alt-R Cas9 crRNA:tra	ARL2-T	C*T*GGCGGTCTGCGCTGTCC	PS modified	NA	NA	acactctttccctacacgacgctcttccgatctCTCCTTGCCCAGATTCAA	gtgactggagttcagacgtgtgctcttccgatctCATCTGCTGAGAAGGCAG
1B, S1A	ARL6	Cas9	Hs.Cas9.ARL6.1	TGCCACTATTATCTAGCCCA	Alt-R Cas9 crRNA:tra	ARL6-T	T*T*GGCCTGAAGAAGAAGG	PS modified	NA	NA	acactctttccctacacgacgctcttccgatctCCAATATTTTCCATAACTTAAGGTGC	gtgactggagttcagacgtgtgctcttccgatctGCATCTAACAAAGATACTTACATTTGAA
1B, S1A	ARL6IP5	Cas9	Hs.Cas9.ARL6IP5.1	CTACCCCACGACGTTCGTTA	Alt-R Cas9 crRNA:tra	ARL6IP5-T	A*T*GGAGATAAGGAAATAG	PS modified	NA	NA	acactctttccctacacgacgctcttccgatctGGCAGCCCACAATAAAGAC	gtgactggagttcagacgtgtgctcttccgatctCACAAAGACCATGACTCCTC
1B, S1A	ARMC10	Cas9	Hs.Cas9.ARMC10.1	TTACCTGCTGGAGTCAACGG	Alt-R Cas9 crRNA:tra	ARMC10-T	A*A*AGTAATCAAAGCTCTTT	PS modified	NA	NA	acactctttccctacacgacgctcttccgatctTTCTTATGCAGAAGACTTAACTGATG	gtgactggagttcagacgtgtgctcttccgatctTGCATTGTTACCCAAAGTAATCAAAG
1B, S1A	ARMC3	Cas9	Hs.Cas9.ARMC3.1	ACTACTGAGTAGCCCTGACC	Alt-R Cas9 crRNA:tra	ARMC3-T	T*T*GTAAATGCATTCCATAG	PS modified	NA	NA	acactctttccctacacgacgctcttccgatctCAAACATGTCTGCAGAGTACAC	gtgactggagttcagacgtgtgctcttccgatctCTTACCTGCACCAAGTTGTAAAT
1B, S1A	ARMC6	Cas9	Hs.Cas9.ARMC6.1	CACACGGATGTCGTCATCGA	Alt-R Cas9 crRNA:tra	ARMC6-T	G*T*CAGGGAAGCCTGCTGG	PS modified	NA	NA	acactctttccctacacgacgctcttccgatctCGCTTGCCTGAAACATGAA	gtgactggagttcagacgtgtgctcttccgatctCCTTTGTTCTCCTGCACAAT
1B, S1A	ARMCX1	Cas9	Hs.Cas9.ARMCX1.1	TTGAACTTGCCTCTCCGGAC	Alt-R Cas9 crRNA:tra	ARMCX1-T	A*A*GTAAGAGCACCAGGGC	PS modified	NA	NA	acactctttccctacacgacgctcttccgatctGGGCAAGTGGAAAATCCAAG	gtgactggagttcagacgtgtgctcttccgatctTTGTTACCCAGAGTGACCAA
1B, S1A	ARNTL2	Cas9	Hs.Cas9.ARNTL2.1	CTCACGCGGCTGGAAACCAC	Alt-R Cas9 crRNA:tra	ARTL2-T	T*A*AAGTGTTGAGAGAGGA	PS modified	NA	NA	acactctttccctacacgacgctcttccgatctGCCCATAGGTAAAGTGTTGA	gtgactggagttcagacgtgtgctcttccgatctGTGGAAACTCTGTCATGTGT
1B, S1A	ARR3	Cas9	Hs.Cas9.ARR3.1	AGTACAATTTGCACCACCGG	Alt-R Cas9 crRNA:tra	ARR3-T	C*G*GCGGATGGTCTGGGCT	PS modified	NA	NA	acactctttccctacacgacgctcttccgatctCTGGCTAATCTCTTGGTCTCT	gtgactggagttcagacgtgtgctcttccgatctATCAGTGAAGAAAGGGCATCA
1B, S1A	ASAP3	Cas9	Hs.Cas9.ASAP3.1	CTAAGGATTCCACGGCCTCT	Alt-R Cas9 crRNA:tra	ASAP3-T	C*C*TCCACAGGCCATGTGGA	PS modified	NA	NA	acactctttccctacacgacgctcttccgatctTGAACACGGCCAAGTTTAG	gtgactggagttcagacgtgtgctcttccgatctAGCTAACTCCAAGCTCCC
1B, S1A	ASB7	Cas9	Hs.Cas9.ASB7.1	TGATCACGGCGTATCGCAAC	Alt-R Cas9 crRNA:tra	ASB7-T	A*C*CACAATGCCAACATCGA	PS modified	NA	NA	acactctttccctacacgacgctcttccgatctTCGCCATTATCCGAGAGAG	gtgactggagttcagacgtgtgctcttccgatctCCTCTCTGAAGGAACATTCG
1B, S1A	ASCC3	Cas9	Hs.Cas9.ASCC3.1	ATGGCTTTACCTCGTCTCAC	Alt-R Cas9 crRNA:tra	ASCC3-T	T*T*GCTTGGTGACATTTGAA	PS modified	NA	NA	acactctttccctacacgacgctcttccgatctCAAATCTACCTTTAAGTCAGCCA	gtgactggagttcagacgtgtgctcttccgatctGTTTCAGGTTTCTTGCTGTATG
1B, S1A	ASCL5	Cas9	Hs.Cas9.ASCL5.1	GCGAGTCAAGTGCGTCAACG	Alt-R Cas9 crRNA:tra	ASCL5-T	G*C*GCCGGGGAGGTGGCCG	PS modified	NA	NA	acactctttccctacacgacgctcttccgatctTCTCCACCTTGCTGAGTC	gtgactggagttcagacgtgtgctcttccgatctAATACCCCTTCGAGCCAG
1B, S1A	ASIC5	Cas9	Hs.Cas9.ASIC5.1	AGAACCGGAGCAAAATTCGC	Alt-R Cas9 crRNA:tra	ASIC5-T	A*G*ACTGAGCCCAGAACCA	PS modified	NA	NA	acactctttccctacacgacgctcttccgatctTAGATCTGCCATGTCACAAGT	gtgactggagttcagacgtgtgctcttccgatctAGCGAAAGAAGTTTGACCATGA
1B, S1A	ASL	Cas9	Hs.Cas9.ASL.1	TGCCTACGACCGGCACCTTT	Alt-R Cas9 crRNA:tra	ASL-T	C*T*GCTGTAGGCTTTGCTGC	PS modified	NA	NA	acactctttccctacacgacgctcttccgatctAATTGTTCTTGCTCTCCTGG	gtgactggagttcagacgtgtgctcttccgatctTCTAGGCCATGGAGTATCTG
1B, S1A	ASPHD2	Cas9	Hs.Cas9.ASPHD2.1	CGTGTGGTACTGTTATCACG	Alt-R Cas9 crRNA:tra	ASPHD2-T	T*T*GACGGAGACGTAGGGC	PS modified	NA	NA	acactctttccctacacgacgctcttccgatctGCTGTCATCACTGTGG	gtgactggagttcagacgtgtgctcttccgatctCGTAGCCATTCTGCAG
1B, S1A	ATF5	Cas9	Hs.Cas9.ATF5.1	TGGGATGGCTCGTAGACTAT	Alt-R Cas9 crRNA:tra	ATF5-T	A*G*GGAGCCAGGGGGGCAC	PS modified	NA	NA	acactctttccctacacgacgctcttccgatctTGTGCTACAGCCATGTCA	gtgactggagttcagacgtgtgctcttccgatctAAGGACCTCATAGGGAGC
1B, S1A	ATG4D	Cas9	Hs.Cas9.ATG4D.1	CGGCTTTTAACCACCCAACC	Alt-R Cas9 crRNA:tra	ATG4D-T	G*C*TTCATCTCGCTGGGCGC	PS modified	NA	NA	acactctttccctacacgacgctcttccgatctGCTGTCATGCAAGTGCTT	gtgactggagttcagacgtgtgctcttccgatctCACAGAGGTGGATGCTG
1B, S1A	ATP10D	Cas9	Hs.Cas9.ATP10D.1	GAGACCCACGCGTTCTTTGT	Alt-R Cas9 crRNA:tra	ATP10D-T	G*A*ATTTATATGCTGTCTTTC	PS modified	NA	NA	acactctttccctacacgacgctcttccgatctCAAGTCAAAAACATTCAAGGGC	gtgactggagttcagacgtgtgctcttccgatctGTGTTTCTAATGGTGCATCCT
1B, S1A	ATP1B4	Cas9	Hs.Cas9.ATP1B4.1	GACGGTGGTGCCCAAATCGG	Alt-R Cas9 crRNA:tra	ATP1B4-T	T*C*CTCCTCTTCTTTCTCCTCC	PS modified	NA	NA	acactctttccctacacgacgctcttccgatctCACTGTTCCAGGATGATCC	gtgactggagttcagacgtgtgctcttccgatctCTCTGGATCCCACAGGTATT
1B, S1A	ATP2A2	Cas9	Hs.Cas9.ATP2A2.1	TTCGGCGTCAACGAGAGTAC	Alt-R Cas9 crRNA:tra	ATP2A2-T	C*T*CCTTAAGCTTCTTGACCT	PS modified	NA	NA	acactctttccctacacgacgctcttccgatctAAGCCATGGAGAACGCG	gtgactggagttcagacgtgtgctcttccgatctCACCTACCGTTGGAGCC
1B, S1A	ATP5E	Cas9	Hs.Cas9.ATP5E.1	TTGCACAGATCTGGGAGTAT	Alt-R Cas9 crRNA:tra	ATP5E-T	C*C*ATATACCTTCTCTCATTT	PS modified	NA	NA	acactctttccctacacgacgctcttccgatctCCAGAAGTCTTCTCAGCATTT	gtgactggagttcagacgtgtgctcttccgatctCACAGTAACAGCTGTCTGG
1B, S1A	ATP5J2	Cas9	Hs.Cas9.ATP5J2.1	GCCAAGCTGGATCTTGATGC	Alt-R Cas9 crRNA:tra	ATP5J2-T	T*G*AAACGCTCCGAAAATGC	PS modified	NA	NA	acactctttccctacacgacgctcttccgatctCTGACCTCTTTGAAACGCTC	gtgactggagttcagacgtgtgctcttccgatctCCGTTTCAGTACCAGTGAAG
1B, S1A	ATP5L2	Cas9	Hs.Cas9.ATP5L2.1	GTACTACACCACGGTTGAGC	Alt-R Cas9 crRNA:tra	ATP5L2-T	A*T*AGCTCTAGGGATCTCAG	PS modified	NA	NA	acactctttccctacacgacgctcttccgatctCTACCAGTCTGAGCACTACT	gtgactggagttcagacgtgtgctcttccgatctTGCTGTGACTTACTTGAAGC
1B, S1A	ATP6AP2	Cas9	Hs.Cas9.ATP6AP2.1	GAAACAGGCGATTACGGAGC	Alt-R Cas9 crRNA:tra	ATP6AP2-T	A*C*TCAGTGTTTGAAGACCT	PS modified	NA	NA	acactctttccctacacgacgctcttccgatctTCTTCTTACAGAGAGTGTATATGGTA	gtgactggagttcagacgtgtgctcttccgatctCTTACTTCATTGTTCCTACTCAGAG
1B, S1A	ATP6V0A2	Cas9	Hs.Cas9.ATP6V0A2.1	TGTTTGGATAGGGGTACACG	Alt-R Cas9 crRNA:tra	ATP6V0A2-T	A*C*TGTGTTCAACTCTTGTCT	PS modified	NA	NA	acactctttccctacacgacgctcttccgatctAGTTCTAGTGCTGACAGATGT	gtgactggagttcagacgtgtgctcttccgatctCACTTCCAGCTTACTCACAGT
1B, S1A	ATP6V1G1	Cas9	Hs.Cas9.ATP6V1G1.1	TTCAGTGCTGCAACTGCCAC	Alt-R Cas9 crRNA:tra	ATP6V1G1-T	C*G*TGACATCACTCCCCATCT	PS modified	NA	NA	acactctttccctacacgacgctcttccgatctGTTCTGATAATCCCTCCGTGA	gtgactggagttcagacgtgtgctcttccgatctGGTTGTCCAAGACTTCATCC
1B, S1A	ATP8B3	Cas9	Hs.Cas9.ATP8B3.1	GCCAACAACCGTGCCTACAA	Alt-R Cas9 crRNA:tra	ATP8B3-T	T*T*GCCAGCACAGGATCACC	PS modified	NA	NA	acactctttccctacacgacgctcttccgatctCCGCCCACCTTGTATTT	gtgactggagttcagacgtgtgctcttccgatctGGTGTTTGTCCTGGGC
1B, S1A	ATXN7	Cas9	Hs.Cas9.ATXN7.1	TAATCAGGTTGTCAAACCGC	Alt-R Cas9 crRNA:tra	ATXN7-T	A*T*GGTTAAGCACTTACCAT	PS modified	NA	NA	acactctttccctacacgacgctcttccgatctGTCCAGCCCATGATGATTT	gtgactggagttcagacgtgtgctcttccgatctACATTATGTCCCATGCATGC
1B, S1A	AVEN	Cas9	Hs.Cas9.AVEN.1	GTTCGGCAGCAACGTTGAGT	Alt-R Cas9 crRNA:tra	AVEN-T	T*G*GTTCGAGCCCTTCAAGA	PS modified	NA	NA	acactctttccctacacgacgctcttccgatctTTTCTCCTCCTGCCCTAAT	gtgactggagttcagacgtgtgctcttccgatctGTGAGTTATTGGTTCGAGCC
1B, S1A	AXL	Cas9	Hs.Cas9.AXL.1	TGCGAAGCCCATAACGCCAA	Alt-R Cas9 crRNA:tra	AXL-T	A*C*CTGTGATGGTGGCTGTG	PS modified	NA	NA	acactctttccctacacgacgctcttccgatctACCCTTCCCTCATATGACT	gtgactggagttcagacgtgtgctcttccgatctCTCCCAACTCTGTCACCT
1B, S1A	B3GNT4	Cas9	Hs.Cas9.B3GNT4.1	ACTCTAACACGTTGGGGACG	Alt-R Cas9 crRNA:tra	B3GT4-T	A*T*TTCATGCTAAAGGGAGA	PS modified	NA	NA	acactctttccctacacgacgctcttccgatctAGGCCCATTTCATGCTAAA	gtgactggagttcagacgtgtgctcttccgatctTGGCTCTGGACATGACATAT
1B, S1A	B4GALNT1	Cas9	Hs.Cas9.B4GALNT1.1	CCCACGGCGCAAGAGGTAGC	Alt-R Cas9 crRNA:tra	B4GALT1-T	T*C*CTGTACGCGAGCACCCG	PS modified	NA	NA	acactctttccctacacgacgctcttccgatctAGGAGCAAGATCTGGCAG	gtgactggagttcagacgtgtgctcttccgatctGTGCGCTCTGGTCCTT
1B, S1A	BAHD1	Cas9	Hs.Cas9.BAHD1.1	ACCAATGGGCGCTTACGAAG	Alt-R Cas9 crRNA:tra	BAHD1-T	C*C*CAGGTCACCTCACAGGC	PS modified	NA	NA	acactctttccctacacgacgctcttccgatctGAGAAAGTCCCTTCCCATG	gtgactggagttcagacgtgtgctcttccgatctATTCTCCAGGCGAGTCAG
1B, S1A	BAK1	Cas9	Hs.Cas9.BAK1.1	GGAACTCTGAGTCATAGCGT	Alt-R Cas9 crRNA:tra	BAK1-T	G*A*CGGCAGCTCGCCATCAT	PS modified	NA	NA	acactctttccctacacgacgctcttccgatctGTGGCAATCTTGGTGAAGTA	gtgactggagttcagacgtgtgctcttccgatctTGGTTACTGGCTCACCTG
1B, S1A	BANF1	Cas9	Hs.Cas9.BANF1.1	CCAAAAGCACCGAGACTTCG	Alt-R Cas9 crRNA:tra	BANF1-T	A*G*GCTCCCCACTGGCTTCTC	PS modified	NA	NA	acactctttccctacacgacgctcttccgatctTCTTACTATGAGATTGCTCGTGG	gtgactggagttcagacgtgtgctcttccgatctACACCTTGTCAAAACCCCTTTC
1B, S1A	BATF2	Cas9	Hs.Cas9.BATF2.1	GGTTTGGGGGACACACGGGT	Alt-R Cas9 crRNA:tra	BATF2-T	G*G*TGCCATTTCTCACCTGG	PS modified	NA	NA	acactctttccctacacgacgctcttccgatctGCCCTTCATAACCACCTAC	gtgactggagttcagacgtgtgctcttccgatctATGACATGGTTGCTGAATGG
1B, S1A	BBOX1	Cas9	Hs.Cas9.BBOX1.1	ACCGGAGCATCTGACAAACC	Alt-R Cas9 crRNA:tra	BBOX1-T	G*A*AACCCATCCTTTTCCCAA	PS modified	NA	NA	acactctttccctacacgacgctcttccgatctCAGCTACCCACTTTGGATTTT	gtgactggagttcagacgtgtgctcttccgatctGTCCTCAGTTGGGAACTATTT
1B, S1A	BBS2	Cas9	Hs.Cas9.BBS2.1	GCCACCAATAATCGCAAGAG	Alt-R Cas9 crRNA:tra	BBS2-T	G*C*AATTGTGCTGGGGACAT	PS modified	NA	NA	acactctttccctacacgacgctcttccgatctATACCGTCCAAAAGAGATCACTT	gtgactggagttcagacgtgtgctcttccgatctCTTTTCTTAGGTAGCAGATGGG
1B, S1A	BCAR3	Cas9	Hs.Cas9.BCAR3.1	GGTGAATCACCAGTTCCCCC	Alt-R Cas9 crRNA:tra	BCAR3-T	G*G*GGACCTGCTGCTCAGAA	PS modified	NA	NA	acactctttccctacacgacgctcttccgatctTGTATAGACACATCTTGATAGGCAT	gtgactggagttcagacgtgtgctcttccgatctGAATTATGGCTGCAGGAAAATTTG
1B, S1A	BCCIP	Cas9	Hs.Cas9.BCCIP.1	CTGCGGAACCCCACTTTCCA	Alt-R Cas9 crRNA:tra	BCCIP-T	A*G*CGGCAACATGGCGTCCA	PS modified	NA	NA	acactctttccctacacgacgctcttccgatctGCTACTATGGGCTGGTC	gtgactggagttcagacgtgtgctcttccgatctTTTCCTCTTCCTCGTCG
1B, S1A	BCHE	Cas9	Hs.Cas9.BCHE.1	TACCGCCCAAGGAGCATTAA	Alt-R Cas9 crRNA:tra	BCHE-T	T*C*ATTGTTCACCAGAGCCAT	PS modified	NA	NA	acactctttccctacacgacgctcttccgatctAGCTAAGTTCAACGTTCTGTTC	gtgactggagttcagacgtgtgctcttccgatctTAGCCTGCATTTGCTTTCTC
1B, S1A	BCO2	Cas9	Hs.Cas9.BCO2.1	GTCAACTATGTGCGGTACAA	Alt-R Cas9 crRNA:tra	BCO2-T	C*A*TAAAGTTGGTCTCAGTG	PS modified	NA	NA	acactctttccctacacgacgctcttccgatctAAATAACTCTAGGGTTTGGTTTCA	gtgactggagttcagacgtgtgctcttccgatctCTTTATTCATAAAGTTGGTCTCAGT
1B, S1A	BECN2	Cas9	Hs.Cas9.BECN2.1	TCACCGCCACGTTTGAGATC	Alt-R Cas9 crRNA:tra	BECN2-T	T*G*AAGTTATTGATGACGCC	PS modified	NA	NA	acactctttccctacacgacgctcttccgatctAGAACCAGCTGCAGTATG	gtgactggagttcagacgtgtgctcttccgatctGGCCCAACCTGAAGTTATT
1B, S1A	BEND7	Cas9	Hs.Cas9.BEND7.1	GAATGGCCTGCAACGTTGAC	Alt-R Cas9 crRNA:tra	BEND7-T	C*A*TCAGCTGGATCAAACTG	PS modified	NA	NA	acactctttccctacacgacgctcttccgatctGATCCTTGTACCTGCTTGAATTT	gtgactggagttcagacgtgtgctcttccgatctGTGAATTCATCAGCTGGATCAAA
1B, S1A	BEX1	Cas9	Hs.Cas9.BEX1.1	GTGTGCCTAGAGGAAATCGT	Alt-R Cas9 crRNA:tra	BEX1-T	T*A*TACTGCAGGATGGGCTG	PS modified	NA	NA	acactctttccctacacgacgctcttccgatctTCCAAGCCTATGCATCATATC	gtgactggagttcagacgtgtgctcttccgatctGCAGTAAACAGTCTCAGCAT
1B, S1A	BEX5	Cas9	Hs.Cas9.BEX5.1	ATGTTATCGACAAGCCTATT	Alt-R Cas9 crRNA:tra	BEX5-T	G*G*CTCCACCTGCCCCGGGT	PS modified	NA	NA	acactctttccctacacgacgctcttccgatctCCTTAGCTCTCTCATCTCCT	gtgactggagttcagacgtgtgctcttccgatctCAAAGTTGTGGAGAAGGCC
1B, S1A	BICDL1	Cas9	Hs.Cas9.BICDL1.1	CGACCCCGGGGAACACCCTC	Alt-R Cas9 crRNA:tra	BICDL1-T	G*G*TCCGGCCCCCTCGGCCA	PS modified	NA	NA	acactctttccctacacgacgctcttccgatctGGAGCTAGAACTGGCGTTA	gtgactggagttcagacgtgtgctcttccgatctGCTCATGTCCTGGTTCCT
1B, S1A	BICDL2	Cas9	Hs.Cas9.BICDL2.1	CAACGAGGCAGCCGACACGT	Alt-R Cas9 crRNA:tra	BICDL2-T	T*C*CGAGCTGGAGGAGGAG	PS modified	NA	NA	acactctttccctacacgacgctcttccgatctTGCAACCCTCTCGATGG	gtgactggagttcagacgtgtgctcttccgatctTCCGAGCTGGAGGAGG
1B, S1A	BLOC1S1	Cas9	Hs.Cas9.BLOC1S1.1	GATCCACCAAAGCTTCTGTC	Alt-R Cas9 crRNA:tra	BLOC1S1-T	A*G*AGGAGGCGAGAGGCTA	PS modified	NA	NA	acactctttccctacacgacgctcttccgatctCAACTAGCAGAATAGTAATGGATGG	gtgactggagttcagacgtgtgctcttccgatctGAGGAAACTGATGTTGATAAGAGGT
1B, S1A	BLOC1S6	Cas9	Hs.Cas9.BLOC1S6.1	GTGTGTCTAACAATACAACT	Alt-R Cas9 crRNA:tra	BLOC1S6-T	T*T*GTCTTGGCTGTGTGTTTT	PS modified	NA	NA	acactctttccctacacgacgctcttccgatctCACTTTAAATATGTAGAGTTTGTCTTGG	gtgactggagttcagacgtgtgctcttccgatctCCAAAGCATTAATATCCAACATAGAATGA
1B, S1A	BLVRA	Cas9	Hs.Cas9.BLVRA.1	ACACGAAGCCAATCAGGTTC	Alt-R Cas9 crRNA:tra	BLVRA-T	G*G*GACTTGCGGAATCCACA	PS modified	NA	NA	acactctttccctacacgacgctcttccgatctCCTACAGTGTTTTCAGACTCCA	gtgactggagttcagacgtgtgctcttccgatctCTGGGCTTTCCATGAATTATGAA
1B, S1A	BMF	Cas9	Hs.Cas9.BMF.1	AGAGAGCAAGCTCCCGGGTT	Alt-R Cas9 crRNA:tra	BMF-T	G*A*TGTGTTCCAACCAGAGC	PS modified	NA	NA	acactctttccctacacgacgctcttccgatctAGAGTCTGGGTAGCTTTGT	gtgactggagttcagacgtgtgctcttccgatctAGGATGATGTGTTCCAACC
1B, S1A	BMP6	Cas9	Hs.Cas9.BMP6.1	CTTCCTCAACGACGCGGACA	Alt-R Cas9 crRNA:tra	BMP6-T	C*C*CAAATCCTTACCCAGGT	PS modified	NA	NA	acactctttccctacacgacgctcttccgatctTCAACCGCAAGAGCCTT	gtgactggagttcagacgtgtgctcttccgatctCGTTACCCCAAATCCTTACC
1B, S1A	BMT2	Cas9	Hs.Cas9.BMT2.1	TGGTGAAGAGCGTCCACCGG	Alt-R Cas9 crRNA:tra	BMT2-T	C*G*CGCTGTTTACCTTCTCGC	PS modified	NA	NA	acactctttccctacacgacgctcttccgatctTTCCTCCCTCACTCAGC	gtgactggagttcagacgtgtgctcttccgatctGCCGAAATACTGCTCGT
1B, S1A	BMX	Cas9	Hs.Cas9.BMX.1	CGTCCACGAAGAACCCACTA	Alt-R Cas9 crRNA:tra	BMX-T	A*G*ATAAGGGGTAACCCCCA	PS modified	NA	NA	acactctttccctacacgacgctcttccgatctCTTGTTTTCTTCCTGTCTGCT	gtgactggagttcagacgtgtgctcttccgatctCACATTACATGCTTCCCAGAG
1B, S1A	BNIPL	Cas9	Hs.Cas9.BNIPL.1	GGTGTTCCGAATGGGACCAC	Alt-R Cas9 crRNA:tra	BNIPL-T	T*A*GGGCTCAATGACAGTCA	PS modified	NA	NA	acactctttccctacacgacgctcttccgatctGTTGTATGTGGGATGTGACT	gtgactggagttcagacgtgtgctcttccgatctATGCTAGCCATTACCTCCAT
1B, S1A	BOLL	Cas9	Hs.Cas9.BOLL.1	GGTGGGACCGAAGTTACCTC	Alt-R Cas9 crRNA:tra	BOLL-T	T*A*CTTACCATAATGGTGTTC	PS modified	NA	NA	acactctttccctacacgacgctcttccgatctGGTTGATGTGTGTTATTATTTGTAATTAT	gtgactggagttcagacgtgtgctcttccgatctCTAACAACTTCAACTGGATATCCTTATA
1B, S1A	BOP1	Cas9	Hs.Cas9.BOP1.1	GTACGATGACTTCCCCCACG	Alt-R Cas9 crRNA:tra	BOP1-T	G*G*CTTGTAGATGCGCCTGC	PS modified	NA	NA	acactctttccctacacgacgctcttccgatctAACTGGTCCAGCTCATCC	gtgactggagttcagacgtgtgctcttccgatctTAGGATCAGATGCCGACAT
1B, S1A	BRAF	Cas9	Hs.Cas9.BRAF.1	GAACTTCTGTACTACAACGC	Alt-R Cas9 crRNA:tra	BRAF-T	G*T*TTCCGCTGTCAAACATGT	PS modified	NA	NA	acactctttccctacacgacgctcttccgatctCCTCACAGACTTTTAGACATCGTAG	gtgactggagttcagacgtgtgctcttccgatctTCACCTTAGCATTTTGTGACTTTT
1B, S1A	BRD8	Cas9	Hs.Cas9.BRD8.1	CCGGAGGTTACCCACTGTGA	Alt-R Cas9 crRNA:tra	BRD8-T	C*C*TCCTGGGGAGGCAGAAT	PS modified	NA	NA	acactctttccctacacgacgctcttccgatctCTCTTCCATAGTGGTTGGAGT	gtgactggagttcagacgtgtgctcttccgatctCTGCTTTAGTAATGCAACATACCT
1B, S1A	BRDT	Cas9	Hs.Cas9.BRDT.1	TTCTCCCTTGAACGTGGTAC	Alt-R Cas9 crRNA:tra	BRDT-T	G*C*CGCAGTTTGTGAACTGG	PS modified	NA	NA	acactctttccctacacgacgctcttccgatctCGCAACAGAAAAAGTATTTAAGCAG	gtgactggagttcagacgtgtgctcttccgatctTTGCTAGACGCTGAAGACTAATTTT
1B, S1A	BRIX1	Cas9	Hs.Cas9.BRIX1.1	GCAACCAAGAGGAAACGGCG	Alt-R Cas9 crRNA:tra	BRIX1-T	T*C*TTTTTGGCTTCTTCGCCT	PS modified	NA	NA	acactctttccctacacgacgctcttccgatctGAGCCGCCGAACCATA	gtgactggagttcagacgtgtgctcttccgatctCGGCTCCGCATCTATTTC
1B, S1A	BSPRY	Cas9	Hs.Cas9.BSPRY.1	CAGGACGTCCGCCACATACT	Alt-R Cas9 crRNA:tra	BSPRY-T	T*G*TGAGAGGCTGCAGTTAC	PS modified	NA	NA	acactctttccctacacgacgctcttccgatctGAACAAGATTGTGGACCAGT	gtgactggagttcagacgtgtgctcttccgatctTCCCTAAGTCTAAGGCCTTAT
1B, S1A	BTBD3	Cas9	Hs.Cas9.BTBD3.1	TCAACGCCAGATCTTGTCGT	Alt-R Cas9 crRNA:tra	BTBD3-T	T*T*GAGGCAGCTCTCAACTG	PS modified	NA	NA	acactctttccctacacgacgctcttccgatctGAAACTCTGAATGCCAAAGAAATT	gtgactggagttcagacgtgtgctcttccgatctCAAGTAAAGTGCCTTTCCTAGAA
1B, S1A	BTF3L4	Cas9	Hs.Cas9.BTF3L4.1	AAGCTTTTTGTCATCAGCTG	Alt-R Cas9 crRNA:tra	BTF3L4-T	G*C*TCGCAGAAAGAAGAAG	PS modified	NA	NA	acactctttccctacacgacgctcttccgatctTGTTTGGTTATTTTTCAGGGTACA	gtgactggagttcagacgtgtgctcttccgatctGCAAAGTGATCATACCTCTTCAATA
1B, S1A	BTN3A1	Cas9	Hs.Cas9.BTN3A1.1	GAGGGAACTTCTGATGGTAC	Alt-R Cas9 crRNA:tra	BTN3A1-T	G*T*GATCATGAGAGGCAGCT	PS modified	NA	NA	acactctttccctacacgacgctcttccgatctAGTAGCAGCATCTGTGATCAT	gtgactggagttcagacgtgtgctcttccgatctAGGTTGACTGCAGACACTAA
1B, S1A	BTN3A2	Cas9	Hs.Cas9.BTN3A2.1	TAATCTTCACGTCGAAGTGA	Alt-R Cas9 crRNA:tra	BTN3A2-T	C*T*GCACTCCAGATGGATCC	PS modified	NA	NA	acactctttccctacacgacgctcttccgatctCAGGAGCTATCCAGAATTTAGGC	gtgactggagttcagacgtgtgctcttccgatctAGATGCTGCTACTTCATATAGGC
1B, S1A	BUD23	Cas9	Hs.Cas9.BUD23.1	GTAGTCTACCACCATGCCAC	Alt-R Cas9 crRNA:tra	BUD23-T	C*T*GATCACAACCCAGGCCA	PS modified	NA	NA	acactctttccctacacgacgctcttccgatctGCTGCCTTTCTTTCCTCA	gtgactggagttcagacgtgtgctcttccgatctGCTATGCCACTACCCTCC
1B, S1A	BZW1	Cas9	Hs.Cas9.BZW1.1	AACTGAAACCGGTACTGATT	Alt-R Cas9 crRNA:tra	BZW1-T	G*C*TCCAGAAGCATCAAGAA	PS modified	NA	NA	acactctttccctacacgacgctcttccgatctAGGTTTGACCCTACTCAGTTT	gtgactggagttcagacgtgtgctcttccgatctCGACGGTAATCAAGTTTTGCT
1B, S1A	C10orf111	Cas9	Hs.Cas9.C10orf111.1	TCGGAGTCGCTGCAAAGTCG	Alt-R Cas9 crRNA:tra	C10orf111-T	C*T*TTAAAATGTTATTCGGCC	PS modified	NA	NA	acactctttccctacacgacgctcttccgatctGTCTTCTAAGTTCTGGCCAA	gtgactggagttcagacgtgtgctcttccgatctGAATATCCCGTCCAGTGTC
1B, S1A	C10orf67	Cas9	Hs.Cas9.C10orf67.1	GGGGGACCTTTGGCACACGC	Alt-R Cas9 crRNA:tra	C10orf67-T	C*C*CGCAGCTCGGTGGCCTT	PS modified	NA	NA	acactctttccctacacgacgctcttccgatctTGAATTCCCGAGCTTCTCG	gtgactggagttcagacgtgtgctcttccgatctGATCGCAGGGCTCATTATGT
1B, S1A	C10orf90	Cas9	Hs.Cas9.C10orf90.1	ACCATCACGGCCAGACGCGT	Alt-R Cas9 crRNA:tra	C10orf90-T	A*G*CAGTCCCCCACACCAGG	PS modified	NA	NA	acactctttccctacacgacgctcttccgatctAACAGTGTGTCCTCAGC	gtgactggagttcagacgtgtgctcttccgatctGATTAGGAATCCCGGCAC
1B, S1A	C11orf57	Cas9	Hs.Cas9.C11orf57.1	ATGCTTACCGGGGGACCAAA	Alt-R Cas9 crRNA:tra	C11orf57-T	T*G*CAACTTCACCTTGAACTC	PS modified	NA	NA	acactctttccctacacgacgctcttccgatctAGGAATCAGATATGTGGAAAATAAGAG	gtgactggagttcagacgtgtgctcttccgatctGAATTATTTTAGTTCTCAGAGCTGCAA
1B, S1A	C11orf74	Cas9	Hs.Cas9.C11orf74.1	CTGGGCCACAAAAGGGATAC	Alt-R Cas9 crRNA:tra	C11orf74-T	G*A*AGTGGAGCAGGATGTA	PS modified	NA	NA	acactctttccctacacgacgctcttccgatctTTCCAGGAGAAGTGGAGCA	gtgactggagttcagacgtgtgctcttccgatctCTGTGGAGTACCTCTTCCGT
1B, S1A	C12orf54	Cas9	Hs.Cas9.C12orf54.1	GTCCTGGGTGCTGATGTCAT	Alt-R Cas9 crRNA:tra	C12orf54-T	G*T*CCACTTTAGGGATGAGC	PS modified	NA	NA	acactctttccctacacgacgctcttccgatctCAGAATGACTCTTCTCTGTGT	gtgactggagttcagacgtgtgctcttccgatctCCATCTCCCTTGAACATTGT
1B, S1A	C12orf77	Cas9	Hs.Cas9.C12orf77.1	AGACGCTGGACAGCATACGA	Alt-R Cas9 crRNA:tra	C12orf77-T	G*G*CATTCGGGGGCAGGTA	PS modified	NA	NA	acactctttccctacacgacgctcttccgatctTGTTTCAGTAGAGATGGCATATTT	gtgactggagttcagacgtgtgctcttccgatctCCTGACAGTTCTAATAAGGTACC
1B, S1A	C14orf132	Cas9	Hs.Cas9.C14orf132.1	TCGGTGCTGAAGTCCTCGTT	Alt-R Cas9 crRNA:tra	C14orf132-T	G*C*TGCCCATGATGGGGGA	PS modified	NA	NA	acactctttccctacacgacgctcttccgatctCATGGCCCTGGATAATTCT	gtgactggagttcagacgtgtgctcttccgatctAATCCATAGCAAGACGGC
1B, S1A	C15orf38-AP3S2	Cas9	Hs.Cas9.C15orf38-AP3S2.1	GGGGTACGGCTGAAGACTCG	Alt-R Cas9 crRNA:tra	C15orf38-AP3S2-T	A*G*GGTTGCATTGCTCACCC	PS modified	NA	NA	acactctttccctacacgacgctcttccgatctGGTTGCATTGCTCACC	gtgactggagttcagacgtgtgctcttccgatctACACAGTGGCGTTCTG
1B, S1A	C15orf53	Cas9	Hs.Cas9.C15orf53.1	CTGGTTTCATGGTTCCTCCG	Alt-R Cas9 crRNA:tra	C15orf53-T	G*G*CCCAAGAGGACCTGGG	PS modified	NA	NA	acactctttccctacacgacgctcttccgatctGGGACTTCAGTTAGTGACA	gtgactggagttcagacgtgtgctcttccgatctGAAACTGAGAACCCTGCTA
1B, S1A	C16orf58	Cas9	Hs.Cas9.C16orf58.1	ACGGAATCCCACAGCTGGTA	Alt-R Cas9 crRNA:tra	C16orf58-T	G*G*GCTTCCCTGATAGCGTC	PS modified	NA	NA	acactctttccctacacgacgctcttccgatctGCACTGTCCTCTGCCC	gtgactggagttcagacgtgtgctcttccgatctCGTGTTCCTGCCTCAGG
1B, S1A	C16orf82	Cas9	Hs.Cas9.C16orf82.1	ATCCTCTGTCCAGAATGAGC	Alt-R Cas9 crRNA:tra	C16orf82-T	T*C*TAAGCTGGGTGACTGTA	PS modified	NA	NA	acactctttccctacacgacgctcttccgatctTCACAGCCAACATTCAGAG	gtgactggagttcagacgtgtgctcttccgatctGCTCTAAGCTGGGTGACT
1B, S1A	C16orf90	Cas9	Hs.Cas9.C16orf90.1	CGCACCCCCCAACATCTACG	Alt-R Cas9 crRNA:tra	C16orf90-T	C*T*GGGGCACTGCGGCTGCC	PS modified	NA	NA	acactctttccctacacgacgctcttccgatctAAGTTCTTGGGCTTGCTT	gtgactggagttcagacgtgtgctcttccgatctCTGTCTCCTCTGCAGATG
1B, S1A	C17orf102	Cas9	Hs.Cas9.C17orf102.1	AAGGATGCACGCTCCCCACC	Alt-R Cas9 crRNA:tra	C17orf102-T	C*C*TCTGGAAGAGCGGCTGC	PS modified	NA	NA	acactctttccctacacgacgctcttccgatctTGATTCCCAGCCAGTG	gtgactggagttcagacgtgtgctcttccgatctCGTTTCTCCAGGGTAGA
1B, S1A	C17orf58	Cas9	Hs.Cas9.C17orf58.1	CCGAGTCCACATGTTAGCCC	Alt-R Cas9 crRNA:tra	C17orf58-T	A*A*CTCTGGACACGGCTTAT	PS modified	NA	NA	acactctttccctacacgacgctcttccgatctCAGGTTTAAACTCTGGACACG	gtgactggagttcagacgtgtgctcttccgatctGGGTAATGCTCTTCTCCAAA
1B, S1A	C17orf99	Cas9	Hs.Cas9.C17orf99.1	GCGGGCCAACTTCACTCTGC	Alt-R Cas9 crRNA:tra	C17orf99-T	C*A*GATCATCTCCACCCTGG	PS modified	NA	NA	acactctttccctacacgacgctcttccgatctCTACCTTCTCCCACCCTG	gtgactggagttcagacgtgtgctcttccgatctCTTCCCGATCAGGCTGTT
1B, S1A	C19orf12	Cas9	Hs.Cas9.C19orf12.1	GCCCACCAAACCCCCGACGA	Alt-R Cas9 crRNA:tra	C19orf12-T	T*C*TGGGAAGGGTGCCCTGC	PS modified	NA	NA	acactctttccctacacgacgctcttccgatctTGTGAGACACCTGCACTTA	gtgactggagttcagacgtgtgctcttccgatctATCATGAAGCTGCTGTGCT
1B, S1A	C19orf33	Cas9	Hs.Cas9.C19orf33.1	GTCCCCACAAAGTTCAGGGC	Alt-R Cas9 crRNA:tra	C19orf33-T	T*T*ACCTTTGGACCCGGACC	PS modified	NA	NA	acactctttccctacacgacgctcttccgatctCAACCACCCAACTTCTCTC	gtgactggagttcagacgtgtgctcttccgatctGACTTACCTTTGGACCCG
1B, S1A	C19orf60	Cas9	Hs.Cas9.C19orf60.1	GCTACCGGAGCACAGTGCAC	Alt-R Cas9 crRNA:tra	C19orf60-T	C*C*TCCCGCGAGGCGGCGG	PS modified	NA	NA	acactctttccctacacgacgctcttccgatctTCAGGGTCCCCACATG	gtgactggagttcagacgtgtgctcttccgatctAGCTCTGCTTCCACCG
1B, S1A	C19orf84	Cas9	Hs.Cas9.C19orf84.1	GGGGGCCTACACCTTCCAAC	Alt-R Cas9 crRNA:tra	C19orf84-T	C*C*TGCCTGGGAGCAGCAG	PS modified	NA	NA	acactctttccctacacgacgctcttccgatctTGTGCCTGACTTCCCAG	gtgactggagttcagacgtgtgctcttccgatctATACGCCTGGACACCCT
1B, S1A	C1D	Cas9	Hs.Cas9.C1D.1	AACCCAAGGAGTTAATCCTA	Alt-R Cas9 crRNA:tra	C1D-T	T*A*AAATCTTACCAATTCCTG	PS modified	NA	NA	acactctttccctacacgacgctcttccgatctGCTCACTTTCATAATTTCAACTCGAATT	gtgactggagttcagacgtgtgctcttccgatctCATTCACCATTTTATTCCATGAAATTTTT
1B, S1A	C1orf112	Cas9	Hs.Cas9.C1orf112.1	TTATTATGGACTGGTGCTTA	Alt-R Cas9 crRNA:tra	C1orf112-T	T*T*GTATTCTTTTCTTTTTATT	PS modified	NA	NA	acactctttccctacacgacgctcttccgatctGAGGTCCTAAGTTACTTGATGTGTTA	gtgactggagttcagacgtgtgctcttccgatctAGGAGAAAAGAATGTCTTCACACAA
1B, S1A	C1orf116	Cas9	Hs.Cas9.C1orf116.1	CTCAGCAAGGACGAACGCCA	Alt-R Cas9 crRNA:tra	C1orf116-T	C*A*GGAGGGTGGGATGAGC	PS modified	NA	NA	acactctttccctacacgacgctcttccgatctCTTCTGGCAATGTGGATATTC	gtgactggagttcagacgtgtgctcttccgatctTCAGGAAGTCATTGCTTTCC
1B, S1A	C1orf210	Cas9	Hs.Cas9.C1orf210.1	TGTGCTGGTAGGATTTGTGC	Alt-R Cas9 crRNA:tra	C1orf210-T	G*C*AAGTGCAATGAGGAGC	PS modified	NA	NA	acactctttccctacacgacgctcttccgatctTTTGGCAGCAAGTGCAAT	gtgactggagttcagacgtgtgctcttccgatctGTCCCTTTCTCTGCAGCA
1B, S1A	C1orf228	Cas9	Hs.Cas9.C1orf228.1	CAGGTCTTTGATCAACTCGA	Alt-R Cas9 crRNA:tra	C1orf228-T	G*G*GGCCTCTCTCCAGCACC	PS modified	NA	NA	acactctttccctacacgacgctcttccgatctCTGCACAGTGCTGATCAGTA	gtgactggagttcagacgtgtgctcttccgatctGAGATCTCCTTGACCGACG
1B, S1A	C1orf27	Cas9	Hs.Cas9.C1orf27.1	CACTAGAACGCCACCCAAAG	Alt-R Cas9 crRNA:tra	C1orf27-T	T*T*AGCTTTGGGATGTTTGA	PS modified	NA	NA	acactctttccctacacgacgctcttccgatctGCTTTTTAATTTGTTGTTGAAGTGTT	gtgactggagttcagacgtgtgctcttccgatctCATTCTTCATCCAAGTTATCCAACTTA
1B, S1A	C1orf61	Cas9	Hs.Cas9.C1orf61.1	TCTATCGTCCACACGGAGGA	Alt-R Cas9 crRNA:tra	C1orf61-T	G*G*GGCGGGTGGGTTCTGC	PS modified	NA	NA	acactctttccctacacgacgctcttccgatctCAAAGCTGACTGCAAACAATT	gtgactggagttcagacgtgtgctcttccgatctTCTCTTCCCTTTAGCTTCTCAC
1B, S1A	C1orf68	Cas9	Hs.Cas9.C1orf68.1	CACCCTGGACATAGCACGTC	Alt-R Cas9 crRNA:tra	C1orf68-T	T*G*ACCTACATCAAAAGTCC	PS modified	NA	NA	acactctttccctacacgacgctcttccgatctCAACCTATGTAAAATGCCCAA	gtgactggagttcagacgtgtgctcttccgatctCTTGCAGGAGCTTGAACATA
1B, S1A	C1QTNF8	Cas9	Hs.Cas9.C1QTNF8.1	GTTCACCAGCTCCGTGTCGA	Alt-R Cas9 crRNA:tra	C1QTNF8-T	G*G*CCTGCACAGCTCCGACC	PS modified	NA	NA	acactctttccctacacgacgctcttccgatctCAGGTGTGCACGTTGAG	gtgactggagttcagacgtgtgctcttccgatctCTACGCCGCCTTCTCC
1B, S1A	C20orf173	Cas9	Hs.Cas9.C20orf173.1	GCCACCACACAGCCGACGAA	Alt-R Cas9 crRNA:tra	C20orf173-T	C*C*ATAGTCTTCCTGGAGCA	PS modified	NA	NA	acactctttccctacacgacgctcttccgatctGGTACCCCATAGTCTTCCTG	gtgactggagttcagacgtgtgctcttccgatctTGTACTTGGTACCACAGCATT
1B, S1A	C20orf203	Cas9	Hs.Cas9.C20orf203.1	ACCTCTGGGACCTTGGCGGT	Alt-R Cas9 crRNA:tra	C20orf203-T	G*G*ACTCGCTGAGCCTTTGA	PS modified	NA	NA	acactctttccctacacgacgctcttccgatctCCACCAACCCAAATCCTTTC	gtgactggagttcagacgtgtgctcttccgatctCTTGCGAGTCTCAGGTACTAA
1B, S1A	C2CD2	Cas9	Hs.Cas9.C2CD2.1	CGACGCACACTGCATTAATG	Alt-R Cas9 crRNA:tra	C2CD2-T	A*C*GCCAACACGATGCCCTT	PS modified	NA	NA	acactctttccctacacgacgctcttccgatctAAGGTGAATTCCTCTTCCCA	gtgactggagttcagacgtgtgctcttccgatctAAAATAAACGCCAACACGATG
1B, S1A	C2orf15	Cas9	Hs.Cas9.C2orf15.1	TGGATGATCACTTAATACGA	Alt-R Cas9 crRNA:tra	C2orf15-T	A*C*TGAGTCGCTGGTTCCAA	PS modified	NA	NA	acactctttccctacacgacgctcttccgatctTTCACATTTGTTCAGCTATCCT	gtgactggagttcagacgtgtgctcttccgatctGAAATAACTGAGTCGCTGGTT
1B, S1A	C2orf16	Cas9	Hs.Cas9.C2orf16.1	GGGTTGACCCCAAAGCTAAC	Alt-R Cas9 crRNA:tra	C2orf16-T	C*T*GCAGCAGCATCCCTAAG	PS modified	NA	NA	acactctttccctacacgacgctcttccgatctCAGAATCTTCAGAAATGGCACAA	gtgactggagttcagacgtgtgctcttccgatctATTTTGGTACCTGAAGATCTGG
1B, S1A	C2orf70	Cas9	Hs.Cas9.C2orf70.1	CGGCATTGAACTCGGTCAGT	Alt-R Cas9 crRNA:tra	C2orf70-T	G*C*GCACCCACCATGGCCTC	PS modified	NA	NA	acactctttccctacacgacgctcttccgatctAGGCTCGCTGTACTCG	gtgactggagttcagacgtgtgctcttccgatctGAGGAGGGCGCTAGTG
1B, S1A	C2orf74	Cas9	Hs.Cas9.C2orf74.1	CCTTGTACAGATGCAAACGG	Alt-R Cas9 crRNA:tra	C2orf74-T	G*C*TTGTCACCACTTTGGCA	PS modified	NA	NA	acactctttccctacacgacgctcttccgatctCACCTTAAAATCAGGGCCATT	gtgactggagttcagacgtgtgctcttccgatctATTGCTTGTCACCACTTTGG
1B, S1A	ATAD3A	Cas9	Hs.Cas9.ATAD3A.1	AAGAATGCCACGCTTGTCGC	Alt-R Cas9 crRNA:tra	ATAD3A-T	G*G*ACGGCTTCCCCAGCCGA	PS modified	NA	NA	acactctttccctacacgacgctcttccgatctCAGTGCGGTGTCTCTG	gtgactggagttcagacgtgtgctcttccgatctCGCTGCTACCTGGATG
1B, S1A	ATAD3C	Cas9	Hs.Cas9.ATAD3C.1	GTCAAAGGACGCCCTGAATC	Alt-R Cas9 crRNA:tra	ATAD3C-T	T*G*CTCCAACTGCAGCGTCT	PS modified	NA	NA	acactctttccctacacgacgctcttccgatctTATCCTAACACCTGCCCTC	gtgactggagttcagacgtgtgctcttccgatctAATCTCCACGCCCGAAC
1D	GAPDH	Cas9	GAPDH-4216-AS.-22	TGGAGGCCATGTGGGCCATG	Alt-R Cas9 crRNA:tra	GAPDH 4216-AS -22 NT	T*T*CCTCTTGTGCTCTTGCTG	PS modified	NA	NA	ACACTCTTTCCCTACACGACGCTCTTCCGATCTGGTGGCTGGCTCAGAAA	GTGACTGGAGTTCAGACGTGTGCTCTTCCGATCTTGAGTGTGGCAGGGACT
1D	GAPDH	Cas9	GAPDH-4227-S.-17	GGTGGACCTCATGGCCCACA	Alt-R Cas9 crRNA:tra	GAPDH 4227-S -17 NT	G*A*ATTTGGCTACAGCAACA	PS modified	NA	NA	ACACTCTTTCCCTACACGACGCTCTTCCGATCTGGTGGCTGGCTCAGAAA	GTGACTGGAGTTCAGACGTGTGCTCTTCCGATCTTGAGTGTGGCAGGGACT
1D	GAPDH	Cas9	GAPDH-4224-AS.-14	TTACTCCTTGGAGGCCATGT	Alt-R Cas9 crRNA:tra	GAPDH 4224-AS -14 NT	T*T*CCTCTTGTGCTCTTGCTG	PS modified	NA	NA	ACACTCTTTCCCTACACGACGCTCTTCCGATCTGGTGGCTGGCTCAGAAA	GTGACTGGAGTTCAGACGTGTGCTCTTCCGATCTTGAGTGTGGCAGGGACT
1D	GAPDH	Cas9	GAPDH-4225-AS.-13	CTTACTCCTTGGAGGCCATG	Alt-R Cas9 crRNA:tra	GAPDH 4225-AS -13 NT	T*T*CCTCTTGTGCTCTTGCTG	PS modified	NA	NA	ACACTCTTTCCCTACACGACGCTCTTCCGATCTGGTGGCTGGCTCAGAAA	GTGACTGGAGTTCAGACGTGTGCTCTTCCGATCTTGAGTGTGGCAGGGACT
1D	GAPDH	Cas9	GAPDH-4236-S.-8	CATGGCCCACATGGCCTCCA	Alt-R Cas9 crRNA:tra	GAPDH 4236-S -8 NT	T*A*CAGCAACAGGGTGGTGG	PS modified	NA	NA	ACACTCTTTCCCTACACGACGCTCTTCCGATCTGGTGGCTGGCTCAGAAA	GTGACTGGAGTTCAGACGTGTGCTCTTCCGATCTTGAGTGTGGCAGGGACT
1D	GAPDH	Cas9	GAPDH-4233-AS.-5	CCAGGGGTCTTACTCCTTGG	Alt-R Cas9 crRNA:tra	GAPDH 4233-AS -5 NT	T*T*CCTCTTGTGCTCTTGCTG	PS modified	NA	NA	ACACTCTTTCCCTACACGACGCTCTTCCGATCTGGTGGCTGGCTCAGAAA	GTGACTGGAGTTCAGACGTGTGCTCTTCCGATCTTGAGTGTGGCAGGGACT
1D	GAPDH	Cas9	GAPDH-4236-AS.-2	GGTCCAGGGGTCTTACTCCT	Alt-R Cas9 crRNA:tra	GAPDH 4236-AS -2 NT	T*T*CCTCTTGTGCTCTTGCTG	PS modified	NA	NA	ACACTCTTTCCCTACACGACGCTCTTCCGATCTGGTGGCTGGCTCAGAAA	GTGACTGGAGTTCAGACGTGTGCTCTTCCGATCTTGAGTGTGGCAGGGACT
1D	GAPDH	Cas9	GAPDH-4250-S.+6	CCTCCAAGGAGTAAGACCCC	Alt-R Cas9 crRNA:tra	GAPDH 4250-S +6 NT	C*A*GGGTGGTGGACCTCATC	PS modified	NA	NA	ACACTCTTTCCCTACACGACGCTCTTCCGATCTGGTGGCTGGCTCAGAAA	GTGACTGGAGTTCAGACGTGTGCTCTTCCGATCTTGAGTGTGGCAGGGACT
1D	GAPDH	Cas9	GAPDH-4249-AS.+11	TGCTGGGGCTGGTGGTCCAG	Alt-R Cas9 crRNA:tra	GAPDH 4249-AS +11 NT	T*C*TCTCTCTTCCTCTTGTGCT	PS modified	NA	NA	ACACTCTTTCCCTACACGACGCTCTTCCGATCTGGTGGCTGGCTCAGAAA	GTGACTGGAGTTCAGACGTGTGCTCTTCCGATCTTGAGTGTGGCAGGGACT
1D	GAPDH	Cas9	GAPDH-4250-AS.+12	TTGCTGGGGCTGGTGGTCCA	Alt-R Cas9 crRNA:tra	GAPDH 4250-AS +12 NT	G*T*CTCTCTCTTCCTCTTGTG	PS modified	NA	NA	ACACTCTTTCCCTACACGACGCTCTTCCGATCTGGTGGCTGGCTCAGAAA	GTGACTGGAGTTCAGACGTGTGCTCTTCCGATCTTGAGTGTGGCAGGGACT
1D	GAPDH	Cas9	GAPDH-4251-AS.+13	CTTGCTGGGGCTGGTGGTCC	Alt-R Cas9 crRNA:tra	GAPDH 4251-AS +13 NT	G*G*TCTCTCTCTTCCTCTTGT	PS modified	NA	NA	ACACTCTTTCCCTACACGACGCTCTTCCGATCTGGTGGCTGGCTCAGAAA	GTGACTGGAGTTCAGACGTGTGCTCTTCCGATCTTGAGTGTGGCAGGGACT
1D	GAPDH	Cas9	GAPDH-4257-AS.+19	TGTGCTCTTGCTGGGGCTGG	Alt-R Cas9 crRNA:tra	GAPDH 4257-AS +19 NT	A*G*TGAGGGTCTCTCTCTTCC	PS modified	NA	NA	ACACTCTTTCCCTACACGACGCTCTTCCGATCTGGTGGCTGGCTCAGAAA	GTGACTGGAGTTCAGACGTGTGCTCTTCCGATCTTGAGTGTGGCAGGGACT
1D	GAPDH	Cas9	GAPDH-4260-AS.+22	TCTTGTGCTCTTGCTGGGGC	Alt-R Cas9 crRNA:tra	GAPDH 4260-AS +22 NT	A*G*CAGTGAGGGTCTCTCTC	PS modified	NA	NA	ACACTCTTTCCCTACACGACGCTCTTCCGATCTGGTGGCTGGCTCAGAAA	GTGACTGGAGTTCAGACGTGTGCTCTTCCGATCTTGAGTGTGGCAGGGACT
1D	GAPDH	Cas9	GAPDH-4216-AS.-22	TGGAGGCCATGTGGGCCATG	Alt-R Cas9 crRNA:tra	GAPDH 4216-AS -22 T	A*C*AACGAATTTGGCTACAG	PS modified	NA	NA	ACACTCTTTCCCTACACGACGCTCTTCCGATCTGGTGGCTGGCTCAGAAA	GTGACTGGAGTTCAGACGTGTGCTCTTCCGATCTTGAGTGTGGCAGGGACT
1D	GAPDH	Cas9	GAPDH-4227-S.-17	GGTGGACCTCATGGCCCACA	Alt-R Cas9 crRNA:tra	GAPDH 4227-S -17 T	T*T*CCTCTTGTGCTCTTGCTG	PS modified	NA	NA	ACACTCTTTCCCTACACGACGCTCTTCCGATCTGGTGGCTGGCTCAGAAA	GTGACTGGAGTTCAGACGTGTGCTCTTCCGATCTTGAGTGTGGCAGGGACT
1D	GAPDH	Cas9	GAPDH-4224-AS.-14	TTACTCCTTGGAGGCCATGT	Alt-R Cas9 crRNA:tra	GAPDH 4224-AS -14 T	T*T*TGGCTACAGCAACAGGG	PS modified	NA	NA	ACACTCTTTCCCTACACGACGCTCTTCCGATCTGGTGGCTGGCTCAGAAA	GTGACTGGAGTTCAGACGTGTGCTCTTCCGATCTTGAGTGTGGCAGGGACT
1D	GAPDH	Cas9	GAPDH-4225-AS.-13	CTTACTCCTTGGAGGCCATG	Alt-R Cas9 crRNA:tra	GAPDH 4225-AS -13 T	T*T*GGCTACAGCAACAGGGT	PS modified	NA	NA	ACACTCTTTCCCTACACGACGCTCTTCCGATCTGGTGGCTGGCTCAGAAA	GTGACTGGAGTTCAGACGTGTGCTCTTCCGATCTTGAGTGTGGCAGGGACT
1D	GAPDH	Cas9	GAPDH-4236-S.-8	CATGGCCCACATGGCCTCCA	Alt-R Cas9 crRNA:tra	GAPDH 4236-S -8 T	T*T*CCTCTTGTGCTCTTGCTG	PS modified	NA	NA	ACACTCTTTCCCTACACGACGCTCTTCCGATCTGGTGGCTGGCTCAGAAA	GTGACTGGAGTTCAGACGTGTGCTCTTCCGATCTTGAGTGTGGCAGGGACT
1D	GAPDH	Cas9	GAPDH-4233-AS.-5	CCAGGGGTCTTACTCCTTGG	Alt-R Cas9 crRNA:tra	GAPDH 4233-AS -5 T	A*G*CAACAGGGTGGTGGAC	PS modified	NA	NA	ACACTCTTTCCCTACACGACGCTCTTCCGATCTGGTGGCTGGCTCAGAAA	GTGACTGGAGTTCAGACGTGTGCTCTTCCGATCTTGAGTGTGGCAGGGACT
1D	GAPDH	Cas9	GAPDH-4236-AS.-2	GGTCCAGGGGTCTTACTCCT	Alt-R Cas9 crRNA:tra	GAPDH 4236-AS -2 T	A*A*CAGGGTGGTGGACCTCA	PS modified	NA	NA	ACACTCTTTCCCTACACGACGCTCTTCCGATCTGGTGGCTGGCTCAGAAA	GTGACTGGAGTTCAGACGTGTGCTCTTCCGATCTTGAGTGTGGCAGGGACT
1D	GAPDH	Cas9	GAPDH-4250-S.+6	CCTCCAAGGAGTAAGACCCC	Alt-R Cas9 crRNA:tra	GAPDH 4250-S +6 T	G*T*CTTCCTCTTGTGCTCTTG	PS modified	NA	NA	ACACTCTTTCCCTACACGACGCTCTTCCGATCTGGTGGCTGGCTCAGAAA	GTGACTGGAGTTCAGACGTGTGCTCTTCCGATCTTGAGTGTGGCAGGGACT
1D	GAPDH	Cas9	GAPDH-4249-AS.+11	TGCTGGGGCTGGTGGTCCAG	Alt-R Cas9 crRNA:tra	GAPDH 4249-AS +11 T	C*A*GGGTGGTGGACCTCATC	PS modified	NA	NA	ACACTCTTTCCCTACACGACGCTCTTCCGATCTGGTGGCTGGCTCAGAAA	GTGACTGGAGTTCAGACGTGTGCTCTTCCGATCTTGAGTGTGGCAGGGACT
1D	GAPDH	Cas9	GAPDH-4250-AS.+12	TTGCTGGGGCTGGTGGTCCA	Alt-R Cas9 crRNA:tra	GAPDH 4250-AS +12 T	C*A*GGGTGGTGGACCTCATC	PS modified	NA	NA	ACACTCTTTCCCTACACGACGCTCTTCCGATCTGGTGGCTGGCTCAGAAA	GTGACTGGAGTTCAGACGTGTGCTCTTCCGATCTTGAGTGTGGCAGGGACT
1D	GAPDH	Cas9	GAPDH-4251-AS.+13	CTTGCTGGGGCTGGTGGTCC	Alt-R Cas9 crRNA:tra	GAPDH 4251-AS +13 T	C*A*GGGTGGTGGACCTCATC	PS modified	NA	NA	ACACTCTTTCCCTACACGACGCTCTTCCGATCTGGTGGCTGGCTCAGAAA	GTGACTGGAGTTCAGACGTGTGCTCTTCCGATCTTGAGTGTGGCAGGGACT
1D	GAPDH	Cas9	GAPDH-4257-AS.+19	TGTGCTCTTGCTGGGGCTGG	Alt-R Cas9 crRNA:tra	GAPDH 4257-AS +19 T	C*A*GGGTGGTGGACCTCATC	PS modified	NA	NA	ACACTCTTTCCCTACACGACGCTCTTCCGATCTGGTGGCTGGCTCAGAAA	GTGACTGGAGTTCAGACGTGTGCTCTTCCGATCTTGAGTGTGGCAGGGACT
1D	GAPDH	Cas9	GAPDH-4260-AS.+22	TCTTGTGCTCTTGCTGGGGC	Alt-R Cas9 crRNA:tra	GAPDH 4260-AS +22 T	C*A*GGGTGGTGGACCTCATC	PS modified	NA	NA	ACACTCTTTCCCTACACGACGCTCTTCCGATCTGGTGGCTGGCTCAGAAA	GTGACTGGAGTTCAGACGTGTGCTCTTCCGATCTTGAGTGTGGCAGGGACT
2B, S2C	HPRT1	Cas9	HPRT1-51bp-PAM-out	TAACATACAGAGAGACTACA; T	Alt-R Cas9 crRNA:tra	HPRT1 51bp PAM-out A Top	G*T*GTTTAGAAACGTCAGTC	PS modified	AAGAATGTTGTGATAAAAGGTGATGCT	ACACATCCATGGGACTTCTGCCTC	NA	NA
2B, S2C	HPRT1	Cas9	HPRT1-51bp-PAM-out	TAACATACAGAGAGACTACA; T	Alt-R Cas9 crRNA:tra	HPRT1 51bp PAM-out A Btm	T*A*CAGTCTGGCTTATATCCA	PS modified	AAGAATGTTGTGATAAAAGGTGATGCT	ACACATCCATGGGACTTCTGCCTC	NA	NA
2B, S2C	HPRT1	Cas9	HPRT1-51bp-PAM-out	TAACATACAGAGAGACTACA; T	Alt-R Cas9 crRNA:tra	HPRT1 51bp PAM-out E Top	G*T*GTTTAGAAACGTCAGTC	PS modified	AAGAATGTTGTGATAAAAGGTGATGCT	ACACATCCATGGGACTTCTGCCTC	NA	NA
2B, S2C	HPRT1	Cas9	HPRT1-51bp-PAM-out	TAACATACAGAGAGACTACA; T	Alt-R Cas9 crRNA:tra	HPRT1 51bp PAM-out E Btm	T*A*CAGTCTGGCTTATATCCA	PS modified	AAGAATGTTGTGATAAAAGGTGATGCT	ACACATCCATGGGACTTCTGCCTC	NA	NA
2B, S2C	HPRT1	Cas9	HPRT1-51bp-PAM-out	TAACATACAGAGAGACTACA; T	Alt-R Cas9 crRNA:tra	HPRT1 51bp PAM-out B Top	G*T*GTTTAGAAACGTCAGTC	PS modified	AAGAATGTTGTGATAAAAGGTGATGCT	ACACATCCATGGGACTTCTGCCTC	NA	NA
2B, S2C	HPRT1	Cas9	HPRT1-51bp-PAM-out	TAACATACAGAGAGACTACA: T	Alt-R Cas9 crRNA:tra	HPRT1 51bp PAM-out B Btm	T*A*CAGTCTGGCTTATATCCA	PS modified	AAGAATGTTGTGATAAAAGGTGATGCT	ACACATCCATGGGACTTCTGCCTC	NA	NA
2B, S2C	HPRT1	Cas9	HPRT1-51bp-PAM-out	TAACATACAGAGAGACTACA: T	Alt-R Cas9 crRNA:tra	HPRT1 51bp PAM-out C Top	G*T*GTTTAGAAACGTCAGTC	PS modified	AAGAATGTTGTGATAAAAGGTGATGCT	ACACATCCATGGGACTTCTGCCTC	NA	NA
2B, S2C	HPRT1	Cas9	HPRT1-51bp-PAM-out	TAACATACAGAGAGACTACA: T	Alt-R Cas9 crRNA:tra	HPRT1 51bp PAM-out C Btm	T*A*CAGTCTGGCTTATATCCA	PS modified	AAGAATGTTGTGATAAAAGGTGATGCT	ACACATCCATGGGACTTCTGCCTC	NA	NA
2B, S2C	HPRT1	Cas9	HPRT1-51bp-PAM-out	TAACATACAGAGAGACTACA: T	Alt-R Cas9 crRNA:tra	HPRT1 51bp PAM-out D Top	G*T*GTTTAGAAACGTCAGTC	PS modified	AAGAATGTTGTGATAAAAGGTGATGCT	ACACATCCATGGGACTTCTGCCTC	NA	NA
2B, S2C	HPRT1	Cas9	HPRT1-51bp-PAM-out	TAACATACAGAGAGACTACA: T	Alt-R Cas9 crRNA:tra	HPRT1 51bp PAM-out D Btm	T*A*CAGTCTGGCTTATATCCA	PS modified	AAGAATGTTGTGATAAAAGGTGATGCT	ACACATCCATGGGACTTCTGCCTC	NA	NA
3B	SAA1	Cas9	SAA1-88	AAGTTTTAAAAATCACTCCT	Alt-R Cas9 crRNA:tra	SAA1-88-ctrl	TGTTGGGAAAGAGAAAACAG	Unmodified	NA	NA	ACACTCTTTCCCTACACGACGCTCTTCCGATCTGCTGTATGCCCCAGGATAA	GTGACTGGAGTTCAGACGTGTGCTCTTCCGATCTGACCAGCAAGGCTCACTATA
3B	SAA1	Cas9	SAA1-88	AAGTTTTAAAAATCACTCCT	Alt-R Cas9 crRNA:tra	SAA1-88-PAM-TGA	TGTTGGGAAAGAGAAAACAG	Unmodified	NA	NA	ACACTCTTTCCCTACACGACGCTCTTCCGATCTGCTGTATGCCCCAGGATAA	GTGACTGGAGTTCAGACGTGTGCTCTTCCGATCTGACCAGCAAGGCTCACTATA
3B	SAA1	Cas9	SAA1-88	AAGTTTTAAAAATCACTCCT	Alt-R Cas9 crRNA:tra	SAA1-88-PAM-TGC	TGTTGGGAAAGAGAAAACAG	Unmodified	NA	NA	ACACTCTTTCCCTACACGACGCTCTTCCGATCTGCTGTATGCCCCAGGATAA	GTGACTGGAGTTCAGACGTGTGCTCTTCCGATCTGACCAGCAAGGCTCACTATA
3B	SAA1	Cas9	SAA1-88	AAGTTTTAAAAATCACTCCT	Alt-R Cas9 crRNA:tra	SAA1-88-PAM-TGT	TGTTGGGAAAGAGAAAACAG	Unmodified	NA	NA	ACACTCTTTCCCTACACGACGCTCTTCCGATCTGCTGTATGCCCCAGGATAA	GTGACTGGAGTTCAGACGTGTGCTCTTCCGATCTGACCAGCAAGGCTCACTATA
3B	SAA1	Cas9	SAA1-88	AAGTTTTAAAAATCACTCCT	Alt-R Cas9 crRNA:tra	SAA1-88-PAM-TAG	TGTTGGGAAAGAGAAAACAG	Unmodified	NA	NA	ACACTCTTTCCCTACACGACGCTCTTCCGATCTGCTGTATGCCCCAGGATAA	GTGACTGGAGTTCAGACGTGTGCTCTTCCGATCTGACCAGCAAGGCTCACTATA
3B	SAA1	Cas9	SAA1-88	AAGTTTTAAAAATCACTCCT	Alt-R Cas9 crRNA:tra	SAA1-88-PAM-TCG	TGTTGGGAAAGAGAAAACAG	Unmodified	NA	NA	ACACTCTTTCCCTACACGACGCTCTTCCGATCTGCTGTATGCCCCAGGATAA	GTGACTGGAGTTCAGACGTGTGCTCTTCCGATCTGACCAGCAAGGCTCACTATA
3B	SAA1	Cas9	SAA1-88	AAGTTTTAAAAATCACTCCT	Alt-R Cas9 crRNA:tra	SAA1-88-PAM-TTG	TGTTGGGAAAGAGAAAACAG	Unmodified	NA	NA	ACACTCTTTCCCTACACGACGCTCTTCCGATCTGCTGTATGCCCCAGGATAA	GTGACTGGAGTTCAGACGTGTGCTCTTCCGATCTGACCAGCAAGGCTCACTATA
3B	SAA1	Cas9	SAA1-88	AAGTTTTAAAAATCACTCCT	Alt-R Cas9 crRNA:tra	SAA1-88-PAM-AGG	TGTTGGGAAAGAGAAAACAG	Unmodified	NA	NA	ACACTCTTTCCCTACACGACGCTCTTCCGATCTGCTGTATGCCCCAGGATAA	GTGACTGGAGTTCAGACGTGTGCTCTTCCGATCTGACCAGCAAGGCTCACTATA
3B	SAA1	Cas9	SAA1-88	AAGTTTTAAAAATCACTCCT	Alt-R Cas9 crRNA:tra	SAA1-88-PAM-CGG	TGTTGGGAAAGAGAAAACAG	Unmodified	NA	NA	ACACTCTTTCCCTACACGACGCTCTTCCGATCTGCTGTATGCCCCAGGATAA	GTGACTGGAGTTCAGACGTGTGCTCTTCCGATCTGACCAGCAAGGCTCACTATA
3B	SAA1	Cas9	SAA1-88	AAGTTTTAAAAATCACTCCT	Alt-R Cas9 crRNA:tra	SAA1-88-PAM-GGG	TGTTGGGAAAGAGAAAACAG	Unmodified	NA	NA	ACACTCTTTCCCTACACGACGCTCTTCCGATCTGCTGTATGCCCCAGGATAA	GTGACTGGAGTTCAGACGTGTGCTCTTCCGATCTGACCAGCAAGGCTCACTATA
3B	SAA1	Cas9	SAA1-88	AAGTTTTAAAAATCACTCCT	Alt-R Cas9 crRNA:tra	SAA1-88-1A	TGTTGGGAAAGAGAAAACAG	Unmodified	NA	NA	ACACTCTTTCCCTACACGACGCTCTTCCGATCTGCTGTATGCCCCAGGATAA	GTGACTGGAGTTCAGACGTGTGCTCTTCCGATCTGACCAGCAAGGCTCACTATA
3B	SAA1	Cas9	SAA1-88	AAGTTTTAAAAATCACTCCT	Alt-R Cas9 crRNA:tra	SAA1-88-1C	TGTTGGGAAAGAGAAAACAG	Unmodified	NA	NA	ACACTCTTTCCCTACACGACGCTCTTCCGATCTGCTGTATGCCCCAGGATAA	GTGACTGGAGTTCAGACGTGTGCTCTTCCGATCTGACCAGCAAGGCTCACTATA
3B	SAA1	Cas9	SAA1-88	AAGTTTTAAAAATCACTCCT	Alt-R Cas9 crRNA:tra	SAA1-88-1G	TGTTGGGAAAGAGAAAACAG	Unmodified	NA	NA	ACACTCTTTCCCTACACGACGCTCTTCCGATCTGCTGTATGCCCCAGGATAA	GTGACTGGAGTTCAGACGTGTGCTCTTCCGATCTGACCAGCAAGGCTCACTATA
3B	SAA1	Cas9	SAA1-88	AAGTTTTAAAAATCACTCCT	Alt-R Cas9 crRNA:tra	SAA1-88-2A	TGTTGGGAAAGAGAAAACAG	Unmodified	NA	NA	ACACTCTTTCCCTACACGACGCTCTTCCGATCTGCTGTATGCCCCAGGATAA	GTGACTGGAGTTCAGACGTGTGCTCTTCCGATCTGACCAGCAAGGCTCACTATA
3B	SAA1	Cas9	SAA1-88	AAGTTTTAAAAATCACTCCT	Alt-R Cas9 crRNA:tra	SAA1-88-2T	TGTTGGGAAAGAGAAAACAG	Unmodified	NA	NA	ACACTCTTTCCCTACACGACGCTCTTCCGATCTGCTGTATGCCCCAGGATAA	GTGACTGGAGTTCAGACGTGTGCTCTTCCGATCTGACCAGCAAGGCTCACTATA
3B	SAA1	Cas9	SAA1-88	AAGTTTTAAAAATCACTCCT	Alt-R Cas9 crRNA:tra	SAA1-88-2G	TGTTGGGAAAGAGAAAACAG	Unmodified	NA	NA	ACACTCTTTCCCTACACGACGCTCTTCCGATCTGCTGTATGCCCCAGGATAA	GTGACTGGAGTTCAGACGTGTGCTCTTCCGATCTGACCAGCAAGGCTCACTATA
3B	SAA1	Cas9	SAA1-88	AAGTTTTAAAAATCACTCCT	Alt-R Cas9 crRNA:tra	SAA1-88-3A	TGTTGGGAAAGAGAAAACAG	Unmodified	NA	NA	ACACTCTTTCCCTACACGACGCTCTTCCGATCTGCTGTATGCCCCAGGATAA	GTGACTGGAGTTCAGACGTGTGCTCTTCCGATCTGACCAGCAAGGCTCACTATA
3B	SAA1	Cas9	SAA1-88	AAGTTTTAAAAATCACTCCT	Alt-R Cas9 crRNA:tra	SAA1-88-3T	TGTTGGGAAAGAGAAAACAG	Unmodified	NA	NA	ACACTCTTTCCCTACACGACGCTCTTCCGATCTGCTGTATGCCCCAGGATAA	GTGACTGGAGTTCAGACGTGTGCTCTTCCGATCTGACCAGCAAGGCTCACTATA
3B	SAA1	Cas9	SAA1-88	AAGTTTTAAAAATCACTCCT	Alt-R Cas9 crRNA:tra	SAA1-88-3G	TGTTGGGAAAGAGAAAACAG	Unmodified	NA	NA	ACACTCTTTCCCTACACGACGCTCTTCCGATCTGCTGTATGCCCCAGGATAA	GTGACTGGAGTTCAGACGTGTGCTCTTCCGATCTGACCAGCAAGGCTCACTATA
3B	SAA1	Cas9	SAA1-88	AAGTTTTAAAAATCACTCCT	Alt-R Cas9 crRNA:tra	SAA1-88-4A	TGTTGGGAAAGAGAAAACAG	Unmodified	NA	NA	ACACTCTTTCCCTACACGACGCTCTTCCGATCTGCTGTATGCCCCAGGATAA	GTGACTGGAGTTCAGACGTGTGCTCTTCCGATCTGACCAGCAAGGCTCACTATA
3B	SAA1	Cas9	SAA1-88	AAGTTTTAAAAATCACTCCT	Alt-R Cas9 crRNA:tra	SAA1-88-4C	TGTTGGGAAAGAGAAAACAG	Unmodified	NA	NA	ACACTCTTTCCCTACACGACGCTCTTCCGATCTGCTGTATGCCCCAGGATAA	GTGACTGGAGTTCAGACGTGTGCTCTTCCGATCTGACCAGCAAGGCTCACTATA
3B	SAA1	Cas9	SAA1-88	AAGTTTTAAAAATCACTCCT	Alt-R Cas9 crRNA:tra	SAA1-88-4G	TGTTGGGAAAGAGAAAACAG	Unmodified	NA	NA	ACACTCTTTCCCTACACGACGCTCTTCCGATCTGCTGTATGCCCCAGGATAA	GTGACTGGAGTTCAGACGTGTGCTCTTCCGATCTGACCAGCAAGGCTCACTATA
3B	SAA1	Cas9	SAA1-88	AAGTTTTAAAAATCACTCCT	Alt-R Cas9 crRNA:tra	SAA1-88-5A	TGTTGGGAAAGAGAAAACAG	Unmodified	NA	NA	ACACTCTTTCCCTACACGACGCTCTTCCGATCTGCTGTATGCCCCAGGATAA	GTGACTGGAGTTCAGACGTGTGCTCTTCCGATCTGACCAGCAAGGCTCACTATA
3B	SAA1	Cas9	SAA1-88	AAGTTTTAAAAATCACTCCT	Alt-R Cas9 crRNA:tra	SAA1-88-5T	TGTTGGGAAAGAGAAAACAG	Unmodified	NA	NA	ACACTCTTTCCCTACACGACGCTCTTCCGATCTGCTGTATGCCCCAGGATAA	GTGACTGGAGTTCAGACGTGTGCTCTTCCGATCTGACCAGCAAGGCTCACTATA
3B	SAA1	Cas9	SAA1-88	AAGTTTTAAAAATCACTCCT	Alt-R Cas9 crRNA:tra	SAA1-88-5G	TGTTGGGAAAGAGAAAACAG	Unmodified	NA	NA	ACACTCTTTCCCTACACGACGCTCTTCCGATCTGCTGTATGCCCCAGGATAA	GTGACTGGAGTTCAGACGTGTGCTCTTCCGATCTGACCAGCAAGGCTCACTATA
3B	SAA1	Cas9	SAA1-88	AAGTTTTAAAAATCACTCCT	Alt-R Cas9 crRNA:tra	SAA1-88-6C	TGTTGGGAAAGAGAAAACAG	Unmodified	NA	NA	ACACTCTTTCCCTACACGACGCTCTTCCGATCTGCTGTATGCCCCAGGATAA	GTGACTGGAGTTCAGACGTGTGCTCTTCCGATCTGACCAGCAAGGCTCACTATA
3B	SAA1	Cas9	SAA1-88	AAGTTTTAAAAATCACTCCT	Alt-R Cas9 crRNA:tra	SAA1-88-6T	TGTTGGGAAAGAGAAAACAG	Unmodified	NA	NA	ACACTCTTTCCCTACACGACGCTCTTCCGATCTGCTGTATGCCCCAGGATAA	GTGACTGGAGTTCAGACGTGTGCTCTTCCGATCTGACCAGCAAGGCTCACTATA
3B	SAA1	Cas9	SAA1-88	AAGTTTTAAAAATCACTCCT	Alt-R Cas9 crRNA:tra	SAA1-88-6G	TGTTGGGAAAGAGAAAACAG	Unmodified	NA	NA	ACACTCTTTCCCTACACGACGCTCTTCCGATCTGCTGTATGCCCCAGGATAA	GTGACTGGAGTTCAGACGTGTGCTCTTCCGATCTGACCAGCAAGGCTCACTATA
3B	SAA1	Cas9	SAA1-88	AAGTTTTAAAAATCACTCCT	Alt-R Cas9 crRNA:tra	SAA1-88-7A	TGTTGGGAAAGAGAAAACAG	Unmodified	NA	NA	ACACTCTTTCCCTACACGACGCTCTTCCGATCTGCTGTATGCCCCAGGATAA	GTGACTGGAGTTCAGACGTGTGCTCTTCCGATCTGACCAGCAAGGCTCACTATA
3B	SAA1	Cas9	SAA1-88	AAGTTTTAAAAATCACTCCT	Alt-R Cas9 crRNA:tra	SAA1-88-7T	TGTTGGGAAAGAGAAAACAG	Unmodified	NA	NA	ACACTCTTTCCCTACACGACGCTCTTCCGATCTGCTGTATGCCCCAGGATAA	GTGACTGGAGTTCAGACGTGTGCTCTTCCGATCTGACCAGCAAGGCTCACTATA
3B	SAA1	Cas9	SAA1-88	AAGTTTTAAAAATCACTCCT	Alt-R Cas9 crRNA:tra	SAA1-88-7G	TGTTGGGAAAGAGAAAACAG	Unmodified	NA	NA	ACACTCTTTCCCTACACGACGCTCTTCCGATCTGCTGTATGCCCCAGGATAA	GTGACTGGAGTTCAGACGTGTGCTCTTCCGATCTGACCAGCAAGGCTCACTATA
3B	SAA1	Cas9	SAA1-88	AAGTTTTAAAAATCACTCCT	Alt-R Cas9 crRNA:tra	SAA1-88-8A	TGTTGGGAAAGAGAAAACAG	Unmodified	NA	NA	ACACTCTTTCCCTACACGACGCTCTTCCGATCTGCTGTATGCCCCAGGATAA	GTGACTGGAGTTCAGACGTGTGCTCTTCCGATCTGACCAGCAAGGCTCACTATA
3B	SAA1	Cas9	SAA1-88	AAGTTTTAAAAATCACTCCT	Alt-R Cas9 crRNA:tra	SAA1-88-8C	TGTTGGGAAAGAGAAAACAG	Unmodified	NA	NA	ACACTCTTTCCCTACACGACGCTCTTCCGATCTGCTGTATGCCCCAGGATAA	GTGACTGGAGTTCAGACGTGTGCTCTTCCGATCTGACCAGCAAGGCTCACTATA
3B	SAA1	Cas9	SAA1-88	AAGTTTTAAAAATCACTCCT	Alt-R Cas9 crRNA:tra	SAA1-88-8G	TGTTGGGAAAGAGAAAACAG	Unmodified	NA	NA	ACACTCTTTCCCTACACGACGCTCTTCCGATCTGCTGTATGCCCCAGGATAA	GTGACTGGAGTTCAGACGTGTGCTCTTCCGATCTGACCAGCAAGGCTCACTATA
3B	SAA1	Cas9	SAA1-88	AAGTTTTAAAAATCACTCCT	Alt-R Cas9 crRNA:tra	SAA1-88-9C	TGTTGGGAAAGAGAAAACAG	Unmodified	NA	NA	ACACTCTTTCCCTACACGACGCTCTTCCGATCTGCTGTATGCCCCAGGATAA	GTGACTGGAGTTCAGACGTGTGCTCTTCCGATCTGACCAGCAAGGCTCACTATA
3B	SAA1	Cas9	SAA1-88	AAGTTTTAAAAATCACTCCT	Alt-R Cas9 crRNA:tra	SAA1-88-9T	TGTTGGGAAAGAGAAAACAG	Unmodified	NA	NA	ACACTCTTTCCCTACACGACGCTCTTCCGATCTGCTGTATGCCCCAGGATAA	GTGACTGGAGTTCAGACGTGTGCTCTTCCGATCTGACCAGCAAGGCTCACTATA
3B	SAA1	Cas9	SAA1-88	AAGTTTTAAAAATCACTCCT	Alt-R Cas9 crRNA:tra	SAA1-88-9G	TGTTGGGAAAGAGAAAACAG	Unmodified	NA	NA	ACACTCTTTCCCTACACGACGCTCTTCCGATCTGCTGTATGCCCCAGGATAA	GTGACTGGAGTTCAGACGTGTGCTCTTCCGATCTGACCAGCAAGGCTCACTATA
3B	SAA1	Cas9	SAA1-88	AAGTTTTAAAAATCACTCCT	Alt-R Cas9 crRNA:tra	SAA1-88-10C	TGTTGGGAAAGAGAAAACAG	Unmodified	NA	NA	ACACTCTTTCCCTACACGACGCTCTTCCGATCTGCTGTATGCCCCAGGATAA	GTGACTGGAGTTCAGACGTGTGCTCTTCCGATCTGACCAGCAAGGCTCACTATA
3B	SAA1	Cas9	SAA1-88	AAGTTTTAAAAATCACTCCT	Alt-R Cas9 crRNA:tra	SAA1-88-10T	TGTTGGGAAAGAGAAAACAG	Unmodified	NA	NA	ACACTCTTTCCCTACACGACGCTCTTCCGATCTGCTGTATGCCCCAGGATAA	GTGACTGGAGTTCAGACGTGTGCTCTTCCGATCTGACCAGCAAGGCTCACTATA
3B	SAA1	Cas9	SAA1-88	AAGTTTTAAAAATCACTCCT	Alt-R Cas9 crRNA:tra	SAA1-88-10G	TGTTGGGAAAGAGAAAACAG	Unmodified	NA	NA	ACACTCTTTCCCTACACGACGCTCTTCCGATCTGCTGTATGCCCCAGGATAA	GTGACTGGAGTTCAGACGTGTGCTCTTCCGATCTGACCAGCAAGGCTCACTATA
3B	SAA1	Cas9	SAA1-88	AAGTTTTAAAAATCACTCCT	Alt-R Cas9 crRNA:tra	SAA1-88-11C	TGTTGGGAAAGAGAAAACAG	Unmodified	NA	NA	ACACTCTTTCCCTACACGACGCTCTTCCGATCTGCTGTATGCCCCAGGATAA	GTGACTGGAGTTCAGACGTGTGCTCTTCCGATCTGACCAGCAAGGCTCACTATA
3B	SAA1	Cas9	SAA1-88	AAGTTTTAAAAATCACTCCT	Alt-R Cas9 crRNA:tra	SAA1-88-11T	TGTTGGGAAAGAGAAAACAG	Unmodified	NA	NA	ACACTCTTTCCCTACACGACGCTCTTCCGATCTGCTGTATGCCCCAGGATAA	GTGACTGGAGTTCAGACGTGTGCTCTTCCGATCTGACCAGCAAGGCTCACTATA
3B	SAA1	Cas9	SAA1-88	AAGTTTTAAAAATCACTCCT	Alt-R Cas9 crRNA:tra	SAA1-88-11G	TGTTGGGAAAGAGAAAACAG	Unmodified	NA	NA	ACACTCTTTCCCTACACGACGCTCTTCCGATCTGCTGTATGCCCCAGGATAA	GTGACTGGAGTTCAGACGTGTGCTCTTCCGATCTGACCAGCAAGGCTCACTATA
3B	SAA1	Cas9	SAA1-88	AAGTTTTAAAAATCACTCCT	Alt-R Cas9 crRNA:tra	SAA1-88-12C	TGTTGGGAAAGAGAAAACAG	Unmodified	NA	NA	ACACTCTTTCCCTACACGACGCTCTTCCGATCTGCTGTATGCCCCAGGATAA	GTGACTGGAGTTCAGACGTGTGCTCTTCCGATCTGACCAGCAAGGCTCACTATA
3B	SAA1	Cas9	SAA1-88	AAGTTTTAAAAATCACTCCT	Alt-R Cas9 crRNA:tra	SAA1-88-12T	TGTTGGGAAAGAGAAAACAG	Unmodified	NA	NA	ACACTCTTTCCCTACACGACGCTCTTCCGATCTGCTGTATGCCCCAGGATAA	GTGACTGGAGTTCAGACGTGTGCTCTTCCGATCTGACCAGCAAGGCTCACTATA
3B	SAA1	Cas9	SAA1-88	AAGTTTTAAAAATCACTCCT	Alt-R Cas9 crRNA:tra	SAA1-88-12G	TGTTGGGAAAGAGAAAACAG	Unmodified	NA	NA	ACACTCTTTCCCTACACGACGCTCTTCCGATCTGCTGTATGCCCCAGGATAA	GTGACTGGAGTTCAGACGTGTGCTCTTCCGATCTGACCAGCAAGGCTCACTATA
3B	STAT3	Cas9	STAT3-105	CTTGGATTGAGAGTCAAGAT	Alt-R Cas9 crRNA:tra	STAT3-105-ctrl	GGAGCTGCGGCAGTTTCTGG	Unmodified	NA	NA	ACACTCTTTCCCTACACGACGCTCTTCCGATCTGAACAGCAAGGCATGACATT	GTGACTGGAGTTCAGACGTGTGCTCTTCCGATCTCTCCATCAGCTCTACAGTGA
3B	STAT3	Cas9	STAT3-105	CTTGGATTGAGAGTCAAGAT	Alt-R Cas9 crRNA:tra	STAT3-105-PAM-TGA	GGAGCTGCGGCAGTTTCTGG	Unmodified	NA	NA	ACACTCTTTCCCTACACGACGCTCTTCCGATCTGAACAGCAAGGCATGACATT	GTGACTGGAGTTCAGACGTGTGCTCTTCCGATCTCTCCATCAGCTCTACAGTGA
3B	STAT3	Cas9	STAT3-105	CTTGGATTGAGAGTCAAGAT	Alt-R Cas9 crRNA:tra	STAT3-105-PAM-TGC	GGAGCTGCGGCAGTTTCTGG	Unmodified	NA	NA	ACACTCTTTCCCTACACGACGCTCTTCCGATCTGAACAGCAAGGCATGACATT	GTGACTGGAGTTCAGACGTGTGCTCTTCCGATCTCTCCATCAGCTCTACAGTGA

3B	STAT3	Cas9	STAT3-105	CTTGGATTGAGAGTCAAGAT	Alt-R Cas9 crRNA:tra	STAT3-105-PAM-TGT	GGAGCTGCGGCAGTTTCTGG	Unmodified	NA	NA	ACACTCTTTCCCTACACGACGCTCTTCCGATCTGAACAGCAAGGCATGACATT	GTGACTGGAGTTCAGACGTGTGCTCTTCCGATCTCTCCATCAGCTCTACAGTGA
3B	STAT3	Cas9	STAT3-105	CTTGGATTGAGAGTCAAGAT	Alt-R Cas9 crRNA:tra	STAT3-105-PAM-TAG	GGAGCTGCGGCAGTTTCTGG	Unmodified	NA	NA	ACACTCTTTCCCTACACGACGCTCTTCCGATCTGAACAGCAAGGCATGACATT	GTGACTGGAGTTCAGACGTGTGCTCTTCCGATCTCTCCATCAGCTCTACAGTGA
3B	STAT3	Cas9	STAT3-105	CTTGGATTGAGAGTCAAGAT	Alt-R Cas9 crRNA:tra	STAT3-105-PAM-TCG	GGAGCTGCGGCAGTTTCTGG	Unmodified	NA	NA	ACACTCTTTCCCTACACGACGCTCTTCCGATCTGAACAGCAAGGCATGACATT	GTGACTGGAGTTCAGACGTGTGCTCTTCCGATCTCTCCATCAGCTCTACAGTGA
3B	STAT3	Cas9	STAT3-105	CTTGGATTGAGAGTCAAGAT	Alt-R Cas9 crRNA:tra	STAT3-105-PAM-TTG	GGAGCTGCGGCAGTTTCTGG	Unmodified	NA	NA	ACACTCTTTCCCTACACGACGCTCTTCCGATCTGAACAGCAAGGCATGACATT	GTGACTGGAGTTCAGACGTGTGCTCTTCCGATCTCTCCATCAGCTCTACAGTGA
3B	STAT3	Cas9	STAT3-105	CTTGGATTGAGAGTCAAGAT	Alt-R Cas9 crRNA:tra	STAT3-105-PAM-AGG	GGAGCTGCGGCAGTTTCTGG	Unmodified	NA	NA	ACACTCTTTCCCTACACGACGCTCTTCCGATCTGAACAGCAAGGCATGACATT	GTGACTGGAGTTCAGACGTGTGCTCTTCCGATCTCTCCATCAGCTCTACAGTGA
3B	STAT3	Cas9	STAT3-105	CTTGGATTGAGAGTCAAGAT	Alt-R Cas9 crRNA:tra	STAT3-105-PAM-CGG	GGAGCTGCGGCAGTTTCTGG	Unmodified	NA	NA	ACACTCTTTCCCTACACGACGCTCTTCCGATCTGAACAGCAAGGCATGACATT	GTGACTGGAGTTCAGACGTGTGCTCTTCCGATCTCTCCATCAGCTCTACAGTGA
3B	STAT3	Cas9	STAT3-105	CTTGGATTGAGAGTCAAGAT	Alt-R Cas9 crRNA:tra	STAT3-105-PAM-GGG	GGAGCTGCGGCAGTTTCTGG	Unmodified	NA	NA	ACACTCTTTCCCTACACGACGCTCTTCCGATCTGAACAGCAAGGCATGACATT	GTGACTGGAGTTCAGACGTGTGCTCTTCCGATCTCTCCATCAGCTCTACAGTGA
3B	STAT3	Cas9	STAT3-105	CTTGGATTGAGAGTCAAGAT	Alt-R Cas9 crRNA:tra	STAT3-105-1A	GGAGCTGCGGCAGTTTCTGG	Unmodified	NA	NA	ACACTCTTTCCCTACACGACGCTCTTCCGATCTGAACAGCAAGGCATGACATT	GTGACTGGAGTTCAGACGTGTGCTCTTCCGATCTCTCCATCAGCTCTACAGTGA
3B	STAT3	Cas9	STAT3-105	CTTGGATTGAGAGTCAAGAT	Alt-R Cas9 crRNA:tra	STAT3-105-1C	GGAGCTGCGGCAGTTTCTGG	Unmodified	NA	NA	ACACTCTTTCCCTACACGACGCTCTTCCGATCTGAACAGCAAGGCATGACATT	GTGACTGGAGTTCAGACGTGTGCTCTTCCGATCTCTCCATCAGCTCTACAGTGA
3B	STAT3	Cas9	STAT3-105	CTTGGATTGAGAGTCAAGAT	Alt-R Cas9 crRNA:tra	STAT3-105-1G	GGAGCTGCGGCAGTTTCTGG	Unmodified	NA	NA	ACACTCTTTCCCTACACGACGCTCTTCCGATCTGAACAGCAAGGCATGACATT	GTGACTGGAGTTCAGACGTGTGCTCTTCCGATCTCTCCATCAGCTCTACAGTGA
3B	STAT3	Cas9	STAT3-105	CTTGGATTGAGAGTCAAGAT	Alt-R Cas9 crRNA:tra	STAT3-105-2C	GGAGCTGCGGCAGTTTCTGG	Unmodified	NA	NA	ACACTCTTTCCCTACACGACGCTCTTCCGATCTGAACAGCAAGGCATGACATT	GTGACTGGAGTTCAGACGTGTGCTCTTCCGATCTCTCCATCAGCTCTACAGTGA
3B	STAT3	Cas9	STAT3-105	CTTGGATTGAGAGTCAAGAT	Alt-R Cas9 crRNA:tra	STAT3-105-2T	GGAGCTGCGGCAGTTTCTGG	Unmodified	NA	NA	ACACTCTTTCCCTACACGACGCTCTTCCGATCTGAACAGCAAGGCATGACATT	GTGACTGGAGTTCAGACGTGTGCTCTTCCGATCTCTCCATCAGCTCTACAGTGA
3B	STAT3	Cas9	STAT3-105	CTTGGATTGAGAGTCAAGAT	Alt-R Cas9 crRNA:tra	STAT3-105-2G	GGAGCTGCGGCAGTTTCTGG	Unmodified	NA	NA	ACACTCTTTCCCTACACGACGCTCTTCCGATCTGAACAGCAAGGCATGACATT	GTGACTGGAGTTCAGACGTGTGCTCTTCCGATCTCTCCATCAGCTCTACAGTGA
3B	STAT3	Cas9	STAT3-105	CTTGGATTGAGAGTCAAGAT	Alt-R Cas9 crRNA:tra	STAT3-105-3A	GGAGCTGCGGCAGTTTCTGG	Unmodified	NA	NA	ACACTCTTTCCCTACACGACGCTCTTCCGATCTGAACAGCAAGGCATGACATT	GTGACTGGAGTTCAGACGTGTGCTCTTCCGATCTCTCCATCAGCTCTACAGTGA
3B	STAT3	Cas9	STAT3-105	CTTGGATTGAGAGTCAAGAT	Alt-R Cas9 crRNA:tra	STAT3-105-3C	GGAGCTGCGGCAGTTTCTGG	Unmodified	NA	NA	ACACTCTTTCCCTACACGACGCTCTTCCGATCTGAACAGCAAGGCATGACATT	GTGACTGGAGTTCAGACGTGTGCTCTTCCGATCTCTCCATCAGCTCTACAGTGA
3B	STAT3	Cas9	STAT3-105	CTTGGATTGAGAGTCAAGAT	Alt-R Cas9 crRNA:tra	STAT3-105-3T	GGAGCTGCGGCAGTTTCTGG	Unmodified	NA	NA	ACACTCTTTCCCTACACGACGCTCTTCCGATCTGAACAGCAAGGCATGACATT	GTGACTGGAGTTCAGACGTGTGCTCTTCCGATCTCTCCATCAGCTCTACAGTGA
3B	STAT3	Cas9	STAT3-105	CTTGGATTGAGAGTCAAGAT	Alt-R Cas9 crRNA:tra	STAT3-105-4C	GGAGCTGCGGCAGTTTCTGG	Unmodified	NA	NA	ACACTCTTTCCCTACACGACGCTCTTCCGATCTGAACAGCAAGGCATGACATT	GTGACTGGAGTTCAGACGTGTGCTCTTCCGATCTCTCCATCAGCTCTACAGTGA
3B	STAT3	Cas9	STAT3-105	CTTGGATTGAGAGTCAAGAT	Alt-R Cas9 crRNA:tra	STAT3-105-4T	GGAGCTGCGGCAGTTTCTGG	Unmodified	NA	NA	ACACTCTTTCCCTACACGACGCTCTTCCGATCTGAACAGCAAGGCATGACATT	GTGACTGGAGTTCAGACGTGTGCTCTTCCGATCTCTCCATCAGCTCTACAGTGA
3B	STAT3	Cas9	STAT3-105	CTTGGATTGAGAGTCAAGAT	Alt-R Cas9 crRNA:tra	STAT3-105-4G	GGAGCTGCGGCAGTTTCTGG	Unmodified	NA	NA	ACACTCTTTCCCTACACGACGCTCTTCCGATCTGAACAGCAAGGCATGACATT	GTGACTGGAGTTCAGACGTGTGCTCTTCCGATCTCTCCATCAGCTCTACAGTGA
3B	STAT3	Cas9	STAT3-105	CTTGGATTGAGAGTCAAGAT	Alt-R Cas9 crRNA:tra	STAT3-105-5C	GGAGCTGCGGCAGTTTCTGG	Unmodified	NA	NA	ACACTCTTTCCCTACACGACGCTCTTCCGATCTGAACAGCAAGGCATGACATT	GTGACTGGAGTTCAGACGTGTGCTCTTCCGATCTCTCCATCAGCTCTACAGTGA
3B	STAT3	Cas9	STAT3-105	CTTGGATTGAGAGTCAAGAT	Alt-R Cas9 crRNA:tra	STAT3-105-5T	GGAGCTGCGGCAGTTTCTGG	Unmodified	NA	NA	ACACTCTTTCCCTACACGACGCTCTTCCGATCTGAACAGCAAGGCATGACATT	GTGACTGGAGTTCAGACGTGTGCTCTTCCGATCTCTCCATCAGCTCTACAGTGA
3B	STAT3	Cas9	STAT3-105	CTTGGATTGAGAGTCAAGAT	Alt-R Cas9 crRNA:tra	STAT3-105-5G	GGAGCTGCGGCAGTTTCTGG	Unmodified	NA	NA	ACACTCTTTCCCTACACGACGCTCTTCCGATCTGAACAGCAAGGCATGACATT	GTGACTGGAGTTCAGACGTGTGCTCTTCCGATCTCTCCATCAGCTCTACAGTGA
3B	STAT3	Cas9	STAT3-105	CTTGGATTGAGAGTCAAGAT	Alt-R Cas9 crRNA:tra	STAT3-105-6A	GGAGCTGCGGCAGTTTCTGG	Unmodified	NA	NA	ACACTCTTTCCCTACACGACGCTCTTCCGATCTGAACAGCAAGGCATGACATT	GTGACTGGAGTTCAGACGTGTGCTCTTCCGATCTCTCCATCAGCTCTACAGTGA
3B	STAT3	Cas9	STAT3-105	CTTGGATTGAGAGTCAAGAT	Alt-R Cas9 crRNA:tra	STAT3-105-6T	GGAGCTGCGGCAGTTTCTGG	Unmodified	NA	NA	ACACTCTTTCCCTACACGACGCTCTTCCGATCTGAACAGCAAGGCATGACATT	GTGACTGGAGTTCAGACGTGTGCTCTTCCGATCTCTCCATCAGCTCTACAGTGA
3B	STAT3	Cas9	STAT3-105	CTTGGATTGAGAGTCAAGAT	Alt-R Cas9 crRNA:tra	STAT3-105-6G	GGAGCTGCGGCAGTTTCTGG	Unmodified	NA	NA	ACACTCTTTCCCTACACGACGCTCTTCCGATCTGAACAGCAAGGCATGACATT	GTGACTGGAGTTCAGACGTGTGCTCTTCCGATCTCTCCATCAGCTCTACAGTGA
3B	STAT3	Cas9	STAT3-105	CTTGGATTGAGAGTCAAGAT	Alt-R Cas9 crRNA:tra	STAT3-105-7A	GGAGCTGCGGCAGTTTCTGG	Unmodified	NA	NA	ACACTCTTTCCCTACACGACGCTCTTCCGATCTGAACAGCAAGGCATGACATT	GTGACTGGAGTTCAGACGTGTGCTCTTCCGATCTCTCCATCAGCTCTACAGTGA
3B	STAT3	Cas9	STAT3-105	CTTGGATTGAGAGTCAAGAT	Alt-R Cas9 crRNA:tra	STAT3-105-7C	GGAGCTGCGGCAGTTTCTGG	Unmodified	NA	NA	ACACTCTTTCCCTACACGACGCTCTTCCGATCTGAACAGCAAGGCATGACATT	GTGACTGGAGTTCAGACGTGTGCTCTTCCGATCTCTCCATCAGCTCTACAGTGA
3B	STAT3	Cas9	STAT3-105	CTTGGATTGAGAGTCAAGAT	Alt-R Cas9 crRNA:tra	STAT3-105-7G	GGAGCTGCGGCAGTTTCTGG	Unmodified	NA	NA	ACACTCTTTCCCTACACGACGCTCTTCCGATCTGAACAGCAAGGCATGACATT	GTGACTGGAGTTCAGACGTGTGCTCTTCCGATCTCTCCATCAGCTCTACAGTGA
3B	STAT3	Cas9	STAT3-105	CTTGGATTGAGAGTCAAGAT	Alt-R Cas9 crRNA:tra	STAT3-105-8A	GGAGCTGCGGCAGTTTCTGG	Unmodified	NA	NA	ACACTCTTTCCCTACACGACGCTCTTCCGATCTGAACAGCAAGGCATGACATT	GTGACTGGAGTTCAGACGTGTGCTCTTCCGATCTCTCCATCAGCTCTACAGTGA
3B	STAT3	Cas9	STAT3-105	CTTGGATTGAGAGTCAAGAT	Alt-R Cas9 crRNA:tra	STAT3-105-8C	GGAGCTGCGGCAGTTTCTGG	Unmodified	NA	NA	ACACTCTTTCCCTACACGACGCTCTTCCGATCTGAACAGCAAGGCATGACATT	GTGACTGGAGTTCAGACGTGTGCTCTTCCGATCTCTCCATCAGCTCTACAGTGA
3B	STAT3	Cas9	STAT3-105	CTTGGATTGAGAGTCAAGAT	Alt-R Cas9 crRNA:tra	STAT3-105-8T	GGAGCTGCGGCAGTTTCTGG	Unmodified	NA	NA	ACACTCTTTCCCTACACGACGCTCTTCCGATCTGAACAGCAAGGCATGACATT	GTGACTGGAGTTCAGACGTGTGCTCTTCCGATCTCTCCATCAGCTCTACAGTGA
3B	STAT3	Cas9	STAT3-105	CTTGGATTGAGAGTCAAGAT	Alt-R Cas9 crRNA:tra	STAT3-105-9C	GGAGCTGCGGCAGTTTCTGG	Unmodified	NA	NA	ACACTCTTTCCCTACACGACGCTCTTCCGATCTGAACAGCAAGGCATGACATT	GTGACTGGAGTTCAGACGTGTGCTCTTCCGATCTCTCCATCAGCTCTACAGTGA
3B	STAT3	Cas9	STAT3-105	CTTGGATTGAGAGTCAAGAT	Alt-R Cas9 crRNA:tra	STAT3-105-9T	GGAGCTGCGGCAGTTTCTGG	Unmodified	NA	NA	ACACTCTTTCCCTACACGACGCTCTTCCGATCTGAACAGCAAGGCATGACATT	GTGACTGGAGTTCAGACGTGTGCTCTTCCGATCTCTCCATCAGCTCTACAGTGA
3B	STAT3	Cas9	STAT3-105	CTTGGATTGAGAGTCAAGAT	Alt-R Cas9 crRNA:tra	STAT3-105-9G	GGAGCTGCGGCAGTTTCTGG	Unmodified	NA	NA	ACACTCTTTCCCTACACGACGCTCTTCCGATCTGAACAGCAAGGCATGACATT	GTGACTGGAGTTCAGACGTGTGCTCTTCCGATCTCTCCATCAGCTCTACAGTGA
3B	STAT3	Cas9	STAT3-105	CTTGGATTGAGAGTCAAGAT	Alt-R Cas9 crRNA:tra	STAT3-105-10A	GGAGCTGCGGCAGTTTCTGG	Unmodified	NA	NA	ACACTCTTTCCCTACACGACGCTCTTCCGATCTGAACAGCAAGGCATGACATT	GTGACTGGAGTTCAGACGTGTGCTCTTCCGATCTCTCCATCAGCTCTACAGTGA
3B	STAT3	Cas9	STAT3-105	CTTGGATTGAGAGTCAAGAT	Alt-R Cas9 crRNA:tra	STAT3-105-10C	GGAGCTGCGGCAGTTTCTGG	Unmodified	NA	NA	ACACTCTTTCCCTACACGACGCTCTTCCGATCTGAACAGCAAGGCATGACATT	GTGACTGGAGTTCAGACGTGTGCTCTTCCGATCTCTCCATCAGCTCTACAGTGA
3B	STAT3	Cas9	STAT3-105	CTTGGATTGAGAGTCAAGAT	Alt-R Cas9 crRNA:tra	STAT3-105-10T	GGAGCTGCGGCAGTTTCTGG	Unmodified	NA	NA	ACACTCTTTCCCTACACGACGCTCTTCCGATCTGAACAGCAAGGCATGACATT	GTGACTGGAGTTCAGACGTGTGCTCTTCCGATCTCTCCATCAGCTCTACAGTGA
3B	STAT3	Cas9	STAT3-105	CTTGGATTGAGAGTCAAGAT	Alt-R Cas9 crRNA:tra	STAT3-105-11C	GGAGCTGCGGCAGTTTCTGG	Unmodified	NA	NA	ACACTCTTTCCCTACACGACGCTCTTCCGATCTGAACAGCAAGGCATGACATT	GTGACTGGAGTTCAGACGTGTGCTCTTCCGATCTCTCCATCAGCTCTACAGTGA
3B	STAT3	Cas9	STAT3-105	CTTGGATTGAGAGTCAAGAT	Alt-R Cas9 crRNA:tra	STAT3-105-11T	GGAGCTGCGGCAGTTTCTGG	Unmodified	NA	NA	ACACTCTTTCCCTACACGACGCTCTTCCGATCTGAACAGCAAGGCATGACATT	GTGACTGGAGTTCAGACGTGTGCTCTTCCGATCTCTCCATCAGCTCTACAGTGA
3B	STAT3	Cas9	STAT3-105	CTTGGATTGAGAGTCAAGAT	Alt-R Cas9 crRNA:tra	STAT3-105-11G	GGAGCTGCGGCAGTTTCTGG	Unmodified	NA	NA	ACACTCTTTCCCTACACGACGCTCTTCCGATCTGAACAGCAAGGCATGACATT	GTGACTGGAGTTCAGACGTGTGCTCTTCCGATCTCTCCATCAGCTCTACAGTGA
3B	STAT3	Cas9	STAT3-105	CTTGGATTGAGAGTCAAGAT	Alt-R Cas9 crRNA:tra	STAT3-105-12A	GGAGCTGCGGCAGTTTCTGG	Unmodified	NA	NA	ACACTCTTTCCCTACACGACGCTCTTCCGATCTGAACAGCAAGGCATGACATT	GTGACTGGAGTTCAGACGTGTGCTCTTCCGATCTCTCCATCAGCTCTACAGTGA
3B	STAT3	Cas9	STAT3-105	CTTGGATTGAGAGTCAAGAT	Alt-R Cas9 crRNA:tra	STAT3-105-12C	GGAGCTGCGGCAGTTTCTGG	Unmodified	NA	NA	ACACTCTTTCCCTACACGACGCTCTTCCGATCTGAACAGCAAGGCATGACATT	GTGACTGGAGTTCAGACGTGTGCTCTTCCGATCTCTCCATCAGCTCTACAGTGA
3B	STAT3	Cas9	STAT3-105	CTTGGATTGAGAGTCAAGAT	Alt-R Cas9 crRNA:tra	STAT3-105-12T	GGAGCTGCGGCAGTTTCTGG	Unmodified	NA	NA	ACACTCTTTCCCTACACGACGCTCTTCCGATCTGAACAGCAAGGCATGACATT	GTGACTGGAGTTCAGACGTGTGCTCTTCCGATCTCTCCATCAGCTCTACAGTGA
3D	ACVR1C	Cas9	Hs.Cas9.ACVR1C.1.AA	TGTAGGAGCACTGTCGACCC	Alt-R Cas9 crRNA:tra	Hs.Cas9.ACVR1C.1.AA 3nt MNP	C*A*CATTTGGTCTCTTTTTCT	PS modified	NA	NA	ACACTCTTTCCCTACACGACGCTCTTCCGATCTCAGCATTTACCAGATTGCACT	GTGACTGGAGTTCAGACGTGTGCTCTTCCGATCTGCTGGCCATCATTATTACTGTG
3D	ADCY2	Cas9	Hs.Cas9.ADCY2.1.AA	GACGCTAAGCACGATGGTGT	Alt-R Cas9 crRNA:tra	Hs.Cas9.ADCY2.1.AA 1nt INS	C*A*GACCAGGTGCTCCTTGC	PS modified	NA	NA	ACACTCTTTCCCTACACGACGCTCTTCCGATCTGCCCGTAGGTATCGTTCTTC	GTGACTGGAGTTCAGACGTGTGCTCTTCCGATCTTGGGATGTGGAAAGTCATTCT
3D	AGAP3	Cas9	Hs.Cas9.AGAP3.1.AA	TGGTGCACCGCTATCTGACG	Alt-R Cas9 crRNA:tra	Hs.Cas9.AGAP3.1.AA 20nt INS	C*A*GGGCATAGTGGGGAAC	PS modified	NA	NA	ACACTCTTTCCCTACACGACGCTCTTCCGATCTTCTGCCCTCTGACCCA	GTGACTGGAGTTCAGACGTGTGCTCTTCCGATCTGGCCTGTGACGCTCAC
3D	AAVS1	Cas9	AAVS1-670	CCTCTAAGGTTTGCTTACGA	Alt-R Cas9 crRNA:tra	Hs.AAVS1670 20nt DEL	G*C*AGATTCCTTATCTGGTG	PS modified	NA	NA	ACACTCTTTCCCTACACGACGCTCTTCCGATCTTGCCAAGCTCTCCCTC	GTGACTGGAGTTCAGACGTGTGCTCTTCCGATCTCCATCCTTAGGCCTCCT
3D	ACVR1C	Cas9	Hs.Cas9.ACVR1C.1.AA	TGTAGGAGCACTGTCGACCC	Alt-R Cas9 crRNA:tra	Hs.Cas9.ACVR1C.1.AA 3nt MNP+silent mutation	C*A*CATTTGGTCTCTTTTTCT	PS modified	NA	NA	ACACTCTTTCCCTACACGACGCTCTTCCGATCTCAGCATTTACCAGATTGCACT	GTGACTGGAGTTCAGACGTGTGCTCTTCCGATCTGCTGGCCATCATTATTACTGTG
3D	ADCY2	Cas9	Hs.Cas9.ADCY2.1.AA	GACGCTAAGCACGATGGTGT	Alt-R Cas9 crRNA:tra	Hs.Cas9.ADCY2.1.AA 1nt INS+silent mutation	C*A*GACCAGGTGCTCCTTGC	PS modified	NA	NA	ACACTCTTTCCCTACACGACGCTCTTCCGATCTGCCCGTAGGTATCGTTCTTC	GTGACTGGAGTTCAGACGTGTGCTCTTCCGATCTTGGGATGTGGAAAGTCATTCT
3D	AGAP3	Cas9	Hs.Cas9.AGAP3.1.AA	TGGTGCACCGCTATCTGACG	Alt-R Cas9 crRNA:tra	Hs.Cas9.AGAP3.1.AA 20nt INS+silent mutation	C*A*GGGCATAGTGGGGAAC	PS modified	NA	NA	ACACTCTTTCCCTACACGACGCTCTTCCGATCTTCTGCCCTCTGACCCA	GTGACTGGAGTTCAGACGTGTGCTCTTCCGATCTGGCCTGTGACGCTCAC
3D	AAVS1	Cas9	AAVS1-670	CCTCTAAGGTTTGCTTACGA	Alt-R Cas9 crRNA:tra	Hs.AAVS1670 20nt DEL+silent mutation	G*C*AGATTCCTTATCTGGTG	PS modified	NA	NA	ACACTCTTTCCCTACACGACGCTCTTCCGATCTTGCCAAGCTCTCCCTC	GTGACTGGAGTTCAGACGTGTGCTCTTCCGATCTCCATCCTTAGGCCTCCT
4A	SERPINC1	Cas9	SERPINC1-166	ACCTCTGGAAAAAGGTAAGA	Alt-R Cas9 crRNA:tra	SERPINC1-166-NT-distal-none	C*C*CTGTGGAAGATTAGCG	PS modified	NA	NA	ACACTCTTTCCCTACACGACGCTCTTCCGATCTCTGGTCTTCTCAAAGGTGTTG	GTGACTGGAGTTCAGACGTGTGCTCTTCCGATCTTTGCCTCAGATCACACTATCT
4A	SERPINC1	Cas9	SERPINC1-166	ACCTCTGGAAAAAGGTAAGA	Alt-R Cas9 crRNA:tra	SERPINC1-166-NT-distal-PAM	C*C*CTGTGGAAGATTAGCG	PS modified	NA	NA	ACACTCTTTCCCTACACGACGCTCTTCCGATCTCTGGTCTTCTCAAAGGTGTTG	GTGACTGGAGTTCAGACGTGTGCTCTTCCGATCTTTGCCTCAGATCACACTATCT
4A	SERPINC1	Cas9	SERPINC1-166	ACCTCTGGAAAAAGGTAAGA	Alt-R Cas9 crRNA:tra	SERPINC1-166-NT-distal-repair track	C*C*CTGTGGAAGATTAGCG	PS modified	NA	NA	ACACTCTTTCCCTACACGACGCTCTTCCGATCTCTGGTCTTCTCAAAGGTGTTG	GTGACTGGAGTTCAGACGTGTGCTCTTCCGATCTTTGCCTCAGATCACACTATCT
4A	SERPINC1	Cas9	SERPINC1-166	ACCTCTGGAAAAAGGTAAGA	Alt-R Cas9 crRNA:tra	SERPINC1-166-NT-distal-PAM-repair track	C*C*CTGTGGAAGATTAGCG	PS modified	NA	NA	ACACTCTTTCCCTACACGACGCTCTTCCGATCTCTGGTCTTCTCAAAGGTGTTG	GTGACTGGAGTTCAGACGTGTGCTCTTCCGATCTTTGCCTCAGATCACACTATCT
4A	SERPINC1	Cas9	SERPINC1-166	ACCTCTGGAAAAAGGTAAGA	Alt-R Cas9 crRNA:tra	SERPINC1-166-NT-prox-none	A*T*TCCAATGTGATAGGAAC	PS modified	NA	NA	ACACTCTTTCCCTACACGACGCTCTTCCGATCTCTGGTCTTCTCAAAGGTGTTG	GTGACTGGAGTTCAGACGTGTGCTCTTCCGATCTTTGCCTCAGATCACACTATCT
4A	SERPINC1	Cas9	SERPINC1-166	ACCTCTGGAAAAAGGTAAGA	Alt-R Cas9 crRNA:tra	SERPINC1-166-NT-prox-PAM	A*T*TCCAATGTGATAGGAAC	PS modified	NA	NA	ACACTCTTTCCCTACACGACGCTCTTCCGATCTCTGGTCTTCTCAAAGGTGTTG	GTGACTGGAGTTCAGACGTGTGCTCTTCCGATCTTTGCCTCAGATCACACTATCT
4A	SERPINC1	Cas9	SERPINC1-166	ACCTCTGGAAAAAGGTAAGA	Alt-R Cas9 crRNA:tra	SERPINC1-166-NT-prox-repair track	A*T*TCCAATGTGATAGGAAC	PS modified	NA	NA	ACACTCTTTCCCTACACGACGCTCTTCCGATCTCTGGTCTTCTCAAAGGTGTTG	GTGACTGGAGTTCAGACGTGTGCTCTTCCGATCTTTGCCTCAGATCACACTATCT
4A	SERPINC1	Cas9	SERPINC1-166	ACCTCTGGAAAAAGGTAAGA	Alt-R Cas9 crRNA:tra	SERPINC1-166-NT-prox-PAM-repair track	A*T*TCCAATGTGATAGGAAC	PS modified	NA	NA	ACACTCTTTCCCTACACGACGCTCTTCCGATCTCTGGTCTTCTCAAAGGTGTTG	GTGACTGGAGTTCAGACGTGTGCTCTTCCGATCTTTGCCTCAGATCACACTATCT
4A	SERPINC1	Cas9	SERPINC1-166	ACCTCTGGAAAAAGGTAAGA	Alt-R Cas9 crRNA:tra	SERPINC1-166-T-distal-none	A*A*ACCCAGTAGGGGCAGG	PS modified	NA	NA	ACACTCTTTCCCTACACGACGCTCTTCCGATCTCTGGTCTTCTCAAAGGTGTTG	GTGACTGGAGTTCAGACGTGTGCTCTTCCGATCTTTGCCTCAGATCACACTATCT
4A	SERPINC1	Cas9	SERPINC1-166	ACCTCTGGAAAAAGGTAAGA	Alt-R Cas9 crRNA:tra	SERPINC1-166-T-distal-PAM	A*A*ACCCAGTAGGGGCAGG	PS modified	NA	NA	ACACTCTTTCCCTACACGACGCTCTTCCGATCTCTGGTCTTCTCAAAGGTGTTG	GTGACTGGAGTTCAGACGTGTGCTCTTCCGATCTTTGCCTCAGATCACACTATCT
4A	SERPINC1	Cas9	SERPINC1-166	ACCTCTGGAAAAAGGTAAGA	Alt-R Cas9 crRNA:tra	SERPINC1-166-T-distal-repair track	A*A*ACCCAGTAGGGGCAGG	PS modified	NA	NA	ACACTCTTTCCCTACACGACGCTCTTCCGATCTCTGGTCTTCTCAAAGGTGTTG	GTGACTGGAGTTCAGACGTGTGCTCTTCCGATCTTTGCCTCAGATCACACTATCT
4A	SERPINC1	Cas9	SERPINC1-166	ACCTCTGGAAAAAGGTAAGA	Alt-R Cas9 crRNA:tra	SERPINC1-166-T-distal-PAM-repair track	A*A*ACCCAGTAGGGGCAGG	PS modified	NA	NA	ACACTCTTTCCCTACACGACGCTCTTCCGATCTCTGGTCTTCTCAAAGGTGTTG	GTGACTGGAGTTCAGACGTGTGCTCTTCCGATCTTTGCCTCAGATCACACTATCT
4A	SERPINC1	Cas9	SERPINC1-166	ACCTCTGGAAAAAGGTAAGA	Alt-R Cas9 crRNA:tra	SERPINC1-166-T-prox-none	T*C*CTGTGAGTCCTTTGGAG	PS modified	NA	NA	ACACTCTTTCCCTACACGACGCTCTTCCGATCTCTGGTCTTCTCAAAGGTGTTG	GTGACTGGAGTTCAGACGTGTGCTCTTCCGATCTTTGCCTCAGATCACACTATCT
4A	SERPINC1	Cas9	SERPINC1-166	ACCTCTGGAAAAAGGTAAGA	Alt-R Cas9 crRNA:tra	SERPINC1-166-T-prox-PAM	T*C*CTGTGAGTCCTTTGGAG	PS modified	NA	NA	ACACTCTTTCCCTACACGACGCTCTTCCGATCTCTGGTCTTCTCAAAGGTGTTG	GTGACTGGAGTTCAGACGTGTGCTCTTCCGATCTTTGCCTCAGATCACACTATCT
4A	SERPINC1	Cas9	SERPINC1-166	ACCTCTGGAAAAAGGTAAGA	Alt-R Cas9 crRNA:tra	SERPINC1-166-T-prox-repair track	T*C*CTGTGAGTCCTTTGGAG	PS modified	NA	NA	ACACTCTTTCCCTACACGACGCTCTTCCGATCTCTGGTCTTCTCAAAGGTGTTG	GTGACTGGAGTTCAGACGTGTGCTCTTCCGATCTTTGCCTCAGATCACACTATCT
4A	SERPINC1	Cas9	SERPINC1-166	ACCTCTGGAAAAAGGTAAGA	Alt-R Cas9 crRNA:tra	SERPINC1-166-T-prox-PAM-repair track	T*C*CTGTGAGTCCTTTGGAG	PS modified	NA	NA	ACACTCTTTCCCTACACGACGCTCTTCCGATCTCTGGTCTTCTCAAAGGTGTTG	GTGACTGGAGTTCAGACGTGTGCTCTTCCGATCTTTGCCTCAGATCACACTATCT
4A	HPRT1	Cas9	HPRT-38087	AATTATGGGGATTACTAGGA	Alt-R Cas9 crRNA:tra	HPRT-38087-NT-distal-none	A*T*GGTCACTTTTAACACAC	PS modified	NA	NA	ACACTCTTTCCCTACACGACGCTCTTCCGATCTCAGAACTGTCCTTCAGGTTC	GTGACTGGAGTTCAGACGTGTGCTCTTCCGATCTCACTGTTTCATTTCATCCGTG
4A	HPRT1	Cas9	HPRT-38087	AATTATGGGGATTACTAGGA	Alt-R Cas9 crRNA:tra	HPRT-38087-NT-distal-PAM	A*T*GGTCACTTTTAACACAC	PS modified	NA	NA	ACACTCTTTCCCTACACGACGCTCTTCCGATCTCAGAACTGTCCTTCAGGTTC	GTGACTGGAGTTCAGACGTGTGCTCTTCCGATCTCACTGTTTCATTTCATCCGTG
4A	HPRT1	Cas9	HPRT-38087	AATTATGGGGATTACTAGGA	Alt-R Cas9 crRNA:tra	HPRT-38087-NT-distal-repair track	A*T*GGTCACTTTTAACACAC	PS modified	NA	NA	ACACTCTTTCCCTACACGACGCTCTTCCGATCTCAGAACTGTCCTTCAGGTTC	GTGACTGGAGTTCAGACGTGTGCTCTTCCGATCTCACTGTTTCATTTCATCCGTG
4A	HPRT1	Cas9	HPRT-38087	AATTATGGGGATTACTAGGA	Alt-R Cas9 crRNA:tra	HPRT-38087-NT-distal-PAM-repair track	A*T*GGTCACTTTTAACACAC	PS modified	NA	NA	ACACTCTTTCCCTACACGACGCTCTTCCGATCTCAGAACTGTCCTTCAGGTTC	GTGACTGGAGTTCAGACGTGTGCTCTTCCGATCTCACTGTTTCATTTCATCCGTG
4A	HPRT1	Cas9	HPRT-38087	AATTATGGGGATTACTAGGA	Alt-R Cas9 crRNA:tra	HPRT-38087-NT-prox-none	A*A*AGACTATGAAATGGAG	PS modified	NA	NA	ACACTCTTTCCCTACACGACGCTCTTCCGATCTCAGAACTGTCCTTCAGGTTC	GTGACTGGAGTTCAGACGTGTGCTCTTCCGATCTCACTGTTTCATTTCATCCGTG
4A	HPRT1	Cas9	HPRT-38087	AATTATGGGGATTACTAGGA	Alt-R Cas9 crRNA:tra	HPRT-38087-NT-prox-PAM	A*A*AGACTATGAAATGGAG	PS modified	NA	NA	ACACTCTTTCCCTACACGACGCTCTTCCGATCTCAGAACTGTCCTTCAGGTTC	GTGACTGGAGTTCAGACGTGTGCTCTTCCGATCTCACTGTTTCATTTCATCCGTG
4A	HPRT1	Cas9	HPRT-38087	AATTATGGGGATTACTAGGA	Alt-R Cas9 crRNA:tra	HPRT-38087-NT-prox-repair track	A*A*AGACTATGAAATGGAG	PS modified	NA	NA	ACACTCTTTCCCTACACGACGCTCTTCCGATCTCAGAACTGTCCTTCAGGTTC	GTGACTGGAGTTCAGACGTGTGCTCTTCCGATCTCACTGTTTCATTTCATCCGTG
4A	HPRT1	Cas9	HPRT-38087	AATTATGGGGATTACTAGGA	Alt-R Cas9 crRNA:tra	HPRT-38087-NT-prox-PAM-repair track	A*A*AGACTATGAAATGGAG	PS modified	NA	NA	ACACTCTTTCCCTACACGACGCTCTTCCGATCTCAGAACTGTCCTTCAGGTTC	GTGACTGGAGTTCAGACGTGTGCTCTTCCGATCTCACTGTTTCATTTCATCCGTG
4A	HPRT1	Cas9	HPRT-38087	AATTATGGGGATTACTAGGA	Alt-R Cas9 crRNA:tra	HPRT-38087-T-distal-none	A*G*TGCCTTGTCTGTAGTGT	PS modified	NA	NA	ACACTCTTTCCCTACACGACGCTCTTCCGATCTCAGAACTGTCCTTCAGGTTC	GTGACTGGAGTTCAGACGTGTGCTCTTCCGATCTCACTGTTTCATTTCATCCGTG
4A	HPRT1	Cas9	HPRT-38087	AATTATGGGGATTACTAGGA	Alt-R Cas9 crRNA:tra	HPRT-38087-T-distal-PAM	A*G*TGCCTTGTCTGTAGTGT	PS modified	NA	NA	ACACTCTTTCCCTACACGACGCTCTTCCGATCTCAGAACTGTCCTTCAGGTTC	GTGACTGGAGTTCAGACGTGTGCTCTTCCGATCTCACTGTTTCATTTCATCCGTG
4A	HPRT1	Cas9	HPRT-38087	AATTATGGGGATTACTAGGA	Alt-R Cas9 crRNA:tra	HPRT-38087-T-distal-repair track	A*G*TGCCTTGTCTGTAGTGT	PS modified	NA	NA	ACACTCTTTCCCTACACGACGCTCTTCCGATCTCAGAACTGTCCTTCAGGTTC	GTGACTGGAGTTCAGACGTGTGCTCTTCCGATCTCACTGTTTCATTTCATCCGTG
4A	HPRT1	Cas9	HPRT-38087	AATTATGGGGATTACTAGGA	Alt-R Cas9 crRNA:tra	HPRT-38087-T-distal-PAM-repair track	A*G*TGCCTTGTCTGTAGTGT	PS modified	NA	NA	ACACTCTTTCCCTACACGACGCTCTTCCGATCTCAGAACTGTCCTTCAGGTTC	GTGACTGGAGTTCAGACGTGTGCTCTTCCGATCTCACTGTTTCATTTCATCCGTG
4A	HPRT1	Cas9	HPRT-38087	AATTATGGGGATTACTAGGA	Alt-R Cas9 crRNA:tra	HPRT-38087-T-prox-none	C*C*TTCAGGTTCCAGGTGAT	PS modified	NA	NA	ACACTCTTTCCCTACACGACGCTCTTCCGATCTCAGAACTGTCCTTCAGGTTC	GTGACTGGAGTTCAGACGTGTGCTCTTCCGATCTCACTGTTTCATTTCATCCGTG
4A	HPRT1	Cas9	HPRT-38087	AATTATGGGGATTACTAGGA	Alt-R Cas9 crRNA:tra	HPRT-38087-T-prox-PAM	C*C*TTCAGGTTCCAGGTGAT	PS modified	NA	NA	ACACTCTTTCCCTACACGACGCTCTTCCGATCTCAGAACTGTCCTTCAGGTTC	GTGACTGGAGTTCAGACGTGTGCTCTTCCGATCTCACTGTTTCATTTCATCCGTG
4A	HPRT1	Cas9	HPRT-38087	AATTATGGGGATTACTAGGA	Alt-R Cas9 crRNA:tra	HPRT-38087-T-prox-repair track	C*C*TTCAGGTTCCAGGTGAT	PS modified	NA	NA	ACACTCTTTCCCTACACGACGCTCTTCCGATCTCAGAACTGTCCTTCAGGTTC	GTGACTGGAGTTCAGACGTGTGCTCTTCCGATCTCACTGTTTCATTTCATCCGTG
4A	HPRT1	Cas9	HPRT-38087	AATTATGGGGATTACTAGGA	Alt-R Cas9 crRNA:tra	HPRT-38087-T-prox-PAM-repair track	C*C*TTCAGGTTCCAGGTGAT	PS modified	NA	NA	ACACTCTTTCCCTACACGACGCTCTTCCGATCTCAGAACTGTCCTTCAGGTTC	GTGACTGGAGTTCAGACGTGTGCTCTTCCGATCTCACTGTTTCATTTCATCCGTG
4A	SAA1	Cas9	SAA1-88	AAGTTTTAAAAATCACTCCT	Alt-R Cas9 crRNA:tra	SAA1-88-NT-distal-none	T*T*TTTTTTAAAGGAAATGA	PS modified	NA	NA	ACACTCTTTCCCTACACGACGCTCTTCCGATCTGCTGTATGCCCCAGGATAA	GTGACTGGAGTTCAGACGTGTGCTCTTCCGATCTGACCAGCAAGGCTCACTATA
4A	SAA1	Cas9	SAA1-88	AAGTTTTAAAAATCACTCCT	Alt-R Cas9 crRNA:tra	SAA1-88-NT-distal-PAM	T*T*TTTTTTAAAGGAAATGA	PS modified	NA	NA	ACACTCTTTCCCTACACGACGCTCTTCCGATCTGCTGTATGCCCCAGGATAA	GTGACTGGAGTTCAGACGTGTGCTCTTCCGATCTGACCAGCAAGGCTCACTATA
4A	SAA1	Cas9	SAA1-88	AAGTTTTAAAAATCACTCCT	Alt-R Cas9 crRNA:tra	SAA1-88-NT-distal-repair track	T*T*TTTTTTAAAGGAAATGA	PS modified	NA	NA	ACACTCTTTCCCTACACGACGCTCTTCCGATCTGCTGTATGCCCCAGGATAA	GTGACTGGAGTTCAGACGTGTGCTCTTCCGATCTGACCAGCAAGGCTCACTATA
4A	SAA1	Cas9	SAA1-88	AAGTTTTAAAAATCACTCCT	Alt-R Cas9 crRNA:tra	SAA1-88-NT-distal-PAM-repair track	T*T*TTTTTTAAAGGAAATGA	PS modified	NA	NA	ACACTCTTTCCCTACACGACGCTCTTCCGATCTGCTGTATGCCCCAGGATAA	GTGACTGGAGTTCAGACGTGTGCTCTTCCGATCTGACCAGCAAGGCTCACTATA
4A	SAA1	Cas9	SAA1-88	AAGTTTTAAAAATCACTCCT	Alt-R Cas9 crRNA:tra	SAA1-88-NT-prox-none	T*G*TTGGGAAAGAGAAAAC	PS modified	NA	NA	ACACTCTTTCCCTACACGACGCTCTTCCGATCTGCTGTATGCCCCAGGATAA	GTGACTGGAGTTCAGACGTGTGCTCTTCCGATCTGACCAGCAAGGCTCACTATA
4A	SAA1	Cas9	SAA1-88	AAGTTTTAAAAATCACTCCT	Alt-R Cas9 crRNA:tra	SAA1-88-NT-prox-PAM	T*G*TTGGGAAAGAGAAAAC	PS modified	NA	NA	ACACTCTTTCCCTACACGACGCTCTTCCGATCTGCTGTATGCCCCAGGATAA	GTGACTGGAGTTCAGACGTGTGCTCTTCCGATCTGACCAGCAAGGCTCACTATA
4A	SAA1	Cas9	SAA1-88	AAGTTTTAAAAATCACTCCT	Alt-R Cas9 crRNA:tra	SAA1-88-NT-prox-PAM-repair track	T*G*TTGGGAAAGAGAAAAC	PS modified	NA	NA	ACACTCTTTCCCTACACGACGCTCTTCCGATCTGCTGTATGCCCCAGGATAA	GTGACTGGAGTTCAGACGTGTGCTCTTCCGATCTGACCAGCAAGGCTCACTATA
4A	SAA1	Cas9	SAA1-88	AAGTTTTAAAAATCACTCCT	Alt-R Cas9 crRNA:tra	SAA1-88-NT-prox-repair track	T*G*TTGGGAAAGAGAAAAC	PS modified	NA	NA	ACACTCTTTCCCTACACGACGCTCTTCCGATCTGCTGTATGCCCCAGGATAA	GTGACTGGAGTTCAGACGTGTGCTCTTCCGATCTGACCAGCAAGGCTCACTATA
4A	SAA1	Cas9	SAA1-88	AAGTTTTAAAAATCACTCCT	Alt-R Cas9 crRNA:tra	SAA1-88-T-distal-none	G*C*TCAGCTACAGCACAGAT	PS modified	NA	NA	ACACTCTTTCCCTACACGACGCTCTTCCGATCTGCTGTATGCCCCAGGATAA	GTGACTGGAGTTCAGACGTGTGCTCTTCCGATCTGACCAGCAAGGCTCACTATA
4A	SAA1	Cas9	SAA1-88	AAGTTTTAAAAATCACTCCT	Alt-R Cas9 crRNA:tra	SAA1-88-T-distal-PAM	G*C*TCAGCTACAGCACAGAT	PS modified	NA	NA	ACACTCTTTCCCTACACGACGCTCTTCCGATCTGCTGTATGCCCCAGGATAA	GTGACTGGAGTTCAGACGTGTGCTCTTCCGATCTGACCAGCAAGGCTCACTATA
4A	SAA1	Cas9	SAA1-88	AAGTTTTAAAAATCACTCCT	Alt-R Cas9 crRNA:tra	SAA1-88-T-distal-repair track	G*C*TCAGCTACAGCACAGAT	PS modified	NA	NA	ACACTCTTTCCCTACACGACGCTCTTCCGATCTGCTGTATGCCCCAGGATAA	GTGACTGGAGTTCAGACGTGTGCTCTTCCGATCTGACCAGCAAGGCTCACTATA
4A	SAA1	Cas9	SAA1-88	AAGTTTTAAAAATCACTCCT	Alt-R Cas9 crRNA:tra	SAA1-88-T-distal-PAM-repair track	G*C*TCAGCTACAGCACAGAT	PS modified	NA	NA	ACACTCTTTCCCTACACGACGCTCTTCCGATCTGCTGTATGCCCCAGGATAA	GTGACTGGAGTTCAGACGTGTGCTCTTCCGATCTGACCAGCAAGGCTCACTATA
4A	SAA1	Cas9	SAA1-88	AAGTTTTAAAAATCACTCCT	Alt-R Cas9 crRNA:tra	SAA1-88-T-prox-none	G*C*TCCCTGGCAGGCAGGG	PS modified	NA	NA	ACACTCTTTCCCTACACGACGCTCTTCCGATCTGCTGTATGCCCCAGGATAA	GTGACTGGAGTTCAGACGTGTGCTCTTCCGATCTGACCAGCAAGGCTCACTATA
4A	SAA1	Cas9	SAA1-88	AAGTTTTAAAAATCACTCCT	Alt-R Cas9 crRNA:tra	SAA1-88-T-prox-PAM	G*C*TCCCTGGCAGGCAGGG	PS modified	NA	NA	ACACTCTTTCCCTACACGACGCTCTTCCGATCTGCTGTATGCCCCAGGATAA	GTGACTGGAGTTCAGACGTGTGCTCTTCCGATCTGACCAGCAAGGCTCACTATA
4A	SAA1	Cas9	SAA1-88	AAGTTTTAAAAATCACTCCT	Alt-R Cas9 crRNA:tra	SAA1-88-T-prox-repair track	G*C*TCCCTGGCAGGCAGGG	PS modified	NA	NA	ACACTCTTTCCCTACACGACGCTCTTCCGATCTGCTGTATGCCCCAGGATAA	GTGACTGGAGTTCAGACGTGTGCTCTTCCGATCTGACCAGCAAGGCTCACTATA
4A	SAA1	Cas9	SAA1-88	AAGTTTTAAAAATCACTCCT	Alt-R Cas9 crRNA:tra	SAA1-88-T-prox-PAM-repair track	G*C*TCCCTGGCAGGCAGGG	PS modified	NA	NA	ACACTCTTTCCCTACACGACGCTCTTCCGATCTGCTGTATGCCCCAGGATAA	GTGACTGGAGTTCAGACGTGTGCTCTTCCGATCTGACCAGCAAGGCTCACTATA
4B	APBB2	Cas9	Hs.Cas9.APBB2.1.AA	TTGGGACAACGTTGTCCAGC	Alt-R Cas9 sgRNA	APBB2-prox-T	AAACCGACTGTGCACTGCAC	Alt-R HDR modified	NA	NA	ACACTCTTTCCCTACACGACGCTCTTCCGATCTGTGTCATCCAGCCCAGAAA	GTGACTGGAGTTCAGACGTGTGCTCTTCCGATCTGTGCCAGCTGAGGCTAC
4B	APBB2	Cas9	Hs.Cas9.APBB2.1.AA	TTGGGACAACGTTGTCCAGC	Alt-R Cas9 sgRNA	APBB2-prox-T-PAMmut	AAACCGACTGTGCACTGCAC	Alt-R HDR modified	NA	NA	ACACTCTTTCCCTACACGACGCTCTTCCGATCTGTGTCATCCAGCCCAGAAA	GTGACTGGAGTTCAGACGTGTGCTCTTCCGATCTGTGCCAGCTGAGGCTAC
4B	APBB2	Cas9	Hs.Cas9.APBB2.1.AA	TTGGGACAACGTTGTCCAGC	Alt-R Cas9 sgRNA	APBB2-prox-T-track	AAACCGACTGTGCACTGCAC	Alt-R HDR modified	NA	NA	ACACTCTTTCCCTACACGACGCTCTTCCGATCTGTGTCATCCAGCCCAGAAA	GTGACTGGAGTTCAGACGTGTGCTCTTCCGATCTGTGCCAGCTGAGGCTAC
4B	APBB2	Cas9	Hs.Cas9.APBB2.1.AA	TTGGGACAACGTTGTCCAGC	Alt-R Cas9 sgRNA	APBB2-prox-NT	GGGGAGCTGGGTGAGGCAC	Alt-R HDR modified	NA	NA	ACACTCTTTCCCTACACGACGCTCTTCCGATCTGTGTCATCCAGCCCAGAAA	GTGACTGGAGTTCAGACGTGTGCTCTTCCGATCTGTGCCAGCTGAGGCTAC
4B	APBB2	Cas9	Hs.Cas9.APBB2.1.AA	TTGGGACAACGTTGTCCAGC	Alt-R Cas9 sgRNA	APBB2-prox-NT-PAMmut	GGGGAGCTGGGTGAGGCAC	Alt-R HDR modified	NA	NA	ACACTCTTTCCCTACACGACGCTCTTCCGATCTGTGTCATCCAGCCCAGAAA	GTGACTGGAGTTCAGACGTGTGCTCTTCCGATCTGTGCCAGCTGAGGCTAC
4B	APBB2	Cas9	Hs.Cas9.APBB2.1.AA	TTGGGACAACGTTGTCCAGC	Alt-R Cas9 sgRNA	APBB2-prox-NT-track	GGGGAGCTGGGTGAGGCAC	Alt-R HDR modified	NA	NA	ACACTCTTTCCCTACACGACGCTCTTCCGATCTGTGTCATCCAGCCCAGAAA	GTGACTGGAGTTCAGACGTGTGCTCTTCCGATCTGTGCCAGCTGAGGCTAC
4B	APBB2	Cas9	Hs.Cas9.APBB2.1.AA	TTGGGACAACGTTGTCCAGC	Alt-R Cas9 sgRNA	APBB2-dist-T	CGGATCCAGAACCTGGCACC	Alt-R HDR modified	NA	NA	ACACTCTTTCCCTACACGACGCTCTTCCGATCTGTGTCATCCAGCCCAGAAA	GTGACTGGAGTTCAGACGTGTGCTCTTCCGATCTGTGCCAGCTGAGGCTAC
4B	APBB2	Cas9	Hs.Cas9.APBB2.1.AA	TTGGGACAACGTTGTCCAGC	Alt-R Cas9 sgRNA	APBB2-dist-T-track	CGGATCCAGAACCTGGCACC	Alt-R HDR modified	NA	NA	ACACTCTTTCCCTACACGACGCTCTTCCGATCTGTGTCATCCAGCCCAGAAA	GTGACTGGAGTTCAGACGTGTGCTCTTCCGATCTGTGCCAGCTGAGGCTAC
4B	APBB2	Cas9	Hs.Cas9.APBB2.1.AA	TTGGGACAACGTTGTCCAGC	Alt-R Cas9 sgRNA	APBB2-dist-NT	CCAGGGGTACCTGTTTCATCC	Alt-R HDR modified	NA	NA	ACACTCTTTCCCTACACGACGCTCTTCCGATCTGTGTCATCCAGCCCAGAAA	GTGACTGGAGTTCAGACGTGTGCTCTTCCGATCTGTGCCAGCTGAGGCTAC
4B	APBB2	Cas9	Hs.Cas9.APBB2.1.AA	TTGGGACAACGTTGTCCAGC	Alt-R Cas9 sgRNA	APBB2-dist-NT-track	CCAGGGGTACCTGTTTCATCC	Alt-R HDR modified	NA	NA	ACACTCTTTCCCTACACGACGCTCTTCCGATCTGTGTCATCCAGCCCAGAAA	GTGACTGGAGTTCAGACGTGTGCTCTTCCGATCTGTGCCAGCTGAGGCTAC
4B	APOBEC2	Cas9	Hs.Cas9.APOBEC2.1.AA	ACCAGGTGACATTGTACCGC	Alt-R Cas9 sgRNA	APOBEC2-prox-T	TGCCCATGCAGAGGAAGCTT	Alt-R HDR modified	NA	NA	ACACTCTTTCCCTACACGACGCTCTTCCGATCTTTGAAGCACAGGGCAAGG	GTGACTGGAGTTCAGACGTGTGCTCTTCCGATCTGAATGAGCAGACGCAGGTT
4B	APOBEC2	Cas9	Hs.Cas9.APOBEC2.1.AA	ACCAGGTGACATTGTACCGC	Alt-R Cas9 sgRNA	APOBEC2-prox-T-PAMmut	TGCCCATGCAGAGGAAGCTT	Alt-R HDR modified	NA	NA	ACACTCTTTCCCTACACGACGCTCTTCCGATCTTTGAAGCACAGGGCAAGG	GTGACTGGAGTTCAGACGTGTGCTCTTCCGATCTGAATGAGCAGACGCAGGTT
4B	APOBEC2	Cas9	Hs.Cas9.APOBEC2.1.AA	ACCAGGTGACATTGTACCGC	Alt-R Cas9 sgRNA	APOBEC2-prox-T-track	TGCCCATGCAGAGGAAGCTT	Alt-R HDR modified	NA	NA	ACACTCTTTCCCTACACGACGCTCTTCCGATCTTTGAAGCACAGGGCAAGG	GTGACTGGAGTTCAGACGTGTGCTCTTCCGATCTGAATGAGCAGACGCAGGTT
4B	APOBEC2	Cas9	Hs.Cas9.APOBEC2.1.AA	ACCAGGTGACATTGTACCGC	Alt-R Cas9 sgRNA	APOBEC2-prox-NT	TGCACAGGGGCTGGAGGAC	Alt-R HDR modified	NA	NA	ACACTCTTTCCCTACACGACGCTCTTCCGATCTTTGAAGCACAGGGCAAGG	GTGACTGGAGTTCAGACGTGTGCTCTTCCGATCTGAATGAGCAGACGCAGGTT
4B	APOBEC2	Cas9	Hs.Cas9.APOBEC2.1.AA	ACCAGGTGACATTGTACCGC	Alt-R Cas9 sgRNA	APOBEC2-prox-NT-PAMmut	TGCACAGGGGCTGGAGGAC	Alt-R HDR modified	NA	NA	ACACTCTTTCCCTACACGACGCTCTTCCGATCTTTGAAGCACAGGGCAAGG	GTGACTGGAGTTCAGACGTGTGCTCTTCCGATCTGAATGAGCAGACGCAGGTT
4B	APOBEC2	Cas9	Hs.Cas9.APOBEC2.1.AA	ACCAGGTGACATTGTACCGC	Alt-R Cas9 sgRNA	APOBEC2-prox-NT-track	TGCACAGGGGCTGGAGGAC	Alt-R HDR modified	NA	NA	ACACTCTTTCCCTACACGACGCTCTTCCGATCTTTGAAGCACAGGGCAAGG	GTGACTGGAGTTCAGACGTGTGCTCTTCCGATCTGAATGAGCAGACGCAGGTT
4B	APOBEC2	Cas9	Hs.Cas9.APOBEC2.1.AA	ACCAGGTGACATTGTACCGC	Alt-R Cas9 sgRNA	APOBEC2-dist-T	TCTTCAACACCATCCTGCCAG	Alt-R HDR modified	NA	NA	ACACTCTTTCCCTACACGACGCTCTTCCGATCTTTGAAGCACAGGGCAAGG	GTGACTGGAGTTCAGACGTGTGCTCTTCCGATCTGAATGAGCAGACGCAGGTT
4B	APOBEC2	Cas9	Hs.Cas9.APOBEC2.1.AA	ACCAGGTGACATTGTACCGC	Alt-R Cas9 sgRNA	APOBEC2-dist-T-track	TCTTCAACACCATCCTGCCAG	Alt-R HDR modified	NA	NA	ACACTCTTTCCCTACACGACGCTCTTCCGATCTTTGAAGCACAGGGCAAGG	GTGACTGGAGTTCAGACGTGTGCTCTTCCGATCTGAATGAGCAGACGCAGGTT
4B	APOBEC2	Cas9	Hs.Cas9.APOBEC2.1.AA	ACCAGGTGACATTGTACCGC	Alt-R Cas9 sgRNA	APOBEC2-dist-NT	ATAATGCGGTCAGCACACGC	Alt-R HDR modified	NA	NA	ACACTCTTTCCCTACACGACGCTCTTCCGATCTTTGAAGCACAGGGCAAGG	GTGACTGGAGTTCAGACGTGTGCTCTTCCGATCTGAATGAGCAGACGCAGGTT
4B	APOBEC2	Cas9	Hs.Cas9.APOBEC2.1.AA	ACCAGGTGACATTGTACCGC	Alt-R Cas9 sgRNA	APOBEC2-dist-NT-track	ATAATGCGGTCAGCACACGC	Alt-R HDR modified	NA	NA	ACACTCTTTCCCTACACGACGCTCTTCCGATCTTTGAAGCACAGGGCAAGG	GTGACTGGAGTTCAGACGTGTGCTCTTCCGATCTGAATGAGCAGACGCAGGTT
4B	APOBEC3	Cas9	Hs.Cas9.APOBEC3A.1.AA	CGGTCAAGATGGACCAGCAC	Alt-R Cas9 sgRNA	APOBEC3A-prox-T	GGGCCCTGCCCGGATTCGGA	Alt-R HDR modified	NA	NA	ACACTCTTTCCCTACACGACGCTCTTCCGATCTGATCCACACATATTCACTTCCAAC	GTGACTGGAGTTCAGACGTGTGCTCTTCCGATCTGCAGGAAACCACAAGTACAATC
4B	APOBEC3	Cas9	Hs.Cas9.APOBEC3A.1.AA	CGGTCAAGATGGACCAGCAC	Alt-R Cas9 sgRNA	APOBEC3A-prox-T-PAMmut	GGGCCCTGCCCGGATTCGGA	Alt-R HDR modified	NA	NA	ACACTCTTTCCCTACACGACGCTCTTCCGATCTGATCCACACATATTCACTTCCAAC	GTGACTGGAGTTCAGACGTGTGCTCTTCCGATCTGCAGGAAACCACAAGTACAATC
4B	APOBEC3	Cas9	Hs.Cas9.APOBEC3A.1.AA	CGGTCAAGATGGACCAGCAC	Alt-R Cas9 sgRNA	APOBEC3A-prox-T-track	GGGCCCTGCCCGGATTCGGA	Alt-R HDR modified	NA	NA	ACACTCTTTCCCTACACGACGCTCTTCCGATCTGATCCACACATATTCACTTCCAAC	GTGACTGGAGTTCAGACGTGTGCTCTTCCGATCTGCAGGAAACCACAAGTACAATC
4B	APOBEC3	Cas9	Hs.Cas9.APOBEC3A.1.AA	CGGTCAAGATGGACCAGCAC	Alt-R Cas9 sgRNA	APOBEC3A-prox-NT	GGAGCGCCTGGACAATGGCA	Alt-R HDR modified	NA	NA	ACACTCTTTCCCTACACGACGCTCTTCCGATCTGATCCACACATATTCACTTCCAAC	GTGACTGGAGTTCAGACGTGTGCTCTTCCGATCTGCAGGAAACCACAAGTACAATC
4B	APOBEC3	Cas9	Hs.Cas9.APOBEC3A.1.AA	CGGTCAAGATGGACCAGCAC	Alt-R Cas9 sgRNA	APOBEC3A-prox-NT-PAMmut	GGAGCGCCTGGACAATGGCA	Alt-R HDR modified	NA	NA	ACACTCTTTCCCTACACGACGCTCTTCCGATCTGATCCACACATATTCACTTCCAAC	GTGACTGGAGTTCAGACGTGTGCTCTTCCGATCTGCAGGAAACCACAAGTACAATC
4B	APOBEC3	Cas9	Hs.Cas9.APOBEC3A.1.AA	CGGTCAAGATGGACCAGCAC	Alt-R Cas9 sgRNA	APOBEC3A-prox-NT-track	GGAGCGCCTGGACAATGGCA	Alt-R HDR modified	NA	NA	ACACTCTTTCCCTACACGACGCTCTTCCGATCTGATCCACACATATTCACTTCCAAC	GTGACTGGAGTTCAGACGTGTGCTCTTCCGATCTGCAGGAAACCACAAGTACAATC
4B	APOBEC3	Cas9	Hs.Cas9.APOBEC3A.1.AA	CGGTCAAGATGGACCAGCAC	Alt-R Cas9 sgRNA	APOBEC3A-dist-T	TGGCTGGGTCGGTCACCTGG	Alt-R HDR modified	NA	NA	ACACTCTTTCCCTACACGACGCTCTTCCGATCTGATCCACACATATTCACTTCCAAC	GTGACTGGAGTTCAGACGTGTGCTCTTCCGATCTGCAGGAAACCACAAGTACAATC
4B	APOBEC3	Cas9	Hs.Cas9.APOBEC3A.1.AA	CGGTCAAGATGGACCAGCAC	Alt-R Cas9 sgRNA	APOBEC3A-dist-T-track	TGGCTGGGTCGGTCACCTGG	Alt-R HDR modified	NA	NA	ACACTCTTTCCCTACACGACGCTCTTCCGATCTGATCCACACATATTCACTTCCAAC	GTGACTGGAGTTCAGACGTGTGCTCTTCCGATCTGCAGGAAACCACAAGTACAATC
4B	APOBEC3	Cas9	Hs.Cas9.APOBEC3A.1.AA	CGGTCAAGATGGACCAGCAC	Alt-R Cas9 sgRNA	APOBEC3A-dist-NT	GACCTACCTGTGCTACGAAG	Alt-R HDR modified	NA	NA	ACACTCTTTCCCTACACGACGCTCTTCCGATCTGATCCACACATATTCACTTCCAAC	GTGACTGGAGTTCAGACGTGTGCTCTTCCGATCTGCAGGAAACCACAAGTACAATC
4B	APOBEC3	Cas9	Hs.Cas9.APOBEC3A.1.AA	CGGTCAAGATGGACCAGCAC	Alt-R Cas9 sgRNA	APOBEC3A-dist-NT-track	GACCTACCTGTGCTACGAAG	Alt-R HDR modified	NA	NA	ACACTCTTTCCCTACACGACGCTCTTCCGATCTGATCCACACATATTCACTTCCAAC	GTGACTGGAGTTCAGACGTGTGCTCTTCCGATCTGCAGGAAACCACAAGTACAATC
4B	APOC1	Cas9	Hs.Cas9.APOC1.1.AA	TCCAAGGCACTGGAGACGTC	Alt-R Cas9 sgRNA	APOC1-prox-T	AATTTTGAACCCCTGCCCATC	Alt-R HDR modified	NA	NA	ACACTCTTTCCCTACACGACGCTCTTCCGATCTACTCCAGCTTGGGCTACA	GTGACTGGAGTTCAGACGTGTGCTCTTCCGATCTCTAACCGCATCTTGGCAGAAA
4B	APOC1	Cas9	Hs.Cas9.APOC1.1.AA	TCCAAGGCACTGGAGACGTC	Alt-R Cas9 sgRNA	APOC1-prox-T-PAMmut	AATTTTGAACCCCTGCCCATC	Alt-R HDR modified	NA	NA	ACACTCTTTCCCTACACGACGCTCTTCCGATCTACTCCAGCTTGGGCTACA	GTGACTGGAGTTCAGACGTGTGCTCTTCCGATCTCTAACCGCATCTTGGCAGAAA
4B	APOC1	Cas9	Hs.Cas9.APOC1.1.AA	TCCAAGGCACTGGAGACGTC	Alt-R Cas9 sgRNA	APOC1-prox-T-track	AATTTTGAACCCCTGCCCATC	Alt-R HDR modified	NA	NA	ACACTCTTTCCCTACACGACGCTCTTCCGATCTACTCCAGCTTGGGCTACA	GTGACTGGAGTTCAGACGTGTGCTCTTCCGATCTCTAACCGCATCTTGGCAGAAA
4B	APOC1	Cas9	Hs.Cas9.APOC1.1.AA	TCCAAGGCACTGGAGACGTC	Alt-R Cas9 sgRNA	APOC1-prox-NT	TGTTTCCAAACTCCTTCAGCT	Alt-R HDR modified	NA	NA	ACACTCTTTCCCTACACGACGCTCTTCCGATCTACTCCAGCTTGGGCTACA	GTGACTGGAGTTCAGACGTGTGCTCTTCCGATCTCTAACCGCATCTTGGCAGAAA
4B	APOC1	Cas9	Hs.Cas9.APOC1.1.AA	TCCAAGGCACTGGAGACGTC	Alt-R Cas9 sgRNA	APOC1-prox-NT-PAMmut	TGTTTCCAAACTCCTTCAGCT	Alt-R HDR modified	NA	NA	ACACTCTTTCCCTACACGACGCTCTTCCGATCTACTCCAGCTTGGGCTACA	GTGACTGGAGTTCAGACGTGTGCTCTTCCGATCTCTAACCGCATCTTGGCAGAAA
4B	APOC1	Cas9	Hs.Cas9.APOC1.1.AA	TCCAAGGCACTGGAGACGTC	Alt-R Cas9 sgRNA	APOC1-prox-NT-track	TGTTTCCAAACTCCTTCAGCT	Alt-R HDR modified	NA	NA	ACACTCTTTCCCTACACGACGCTCTTCCGATCTACTCCAGCTTGGGCTACA	GTGACTGGAGTTCAGACGTGTGCTCTTCCGATCTCTAACCGCATCTTGGCAGAAA
4B	APOC1	Cas9	Hs.Cas9.APOC1.1.AA	TCCAAGGCACTGGAGACGTC	Alt-R Cas9 sgRNA	APOC1-dist-T	CTTCCTGGCAGGCCCAGCCCC	Alt-R HDR modified	NA	NA	ACACTCTTTCCCTACACGACGCTCTTCCGATCTACTCCAGCTTGGGCTACA	GTGACTGGAGTTCAGACGTGTGCTCTTCCGATCTCTAACCGCATCTTGGCAGAAA
4B	APOC1	Cas9	Hs.Cas9.APOC1.1.AA	TCCAAGGCACTGGAGACGTC	Alt-R Cas9 sgRNA	APOC1-dist-T-track	CTTCCTGGCAGGCCCAGCCCC	Alt-R HDR modified	NA	NA	ACACTCTTTCCCTACACGACGCTCTTCCGATCTACTCCAGCTTGGGCTACA	GTGACTGGAGTTCAGACGTGTGCTCTTCCGATCTCTAACCGCATCTTGGCAGAAA
4B	APOC1	Cas9	Hs.Cas9.APOC1.1.AA	TCCAAGGCACTGGAGACGTC	Alt-R Cas9 sgRNA	APOC1-dist-NT	CCGAGCCTTGTCCTCCAGTGT	Alt-R HDR modified	NA	NA	ACACTCTTTCCCTACACGACGCTCTTCCGATCTACTCCAGCTTGGGCTACA	GTGACTGGAGTTCAGACGTGTGCTCTTCCGATCTCTAACCGCATCTTGGCAGAAA
4B	APOC1	Cas9	Hs.Cas9.APOC1.1.AA	TCCAAGGCACTGGAGACGTC	Alt-R Cas9 sgRNA	APOC1-dist-NT-track	CCGAGCCTTGTCCTCCAGTGT	Alt-R HDR modified	NA	NA	ACACTCTTTCCCTACACGACGCTCTTCCGATCTACTCCAGCTTGGGCTACA	GTGACTGGAGTTCAGACGTGTGCTCTTCCGATCTCTAACCGCATCTTGGCAGAAA
4B	ARF5	Cas9	Hs.Cas9.ARF5.1.AA	CCCCCAACTTCAGTTTGTAC	Alt-R Cas9 sgRNA	ARF5-prox-T	CCCTGTGCCCCTTTCCGTTGC	Alt-R HDR modified	NA	NA	ACACTCTTTCCCTACACGACGCTCTTCCGATCTCTCCCTTTCCCTGGTCTCTA	GTGACTGGAGTTCAGACGTGTGCTCTTCCGATCTGCAGAACTGGGAGCAGAC
4B	ARF5	Cas9	Hs.Cas9.ARF5.1.AA	CCCCCAACTTCAGTTTGTAC	Alt-R Cas9 sgRNA	ARF5-prox-T-PAMmut	CCCTGTGCCCCTTTCCGTTGC	Alt-R HDR modified	NA	NA	ACACTCTTTCCCTACACGACGCTCTTCCGATCTCTCCCTTTCCCTGGTCTCTA	GTGACTGGAGTTCAGACGTGTGCTCTTCCGATCTGCAGAACTGGGAGCAGAC
4B	ARF5	Cas9	Hs.Cas9.ARF5.1.AA	CCCCCAACTTCAGTTTGTAC	Alt-R Cas9 sgRNA	ARF5-prox-T-track	CCCTGTGCCCCTTTCCGTTGC	Alt-R HDR modified	NA	NA	ACACTCTTTCCCTACACGACGCTCTTCCGATCTCTCCCTTTCCCTGGTCTCTA	GTGACTGGAGTTCAGACGTGTGCTCTTCCGATCTGCAGAACTGGGAGCAGAC
4B	ARF5	Cas9	Hs.Cas9.ARF5.1.AA	CCCCCAACTTCAGTTTGTAC	Alt-R Cas9 sgRNA	ARF5-prox-NT	GGTTGGGATGGTGGTGACAA	Alt-R HDR modified	NA	NA	ACACTCTTTCCCTACACGACGCTCTTCCGATCTCTCCCTTTCCCTGGTCTCTA	GTGACTGGAGTTCAGACGTGTGCTCTTCCGATCTGCAGAACTGGGAGCAGAC
4B	ARF5	Cas9	Hs.Cas9.ARF5.1.AA	CCCCCAACTTCAGTTTGTAC	Alt-R Cas9 sgRNA	ARF5-prox-NT-PAMmut	GGTTGGGATGGTGGTGACAA	Alt-R HDR modified	NA	NA	ACACTCTTTCCCTACACGACGCTCTTCCGATCTCTCCCTTTCCCTGGTCTCTA	GTGACTGGAGTTCAGACGTGTGCTCTTCCGATCTGCAGAACTGGGAGCAGAC
4B	ARF5	Cas9	Hs.Cas9.ARF5.1.AA	CCCCCAACTTCAGTTTGTAC	Alt-R Cas9 sgRNA	ARF5-prox-NT-track	GGTTGGGATGGTGGTGACAA	Alt-R HDR modified	NA	NA	ACACTCTTTCCCTACACGACGCTCTTCCGATCTCTCCCTTTCCCTGGTCTCTA	GTGACTGGAGTTCAGACGTGTGCTCTTCCGATCTGCAGAACTGGGAGCAGAC
4B	ARF5	Cas9	Hs.Cas9.ARF5.1.AA	CCCCCAACTTCAGTTTGTAC	Alt-R Cas9 sgRNA	ARF5-dist-T	CAGTTGGCTTGGATGCGGCT	Alt-R HDR modified	NA	NA	ACACTCTTTCCCTACACGACGCTCTTCCGATCTCTCCCTTTCCCTGGTCTCTA	GTGACTGGAGTTCAGACGTGTGCTCTTCCGATCTGCAGAACTGGGAGCAGAC
4B	ARF5	Cas9	Hs.Cas9.ARF5.1.AA	CCCCCAACTTCAGTTTGTAC	Alt-R Cas9 sgRNA	ARF5-dist-T-track	CAGTTGGCTTGGATGCGGCT	Alt-R HDR modified	NA	NA	ACACTCTTTCCCTACACGACGCTCTTCCGATCTCTCCCTTTCCCTGGTCTCTA	GTGACTGGAGTTCAGACGTGTGCTCTTCCGATCTGCAGAACTGGGAGCAGAC
4B	ARF5	Cas9	Hs.Cas9.ARF5.1.AA	CCCCCAACTTCAGTTTGTAC	Alt-R Cas9 sgRNA	ARF5-dist-NT	TCGTCCCCGGGCTCACCTATG	Alt-R HDR modified	NA	NA	ACACTCTTTCCCTACACGACGCTCTTCCGATCTCTCCCTTTCCCTGGTCTCTA	GTGACTGGAGTTCAGACGTGTGCTCTTCCGATCTGCAGAACTGGGAGCAGAC
4B	ARF5	Cas9	Hs.Cas9.ARF5.1.AA	CCCCCAACTTCAGTTTGTAC	Alt-R Cas9 sgRNA	ARF5-dist-NT-track	TCGTCCCCGGGCTCACCTATG	Alt-R HDR modified	NA	NA	ACACTCTTTCCCTACACGACGCTCTTCCGATCTCTCCCTTTCCCTGGTCTCTA	GTGACTGGAGTTCAGACGTGTGCTCTTCCGATCTGCAGAACTGGGAGCAGAC
4B	ARHGAP22	Cas9	Hs.Cas9.ARHGAP22.1.AA	GTCCACCACGAGCGGAAGTA	Alt-R Cas9 sgRNA	ARHGAP22-prox-T	CGCTCCCGGATGAAGTCCAC	Alt-R HDR modified	NA	NA	ACACTCTTTCCCTACACGACGCTCTTCCGATCTGTGGTGGAGGGTGTGACTA	GTGACTGGAGTTCAGACGTGTGCTCTTCCGATCTCCTGGCATGCGGAACAG
4B	ARHGAP22	Cas9	Hs.Cas9.ARHGAP22.1.AA	GTCCACCACGAGCGGAAGTA	Alt-R Cas9 sgRNA	ARHGAP22-prox-T-PAMmut	CGCTCCCGGATGAAGTCCAC	Alt-R HDR modified	NA	NA	ACACTCTTTCCCTACACGACGCTCTTCCGATCTGTGGTGGAGGGTGTGACTA	GTGACTGGAGTTCAGACGTGTGCTCTTCCGATCTCCTGGCATGCGGAACAG
4B	ARHGAP22	Cas9	Hs.Cas9.ARHGAP22.1.AA	GTCCACCACGAGCGGAAGTA	Alt-R Cas9 sgRNA	ARHGAP22-prox-T-track	CGCTCCCGGATGAAGTCCAC	Alt-R HDR modified	NA	NA	ACACTCTTTCCCTACACGACGCTCTTCCGATCTGTGGTGGAGGGTGTGACTA	GTGACTGGAGTTCAGACGTGTGCTCTTCCGATCTCCTGGCATGCGGAACAG
4B	ARHGAP22	Cas9	Hs.Cas9.ARHGAP22.1.AA	GTCCACCACGAGCGGAAGTA	Alt-R Cas9 sgRNA	ARHGAP22-prox-NT	TTGGGCAGCGCCTAGAGGAA	Alt-R HDR modified	NA	NA	ACACTCTTTCCCTACACGACGCTCTTCCGATCTGTGGTGGAGGGTGTGACTA	GTGACTGGAGTTCAGACGTGTGCTCTTCCGATCTCCTGGCATGCGGAACAG
4B	ARHGAP22	Cas9	Hs.Cas9.ARHGAP22.1.AA	GTCCACCACGAGCGGAAGTA	Alt-R Cas9 sgRNA	ARHGAP22-prox-NT-PAMmut	TTGGGCAGCGCCTAGAGGAA	Alt-R HDR modified	NA	NA	ACACTCTTTCCCTACACGACGCTCTTCCGATCTGTGGTGGAGGGTGTGACTA	GTGACTGGAGTTCAGACGTGTGCTCTTCCGATCTCCTGGCATGCGGAACAG
4B	ARHGAP22	Cas9	Hs.Cas9.ARHGAP22.1.AA	GTCCACCACGAGCGGAAGTA	Alt-R Cas9 sgRNA	ARHGAP22-prox-NT-track	TTGGGCAGCGCCTAGAGGAA	Alt-R HDR modified	NA	NA	ACACTCTTTCCCTACACGACGCTCTTCCGATCTGTGGTGGAGGGTGTGACTA	GTGACTGGAGTTCAGACGTGTGCTCTTCCGATCTCCTGGCATGCGGAACAG
4B	ARHGAP22	Cas9	Hs.Cas9.ARHGAP22.1.AA	GTCCACCACGAGCGGAAGTA	Alt-R Cas9 sgRNA	ARHGAP22-dist-T	ACACTGCTCCACCAGCAGGG	Alt-R HDR modified	NA	NA	ACACTCTTTCCCTACACGACGCTCTTCCGATCTGTGGTGGAGGGTGTGACTA	GTGACTGGAGTTCAGACGTGTGCTCTTCCGATCTCCTGGCATGCGGAACAG
4B	ARHGAP22	Cas9	Hs.Cas9.ARHGAP22.1.AA	GTCCACCACGAGCGGAAGTA	Alt-R Cas9 sgRNA	ARHGAP22-dist-T-track	ACACTGCTCCACCAGCAGGG	Alt-R HDR modified	NA	NA	ACACTCTTTCCCTACACGACGCTCTTCCGATCTGTGGTGGAGGGTGTGACTA	GTGACTGGAGTTCAGACGTGTGCTCTTCCGATCTCCTGGCATGCGGAACAG
4B	ARHGAP22	Cas9	Hs.Cas9.ARHGAP22.1.AA	GTCCACCACGAGCGGAAGTA	Alt-R Cas9 sgRNA	ARHGAP22-dist-NT	CTCTCTGTGCTCAGGGATCTT	Alt-R HDR modified	NA	NA	ACACTCTTTCCCTACACGACGCTCTTCCGATCTGTGGTGGAGGGTGTGACTA	GTGACTGGAGTTCAGACGTGTGCTCTTCCGATCTCCTGGCATGCGGAACAG
4B	ARHGAP22	Cas9	Hs.Cas9.ARHGAP22.1.AA	GTCCACCACGAGCGGAAGTA	Alt-R Cas9 sgRNA	ARHGAP22-dist-NT-track	CTCTCTGTGCTCAGGGATCTT	Alt-R HDR modified	NA	NA	ACACTCTTTCCCTACACGACGCTCTTCCGATCTGTGGTGGAGGGTGTGACTA	GTGACTGGAGTTCAGACGTGTGCTCTTCCGATCTCCTGGCATGCGGAACAG
4B	ARHGAP23	Cas9	Hs.Cas9.ARHGAP23.1.AA	CATCGTGTACCCACCCGAGT	Alt-R Cas9 sgRNA	ARHGAP23-prox-T	TCCCCAGACCAGGGCAGCGG	Alt-R HDR modified	NA	NA	ACACTCTTTCCCTACACGACGCTCTTCCGATCTAAGAGATGGGTGCTCTCCTA	GTGACTGGAGTTCAGACGTGTGCTCTTCCGATCTAAAGAGTCTCCCTCCCTCAT
4B	ARHGAP23	Cas9	Hs.Cas9.ARHGAP23.1.AA	CATCGTGTACCCACCCGAGT	Alt-R Cas9 sgRNA	ARHGAP23-prox-T-PAMmut	TCCCCAGACCAGGGCAGCGG	Alt-R HDR modified	NA	NA	ACACTCTTTCCCTACACGACGCTCTTCCGATCTAAGAGATGGGTGCTCTCCTA	GTGACTGGAGTTCAGACGTGTGCTCTTCCGATCTAAAGAGTCTCCCTCCCTCAT
4B	ARHGAP23	Cas9	Hs.Cas9.ARHGAP23.1.AA	CATCGTGTACCCACCCGAGT	Alt-R Cas9 sgRNA	ARHGAP23-prox-T-track	TCCCCAGACCAGGGCAGCGG	Alt-R HDR modified	NA	NA	ACACTCTTTCCCTACACGACGCTCTTCCGATCTAAGAGATGGGTGCTCTCCTA	GTGACTGGAGTTCAGACGTGTGCTCTTCCGATCTAAAGAGTCTCCCTCCCTCAT
4B	ARHGAP23	Cas9	Hs.Cas9.ARHGAP23.1.AA	CATCGTGTACCCACCCGAGT	Alt-R Cas9 sgRNA	ARHGAP23-prox-NT	TTTGGCTTCACTCTGCGCCAC	Alt-R HDR modified	NA	NA	ACACTCTTTCCCTACACGACGCTCTTCCGATCTAAGAGATGGGTGCTCTCCTA	GTGACTGGAGTTCAGACGTGTGCTCTTCCGATCTAAAGAGTCTCCCTCCCTCAT
4B	ARHGAP23	Cas9	Hs.Cas9.ARHGAP23.1.AA	CATCGTGTACCCACCCGAGT	Alt-R Cas9 sgRNA	ARHGAP23-prox-NT-PAMmut	TTTGGCTTCACTCTGCGCCAC	Alt-R HDR modified	NA	NA	ACACTCTTTCCCTACACGACGCTCTTCCGATCTAAGAGATGGGTGCTCTCCTA	GTGACTGGAGTTCAGACGTGTGCTCTTCCGATCTAAAGAGTCTCCCTCCCTCAT
4B	ARHGAP23	Cas9	Hs.Cas9.ARHGAP23.1.AA	CATCGTGTACCCACCCGAGT	Alt-R Cas9 sgRNA	ARHGAP23-prox-NT-track	TTTGGCTTCACTCTGCGCCAC	Alt-R HDR modified	NA	NA	ACACTCTTTCCCTACACGACGCTCTTCCGATCTAAGAGATGGGTGCTCTCCTA	GTGACTGGAGTTCAGACGTGTGCTCTTCCGATCTAAAGAGTCTCCCTCCCTCAT
4B	ARHGAP23	Cas9	Hs.Cas9.ARHGAP23.1.AA	CATCGTGTACCCACCCGAGT	Alt-R Cas9 sgRNA	ARHGAP23-dist-T	CGAGCCGGGCATACCTTCAG	Alt-R HDR modified	NA	NA	ACACTCTTTCCCTACACGACGCTCTTCCGATCTAAGAGATGGGTGCTCTCCTA	GTGACTGGAGTTCAGACGTGTGCTCTTCCGATCTAAAGAGTCTCCCTCCCTCAT
4B	ARHGAP23	Cas9	Hs.Cas9.ARHGAP23.1.AA	CATCGTGTACCCACCCGAGT	Alt-R Cas9 sgRNA	ARHGAP23-dist-T-track	CGAGCCGGGCATACCTTCAG	Alt-R HDR modified	NA	NA	ACACTCTTTCCCTACACGACGCTCTTCCGATCTAAGAGATGGGTGCTCTCCTA	GTGACTGGAGTTCAGACGTGTGCTCTTCCGATCTAAAGAGTCTCCCTCCCTCAT
4B	ARHGAP23	Cas9	Hs.Cas9.ARHGAP23.1.AA	CATCGTGTACCCACCCGAGT	Alt-R Cas9 sgRNA	ARHGAP23-dist-NT	TACAAAAGTCCCCAGGACGG	Alt-R HDR modified	NA	NA	ACACTCTTTCCCTACACGACGCTCTTCCGATCTAAGAGATGGGTGCTCTCCTA	GTGACTGGAGTTCAGACGTGTGCTCTTCCGATCTAAAGAGTCTCCCTCCCTCAT
4B	ARHGAP23	Cas9	Hs.Cas9.ARHGAP23.1.AA	CATCGTGTACCCACCCGAGT	Alt-R Cas9 sgRNA	ARHGAP23-dist-NT-track	TACAAAAGTCCCCAGGACGG	Alt-R HDR modified	NA	NA	ACACTCTTTCCCTACACGACGCTCTTCCGATCTAAGAGATGGGTGCTCTCCTA	GTGACTGGAGTTCAGACGTGTGCTCTTCCGATCTAAAGAGTCTCCCTCCCTCAT
4B	ARHGAP24	Cas9	Hs.Cas9.ARHGAP24.1.AA	TCAAGACTTGGCATACTCGC	Alt-R Cas9 sgRNA	ARHGAP24-prox-T	CTTGGTTTCATCTTCATCTTTG	Alt-R HDR modified	NA	NA	ACACTCTTTCCCTACACGACGCTCTTCCGATCTACAATGACTCCACGGAGAAC	GTGACTGGAGTTCAGACGTGTGCTCTTCCGATCTTGGCTTTCTTAGGCCCTTAAT
4B	ARHGAP24	Cas9	Hs.Cas9.ARHGAP24.1.AA	TCAAGACTTGGCATACTCGC	Alt-R Cas9 sgRNA	ARHGAP24-prox-T-PAMmut	CTTGGTTTCATCTTCATCTTTG	Alt-R HDR modified	NA	NA	ACACTCTTTCCCTACACGACGCTCTTCCGATCTACAATGACTCCACGGAGAAC	GTGACTGGAGTTCAGACGTGTGCTCTTCCGATCTTGGCTTTCTTAGGCCCTTAAT
4B	ARHGAP24	Cas9	Hs.Cas9.ARHGAP24.1.AA	TCAAGACTTGGCATACTCGC	Alt-R Cas9 sgRNA	ARHGAP24-prox-T-track	CTTGGTTTCATCTTCATCTTTG	Alt-R HDR modified	NA	NA	ACACTCTTTCCCTACACGACGCTCTTCCGATCTACAATGACTCCACGGAGAAC	GTGACTGGAGTTCAGACGTGTGCTCTTCCGATCTTGGCTTTCTTAGGCCCTTAAT
4B	ARHGAP24	Cas9	Hs.Cas9.ARHGAP24.1.AA	TCAAGACTTGGCATACTCGC	Alt-R Cas9 sgRNA	ARHGAP24-prox-NT	GCTGAGGAAGCAAGGAGGC	Alt-R HDR modified	NA	NA	ACACTCTTTCCCTACACGACGCTCTTCCGATCTACAATGACTCCACGGAGAAC	GTGACTGGAGTTCAGACGTGTGCTCTTCCGATCTTGGCTTTCTTAGGCCCTTAAT
4B	ARHGAP24	Cas9	Hs.Cas9.ARHGAP24.1.AA	TCAAGACTTGGCATACTCGC	Alt-R Cas9 sgRNA	ARHGAP24-prox-NT-PAMmut	GCTGAGGAAGCAAGGAGGC	Alt-R HDR modified	NA	NA	ACACTCTTTCCCTACACGACGCTCTTCCGATCTACAATGACTCCACGGAGAAC	GTGACTGGAGTTCAGACGTGTGCTCTTCCGATCTTGGCTTTCTTAGGCCCTTAAT
4B	ARHGAP24	Cas9	Hs.Cas9.ARHGAP24.1.AA	TCAAGACTTGGCATACTCGC	Alt-R Cas9 sgRNA	ARHGAP24-prox-NT-track	GCTGAGGAAGCAAGGAGGC	Alt-R HDR modified	NA	NA	ACACTCTTTCCCTACACGACGCTCTTCCGATCTACAATGACTCCACGGAGAAC	GTGACTGGAGTTCAGACGTGTGCTCTTCCGATCTTGGCTTTCTTAGGCCCTTAAT
4B	ARHGAP24	Cas9	Hs.Cas9.ARHGAP24.1.AA	TCAAGACTTGGCATACTCGC	Alt-R Cas9 sgRNA	ARHGAP24-dist-T	TGAAATAATAGAGCTGATCC	Alt-R HDR modified	NA	NA	ACACTCTTTCCCTACACGACGCTCTTCCGATCTACAATGACTCCACGGAGAAC	GTGACTGGAGTTCAGACGTGTGCTCTTCCGATCTTGGCTTTCTTAGGCCCTTAAT
4B	ARHGAP24	Cas9	Hs.Cas9.ARHGAP24.1.AA	TCAAGACTTGGCATACTCGC	Alt-R Cas9 sgRNA	ARHGAP24-dist-T-track	TGAAATAATAGAGCTGATCC	Alt-R HDR modified	NA	NA	ACACTCTTTCCCTACACGACGCTCTTCCGATCTACAATGACTCCACGGAGAAC	GTGACTGGAGTTCAGACGTGTGCTCTTCCGATCTTGGCTTTCTTAGGCCCTTAAT
4B	ARHGAP24	Cas9	Hs.Cas9.ARHGAP24.1.AA	TCAAGACTTGGCATACTCGC	Alt-R Cas9 sgRNA	ARHGAP24-dist-NT	GAATGCCATCAAGTGTGGGT	Alt-R HDR modified	NA	NA	ACACTCTTTCCCTACACGACGCTCTTCCGATCTACAATGACTCCACGGAGAAC	GTGACTGGAGTTCAGACGTGTGCTCTTCCGATCTTGGCTTTCTTAGGCCCTTAAT
4B	ARHGAP24	Cas9	Hs.Cas9.ARHGAP24.1.AA	TCAAGACTTGGCATACTCGC	Alt-R Cas9 sgRNA	ARHGAP24-dist-NT-track	GAATGCCATCAAGTGTGGGT	Alt-R HDR modified	NA	NA	ACACTCTTTCCCTACACGACGCTCTTCCGATCTACAATGACTCCACGGAGAAC	GTGACTGGAGTTCAGACGTGTGCTCTTCCGATCTTGGCTTTCTTAGGCCCTTAAT
4B	ARHGAP36	Cas9	Hs.Cas9.ARHGAP36.1.AA	AAGGTGTTCGGACGGTCAAT	Alt-R Cas9 sgRNA	ARHGAP36-prox-T	TATTAAGTCCTATTTGTAAGT	Alt-R HDR modified	NA	NA	ACACTCTTTCCCTACACGACGCTCTTCCGATCTGATCAGGGCATTGGGATGTT	GTGACTGGAGTTCAGACGTGTGCTCTTCCGATCTATAGCCAGGGCCACACTA
4B	ARHGAP36	Cas9	Hs.Cas9.ARHGAP36.1.AA	AAGGTGTTCGGACGGTCAAT	Alt-R Cas9 sgRNA	ARHGAP36-prox-T-PAMmut	TATTAAGTCCTATTTGTAAGT	Alt-R HDR modified	NA	NA	ACACTCTTTCCCTACACGACGCTCTTCCGATCTGATCAGGGCATTGGGATGTT	GTGACTGGAGTTCAGACGTGTGCTCTTCCGATCTATAGCCAGGGCCACACTA
4B	ARHGAP36	Cas9	Hs.Cas9.ARHGAP36.1.AA	AAGGTGTTCGGACGGTCAAT	Alt-R Cas9 sgRNA	ARHGAP36-prox-T-track	TATTAAGTCCTATTTGTAAGT	Alt-R HDR modified	NA	NA	ACACTCTTTCCCTACACGACGCTCTTCCGATCTGATCAGGGCATTGGGATGTT	GTGACTGGAGTTCAGACGTGTGCTCTTCCGATCTATAGCCAGGGCCACACTA
4B	ARHGAP36	Cas9	Hs.Cas9.ARHGAP36.1.AA	AAGGTGTTCGGACGGTCAAT	Alt-R Cas9 sgRNA	ARHGAP36-prox-NT	TCAAAATCAGAAACCACTTAT	Alt-R HDR modified	NA	NA	ACACTCTTTCCCTACACGACGCTCTTCCGATCTGATCAGGGCATTGGGATGTT	GTGACTGGAGTTCAGACGTGTGCTCTTCCGATCTATAGCCAGGGCCACACTA
4B	ARHGAP36	Cas9	Hs.Cas9.ARHGAP36.1.AA	AAGGTGTTCGGACGGTCAAT	Alt-R Cas9 sgRNA	ARHGAP36-prox-NT-PAMmut	TCAAAATCAGAAACCACTTAT	Alt-R HDR modified	NA	NA	ACACTCTTTCCCTACACGACGCTCTTCCGATCTGATCAGGGCATTGGGATGTT	GTGACTGGAGTTCAGACGTGTGCTCTTCCGATCTATAGCCAGGGCCACACTA
4B	ARHGAP36	Cas9	Hs.Cas9.ARHGAP36.1.AA	AAGGTGTTCGGACGGTCAAT	Alt-R Cas9 sgRNA	ARHGAP36-prox-NT-track	TCAAAATCAGAAACCACTTAT	Alt-R HDR modified	NA	NA	ACACTCTTTCCCTACACGACGCTCTTCCGATCTGATCAGGGCATTGGGATGTT	GTGACTGGAGTTCAGACGTGTGCTCTTCCGATCTATAGCCAGGGCCACACTA
4B	ARHGAP36	Cas9	Hs.Cas9.ARHGAP36.1.AA	AAGGTGTTCGGACGGTCAAT	Alt-R Cas9 sgRNA	ARHGAP36-dist-T	TGTATCTTTTCTTTTTTCTCTT	Alt-R HDR modified	NA	NA	ACACTCTTTCCCTACACGACGCTCTTCCGATCTGATCAGGGCATTGGGATGTT	GTGACTGGAGTTCAGACGTGTGCTCTTCCGATCTATAGCCAGGGCCACACTA
4B	ARHGAP36	Cas9	Hs.Cas9.ARHGAP36.1.AA	AAGGTGTTCGGACGGTCAAT	Alt-R Cas9 sgRNA	ARHGAP36-dist-T-track	TGTATCTTTTCTTTTTTCTCTT	Alt-R HDR modified	NA	NA	ACACTCTTTCCCTACACGACGCTCTTCCGATCTGATCAGGGCATTGGGATGTT	GTGACTGGAGTTCAGACGTGTGCTCTTCCGATCTATAGCCAGGGCCACACTA
4B	ARHGAP36	Cas9	Hs.Cas9.ARHGAP36.1.AA	AAGGTGTTCGGACGGTCAAT	Alt-R Cas9 sgRNA	ARHGAP36-dist-NT	CTCACCCCTCTGCTGTCCTCTC	Alt-R HDR modified	NA	NA	ACACTCTTTCCCTACACGACGCTCTTCCGATCTGATCAGGGCATTGGGATGTT	GTGACTGGAGTTCAGACGTGTGCTCTTCCGATCTATAGCCAGGGCCACACTA
4B	ARHGAP36	Cas9	Hs.Cas9.ARHGAP36.1.AA	AAGGTGTTCGGACGGTCAAT	Alt-R Cas9 sgRNA	ARHGAP36-dist-NT-track	CTCACCCCTCTGCTGTCCTCTC	Alt-R HDR modified	NA	NA	ACACTCTTTCCCTACACGACGCTCTTCCGATCTGATCAGGGCATTGGGATGTT	GTGACTGGAGTTCAGACGTGTGCTCTTCCGATCTATAGCCAGGGCCACACTA
4B	ARHGAP6	Cas9	Hs.Cas9.ARHGAP6.1.AA	TCGGATTTCCACACGACGTA	Alt-R Cas9 sgRNA	ARHGAP6-prox-T	AGGAGGTGGCTCCAGCAGAG	Alt-R HDR modified	NA	NA	ACACTCTTTCCCTACACGACGCTCTTCCGATCTTTCCAGCATCCTCTGTTCATC	GTGACTGGAGTTCAGACGTGTGCTCTTCCGATCTGCCTTGCTCTGTAAATGTAATGG
4B	ARHGAP6	Cas9	Hs.Cas9.ARHGAP6.1.AA	TCGGATTTCCACACGACGTA	Alt-R Cas9 sgRNA	ARHGAP6-prox-T-PAMmut	AGGAGGTGGCTCCAGCAGAG	Alt-R HDR modified	NA	NA	ACACTCTTTCCCTACACGACGCTCTTCCGATCTTTCCAGCATCCTCTGTTCATC	GTGACTGGAGTTCAGACGTGTGCTCTTCCGATCTGCCTTGCTCTGTAAATGTAATGG
4B	ARHGAP6	Cas9	Hs.Cas9.ARHGAP6.1.AA	TCGGATTTCCACACGACGTA	Alt-R Cas9 sgRNA	ARHGAP6-prox-T-track	AGGAGGTGGCTCCAGCAGAG	Alt-R HDR modified	NA	NA	ACACTCTTTCCCTACACGACGCTCTTCCGATCTTTCCAGCATCCTCTGTTCATC	GTGACTGGAGTTCAGACGTGTGCTCTTCCGATCTGCCTTGCTCTGTAAATGTAATGG
4B	ARHGAP6	Cas9	Hs.Cas9.ARHGAP6.1.AA	TCGGATTTCCACACGACGTA	Alt-R Cas9 sgRNA	ARHGAP6-prox-NT	GACGCGCAGCGCTGGTCCCT	Alt-R HDR modified	NA	NA	ACACTCTTTCCCTACACGACGCTCTTCCGATCTTTCCAGCATCCTCTGTTCATC	GTGACTGGAGTTCAGACGTGTGCTCTTCCGATCTGCCTTGCTCTGTAAATGTAATGG
4B	ARHGAP6	Cas9	Hs.Cas9.ARHGAP6.1.AA	TCGGATTTCCACACGACGTA	Alt-R Cas9 sgRNA	ARHGAP6-prox-NT-PAMmut	GACGCGCAGCGCTGGTCCCT	Alt-R HDR modified	NA	NA	ACACTCTTTCCCTACACGACGCTCTTCCGATCTTTCCAGCATCCTCTGTTCATC	GTGACTGGAGTTCAGACGTGTGCTCTTCCGATCTGCCTTGCTCTGTAAATGTAATGG
4B	ARHGAP6	Cas9	Hs.Cas9.ARHGAP6.1.AA	TCGGATTTCCACACGACGTA	Alt-R Cas9 sgRNA	ARHGAP6-prox-NT-track	GACGCGCAGCGCTGGTCCCT	Alt-R HDR modified	NA	NA	ACACTCTTTCCCTACACGACGCTCTTCCGATCTTTCCAGCATCCTCTGTTCATC	GTGACTGGAGTTCAGACGTGTGCTCTTCCGATCTGCCTTGCTCTGTAAATGTAATGG
4B	ARHGAP6	Cas9	Hs.Cas9.ARHGAP6.1.AA	TCGGATTTCCACACGACGTA	Alt-R Cas9 sgRNA	ARHGAP6-dist-T	GAAGTTCCAGTCCCCACCCGA	Alt-R HDR modified	NA	NA	ACACTCTTTCCCTACACGACGCTCTTCCGATCTTTCCAGCATCCTCTGTTCATC	GTGACTGGAGTTCAGACGTGTGCTCTTCCGATCTGCCTTGCTCTGTAAATGTAATGG
4B	ARHGAP6	Cas9	Hs.Cas9.ARHGAP6.1.AA	TCGGATTTCCACACGACGTA	Alt-R Cas9 sgRNA	ARHGAP6-dist-T-track	GAAGTTCCAGTCCCCACCCGA	Alt-R HDR modified	NA	NA	ACACTCTTTCCCTACACGACGCTCTTCCGATCTTTCCAGCATCCTCTGTTCATC	GTGACTGGAGTTCAGACGTGTGCTCTTCCGATCTGCCTTGCTCTGTAAATGTAATGG
4B	ARHGAP6	Cas9	Hs.Cas9.ARHGAP6.1.AA	TCGGATTTCCACACGACGTA	Alt-R Cas9 sgRNA	ARHGAP6-dist-NT	GGCCTCCTCCTTGGGCCGTG	Alt-R HDR modified	NA	NA	ACACTCTTTCCCTACACGACGCTCTTCCGATCTTTCCAGCATCCTCTGTTCATC	GTGACTGGAGTTCAGACGTGTGCTCTTCCGATCTGCCTTGCTCTGTAAATGTAATGG
4B	ARHGAP6	Cas9	Hs.Cas9.ARHGAP6.1.AA	TCGGATTTCCACACGACGTA	Alt-R Cas9 sgRNA	ARHGAP6-dist-NT-track	GGCCTCCTCCTTGGGCCGTG	Alt-R HDR modified	NA	NA	ACACTCTTTCCCTACACGACGCTCTTCCGATCTTTCCAGCATCCTCTGTTCATC	GTGACTGGAGTTCAGACGTGTGCTCTTCCGATCTGCCTTGCTCTGTAAATGTAATGG
4B	ARHGEF33	Cas9	Hs.Cas9.ARHGEF33.1.AA	GATGGCTTGGATCATCGACA	Alt-R Cas9 sgRNA	ARHGEF33-prox-T	ATGTACCACGTTAAAACATAC	Alt-R HDR modified	NA	NA	ACACTCTTTCCCTACACGACGCTCTTCCGATCTAGTGTACAGTGTGCTAGATTATGTT	GTGACTGGAGTTCAGACGTGTGCTCTTCCGATCTGATTCTCTTCGCTTCTCCTGTT
4B	ARHGEF33	Cas9	Hs.Cas9.ARHGEF33.1.AA	GATGGCTTGGATCATCGACA	Alt-R Cas9 sgRNA	ARHGEF33-prox-T-PAMmut	ATGTACCACGTTAAAACATAC	Alt-R HDR modified	NA	NA	ACACTCTTTCCCTACACGACGCTCTTCCGATCTAGTGTACAGTGTGCTAGATTATGTT	GTGACTGGAGTTCAGACGTGTGCTCTTCCGATCTGATTCTCTTCGCTTCTCCTGTT
4B	ARHGEF33	Cas9	Hs.Cas9.ARHGEF33.1.AA	GATGGCTTGGATCATCGACA	Alt-R Cas9 sgRNA	ARHGEF33-prox-T-track	ATGTACCACGTTAAAACATAC	Alt-R HDR modified	NA	NA	ACACTCTTTCCCTACACGACGCTCTTCCGATCTAGTGTACAGTGTGCTAGATTATGTT	GTGACTGGAGTTCAGACGTGTGCTCTTCCGATCTGATTCTCTTCGCTTCTCCTGTT
4B	ARHGEF33	Cas9	Hs.Cas9.ARHGEF33.1.AA	GATGGCTTGGATCATCGACA	Alt-R Cas9 sgRNA	ARHGEF33-prox-NT	TGCATTTCTTCTTGTTTGGAA	Alt-R HDR modified	NA	NA	ACACTCTTTCCCTACACGACGCTCTTCCGATCTAGTGTACAGTGTGCTAGATTATGTT	GTGACTGGAGTTCAGACGTGTGCTCTTCCGATCTGATTCTCTTCGCTTCTCCTGTT

4B	ARHGEF33	Cas9	Hs.Cas9.ARHGEF33.1.AA	GATGGCTTGGATCATCGACA	Alt-R Cas9 sgRNA	ARHGEF33-prox-NT-PAMmut	TGCATTTCTTCTTGTTTGGAA	Alt-R HDR modified	NA	NA	ACACTCTTTCCCTACACGACGCTCTTCCGATCTAGTGTACAGTGTGCTAGATTATGTT	GTGACTGGAGTTCAGACGTGTGCTCTTCCGATCTGATTCTCTTCGCTTCTCCTGTT
4B	ARHGEF33	Cas9	Hs.Cas9.ARHGEF33.1.AA	GATGGCTTGGATCATCGACA	Alt-R Cas9 sgRNA	ARHGEF33-prox-NT-track	TGCATTTCTTCTTGTTTGGAA	Alt-R HDR modified	NA	NA	ACACTCTTTCCCTACACGACGCTCTTCCGATCTAGTGTACAGTGTGCTAGATTATGTT	GTGACTGGAGTTCAGACGTGTGCTCTTCCGATCTGATTCTCTTCGCTTCTCCTGTT
4B	ARHGEF33	Cas9	Hs.Cas9.ARHGEF33.1.AA	GATGGCTTGGATCATCGACA	Alt-R Cas9 sgRNA	ARHGEF33-dist-T	GCAATTCTTATTTTTTAGGAA	Alt-R HDR modified	NA	NA	ACACTCTTTCCCTACACGACGCTCTTCCGATCTAGTGTACAGTGTGCTAGATTATGTT	GTGACTGGAGTTCAGACGTGTGCTCTTCCGATCTGATTCTCTTCGCTTCTCCTGTT
4B	ARHGEF33	Cas9	Hs.Cas9.ARHGEF33.1.AA	GATGGCTTGGATCATCGACA	Alt-R Cas9 sgRNA	ARHGEF33-dist-T-track	GCAATTCTTATTTTTTAGGAA	Alt-R HDR modified	NA	NA	ACACTCTTTCCCTACACGACGCTCTTCCGATCTAGTGTACAGTGTGCTAGATTATGTT	GTGACTGGAGTTCAGACGTGTGCTCTTCCGATCTGATTCTCTTCGCTTCTCCTGTT
4B	ARHGEF33	Cas9	Hs.Cas9.ARHGEF33.1.AA	GATGGCTTGGATCATCGACA	Alt-R Cas9 sgRNA	ARHGEF33-dist-NT	GAAGCTGCTCGATTTTCTGTT	Alt-R HDR modified	NA	NA	ACACTCTTTCCCTACACGACGCTCTTCCGATCTAGTGTACAGTGTGCTAGATTATGTT	GTGACTGGAGTTCAGACGTGTGCTCTTCCGATCTGATTCTCTTCGCTTCTCCTGTT
4B	ARHGEF33	Cas9	Hs.Cas9.ARHGEF33.1.AA	GATGGCTTGGATCATCGACA	Alt-R Cas9 sgRNA	ARHGEF33-dist-NT-track	GAAGCTGCTCGATTTTCTGTT	Alt-R HDR modified	NA	NA	ACACTCTTTCCCTACACGACGCTCTTCCGATCTAGTGTACAGTGTGCTAGATTATGTT	GTGACTGGAGTTCAGACGTGTGCTCTTCCGATCTGATTCTCTTCGCTTCTCCTGTT
4B	ARID1B	Cas9	Hs.Cas9.ARID1B.1.AA	TGCCAATTGGATACCGCTGT	Alt-R Cas9 sgRNA	ARID1B-prox-T	TTCTCAGCCTCAGCAGAGCAC	Alt-R HDR modified	NA	NA	ACACTCTTTCCCTACACGACGCTCTTCCGATCTTTGTATGGCATGGGCAGTAA	GTGACTGGAGTTCAGACGTGTGCTCTTCCGATCTGGGACCAGCACAAACCT
4B	ARID1B	Cas9	Hs.Cas9.ARID1B.1.AA	TGCCAATTGGATACCGCTGT	Alt-R Cas9 sgRNA	ARID1B-prox-T-PAMmut	TTCTCAGCCTCAGCAGAGCAC	Alt-R HDR modified	NA	NA	ACACTCTTTCCCTACACGACGCTCTTCCGATCTTTGTATGGCATGGGCAGTAA	GTGACTGGAGTTCAGACGTGTGCTCTTCCGATCTGGGACCAGCACAAACCT
4B	ARID1B	Cas9	Hs.Cas9.ARID1B.1.AA	TGCCAATTGGATACCGCTGT	Alt-R Cas9 sgRNA	ARID1B-prox-T-track	TTCTCAGCCTCAGCAGAGCAC	Alt-R HDR modified	NA	NA	ACACTCTTTCCCTACACGACGCTCTTCCGATCTTTGTATGGCATGGGCAGTAA	GTGACTGGAGTTCAGACGTGTGCTCTTCCGATCTGGGACCAGCACAAACCT
4B	ARID1B	Cas9	Hs.Cas9.ARID1B.1.AA	TGCCAATTGGATACCGCTGT	Alt-R Cas9 sgRNA	ARID1B-prox-NT	GGCCCCGGGAGTCCGACCCT	Alt-R HDR modified	NA	NA	ACACTCTTTCCCTACACGACGCTCTTCCGATCTTTGTATGGCATGGGCAGTAA	GTGACTGGAGTTCAGACGTGTGCTCTTCCGATCTGGGACCAGCACAAACCT
4B	ARID1B	Cas9	Hs.Cas9.ARID1B.1.AA	TGCCAATTGGATACCGCTGT	Alt-R Cas9 sgRNA	ARID1B-prox-NT-PAMmut	GGCCCCGGGAGTCCGACCCT	Alt-R HDR modified	NA	NA	ACACTCTTTCCCTACACGACGCTCTTCCGATCTTTGTATGGCATGGGCAGTAA	GTGACTGGAGTTCAGACGTGTGCTCTTCCGATCTGGGACCAGCACAAACCT
4B	ARID1B	Cas9	Hs.Cas9.ARID1B.1.AA	TGCCAATTGGATACCGCTGT	Alt-R Cas9 sgRNA	ARID1B-prox-NT-track	GGCCCCGGGAGTCCGACCCT	Alt-R HDR modified	NA	NA	ACACTCTTTCCCTACACGACGCTCTTCCGATCTTTGTATGGCATGGGCAGTAA	GTGACTGGAGTTCAGACGTGTGCTCTTCCGATCTGGGACCAGCACAAACCT
4B	ARID1B	Cas9	Hs.Cas9.ARID1B.1.AA	TGCCAATTGGATACCGCTGT	Alt-R Cas9 sgRNA	ARID1B-dist-T	GTCCGTACCCAGGAGGTTCCT	Alt-R HDR modified	NA	NA	ACACTCTTTCCCTACACGACGCTCTTCCGATCTTTGTATGGCATGGGCAGTAA	GTGACTGGAGTTCAGACGTGTGCTCTTCCGATCTGGGACCAGCACAAACCT
4B	ARID1B	Cas9	Hs.Cas9.ARID1B.1.AA	TGCCAATTGGATACCGCTGT	Alt-R Cas9 sgRNA	ARID1B-dist-T-track	GTCCGTACCCAGGAGGTTCCT	Alt-R HDR modified	NA	NA	ACACTCTTTCCCTACACGACGCTCTTCCGATCTTTGTATGGCATGGGCAGTAA	GTGACTGGAGTTCAGACGTGTGCTCTTCCGATCTGGGACCAGCACAAACCT
4B	ARID1B	Cas9	Hs.Cas9.ARID1B.1.AA	TGCCAATTGGATACCGCTGT	Alt-R Cas9 sgRNA	ARID1B-dist-NT	GGGTACTGCATTCCGGCCATC	Alt-R HDR modified	NA	NA	ACACTCTTTCCCTACACGACGCTCTTCCGATCTTTGTATGGCATGGGCAGTAA	GTGACTGGAGTTCAGACGTGTGCTCTTCCGATCTGGGACCAGCACAAACCT
4B	ARID1B	Cas9	Hs.Cas9.ARID1B.1.AA	TGCCAATTGGATACCGCTGT	Alt-R Cas9 sgRNA	ARID1B-dist-NT-track	GGGTACTGCATTCCGGCCATC	Alt-R HDR modified	NA	NA	ACACTCTTTCCCTACACGACGCTCTTCCGATCTTTGTATGGCATGGGCAGTAA	GTGACTGGAGTTCAGACGTGTGCTCTTCCGATCTGGGACCAGCACAAACCT
5B	HPRT	Cas12a	HPRT-38330-AS	GGTTAAAGATGGTTAAATGAT	Alt-R Cas12a crRNA	HPRT-38330 EcoRI 0	CATTTCACATAAAACTCTTTT	Unmodified	AAGAATGTTGTGATAAAAGGTGATGCT	ACACATCCATGGGACTTCTGCCTC	NA	NA
5B	HPRT	Cas12a	HPRT-38330-AS	GGTTAAAGATGGTTAAATGAT	Alt-R Cas12a crRNA	HPRT-38330 EcoRI +2	CATTTCACATAAAACTCTTTT	Unmodified	AAGAATGTTGTGATAAAAGGTGATGCT	ACACATCCATGGGACTTCTGCCTC	NA	NA
5B	HPRT	Cas12a	HPRT-38330-AS	GGTTAAAGATGGTTAAATGAT	Alt-R Cas12a crRNA	HPRT-38330 EcoRI +4	CATTTCACATAAAACTCTTTT	Unmodified	AAGAATGTTGTGATAAAAGGTGATGCT	ACACATCCATGGGACTTCTGCCTC	NA	NA
5B	HPRT	Cas12a	HPRT-38330-AS	GGTTAAAGATGGTTAAATGAT	Alt-R Cas12a crRNA	HPRT-38330 EcoRI +6	CATTTCACATAAAACTCTTTT	Unmodified	AAGAATGTTGTGATAAAAGGTGATGCT	ACACATCCATGGGACTTCTGCCTC	NA	NA
5B	HPRT	Cas12a	HPRT-38330-AS	GGTTAAAGATGGTTAAATGAT	Alt-R Cas12a crRNA	HPRT-38330 EcoRI +10	CATTTCACATAAAACTCTTTT	Unmodified	AAGAATGTTGTGATAAAAGGTGATGCT	ACACATCCATGGGACTTCTGCCTC	NA	NA
5B	HPRT	Cas12a	HPRT-38330-AS	GGTTAAAGATGGTTAAATGAT	Alt-R Cas12a crRNA	HPRT-38330 EcoRI +15	CATTTCACATAAAACTCTTTT	Unmodified	AAGAATGTTGTGATAAAAGGTGATGCT	ACACATCCATGGGACTTCTGCCTC	NA	NA
5B	HPRT	Cas12a	HPRT-38330-AS	GGTTAAAGATGGTTAAATGAT	Alt-R Cas12a crRNA	HPRT-38330 EcoRI +21	CATTTCACATAAAACTCTTTT	Unmodified	AAGAATGTTGTGATAAAAGGTGATGCT	ACACATCCATGGGACTTCTGCCTC	NA	NA
5B	HPRT	Cas12a	HPRT-38330-AS	GGTTAAAGATGGTTAAATGAT	Alt-R Cas12a crRNA	HPRT-38330 EcoRI +27	CATTTCACATAAAACTCTTTT	Unmodified	AAGAATGTTGTGATAAAAGGTGATGCT	ACACATCCATGGGACTTCTGCCTC	NA	NA
5B	HPRT	Cas12a	HPRT-38330-AS	GGTTAAAGATGGTTAAATGAT	Alt-R Cas12a crRNA	HPRT-38330 EcoRI -2	CATTTCACATAAAACTCTTTT	Unmodified	AAGAATGTTGTGATAAAAGGTGATGCT	ACACATCCATGGGACTTCTGCCTC	NA	NA
5B	HPRT	Cas12a	HPRT-38330-AS	GGTTAAAGATGGTTAAATGAT	Alt-R Cas12a crRNA	HPRT-38330 EcoRI -4	CATTTCACATAAAACTCTTTT	Unmodified	AAGAATGTTGTGATAAAAGGTGATGCT	ACACATCCATGGGACTTCTGCCTC	NA	NA
5B	HPRT	Cas12a	HPRT-38330-AS	GGTTAAAGATGGTTAAATGAT	Alt-R Cas12a crRNA	HPRT-38330 EcoRI -6	CATTTCACATAAAACTCTTTT	Unmodified	AAGAATGTTGTGATAAAAGGTGATGCT	ACACATCCATGGGACTTCTGCCTC	NA	NA
5B	HPRT	Cas12a	HPRT-38330-AS	GGTTAAAGATGGTTAAATGAT	Alt-R Cas12a crRNA	HPRT-38330 EcoRI -1	AATTATAAGCCATTTCACATA	Unmodified	AAGAATGTTGTGATAAAAGGTGATGCT	ACACATCCATGGGACTTCTGCCTC	NA	NA
5B	HPRT	Cas12a	HPRT-38330-AS	GGTTAAAGATGGTTAAATGAT	Alt-R Cas12a crRNA	HPRT-38330 EcoRI -15	TAAGCAATTATAAGCCATTTC	Unmodified	AAGAATGTTGTGATAAAAGGTGATGCT	ACACATCCATGGGACTTCTGCCTC	NA	NA
5B	HPRT	Cas12a	HPRT-38330-AS	GGTTAAAGATGGTTAAATGAT	Alt-R Cas12a crRNA	HPRT-38330 EcoRI -21	ATTCTCTAAGCAATTATAAGC	Unmodified	AAGAATGTTGTGATAAAAGGTGATGCT	ACACATCCATGGGACTTCTGCCTC	NA	NA
5B	HPRT	Cas12a	HPRT-38330-AS	GGTTAAAGATGGTTAAATGAT	Alt-R Cas12a crRNA	HPRT-38330 EcoRI -27	ACAAATATTCTCTAAGCAATT	Unmodified	AAGAATGTTGTGATAAAAGGTGATGCT	ACACATCCATGGGACTTCTGCCTC	NA	NA
5B	HPRT	Cas12a	HPRT-38115-AS	ACACACCCAAGGAAAGACTAT	Alt-R Cas12a crRNA	HPRT-38115 EcoRI 0	GAGTGTACCATGGTCACTTTT	Unmodified	AAGAATGTTGTGATAAAAGGTGATGCT	ACACATCCATGGGACTTCTGCCTC	NA	NA
5B	HPRT	Cas12a	HPRT-38115-AS	ACACACCCAAGGAAAGACTAT	Alt-R Cas12a crRNA	HPRT-38115 EcoRI +2	GAGTGTACCATGGTCACTTTT	Unmodified	AAGAATGTTGTGATAAAAGGTGATGCT	ACACATCCATGGGACTTCTGCCTC	NA	NA
5B	HPRT	Cas12a	HPRT-38115-AS	ACACACCCAAGGAAAGACTAT	Alt-R Cas12a crRNA	HPRT-38115 EcoRI +4	GAGTGTACCATGGTCACTTTT	Unmodified	AAGAATGTTGTGATAAAAGGTGATGCT	ACACATCCATGGGACTTCTGCCTC	NA	NA
5B	HPRT	Cas12a	HPRT-38115-AS	ACACACCCAAGGAAAGACTAT	Alt-R Cas12a crRNA	HPRT-38115 EcoRI +6	GAGTGTACCATGGTCACTTTT	Unmodified	AAGAATGTTGTGATAAAAGGTGATGCT	ACACATCCATGGGACTTCTGCCTC	NA	NA
5B	HPRT	Cas12a	HPRT-38115-AS	ACACACCCAAGGAAAGACTAT	Alt-R Cas12a crRNA	HPRT-38115 EcoRI +10	GAGTGTACCATGGTCACTTTT	Unmodified	AAGAATGTTGTGATAAAAGGTGATGCT	ACACATCCATGGGACTTCTGCCTC	NA	NA
5B	HPRT	Cas12a	HPRT-38115-AS	ACACACCCAAGGAAAGACTAT	Alt-R Cas12a crRNA	HPRT-38115 EcoRI +15	GAGTGTACCATGGTCACTTTT	Unmodified	AAGAATGTTGTGATAAAAGGTGATGCT	ACACATCCATGGGACTTCTGCCTC	NA	NA
5B	HPRT	Cas12a	HPRT-38115-AS	ACACACCCAAGGAAAGACTAT	Alt-R Cas12a crRNA	HPRT-38115 EcoRI +21	GAGTGTACCATGGTCACTTTT	Unmodified	AAGAATGTTGTGATAAAAGGTGATGCT	ACACATCCATGGGACTTCTGCCTC	NA	NA
5B	HPRT	Cas12a	HPRT-38115-AS	ACACACCCAAGGAAAGACTAT	Alt-R Cas12a crRNA	HPRT-38115 EcoRI +27	GAGTGTACCATGGTCACTTTT	Unmodified	AAGAATGTTGTGATAAAAGGTGATGCT	ACACATCCATGGGACTTCTGCCTC	NA	NA
5B	HPRT	Cas12a	HPRT-38115-AS	ACACACCCAAGGAAAGACTAT	Alt-R Cas12a crRNA	HPRT-38115 EcoRI -2	GAGTGTACCATGGTCACTTTT	Unmodified	AAGAATGTTGTGATAAAAGGTGATGCT	ACACATCCATGGGACTTCTGCCTC	NA	NA
5B	HPRT	Cas12a	HPRT-38115-AS	ACACACCCAAGGAAAGACTAT	Alt-R Cas12a crRNA	HPRT-38115 EcoRI -4	GAGTGTACCATGGTCACTTTT	Unmodified	AAGAATGTTGTGATAAAAGGTGATGCT	ACACATCCATGGGACTTCTGCCTC	NA	NA
5B	HPRT	Cas12a	HPRT-38115-AS	ACACACCCAAGGAAAGACTAT	Alt-R Cas12a crRNA	HPRT-38115 EcoRI -6	GAGTGTACCATGGTCACTTTT	Unmodified	AAGAATGTTGTGATAAAAGGTGATGCT	ACACATCCATGGGACTTCTGCCTC	NA	NA
5B	HPRT	Cas12a	HPRT-38115-AS	ACACACCCAAGGAAAGACTAT	Alt-R Cas12a crRNA	HPRT-38115 EcoRI -10	GAGTGTACCATGGTCACTTTT	Unmodified	AAGAATGTTGTGATAAAAGGTGATGCT	ACACATCCATGGGACTTCTGCCTC	NA	NA
5B	HPRT	Cas12a	HPRT-38115-AS	ACACACCCAAGGAAAGACTAT	Alt-R Cas12a crRNA	HPRT-38115 EcoRI -15	GAGTGTACCATGGTCACTTTT	Unmodified	AAGAATGTTGTGATAAAAGGTGATGCT	ACACATCCATGGGACTTCTGCCTC	NA	NA
5B	HPRT	Cas12a	HPRT-38115-AS	ACACACCCAAGGAAAGACTAT	Alt-R Cas12a crRNA	HPRT-38115 EcoRI -21	GAGTGTACCATGGTCACTTga	Unmodified	AAGAATGTTGTGATAAAAGGTGATGCT	ACACATCCATGGGACTTCTGCCTC	NA	NA
5B	HPRT	Cas12a	HPRT-38115-AS	ACACACCCAAGGAAAGACTAT	Alt-R Cas12a crRNA	HPRT-38115 EcoRI -27	GAGTGTACCATGGgaattcTCA	Unmodified	AAGAATGTTGTGATAAAAGGTGATGCT	ACACATCCATGGGACTTCTGCCTC	NA	NA
5B	HPRT	Cas12a	HPRT-38104-S	CTTGGGTGTGTTAAAAGTGAC	Alt-R Cas12a crRNA	HPRT-38104 EcoRI 0	GCTCTCCATTTCATAGTCTTTC	Unmodified	AAGAATGTTGTGATAAAAGGTGATGCT	ACACATCCATGGGACTTCTGCCTC	NA	NA
5B	HPRT	Cas12a	HPRT-38104-S	CTTGGGTGTGTTAAAAGTGAC	Alt-R Cas12a crRNA	HPRT-38104 EcoRI +2	GCTCTCCATTTCATAGTCTTTC	Unmodified	AAGAATGTTGTGATAAAAGGTGATGCT	ACACATCCATGGGACTTCTGCCTC	NA	NA
5B	HPRT	Cas12a	HPRT-38104-S	CTTGGGTGTGTTAAAAGTGAC	Alt-R Cas12a crRNA	HPRT-38104 EcoRI +4	GCTCTCCATTTCATAGTCTTTC	Unmodified	AAGAATGTTGTGATAAAAGGTGATGCT	ACACATCCATGGGACTTCTGCCTC	NA	NA
5B	HPRT	Cas12a	HPRT-38104-S	CTTGGGTGTGTTAAAAGTGAC	Alt-R Cas12a crRNA	HPRT-38104 EcoRI +6	GCTCTCCATTTCATAGTCTTTC	Unmodified	AAGAATGTTGTGATAAAAGGTGATGCT	ACACATCCATGGGACTTCTGCCTC	NA	NA
5B	HPRT	Cas12a	HPRT-38104-S	CTTGGGTGTGTTAAAAGTGAC	Alt-R Cas12a crRNA	HPRT-38104 EcoRI +10	GCTCTCCATTTCATAGTCTTTC	Unmodified	AAGAATGTTGTGATAAAAGGTGATGCT	ACACATCCATGGGACTTCTGCCTC	NA	NA
5B	HPRT	Cas12a	HPRT-38104-S	CTTGGGTGTGTTAAAAGTGAC	Alt-R Cas12a crRNA	HPRT-38104 EcoRI +15	GCTCTCCATTTCATAGTCTTTC	Unmodified	AAGAATGTTGTGATAAAAGGTGATGCT	ACACATCCATGGGACTTCTGCCTC	NA	NA
5B	HPRT	Cas12a	HPRT-38104-S	CTTGGGTGTGTTAAAAGTGAC	Alt-R Cas12a crRNA	HPRT-38104 EcoRI +21	GCTCTCCATTTCATAGTCTTTC	Unmodified	AAGAATGTTGTGATAAAAGGTGATGCT	ACACATCCATGGGACTTCTGCCTC	NA	NA
5B	HPRT	Cas12a	HPRT-38104-S	CTTGGGTGTGTTAAAAGTGAC	Alt-R Cas12a crRNA	HPRT-38104 EcoRI +27	GCTCTCCATTTCATAGTCTTTC	Unmodified	AAGAATGTTGTGATAAAAGGTGATGCT	ACACATCCATGGGACTTCTGCCTC	NA	NA
5B	HPRT	Cas12a	HPRT-38104-S	CTTGGGTGTGTTAAAAGTGAC	Alt-R Cas12a crRNA	HPRT-38104 EcoRI -2	GCTCTCCATTTCATAGTCTTTC	Unmodified	AAGAATGTTGTGATAAAAGGTGATGCT	ACACATCCATGGGACTTCTGCCTC	NA	NA
5B	HPRT	Cas12a	HPRT-38104-S	CTTGGGTGTGTTAAAAGTGAC	Alt-R Cas12a crRNA	HPRT-38104 EcoRI -4	GCTCTCCATTTCATAGTCTTTC	Unmodified	AAGAATGTTGTGATAAAAGGTGATGCT	ACACATCCATGGGACTTCTGCCTC	NA	NA
5B	HPRT	Cas12a	HPRT-38104-S	CTTGGGTGTGTTAAAAGTGAC	Alt-R Cas12a crRNA	HPRT-38104 EcoRI -6	GCTCTCCATTTCATAGTCTTTC	Unmodified	AAGAATGTTGTGATAAAAGGTGATGCT	ACACATCCATGGGACTTCTGCCTC	NA	NA
5B	HPRT	Cas12a	HPRT-38104-S	CTTGGGTGTGTTAAAAGTGAC	Alt-R Cas12a crRNA	HPRT-38104 EcoRI -10	GCTCTCCATTTCATAGTCTTTC	Unmodified	AAGAATGTTGTGATAAAAGGTGATGCT	ACACATCCATGGGACTTCTGCCTC	NA	NA
5B	HPRT	Cas12a	HPRT-38104-S	CTTGGGTGTGTTAAAAGTGAC	Alt-R Cas12a crRNA	HPRT-38104 EcoRI -15	GCTCTCCATTTCATAGTCTTTC	Unmodified	AAGAATGTTGTGATAAAAGGTGATGCT	ACACATCCATGGGACTTCTGCCTC	NA	NA
5B	HPRT	Cas12a	HPRT-38104-S	CTTGGGTGTGTTAAAAGTGAC	Alt-R Cas12a crRNA	HPRT-38104 EcoRI -21	GCTCTCCATTTCATAGTCTgaa	Unmodified	AAGAATGTTGTGATAAAAGGTGATGCT	ACACATCCATGGGACTTCTGCCTC	NA	NA
5B	HPRT	Cas12a	HPRT-38104-S	CTTGGGTGTGTTAAAAGTGAC	Alt-R Cas12a crRNA	HPRT-38104 EcoRI -27	GCTCTCCATTTCAgaattcTAGT	Unmodified	AAGAATGTTGTGATAAAAGGTGATGCT	ACACATCCATGGGACTTCTGCCTC	NA	NA
5B	HPRT	Cas12a	HPRT-38186-S	TAATGCCCTGTAGTCTCTCTG	Alt-R Cas12a crRNA	HPRT-38186 EcoRI 0	GAAACGTCAGTCTTCTCTTTT	Unmodified	AAGAATGTTGTGATAAAAGGTGATGCT	ACACATCCATGGGACTTCTGCCTC	NA	NA
5B	HPRT	Cas12a	HPRT-38186-S	TAATGCCCTGTAGTCTCTCTG	Alt-R Cas12a crRNA	HPRT-38186 EcoRI +2	GAAACGTCAGTCTTCTCTTTT	Unmodified	AAGAATGTTGTGATAAAAGGTGATGCT	ACACATCCATGGGACTTCTGCCTC	NA	NA
5B	HPRT	Cas12a	HPRT-38186-S	TAATGCCCTGTAGTCTCTCTG	Alt-R Cas12a crRNA	HPRT-38186 EcoRI +4	GAAACGTCAGTCTTCTCTTTT	Unmodified	AAGAATGTTGTGATAAAAGGTGATGCT	ACACATCCATGGGACTTCTGCCTC	NA	NA
5B	HPRT	Cas12a	HPRT-38186-S	TAATGCCCTGTAGTCTCTCTG	Alt-R Cas12a crRNA	HPRT-38186 EcoRI +6	GAAACGTCAGTCTTCTCTTTT	Unmodified	AAGAATGTTGTGATAAAAGGTGATGCT	ACACATCCATGGGACTTCTGCCTC	NA	NA
5B	HPRT	Cas12a	HPRT-38186-S	TAATGCCCTGTAGTCTCTCTG	Alt-R Cas12a crRNA	HPRT-38186 EcoRI +10	GAAACGTCAGTCTTCTCTTTT	Unmodified	AAGAATGTTGTGATAAAAGGTGATGCT	ACACATCCATGGGACTTCTGCCTC	NA	NA
5B	HPRT	Cas12a	HPRT-38186-S	TAATGCCCTGTAGTCTCTCTG	Alt-R Cas12a crRNA	HPRT-38186 EcoRI +15	GAAACGTCAGTCTTCTCTTTT	Unmodified	AAGAATGTTGTGATAAAAGGTGATGCT	ACACATCCATGGGACTTCTGCCTC	NA	NA
5B	HPRT	Cas12a	HPRT-38186-S	TAATGCCCTGTAGTCTCTCTG	Alt-R Cas12a crRNA	HPRT-38186 EcoRI +21	GAAACGTCAGTCTTCTCTTTT	Unmodified	AAGAATGTTGTGATAAAAGGTGATGCT	ACACATCCATGGGACTTCTGCCTC	NA	NA
5B	HPRT	Cas12a	HPRT-38186-S	TAATGCCCTGTAGTCTCTCTG	Alt-R Cas12a crRNA	HPRT-38186 EcoRI +27	GAAACGTCAGTCTTCTCTTTT	Unmodified	AAGAATGTTGTGATAAAAGGTGATGCT	ACACATCCATGGGACTTCTGCCTC	NA	NA
5B	HPRT	Cas12a	HPRT-38186-S	TAATGCCCTGTAGTCTCTCTG	Alt-R Cas12a crRNA	HPRT-38186 EcoRI -2	GAAACGTCAGTCTTCTCTTTT	Unmodified	AAGAATGTTGTGATAAAAGGTGATGCT	ACACATCCATGGGACTTCTGCCTC	NA	NA
5B	HPRT	Cas12a	HPRT-38186-S	TAATGCCCTGTAGTCTCTCTG	Alt-R Cas12a crRNA	HPRT-38186 EcoRI -4	GAAACGTCAGTCTTCTCTTTT	Unmodified	AAGAATGTTGTGATAAAAGGTGATGCT	ACACATCCATGGGACTTCTGCCTC	NA	NA
5B	HPRT	Cas12a	HPRT-38186-S	TAATGCCCTGTAGTCTCTCTG	Alt-R Cas12a crRNA	HPRT-38186 EcoRI -6	GAAACGTCAGTCTTCTCTTTT	Unmodified	AAGAATGTTGTGATAAAAGGTGATGCT	ACACATCCATGGGACTTCTGCCTC	NA	NA
5B	HPRT	Cas12a	HPRT-38186-S	TAATGCCCTGTAGTCTCTCTG	Alt-R Cas12a crRNA	HPRT-38186 EcoRI -10	GAAACGTCAGTCTTCTCTTTT	Unmodified	AAGAATGTTGTGATAAAAGGTGATGCT	ACACATCCATGGGACTTCTGCCTC	NA	NA
5B	HPRT	Cas12a	HPRT-38186-S	TAATGCCCTGTAGTCTCTCTG	Alt-R Cas12a crRNA	HPRT-38186 EcoRI -15	GAAACGTCAGTCTTCTCTTTT	Unmodified	AAGAATGTTGTGATAAAAGGTGATGCT	ACACATCCATGGGACTTCTGCCTC	NA	NA
5B	HPRT	Cas12a	HPRT-38186-S	TAATGCCCTGTAGTCTCTCTG	Alt-R Cas12a crRNA	HPRT-38186 EcoRI -21	GAAACGTCAGTCTTCTCTTga	Unmodified	AAGAATGTTGTGATAAAAGGTGATGCT	ACACATCCATGGGACTTCTGCCTC	NA	NA
5B	HPRT	Cas12a	HPRT-38186-S	TAATGCCCTGTAGTCTCTCTG	Alt-R Cas12a crRNA	HPRT-38186 EcoRI -27	GAAACGTCAGTCTgaattcTCT	Unmodified	AAGAATGTTGTGATAAAAGGTGATGCT	ACACATCCATGGGACTTCTGCCTC	NA	NA
5B	HPRT	Cas12a	HPRT-38228-S	TAATTAACAGCTTGCTGGTGA	Alt-R Cas12a crRNA	HPRT-38228 EcoRI 0	GTATGTTATATGTCACATTTT	Unmodified	AAGAATGTTGTGATAAAAGGTGATGCT	ACACATCCATGGGACTTCTGCCTC	NA	NA
5B	HPRT	Cas12a	HPRT-38228-S	TAATTAACAGCTTGCTGGTGA	Alt-R Cas12a crRNA	HPRT-38228 EcoRI +2	GTATGTTATATGTCACATTTT	Unmodified	AAGAATGTTGTGATAAAAGGTGATGCT	ACACATCCATGGGACTTCTGCCTC	NA	NA
5B	HPRT	Cas12a	HPRT-38228-S	TAATTAACAGCTTGCTGGTGA	Alt-R Cas12a crRNA	HPRT-38228 EcoRI +4	GTATGTTATATGTCACATTTT	Unmodified	AAGAATGTTGTGATAAAAGGTGATGCT	ACACATCCATGGGACTTCTGCCTC	NA	NA
5B	HPRT	Cas12a	HPRT-38228-S	TAATTAACAGCTTGCTGGTGA	Alt-R Cas12a crRNA	HPRT-38228 EcoRI +6	GTATGTTATATGTCACATTTT	Unmodified	AAGAATGTTGTGATAAAAGGTGATGCT	ACACATCCATGGGACTTCTGCCTC	NA	NA
5B	HPRT	Cas12a	HPRT-38228-S	TAATTAACAGCTTGCTGGTGA	Alt-R Cas12a crRNA	HPRT-38228 EcoRI +10	GTATGTTATATGTCACATTTT	Unmodified	AAGAATGTTGTGATAAAAGGTGATGCT	ACACATCCATGGGACTTCTGCCTC	NA	NA
5B	HPRT	Cas12a	HPRT-38228-S	TAATTAACAGCTTGCTGGTGA	Alt-R Cas12a crRNA	HPRT-38228 EcoRI +15	GTATGTTATATGTCACATTTT	Unmodified	AAGAATGTTGTGATAAAAGGTGATGCT	ACACATCCATGGGACTTCTGCCTC	NA	NA
5B	HPRT	Cas12a	HPRT-38228-S	TAATTAACAGCTTGCTGGTGA	Alt-R Cas12a crRNA	HPRT-38228 EcoRI +21	GTATGTTATATGTCACATTTT	Unmodified	AAGAATGTTGTGATAAAAGGTGATGCT	ACACATCCATGGGACTTCTGCCTC	NA	NA
5B	HPRT	Cas12a	HPRT-38228-S	TAATTAACAGCTTGCTGGTGA	Alt-R Cas12a crRNA	HPRT-38228 EcoRI +27	GTATGTTATATGTCACATTTT	Unmodified	AAGAATGTTGTGATAAAAGGTGATGCT	ACACATCCATGGGACTTCTGCCTC	NA	NA
5B	HPRT	Cas12a	HPRT-38228-S	TAATTAACAGCTTGCTGGTGA	Alt-R Cas12a crRNA	HPRT-38228 EcoRI -2	GTATGTTATATGTCACATTTT	Unmodified	AAGAATGTTGTGATAAAAGGTGATGCT	ACACATCCATGGGACTTCTGCCTC	NA	NA
5B	HPRT	Cas12a	HPRT-38228-S	TAATTAACAGCTTGCTGGTGA	Alt-R Cas12a crRNA	HPRT-38228 EcoRI -4	GTATGTTATATGTCACATTTT	Unmodified	AAGAATGTTGTGATAAAAGGTGATGCT	ACACATCCATGGGACTTCTGCCTC	NA	NA
5B	HPRT	Cas12a	HPRT-38228-S	TAATTAACAGCTTGCTGGTGA	Alt-R Cas12a crRNA	HPRT-38228 EcoRI -6	GTATGTTATATGTCACATTTT	Unmodified	AAGAATGTTGTGATAAAAGGTGATGCT	ACACATCCATGGGACTTCTGCCTC	NA	NA
5B	HPRT	Cas12a	HPRT-38228-S	TAATTAACAGCTTGCTGGTGA	Alt-R Cas12a crRNA	HPRT-38228 EcoRI -10	GTATGTTATATGTCACATTTT	Unmodified	AAGAATGTTGTGATAAAAGGTGATGCT	ACACATCCATGGGACTTCTGCCTC	NA	NA
5B	HPRT	Cas12a	HPRT-38228-S	TAATTAACAGCTTGCTGGTGA	Alt-R Cas12a crRNA	HPRT-38228 EcoRI -15	GTATGTTATATGTCACATTTT	Unmodified	AAGAATGTTGTGATAAAAGGTGATGCT	ACACATCCATGGGACTTCTGCCTC	NA	NA
5B	HPRT	Cas12a	HPRT-38228-S	TAATTAACAGCTTGCTGGTGA	Alt-R Cas12a crRNA	HPRT-38228 EcoRI -21	GTATGTTATATGTCACATTgaa	Unmodified	AAGAATGTTGTGATAAAAGGTGATGCT	ACACATCCATGGGACTTCTGCCTC	NA	NA
5B	HPRT	Cas12a	HPRT-38228-S	TAATTAACAGCTTGCTGGTGA	Alt-R Cas12a crRNA	HPRT-38228 EcoRI -27	GTATGTTATATGTgaattcCAC	Unmodified	AAGAATGTTGTGATAAAAGGTGATGCT	ACACATCCATGGGACTTCTGCCTC	NA	NA
5C	EMX1	Cas12a	EMX1-17983-S	TTTTGATGCATTTCTGTTTTA	Alt-R Cas12a crRNA	EMX1-17983-S-T-EcoRI	CTGGGGATCACTAAACTACAC	Alt-R modified	NA	NA	acactctttccctacacgacgctcttccgatctCCACTTGGCCTTCTCCTC	gtgactggagttcagacgtgtgctcttccgatctCTGGATGCCCGTGTCATTAA
5C	APOC	Cas12a	APOC-1198-AS	TCAAAGTATCAGCAAACTTAT	Alt-R Cas12a crRNA	APOC-1198-AS-T-EcoRI	GCCTTCCTTGACAGCTTTGTG	Alt-R modified	NA	NA	acactctttccctacacgacgctcttccgatctTCATCTCTAAGGTAGGAATCACC	gtgactggagttcagacgtgtgctcttccgatctCAGCTCCACAGTTTTGAGTAAA
5C	HPRT	Cas12a	HPRT-803-S	AAGCCGAAATTCACATGTGCT	Alt-R Cas12a crRNA	HPRT-803-S-T-EcoRI	CTATATCCTGGGGCATCGGTC	Alt-R modified	NA	NA	acactctttccctacacgacgctcttccgatctCGTATTTGACACACGAAGGAACTAG	gtgactggagttcagacgtgtgctcttccgatctCAGAGGCTGATTACGGTTAAGAAT
5C	EMX1	Cas12a	EMX1-10875-S	CGATTTTCTATTAGTTATATG	Alt-R Cas12a crRNA	EMX1-10875-S-T-EcoRI	AATGAACAAATGGGTAAATA	Alt-R modified	NA	NA	acactctttccctacacgacgctcttccgatctTGCTCTCATTAAACTGCCTC	gtgactggagttcagacgtgtgctcttccgatctCTGTTTGTCAGTTGCGAAATA
5C	DICER	Cas12a	DICER-33765-S	TGCGGAATTATTTTTGTGGAA	Alt-R Cas12a crRNA	DICER-33765-S-T-EcoRI	TACAGATTTACCTGTTTAAGA	Alt-R modified	NA	NA	acactctttccctacacgacgctcttccgatctTCCCTGTTCTCATGTGAAAG	gtgactggagttcagacgtgtgctcttccgatctAGCCAGAGACAAATTTTCCTT
5C	HPRT	Cas12a	HPRT-38628-S	AACAGACTGATGGTCCCATT	Alt-R Cas12a crRNA	HPRT-38628-S-T-EcoRI	TTCTAAGGACGTCTGTACTAG	Alt-R modified	NA	NA	acactctttccctacacgacgctcttccgatctTGCCCTTGACTATAATGAATACTT	gtgactggagttcagacgtgtgctcttccgatctACTATTTCAGCCAACAAGAAGT
5C	RAG2	Cas12a	RAG2-907-S	CTGGTGGATTTTGAATTTGGG	Alt-R Cas12a crRNA	RAG2-907-S-T-EcoRI	GCCCATCCTGAAGTTCTGGAA	Alt-R modified	NA	NA	acactctttccctacacgacgctcttccgatctGAGACATGAAAAGATAGCCCAT	gtgactggagttcagacgtgtgctcttccgatctCTCATACATGCCTTCTACCCA
5C	HPRT	Cas12a	HPRT-677-S	CACACACTGTAGTTCATCTTT	Alt-R Cas12a crRNA	HPRT-677-S-T-EcoRI	AGCTGTAGTGGGGCTTCAATC	Alt-R modified	NA	NA	acactctttccctacacgacgctcttccgatctGAGAGGCCCTTCCCTG	gtgactggagttcagacgtgtgctcttccgatctCGCATGGTTACCGCTAC
5C	AR	Cas12a	AR-710-S	CGTGGTTGCTCCCGCAAGTTT	Alt-R Cas12a crRNA	AR-710-S-T-EcoRI	CTGCAGCTAGCTGCCCACCTG	Alt-R modified	NA	NA	acactctttccctacacgacgctcttccgatctTTCTGCACGAGACTTTGAG	gtgactggagttcagacgtgtgctcttccgatctTCAACAGGCTGTGATGATG
5C	G6PC	Cas12a	G6PC-945-AS	AACTCACTGGTAGGATTTTTT	Alt-R Cas12a crRNA	G6PC-945-AS-T-EcoRI	CTCTCTGACTTTGGATCATCT	Alt-R modified	NA	NA	acactctttccctacacgacgctcttccgatctCCTCTCTCTGACTTTGGATCAT	gtgactggagttcagacgtgtgctcttccgatctGACTTTTCACGAAATTCAACTGTC
5C	GCK	Cas12a	GCK-1282-S	CTGTGAAACACTGGGGGGAAT	Alt-R Cas12a crRNA	GCK-1282-S-T-EcoRI	CCCACTCTGCCTGGGCCCCCA	Alt-R modified	NA	NA	acactctttccctacacgacgctcttccgatctTATGCTGACCTGGTCATTTCA	gtgactggagttcagacgtgtgctcttccgatctAAGAGAAATCCAGGCATGCT
5C	C5	Cas12a	C5-562-AS	CTCCTGTCCCCAGGTTTTCCC	Alt-R Cas12a crRNA	C5-562-AS-T-EcoRI	CATGGGCCTTTTGGGAATACT	Alt-R modified	NA	NA	acactctttccctacacgacgctcttccgatctGAGATGATACTATTCACTTGCTGTT	gtgactggagttcagacgtgtgctcttccgatctTCAAAAGTGTTATATCCGTGGTTTC
5C	MET	Cas12a	MET-1379-S	TTTACTTCTTGACGGTCCAAA	Alt-R Cas12a crRNA	MET-1379-S-T-EcoRI	ATTATTCTTGTGTGAAAAGTC	Alt-R modified	NA	NA	acactctttccctacacgacgctcttccgatctCTTACCCCATTAAGTATGTCCATG	gtgactggagttcagacgtgtgctcttccgatctTGGAACAGAACCTGATTATTCTTGT
5C	MET	Cas12a	MET-540-S	TCTTCATTTCTGACAACTGAA	Alt-R Cas12a crRNA	MET-540-S-T-EcoRI	GAGAGGTTTATCTGCCAAAA	Alt-R modified	NA	NA	acactctttccctacacgacgctcttccgatctTTCCTTCTATGTAAAAGTCCAGTTG	gtgactggagttcagacgtgtgctcttccgatctGCCTTCATTATGAGAGGTTTATCTG
5C	GYS2	Cas12a	GYS2-959-AS	AATGCTTTCCTCACTCACTAG	Alt-R Cas12a crRNA	GYS2-959-AS-T-EcoRI	AGTAAGATTTCACAGATAACT	Alt-R modified	NA	NA	acactctttccctacacgacgctcttccgatctCACATACACAAGCTCTGTATTCATAGC	gtgactggagttcagacgtgtgctcttccgatctTGACTGAGGAATGTAGAGTAAGATTT
5C	EMX1	Cas12a	EMX1-17983-S	TTTTGATGCATTTCTGTTTTA	Alt-R Cas12a crRNA	EMX1-17983-S-NT-EcoRI	TCCGTGTCTCCAATCTCCCTT	Alt-R modified	NA	NA	acactctttccctacacgacgctcttccgatctCCACTTGGCCTTCTCCTC	gtgactggagttcagacgtgtgctcttccgatctCTGGATGCCCGTGTCATTAA
5C	APOC	Cas12a	APOC-1198-AS	TCAAAGTATCAGCAAACTTAT	Alt-R Cas12a crRNA	APOC-1198-AS-NT-EcoRI	TGTTGTGCAGCACCTAACAGT	Alt-R modified	NA	NA	acactctttccctacacgacgctcttccgatctTCATCTCTAAGGTAGGAATCACC	gtgactggagttcagacgtgtgctcttccgatctCAGCTCCACAGTTTTGAGTAAA
5C	HPRT	Cas12a	HPRT-803-S	AAGCCGAAATTCACATGTGCT	Alt-R Cas12a crRNA	HPRT-803-S-NT-EcoRI	AGGGAAAAGGCATTAGGTCA	Alt-R modified	NA	NA	acactctttccctacacgacgctcttccgatctCGTATTTGACACACGAAGGAACTAG	gtgactggagttcagacgtgtgctcttccgatctCAGAGGCTGATTACGGTTAAGAAT
5C	EMX1	Cas12a	EMX1-10875-S	CGATTTTCTATTAGTTATATG	Alt-R Cas12a crRNA	EMX1-10875-S-NT-EcoRI	CCAAAACTGGCTAATTTATAT	Alt-R modified	NA	NA	acactctttccctacacgacgctcttccgatctTGCTCTCATTAAACTGCCTC	gtgactggagttcagacgtgtgctcttccgatctCTGTTTGTCAGTTGCGAAATA
5C	DICER	Cas12a	DICER-33765-S	TGCGGAATTATTTTTGTGGAA	Alt-R Cas12a crRNA	DICER-33765-S-NT-EcoRI	CCTTCTCCTTTTACCAACATTT	Alt-R modified	NA	NA	acactctttccctacacgacgctcttccgatctTCCCTGTTCTCATGTGAAAG	gtgactggagttcagacgtgtgctcttccgatctAGCCAGAGACAAATTTTCCTT
5C	HPRT	Cas12a	HPRT-38628-S	AACAGACTGATGGTCCCATT	Alt-R Cas12a crRNA	HPRT-38628-S-NT-EcoRI	CTCCTTCCAGCACCTCATAAT	Alt-R modified	NA	NA	acactctttccctacacgacgctcttccgatctTGCCCTTGACTATAATGAATACTT	gtgactggagttcagacgtgtgctcttccgatctACTATTTCAGCCAACAAGAAGT
5C	RAG2	Cas12a	RAG2-907-S	CTGGTGGATTTTGAATTTGGG	Alt-R Cas12a crRNA	RAG2-907-S-NT-EcoRI	GCTGACTGCCTGCCCTGTGTT	Alt-R modified	NA	NA	acactctttccctacacgacgctcttccgatctGAGACATGAAAAGATAGCCCAT	gtgactggagttcagacgtgtgctcttccgatctCTCATACATGCCTTCTACCCA
5C	HPRT	Cas12a	HPRT-677-S	CACACACTGTAGTTCATCTTT	Alt-R Cas12a crRNA	HPRT-677-S-NT-EcoRI	GACCAGGTTTTGCCTTTAGTT	Alt-R modified	NA	NA	acactctttccctacacgacgctcttccgatctGAGAGGCCCTTCCCTG	gtgactggagttcagacgtgtgctcttccgatctCGCATGGTTACCGCTAC
5C	AR	Cas12a	AR-710-S	CGTGGTTGCTCCCGCAAGTTT	Alt-R Cas12a crRNA	AR-710-S-NT-EcoRI	TTGAGGCTGTCAGAGCGCTTT	Alt-R modified	NA	NA	acactctttccctacacgacgctcttccgatctTTCTGCACGAGACTTTGAG	gtgactggagttcagacgtgtgctcttccgatctTCAACAGGCTGTGATGATG
5C	G6PC	Cas12a	G6PC-945-AS	AACTCACTGGTAGGATTTTTT	Alt-R Cas12a crRNA	G6PC-945-AS-NT-EcoRI	TGAAATAGGCTTTCCTGTATT	Alt-R modified	NA	NA	acactctttccctacacgacgctcttccgatctCCTCTCTCTGACTTTGGATCAT	gtgactggagttcagacgtgtgctcttccgatctGACTTTTCACGAAATTCAACTGTC
5C	GCK	Cas12a	GCK-1282-S	CTGTGAAACACTGGGGGGAAT	Alt-R Cas12a crRNA	GCK-1282-S-NT-EcoRI	GAGAGCTGGAGACAGGGGA	Alt-R modified	NA	NA	acactctttccctacacgacgctcttccgatctTATGCTGACCTGGTCATTTCA	gtgactggagttcagacgtgtgctcttccgatctAAGAGAAATCCAGGCATGCT
5C	C5	Cas12a	C5-562-AS	CTCCTGTCCCCAGGTTTTCCC	Alt-R Cas12a crRNA	C5-562-AS-NT-EcoRI	GATAGCTTGTTTTACTTACGT	Alt-R modified	NA	NA	acactctttccctacacgacgctcttccgatctGAGATGATACTATTCACTTGCTGTT	gtgactggagttcagacgtgtgctcttccgatctTCAAAAGTGTTATATCCGTGGTTTC
5C	MET	Cas12a	MET-1379-S	TTTACTTCTTGACGGTCCAAA	Alt-R Cas12a crRNA	MET-1379-S-NT-EcoRI	ATGCCTTTGAAAGCAACAATT	Alt-R modified	NA	NA	acactctttccctacacgacgctcttccgatctCTTACCCCATTAAGTATGTCCATG	gtgactggagttcagacgtgtgctcttccgatctTGGAACAGAACCTGATTATTCTTGT
5C	MET	Cas12a	MET-540-S	TCTTCATTTCTGACAACTGAA	Alt-R Cas12a crRNA	MET-540-S-NT-EcoRI	AATGGTATAGGTCTTTCAGTT	Alt-R modified	NA	NA	acactctttccctacacgacgctcttccgatctTTCCTTCTATGTAAAAGTCCAGTTG	gtgactggagttcagacgtgtgctcttccgatctGCCTTCATTATGAGAGGTTTATCTG
5C	GYS2	Cas12a	GYS2-959-AS	AATGCTTTCCTCACTCACTAG	Alt-R Cas12a crRNA	GYS2-959-AS-NT-EcoRI	TAGCCTAAATATATATTCCGT	Alt-R modified	NA	NA	acactctttccctacacgacgctcttccgatctCACATACACAAGCTCTGTATTCATAGC	gtgactggagttcagacgtgtgctcttccgatctTGACTGAGGAATGTAGAGTAAGATTT
5E	HPRT1	Cas12a	HPRT-38330	GGTTAAAGATGGTTAAATGAT	Alt-R Cas12a crRNA	HPRT-38330 p15ins	G*C*AATTATAAGCCATTTCA	PS modified	NA	NA	ACACTCTTTCCCTACACGACGCTCTTCCGATCTGTCAGTCTTCTCTTTTGTAATGCC	GTGACTGGAGTTCAGACGTGTGCTCTTCCGATCTGCAAATGTGCCTCTCTACAAATATT
5E	HPRT1	Cas12a	HPRT-38330	GGTTAAAGATGGTTAAATGAT	Alt-R Cas12a crRNA	HPRT-38330 p15ins_PAM	G*C*AATTATAAGCCATTTCA	PS modified	NA	NA	ACACTCTTTCCCTACACGACGCTCTTCCGATCTGTCAGTCTTCTCTTTTGTAATGCC	GTGACTGGAGTTCAGACGTGTGCTCTTCCGATCTGCAAATGTGCCTCTCTACAAATATT
5E	HPRT1	Cas12a	HPRT-38330	GGTTAAAGATGGTTAAATGAT	Alt-R Cas12a crRNA	HPRT-38330 p15ins_p3	G*C*AATTATAAGCCATTTCA	PS modified	NA	NA	ACACTCTTTCCCTACACGACGCTCTTCCGATCTGTCAGTCTTCTCTTTTGTAATGCC	GTGACTGGAGTTCAGACGTGTGCTCTTCCGATCTGCAAATGTGCCTCTCTACAAATATT
5E	HPRT1	Cas12a	HPRT-38330	GGTTAAAGATGGTTAAATGAT	Alt-R Cas12a crRNA	HPRT-38330 p15ins_p8	G*C*AATTATAAGCCATTTCA	PS modified	NA	NA	ACACTCTTTCCCTACACGACGCTCTTCCGATCTGTCAGTCTTCTCTTTTGTAATGCC	GTGACTGGAGTTCAGACGTGTGCTCTTCCGATCTGCAAATGTGCCTCTCTACAAATATT
5E	HPRT1	Cas12a	HPRT-38330	GGTTAAAGATGGTTAAATGAT	Alt-R Cas12a crRNA	HPRT-38330 p15ins_p14	G*C*AATTATAAGCCATTTCA	PS modified	NA	NA	ACACTCTTTCCCTACACGACGCTCTTCCGATCTGTCAGTCTTCTCTTTTGTAATGCC	GTGACTGGAGTTCAGACGTGTGCTCTTCCGATCTGCAAATGTGCCTCTCTACAAATATT
5E	HPRT1	Cas12a	HPRT-38330	GGTTAAAGATGGTTAAATGAT	Alt-R Cas12a crRNA	HPRT-38330 p15ins_p18	G*C*AATTATAAGCCATTTCA	PS modified	NA	NA	ACACTCTTTCCCTACACGACGCTCTTCCGATCTGTCAGTCTTCTCTTTTGTAATGCC	GTGACTGGAGTTCAGACGTGTGCTCTTCCGATCTGCAAATGTGCCTCTCTACAAATATT
5E	HPRT1	Cas12a	HPRT-38330	GGTTAAAGATGGTTAAATGAT	Alt-R Cas12a crRNA	HPRT-38330 p24ins	G*C*AATTATAAGCCATTTCA	PS modified	NA	NA	ACACTCTTTCCCTACACGACGCTCTTCCGATCTGTCAGTCTTCTCTTTTGTAATGCC	GTGACTGGAGTTCAGACGTGTGCTCTTCCGATCTGCAAATGTGCCTCTCTACAAATATT
5E	HPRT1	Cas12a	HPRT-38330	GGTTAAAGATGGTTAAATGAT	Alt-R Cas12a crRNA	HPRT-38330 p24ins_PAM	G*C*AATTATAAGCCATTTCA	PS modified	NA	NA	ACACTCTTTCCCTACACGACGCTCTTCCGATCTGTCAGTCTTCTCTTTTGTAATGCC	GTGACTGGAGTTCAGACGTGTGCTCTTCCGATCTGCAAATGTGCCTCTCTACAAATATT
5E	HPRT1	Cas12a	HPRT-38330	GGTTAAAGATGGTTAAATGAT	Alt-R Cas12a crRNA	HPRT-38330 p24ins_p3	G*C*AATTATAAGCCATTTCA	PS modified	NA	NA	ACACTCTTTCCCTACACGACGCTCTTCCGATCTGTCAGTCTTCTCTTTTGTAATGCC	GTGACTGGAGTTCAGACGTGTGCTCTTCCGATCTGCAAATGTGCCTCTCTACAAATATT
5E	HPRT1	Cas12a	HPRT-38330	GGTTAAAGATGGTTAAATGAT	Alt-R Cas12a crRNA	HPRT-38330 p24ins_p8	G*C*AATTATAAGCCATTTCA	PS modified	NA	NA	ACACTCTTTCCCTACACGACGCTCTTCCGATCTGTCAGTCTTCTCTTTTGTAATGCC	GTGACTGGAGTTCAGACGTGTGCTCTTCCGATCTGCAAATGTGCCTCTCTACAAATATT
5E	HPRT1	Cas12a	HPRT-38330	GGTTAAAGATGGTTAAATGAT	Alt-R Cas12a crRNA	HPRT-38330 p24ins_p14	G*C*AATTATAAGCCATTTCA	PS modified	NA	NA	ACACTCTTTCCCTACACGACGCTCTTCCGATCTGTCAGTCTTCTCTTTTGTAATGCC	GTGACTGGAGTTCAGACGTGTGCTCTTCCGATCTGCAAATGTGCCTCTCTACAAATATT
5E	HPRT1	Cas12a	HPRT-38330	GGTTAAAGATGGTTAAATGAT	Alt-R Cas12a crRNA	HPRT-38330 p24ins_p18	G*C*AATTATAAGCCATTTCA	PS modified	NA	NA	ACACTCTTTCCCTACACGACGCTCTTCCGATCTGTCAGTCTTCTCTTTTGTAATGCC	GTGACTGGAGTTCAGACGTGTGCTCTTCCGATCTGCAAATGTGCCTCTCTACAAATATT
5E	HPRT1	Cas12a	HPRT-38115	ACACACCCAAGGAAAGACTAT	Alt-R Cas12a crRNA	HPRT-38115 p15ins	T*T*CATCCGTGCTGAGTGTA	PS modified	NA	NA	ACACTCTTTCCCTACACGACGCTCTTCCGATCTTCAGAACTGTCCTTCAGGTTC	GTGACTGGAGTTCAGACGTGTGCTCTTCCGATCTCAGGGCATTACAAAAGAGAAGA
5E	HPRT1	Cas12a	HPRT-38115	ACACACCCAAGGAAAGACTAT	Alt-R Cas12a crRNA	HPRT-38115 p15ins_PAM	T*T*CATCCGTGCTGAGTGTA	PS modified	NA	NA	ACACTCTTTCCCTACACGACGCTCTTCCGATCTTCAGAACTGTCCTTCAGGTTC	GTGACTGGAGTTCAGACGTGTGCTCTTCCGATCTCAGGGCATTACAAAAGAGAAGA
5E	HPRT1	Cas12a	HPRT-38115	ACACACCCAAGGAAAGACTAT	Alt-R Cas12a crRNA	HPRT-38115 p15ins_p3	T*T*CATCCGTGCTGAGTGTA	PS modified	NA	NA	ACACTCTTTCCCTACACGACGCTCTTCCGATCTTCAGAACTGTCCTTCAGGTTC	GTGACTGGAGTTCAGACGTGTGCTCTTCCGATCTCAGGGCATTACAAAAGAGAAGA
5E	HPRT1	Cas12a	HPRT-38115	ACACACCCAAGGAAAGACTAT	Alt-R Cas12a crRNA	HPRT-38115 p15ins_p8	T*T*CATCCGTGCTGAGTGTA	PS modified	NA	NA	ACACTCTTTCCCTACACGACGCTCTTCCGATCTTCAGAACTGTCCTTCAGGTTC	GTGACTGGAGTTCAGACGTGTGCTCTTCCGATCTCAGGGCATTACAAAAGAGAAGA
5E	HPRT1	Cas12a	HPRT-38115	ACACACCCAAGGAAAGACTAT	Alt-R Cas12a crRNA	HPRT-38115 p15ins_p14	T*T*CATCCGTGCTGAGTGTA	PS modified	NA	NA	ACACTCTTTCCCTACACGACGCTCTTCCGATCTTCAGAACTGTCCTTCAGGTTC	GTGACTGGAGTTCAGACGTGTGCTCTTCCGATCTCAGGGCATTACAAAAGAGAAGA
5E	HPRT1	Cas12a	HPRT-38115	ACACACCCAAGGAAAGACTAT	Alt-R Cas12a crRNA	HPRT-38115 p15ins_p18	T*T*CATCCGTGCTGAGTGTA	PS modified	NA	NA	ACACTCTTTCCCTACACGACGCTCTTCCGATCTTCAGAACTGTCCTTCAGGTTC	GTGACTGGAGTTCAGACGTGTGCTCTTCCGATCTCAGGGCATTACAAAAGAGAAGA
5E	HPRT1	Cas12a	HPRT-38115	ACACACCCAAGGAAAGACTAT	Alt-R Cas12a crRNA	HPRT-38115 p24ins	T*T*CATCCGTGCTGAGTGTA	PS modified	NA	NA	ACACTCTTTCCCTACACGACGCTCTTCCGATCTTCAGAACTGTCCTTCAGGTTC	GTGACTGGAGTTCAGACGTGTGCTCTTCCGATCTCAGGGCATTACAAAAGAGAAGA
5E	HPRT1	Cas12a	HPRT-38115	ACACACCCAAGGAAAGACTAT	Alt-R Cas12a crRNA	HPRT-38115 p24ins mutPAM	T*T*CATCCGTGCTGAGTGTA	PS modified	NA	NA	ACACTCTTTCCCTACACGACGCTCTTCCGATCTTCAGAACTGTCCTTCAGGTTC	GTGACTGGAGTTCAGACGTGTGCTCTTCCGATCTCAGGGCATTACAAAAGAGAAGA
5E	HPRT1	Cas12a	HPRT-38115	ACACACCCAAGGAAAGACTAT	Alt-R Cas12a crRNA	HPRT-38115 p24ins_p3	T*T*CATCCGTGCTGAGTGTA	PS modified	NA	NA	ACACTCTTTCCCTACACGACGCTCTTCCGATCTTCAGAACTGTCCTTCAGGTTC	GTGACTGGAGTTCAGACGTGTGCTCTTCCGATCTCAGGGCATTACAAAAGAGAAGA
5E	HPRT1	Cas12a	HPRT-38115	ACACACCCAAGGAAAGACTAT	Alt-R Cas12a crRNA	HPRT-38115 p24ins_p8	T*T*CATCCGTGCTGAGTGTA	PS modified	NA	NA	ACACTCTTTCCCTACACGACGCTCTTCCGATCTTCAGAACTGTCCTTCAGGTTC	GTGACTGGAGTTCAGACGTGTGCTCTTCCGATCTCAGGGCATTACAAAAGAGAAGA
5E	HPRT1	Cas12a	HPRT-38115	ACACACCCAAGGAAAGACTAT	Alt-R Cas12a crRNA	HPRT-38115 p24ins_p14	T*T*CATCCGTGCTGAGTGTA	PS modified	NA	NA	ACACTCTTTCCCTACACGACGCTCTTCCGATCTTCAGAACTGTCCTTCAGGTTC	GTGACTGGAGTTCAGACGTGTGCTCTTCCGATCTCAGGGCATTACAAAAGAGAAGA
5E	HPRT1	Cas12a	HPRT-38115	ACACACCCAAGGAAAGACTAT	Alt-R Cas12a crRNA	HPRT-38115 p24ins_p18	T*T*CATCCGTGCTGAGTGTA	PS modified	NA	NA	ACACTCTTTCCCTACACGACGCTCTTCCGATCTTCAGAACTGTCCTTCAGGTTC	GTGACTGGAGTTCAGACGTGTGCTCTTCCGATCTCAGGGCATTACAAAAGAGAAGA
S1B	TNPO3	Cas9	TNPO3.-26-S	TTCCGAGGGGGCAACTTCCA	Alt-R Cas9 crRNA:tra	TNPO3 -26-S NT	C*C*TGAATACGACCTAATAG	PS modified	NA	NA	ACACTCTTTCCCTACACGACGCTCTTCCGATCTCTCAAGAGTCTAGAATCAAGTGGAAA	GTGACTGGAGTTCAGACGTGTGCTCTTCCGATCTGGTAGCGACGGCTCTGAT
S1B	TNPO3	Cas9	TNPO3.-20-AS	CCAGGGCAGAAGGCTCTCCG	Alt-R Cas9 crRNA:tra	TNPO3 -20-AS NT	T*C*GCGCTTCCTCACTGTCTC	PS modified	NA	NA	ACACTCTTTCCCTACACGACGCTCTTCCGATCTCTCAAGAGTCTAGAATCAAGTGGAAA	GTGACTGGAGTTCAGACGTGTGCTCTTCCGATCTGGTAGCGACGGCTCTGAT
S1B	TNPO3	Cas9	TNPO3.-10-AS	TTGGCCGTTACCAGGGCAGA	Alt-R Cas9 crRNA:tra	TNPO3 -10-AS NT	T*C*GCGCTTCCTCACTGTCTC	PS modified	NA	NA	ACACTCTTTCCCTACACGACGCTCTTCCGATCTCTCAAGAGTCTAGAATCAAGTGGAAA	GTGACTGGAGTTCAGACGTGTGCTCTTCCGATCTGGTAGCGACGGCTCTGAT
S1B	TNPO3	Cas9	TNPO3.-9-S	CCACGGAGAGCCTTCTGCCC	Alt-R Cas9 crRNA:tra	TNPO3 -9-S NT	C*C*TGAATACGACCTAATAG	PS modified	NA	NA	ACACTCTTTCCCTACACGACGCTCTTCCGATCTCTCAAGAGTCTAGAATCAAGTGGAAA	GTGACTGGAGTTCAGACGTGTGCTCTTCCGATCTGGTAGCGACGGCTCTGAT
S1B	TNPO3	Cas9	TNPO3.-3-S	AGAGCCTTCTGCCCTGGTAA	Alt-R Cas9 crRNA:tra	TNPO3 -3-S NT	C*C*TGAATACGACCTAATAG	PS modified	NA	NA	ACACTCTTTCCCTACACGACGCTCTTCCGATCTCTCAAGAGTCTAGAATCAAGTGGAAA	GTGACTGGAGTTCAGACGTGTGCTCTTCCGATCTGGTAGCGACGGCTCTGAT
S1B	TNPO3	Cas9	TNPO3.-3-AS	CTCCTCTTTGGCCGTTACCA	Alt-R Cas9 crRNA:tra	TNPO3 -3-AS NT	T*C*GCGCTTCCTCACTGTCTC	PS modified	NA	NA	ACACTCTTTCCCTACACGACGCTCTTCCGATCTCTCAAGAGTCTAGAATCAAGTGGAAA	GTGACTGGAGTTCAGACGTGTGCTCTTCCGATCTGGTAGCGACGGCTCTGAT
S1B	TNPO3	Cas9	TNPO3.-2-AS	CCTCCTCTTTGGCCGTTACC	Alt-R Cas9 crRNA:tra	TNPO3 -2-AS NT	T*C*GCGCTTCCTCACTGTCTC	PS modified	NA	NA	ACACTCTTTCCCTACACGACGCTCTTCCGATCTCTCAAGAGTCTAGAATCAAGTGGAAA	GTGACTGGAGTTCAGACGTGTGCTCTTCCGATCTGGTAGCGACGGCTCTGAT
S1B	TNPO3	Cas9	TNPO3.+6-S	TGCCCTGGTAACGGCCAAAG	Alt-R Cas9 crRNA:tra	TNPO3 +6-S NT	C*C*TGAATACGACCTAATAG	PS modified	NA	NA	ACACTCTTTCCCTACACGACGCTCTTCCGATCTCTCAAGAGTCTAGAATCAAGTGGAAA	GTGACTGGAGTTCAGACGTGTGCTCTTCCGATCTGGTAGCGACGGCTCTGAT
S1B	TNPO3	Cas9	TNPO3.+9-S	CCTGGTAACGGCCAAAGAGG	Alt-R Cas9 crRNA:tra	TNPO3 +9-S NT	C*C*TGAATACGACCTAATAG	PS modified	NA	NA	ACACTCTTTCCCTACACGACGCTCTTCCGATCTCTCAAGAGTCTAGAATCAAGTGGAAA	GTGACTGGAGTTCAGACGTGTGCTCTTCCGATCTGGTAGCGACGGCTCTGAT
S1B	TNPO3	Cas9	TNPO3.+9-AS	TGGCGCCATCTCCTCCTCTT	Alt-R Cas9 crRNA:tra	TNPO3 +9-AS NT	T*C*GCGCTTCCTCACTGTCTC	PS modified	NA	NA	ACACTCTTTCCCTACACGACGCTCTTCCGATCTCTCAAGAGTCTAGAATCAAGTGGAAA	GTGACTGGAGTTCAGACGTGTGCTCTTCCGATCTGGTAGCGACGGCTCTGAT
S1B	TNPO3	Cas9	TNPO3.+15-S	AACGGCCAAAGAGGAGGAGA	Alt-R Cas9 crRNA:tra	TNPO3 +15-S NT	C*C*TGAATACGACCTAATAG	PS modified	NA	NA	ACACTCTTTCCCTACACGACGCTCTTCCGATCTCTCAAGAGTCTAGAATCAAGTGGAAA	GTGACTGGAGTTCAGACGTGTGCTCTTCCGATCTGGTAGCGACGGCTCTGAT
S1B	TNPO3	Cas9	TNPO3.+26-S	AGGAGGAGATGGCGCCAGTC	Alt-R Cas9 crRNA:tra	TNPO3 +26-S NT	C*C*TGAATACGACCTAATAG	PS modified	NA	NA	ACACTCTTTCCCTACACGACGCTCTTCCGATCTCTCAAGAGTCTAGAATCAAGTGGAAA	GTGACTGGAGTTCAGACGTGTGCTCTTCCGATCTGGTAGCGACGGCTCTGAT
S1B	TNPO3	Cas9	TNPO3.-26-S	TTCCGAGGGGGCAACTTCCA	Alt-R Cas9 crRNA:tra	TNPO3 -26-S T	T*C*GCGCTTCCTCACTGTCTC	PS modified	NA	NA	ACACTCTTTCCCTACACGACGCTCTTCCGATCTCTCAAGAGTCTAGAATCAAGTGGAAA	GTGACTGGAGTTCAGACGTGTGCTCTTCCGATCTGGTAGCGACGGCTCTGAT
S1B	TNPO3	Cas9	TNPO3.-20-AS	CCAGGGCAGAAGGCTCTCCG	Alt-R Cas9 crRNA:tra	TNPO3 -20-AS T	C*C*TGAATACGACCTAATAG	PS modified	NA	NA	ACACTCTTTCCCTACACGACGCTCTTCCGATCTCTCAAGAGTCTAGAATCAAGTGGAAA	GTGACTGGAGTTCAGACGTGTGCTCTTCCGATCTGGTAGCGACGGCTCTGAT
S1B	TNPO3	Cas9	TNPO3.-10-AS	TTGGCCGTTACCAGGGCAGA	Alt-R Cas9 crRNA:tra	TNPO3 -10-AS T	C*C*TGAATACGACCTAATAG	PS modified	NA	NA	ACACTCTTTCCCTACACGACGCTCTTCCGATCTCTCAAGAGTCTAGAATCAAGTGGAAA	GTGACTGGAGTTCAGACGTGTGCTCTTCCGATCTGGTAGCGACGGCTCTGAT
S1B	TNPO3	Cas9	TNPO3.-9-S	CCACGGAGAGCCTTCTGCCC	Alt-R Cas9 crRNA:tra	TNPO3 -9-S T	T*C*GCGCTTCCTCACTGTCTC	PS modified	NA	NA	ACACTCTTTCCCTACACGACGCTCTTCCGATCTCTCAAGAGTCTAGAATCAAGTGGAAA	GTGACTGGAGTTCAGACGTGTGCTCTTCCGATCTGGTAGCGACGGCTCTGAT
S1B	TNPO3	Cas9	TNPO3.-3-S	AGAGCCTTCTGCCCTGGTAA	Alt-R Cas9 crRNA:tra	TNPO3 -3-S T	T*C*GCGCTTCCTCACTGTCTC	PS modified	NA	NA	ACACTCTTTCCCTACACGACGCTCTTCCGATCTCTCAAGAGTCTAGAATCAAGTGGAAA	GTGACTGGAGTTCAGACGTGTGCTCTTCCGATCTGGTAGCGACGGCTCTGAT
S1B	TNPO3	Cas9	TNPO3.-3-AS	CTCCTCTTTGGCCGTTACCA	Alt-R Cas9 crRNA:tra	TNPO3 -3-AS T	C*C*TGAATACGACCTAATAG	PS modified	NA	NA	ACACTCTTTCCCTACACGACGCTCTTCCGATCTCTCAAGAGTCTAGAATCAAGTGGAAA	GTGACTGGAGTTCAGACGTGTGCTCTTCCGATCTGGTAGCGACGGCTCTGAT
S1B	TNPO3	Cas9	TNPO3.-2-AS	CCTCCTCTTTGGCCGTTACC	Alt-R Cas9 crRNA:tra	TNPO3 -2-AS T	C*C*TGAATACGACCTAATAG	PS modified	NA	NA	ACACTCTTTCCCTACACGACGCTCTTCCGATCTCTCAAGAGTCTAGAATCAAGTGGAAA	GTGACTGGAGTTCAGACGTGTGCTCTTCCGATCTGGTAGCGACGGCTCTGAT
S1B	TNPO3	Cas9	TNPO3.+6-S	TGCCCTGGTAACGGCCAAAG	Alt-R Cas9 crRNA:tra	TNPO3 +6-S T	T*C*GCGCTTCCTCACTGTCTC	PS modified	NA	NA	ACACTCTTTCCCTACACGACGCTCTTCCGATCTCTCAAGAGTCTAGAATCAAGTGGAAA	GTGACTGGAGTTCAGACGTGTGCTCTTCCGATCTGGTAGCGACGGCTCTGAT
S1B	TNPO3	Cas9	TNPO3.+9-S	CCTGGTAACGGCCAAAGAGG	Alt-R Cas9 crRNA:tra	TNPO3 +9-S T	T*C*GCGCTTCCTCACTGTCTC	PS modified	NA	NA	ACACTCTTTCCCTACACGACGCTCTTCCGATCTCTCAAGAGTCTAGAATCAAGTGGAAA	GTGACTGGAGTTCAGACGTGTGCTCTTCCGATCTGGTAGCGACGGCTCTGAT
S1B	TNPO3	Cas9	TNPO3.+9-AS	TGGCGCCATCTCCTCCTCTT	Alt-R Cas9 crRNA:tra	TNPO3 +9-AS T	C*C*TGAATACGACCTAATAG	PS modified	NA	NA	ACACTCTTTCCCTACACGACGCTCTTCCGATCTCTCAAGAGTCTAGAATCAAGTGGAAA	GTGACTGGAGTTCAGACGTGTGCTCTTCCGATCTGGTAGCGACGGCTCTGAT
S1B	TNPO3	Cas9	TNPO3.+15-S	AACGGCCAAAGAGGAGGAGA	Alt-R Cas9 crRNA:tra	TNPO3 +15-S T	T*C*GCGCTTCCTCACTGTCTC	PS modified	NA	NA	ACACTCTTTCCCTACACGACGCTCTTCCGATCTCTCAAGAGTCTAGAATCAAGTGGAAA	GTGACTGGAGTTCAGACGTGTGCTCTTCCGATCTGGTAGCGACGGCTCTGAT
S1B	TNPO3	Cas9	TNPO3.+26-S	AGGAGGAGATGGCGCCAGTC	Alt-R Cas9 crRNA:tra	TNPO3 +26-S T	T*C*GCGCTTCCTCACTGTCTC	PS modified	NA	NA	ACACTCTTTCCCTACACGACGCTCTTCCGATCTCTCAAGAGTCTAGAATCAAGTGGAAA	GTGACTGGAGTTCAGACGTGTGCTCTTCCGATCTGGTAGCGACGGCTCTGAT
S2B	HPRT1	Cas9	HPRT1-38085-AS	TTATGGGGATTACTAGGAAG	Alt-R Cas9 crRNA:tra	NA			AAGAATGTTGTGATAAAAGGTGATGCT	ACACATCCATGGGACTTCTGCCTC	NA	NA
S2B	HPRT1	Cas9	HPRT1-38095-S	CCATTTCATAGTCTTTCCTT	Alt-R Cas9 crRNA:tra	NA			AAGAATGTTGTGATAAAAGGTGATGCT	ACACATCCATGGGACTTCTGCCTC	NA	NA
S2B	HPRT1	Cas9	HPRT1-38102-AS	TGAAATGGAGAGCTAAATTA	Alt-R Cas9 crRNA:tra	NA			AAGAATGTTGTGATAAAAGGTGATGCT	ACACATCCATGGGACTTCTGCCTC	NA	NA
S2B	HPRT1	Cas9	HPRT1-38115-S	GGGTGTGTTAAAAGTGACCA	Alt-R Cas9 crRNA:tra	NA			AAGAATGTTGTGATAAAAGGTGATGCT	ACACATCCATGGGACTTCTGCCTC	NA	NA
S2B	HPRT1	Cas9	HPRT1-38154-AS	CATCCGTGCTGAGTGTACCA	Alt-R Cas9 crRNA:tra	NA			AAGAATGTTGTGATAAAAGGTGATGCT	ACACATCCATGGGACTTCTGCCTC	NA	NA
S2B	HPRT1	Cas9	HPRT1-38221-AS	TAACATACAGAGAGACTACA	Alt-R Cas9 crRNA:tra	NA			AAGAATGTTGTGATAAAAGGTGATGCT	ACACATCCATGGGACTTCTGCCTC	NA	NA
S2B	HPRT1	Cas9	HPRT1-38239-S	TTAACAGCTTGCTGGTGAAA	Alt-R Cas9 crRNA:tra	NA			AAGAATGTTGTGATAAAAGGTGATGCT	ACACATCCATGGGACTTCTGCCTC	NA	NA
S2B	HPRT1	Cas9	HPRT1-38256-S	AAAAGGACCCCACGAAGTGT	Alt-R Cas9 crRNA:tra	NA			AAGAATGTTGTGATAAAAGGTGATGCT	ACACATCCATGGGACTTCTGCCTC	NA	NA
S2B	HPRT1	Cas9	HPRT1-38286-AS	GCTTATATCCAACACTTCGT	Alt-R Cas9 crRNA:tra	NA			AAGAATGTTGTGATAAAAGGTGATGCT	ACACATCCATGGGACTTCTGCCTC	NA	NA
S2B	HPRT1	Cas9	HPRT1-38308-AS	AAAGTAATTCACTTACAGTC	Alt-R Cas9 crRNA:tra	NA			AAGAATGTTGTGATAAAAGGTGATGCT	ACACATCCATGGGACTTCTGCCTC	NA	NA
S2B	HPRT1	Cas9	HPRT1-38338-S	TAAAAGAGTTTTATGTGAAA	Alt-R Cas9 crRNA:tra	NA			AAGAATGTTGTGATAAAAGGTGATGCT	ACACATCCATGGGACTTCTGCCTC	NA	NA
S2B	HPRT1	Cas9	HPRT1-38348-AS	AAACTCTTTTAGGTTAAAGA	Alt-R Cas9 crRNA:tra	NA			AAGAATGTTGTGATAAAAGGTGATGCT	ACACATCCATGGGACTTCTGCCTC	NA	NA
S2B	HPRT1	Cas9	HPRT1-38478-S	ATTCCAGACAAGTTTGTTGT	Alt-R Cas9 crRNA:tra	NA			AAGAATGTTGTGATAAAAGGTGATGCT	ACACATCCATGGGACTTCTGCCTC	NA	NA
S2B	HPRT1	Cas9	HPRT1-38503-AS	TATCCTACAACAAACTTGTC	Alt-R Cas9 crRNA:tra	NA			AAGAATGTTGTGATAAAAGGTGATGCT	ACACATCCATGGGACTTCTGCCTC	NA	NA
S2B	HPRT1	Cas9	HPRT1-38510-S	TGACTATAATGAATACTTCA	Alt-R Cas9 crRNA:tra	NA			AAGAATGTTGTGATAAAAGGTGATGCT	ACACATCCATGGGACTTCTGCCTC	NA	NA
S2B	HPRT1	Cas9	HPRT1-38529-AS	TGAAGTATTCATTATAGTCA	Alt-R Cas9 crRNA:tra	NA			AAGAATGTTGTGATAAAAGGTGATGCT	ACACATCCATGGGACTTCTGCCTC	NA	NA
S2B	HPRT1	Cas9	HPRT1-38574-S	CAAAACACGCATAAAAATTT	Alt-R Cas9 crRNA:tra	NA			AAGAATGTTGTGATAAAAGGTGATGCT	ACACATCCATGGGACTTCTGCCTC	NA	NA
S2B	HPRT1	Cas9	HPRT1-38626-S	TCATAATTTGAACAGACTGA	Alt-R Cas9 crRNA:tra	NA			AAGAATGTTGTGATAAAAGGTGATGCT	ACACATCCATGGGACTTCTGCCTC	NA	NA
S2B	HPRT1	Cas9	HPRT1-38640-AS	CTGTTCAAATTATGAGGTGC	Alt-R Cas9 crRNA:tra	NA			AAGAATGTTGTGATAAAAGGTGATGCT	ACACATCCATGGGACTTCTGCCTC	NA	NA
S2B	HPRT1	Cas9	HPRT1-38673-AS	TACAGCTTTATGTGACTAAT	Alt-R Cas9 crRNA:tra	NA			AAGAATGTTGTGATAAAAGGTGATGCT	ACACATCCATGGGACTTCTGCCTC	NA	NA
S2E	AAVS1	Cas9	AAVS1-46bp-PAM-out	AGAACCAGAGCCACATTAAC: C	Alt-R Cas9 crRNA:tra	AAVS1 46 PAM-out A Top	G*G*CTTTTCTGTCACCAATC	PS modified	GAATATGTCCCAGATAGCAC	CCCCGTTCTCCTGTGGATTC	NA	NA
S2E	AAVS1	Cas9	AAVS1-46bp-PAM-out	AGAACCAGAGCCACATTAAC: C	Alt-R Cas9 crRNA:tra	AAVS1 46 PAM-out A Btm	G*T*CCCCGAGCTGGGACCA	PS modified	GAATATGTCCCAGATAGCAC	CCCCGTTCTCCTGTGGATTC	NA	NA
S2E	AAVS1	Cas9	AAVS1-46bp-PAM-out	AGAACCAGAGCCACATTAAC: C	Alt-R Cas9 crRNA:tra	AAVS1 46 PAM-out B Top	G*G*CTTTTCTGTCACCAATC	PS modified	GAATATGTCCCAGATAGCAC	CCCCGTTCTCCTGTGGATTC	NA	NA
S2E	AAVS1	Cas9	AAVS1-46bp-PAM-out	AGAACCAGAGCCACATTAAC: C	Alt-R Cas9 crRNA:tra	AAVS1 46 PAM-out B Btm	G*T*CCCCGAGCTGGGACCA	PS modified	GAATATGTCCCAGATAGCAC	CCCCGTTCTCCTGTGGATTC	NA	NA
S2E	AAVS1	Cas9	AAVS1-46bp-PAM-out	AGAACCAGAGCCACATTAAC; C	Alt-R Cas9 crRNA:tra	AAVS1 46 PAM-out C Top	G*G*CTTTTCTGTCACCAATC	PS modified	GAATATGTCCCAGATAGCAC	CCCCGTTCTCCTGTGGATTC	NA	NA
S2E	AAVS1	Cas9	AAVS1-46bp-PAM-out	AGAACCAGAGCCACATTAAC; C	Alt-R Cas9 crRNA:tra	AAVS1 46 PAM-out C Btm	G*T*CCCCGAGCTGGGACCA	PS modified	GAATATGTCCCAGATAGCAC	CCCCGTTCTCCTGTGGATTC	NA	NA
S2E	AAVS1	Cas9	AAVS1-46bp-PAM-out	AGAACCAGAGCCACATTAAC; C	Alt-R Cas9 crRNA:tra	AAVS1 46 PAM-out D Top	G*G*CTTTTCTGTCACCAATC	PS modified	GAATATGTCCCAGATAGCAC	CCCCGTTCTCCTGTGGATTC	NA	NA
S2E	AAVS1	Cas9	AAVS1-46bp-PAM-out	AGAACCAGAGCCACATTAAC; C	Alt-R Cas9 crRNA:tra	AAVS1 46 PAM-out D Btm	G*T*CCCCGAGCTGGGACCA	PS modified	GAATATGTCCCAGATAGCAC	CCCCGTTCTCCTGTGGATTC	NA	NA
S2E	AAVS1	Cas9	AAVS1-46bp-PAM-out	AGAACCAGAGCCACATTAAC; C	Alt-R Cas9 crRNA:tra	AAVS1 46 PAM-out E Top	G*G*CTTTTCTGTCACCAATC	PS modified	GAATATGTCCCAGATAGCAC	CCCCGTTCTCCTGTGGATTC	NA	NA
S2E	AAVS1	Cas9	AAVS1-46bp-PAM-out	AGAACCAGAGCCACATTAAC; C	Alt-R Cas9 crRNA:tra	AAVS1 46 PAM-out E Btm	G*T*CCCCGAGCTGGGACCA	PS modified	GAATATGTCCCAGATAGCAC	CCCCGTTCTCCTGTGGATTC	NA	NA
S2E	AAVS1	Cas9	AAVS1-46bp-PAM-out	AGAACCAGAGCCACATTAAC: C	Alt-R Cas9 crRNA:tra	AAVS1 46 PAM-out F Top	G*G*CTTTTCTGTCACCAATC	PS modified	GAATATGTCCCAGATAGCAC	CCCCGTTCTCCTGTGGATTC	NA	NA
S2E	AAVS1	Cas9	AAVS1-46bp-PAM-out	AGAACCAGAGCCACATTAAC: C	Alt-R Cas9 crRNA:tra	AAVS1 46 PAM-out F Btm	G*T*CCCCGAGCTGGGACCA	PS modified	GAATATGTCCCAGATAGCAC	CCCCGTTCTCCTGTGGATTC	NA	NA
S2E	AAVS1	Cas9	AAVS1-46bp-PAM-out	AGAACCAGAGCCACATTAAC: C	Alt-R Cas9 crRNA:tra	AAVS1 46 PAM-out G Top	G*G*CTTTTCTGTCACCAATC	PS modified	GAATATGTCCCAGATAGCAC	CCCCGTTCTCCTGTGGATTC	NA	NA
S2E	AAVS1	Cas9	AAVS1-46bp-PAM-out	AGAACCAGAGCCACATTAAC: C	Alt-R Cas9 crRNA:tra	AAVS1 46 PAM-out G Btm	G*T*CCCCGAGCTGGGACCA	PS modified	GAATATGTCCCAGATAGCAC	CCCCGTTCTCCTGTGGATTC	NA	NA

S3A	SERPINC1	Cas9	SERPINC1-166	ACCTCTGGAAAAAGGTAAGA	Alt-R Cas9 crRNA:tra	SERPINC1-166 none	A*T*TCCAATGTGATAGGAAC	PS modified	NA	NA	ACACTCTTTCCCTACACGACGCTCTTCCGATCTCTGGTCTTCTCAAAGGTGTTG	GTGACTGGAGTTCAGACGTGTGCTCTTCCGATCTTTGCCTCAGATCACACTATCT
S3A	SERPINC1	Cas9	SERPINC1-166	ACCTCTGGAAAAAGGTAAGA	Alt-R Cas9 crRNA:tra	SERPINC1-166 1PAM	A*T*TCCAATGTGATAGGAAC	PS modified	NA	NA	ACACTCTTTCCCTACACGACGCTCTTCCGATCTCTGGTCTTCTCAAAGGTGTTG	GTGACTGGAGTTCAGACGTGTGCTCTTCCGATCTTTGCCTCAGATCACACTATCT
S3A	SERPINC1	Cas9	SERPINC1-166	ACCTCTGGAAAAAGGTAAGA	Alt-R Cas9 crRNA:tra	SERPINC1-166 2PAM	A*T*TCCAATGTGATAGGAAC	PS modified	NA	NA	ACACTCTTTCCCTACACGACGCTCTTCCGATCTCTGGTCTTCTCAAAGGTGTTG	GTGACTGGAGTTCAGACGTGTGCTCTTCCGATCTTTGCCTCAGATCACACTATCT
S3A	SERPINC1	Cas9	SERPINC1-166	ACCTCTGGAAAAAGGTAAGA	Alt-R Cas9 crRNA:tra	SERPINC1-166 1 seed A	A*T*TCCAATGTGATAGGAAC	PS modified	NA	NA	ACACTCTTTCCCTACACGACGCTCTTCCGATCTCTGGTCTTCTCAAAGGTGTTG	GTGACTGGAGTTCAGACGTGTGCTCTTCCGATCTTTGCCTCAGATCACACTATCT
S3A	SERPINC1	Cas9	SERPINC1-166	ACCTCTGGAAAAAGGTAAGA	Alt-R Cas9 crRNA:tra	SERPINC1-166 1 seed B	A*T*TCCAATGTGATAGGAAC	PS modified	NA	NA	ACACTCTTTCCCTACACGACGCTCTTCCGATCTCTGGTCTTCTCAAAGGTGTTG	GTGACTGGAGTTCAGACGTGTGCTCTTCCGATCTTTGCCTCAGATCACACTATCT
S3A	SERPINC1	Cas9	SERPINC1-166	ACCTCTGGAAAAAGGTAAGA	Alt-R Cas9 crRNA:tra	SERPINC1-166 2 seed	A*T*TCCAATGTGATAGGAAC	PS modified	NA	NA	ACACTCTTTCCCTACACGACGCTCTTCCGATCTCTGGTCTTCTCAAAGGTGTTG	GTGACTGGAGTTCAGACGTGTGCTCTTCCGATCTTTGCCTCAGATCACACTATCT
S3A	SERPINC1	Cas9	SERPINC1-166	ACCTCTGGAAAAAGGTAAGA	Alt-R Cas9 crRNA:tra	SERPINC1-166 1PAM + 1 seed A	A*T*TCCAATGTGATAGGAAC	PS modified	NA	NA	ACACTCTTTCCCTACACGACGCTCTTCCGATCTCTGGTCTTCTCAAAGGTGTTG	GTGACTGGAGTTCAGACGTGTGCTCTTCCGATCTTTGCCTCAGATCACACTATCT
S3A	SERPINC1	Cas9	SERPINC1-166	ACCTCTGGAAAAAGGTAAGA	Alt-R Cas9 crRNA:tra	SERPINC1-166 1PAM + 1 seed B	A*T*TCCAATGTGATAGGAAC	PS modified	NA	NA	ACACTCTTTCCCTACACGACGCTCTTCCGATCTCTGGTCTTCTCAAAGGTGTTG	GTGACTGGAGTTCAGACGTGTGCTCTTCCGATCTTTGCCTCAGATCACACTATCT
S3A	SERPINC1	Cas9	SERPINC1-166	ACCTCTGGAAAAAGGTAAGA	Alt-R Cas9 crRNA:tra	SERPINC1-166 2 PAM + 1 seed A	A*T*TCCAATGTGATAGGAAC	PS modified	NA	NA	ACACTCTTTCCCTACACGACGCTCTTCCGATCTCTGGTCTTCTCAAAGGTGTTG	GTGACTGGAGTTCAGACGTGTGCTCTTCCGATCTTTGCCTCAGATCACACTATCT
S3A	SERPINC1	Cas9	SERPINC1-166	ACCTCTGGAAAAAGGTAAGA	Alt-R Cas9 crRNA:tra	SERPINC1-166 1 PAM + 2 seed	A*T*TCCAATGTGATAGGAAC	PS modified	NA	NA	ACACTCTTTCCCTACACGACGCTCTTCCGATCTCTGGTCTTCTCAAAGGTGTTG	GTGACTGGAGTTCAGACGTGTGCTCTTCCGATCTTTGCCTCAGATCACACTATCT
S3A	SERPINC1	Cas9	SERPINC1-166	ACCTCTGGAAAAAGGTAAGA	Alt-R Cas9 crRNA:tra	SERPINC1-166 2 PAM + 2 seed	A*T*TCCAATGTGATAGGAAC	PS modified	NA	NA	ACACTCTTTCCCTACACGACGCTCTTCCGATCTCTGGTCTTCTCAAAGGTGTTG	GTGACTGGAGTTCAGACGTGTGCTCTTCCGATCTTTGCCTCAGATCACACTATCT
S3A	SAA1	Cas9	SAA1-88	AAGTTTTAAAAATCACTCCT	Alt-R Cas9 crRNA:tra	SAA1-88 none	T*G*TTGGGAAAGAGAAAAC	PS modified	NA	NA	ACACTCTTTCCCTACACGACGCTCTTCCGATCTGCTGTATGCCCCAGGATAA	GTGACTGGAGTTCAGACGTGTGCTCTTCCGATCTGACCAGCAAGGCTCACTATA
S3A	SAA1	Cas9	SAA1-88	AAGTTTTAAAAATCACTCCT	Alt-R Cas9 crRNA:tra	SAA1-88 1 PAM	T*G*TTGGGAAAGAGAAAAC	PS modified	NA	NA	ACACTCTTTCCCTACACGACGCTCTTCCGATCTGCTGTATGCCCCAGGATAA	GTGACTGGAGTTCAGACGTGTGCTCTTCCGATCTGACCAGCAAGGCTCACTATA
S3A	SAA1	Cas9	SAA1-88	AAGTTTTAAAAATCACTCCT	Alt-R Cas9 crRNA:tra	SAA1-88 2 PAM	T*G*TTGGGAAAGAGAAAAC	PS modified	NA	NA	ACACTCTTTCCCTACACGACGCTCTTCCGATCTGCTGTATGCCCCAGGATAA	GTGACTGGAGTTCAGACGTGTGCTCTTCCGATCTGACCAGCAAGGCTCACTATA
S3A	SAA1	Cas9	SAA1-88	AAGTTTTAAAAATCACTCCT	Alt-R Cas9 crRNA:tra	SAA1-88 1 seed A	T*G*TTGGGAAAGAGAAAAC	PS modified	NA	NA	ACACTCTTTCCCTACACGACGCTCTTCCGATCTGCTGTATGCCCCAGGATAA	GTGACTGGAGTTCAGACGTGTGCTCTTCCGATCTGACCAGCAAGGCTCACTATA
S3A	SAA1	Cas9	SAA1-88	AAGTTTTAAAAATCACTCCT	Alt-R Cas9 crRNA:tra	SAA1-88 1 seed B	T*G*TTGGGAAAGAGAAAAC	PS modified	NA	NA	ACACTCTTTCCCTACACGACGCTCTTCCGATCTGCTGTATGCCCCAGGATAA	GTGACTGGAGTTCAGACGTGTGCTCTTCCGATCTGACCAGCAAGGCTCACTATA
S3A	SAA1	Cas9	SAA1-88	AAGTTTTAAAAATCACTCCT	Alt-R Cas9 crRNA:tra	SAA1-88 1 PAM+1 seed A	T*G*TTGGGAAAGAGAAAAC	PS modified	NA	NA	ACACTCTTTCCCTACACGACGCTCTTCCGATCTGCTGTATGCCCCAGGATAA	GTGACTGGAGTTCAGACGTGTGCTCTTCCGATCTGACCAGCAAGGCTCACTATA
S3A	SAA1	Cas9	SAA1-88	AAGTTTTAAAAATCACTCCT	Alt-R Cas9 crRNA:tra	SAA1-88 1 PAM+1 seed B	T*G*TTGGGAAAGAGAAAAC	PS modified	NA	NA	ACACTCTTTCCCTACACGACGCTCTTCCGATCTGCTGTATGCCCCAGGATAA	GTGACTGGAGTTCAGACGTGTGCTCTTCCGATCTGACCAGCAAGGCTCACTATA
S3A	SAA1	Cas9	SAA1-88	AAGTTTTAAAAATCACTCCT	Alt-R Cas9 crRNA:tra	SAA1-88 2 PAM+1 seed A	T*G*TTGGGAAAGAGAAAAC	PS modified	NA	NA	ACACTCTTTCCCTACACGACGCTCTTCCGATCTGCTGTATGCCCCAGGATAA	GTGACTGGAGTTCAGACGTGTGCTCTTCCGATCTGACCAGCAAGGCTCACTATA
S3A	SAA1	Cas9	SAA1-88	AAGTTTTAAAAATCACTCCT	Alt-R Cas9 crRNA:tra	SAA1-88 1 PAM+2 seed	T*G*TTGGGAAAGAGAAAAC	PS modified	NA	NA	ACACTCTTTCCCTACACGACGCTCTTCCGATCTGCTGTATGCCCCAGGATAA	GTGACTGGAGTTCAGACGTGTGCTCTTCCGATCTGACCAGCAAGGCTCACTATA
S3A	SAA1	Cas9	SAA1-88	AAGTTTTAAAAATCACTCCT	Alt-R Cas9 crRNA:tra	SAA1-88 2 PAM+2 seed	T*G*TTGGGAAAGAGAAAAC	PS modified	NA	NA	ACACTCTTTCCCTACACGACGCTCTTCCGATCTGCTGTATGCCCCAGGATAA	GTGACTGGAGTTCAGACGTGTGCTCTTCCGATCTGACCAGCAAGGCTCACTATA
S3A	STAT3	Cas9	STAT3-105	CTTGGATTGAGAGTCAAGAT	Alt-R Cas9 crRNA:tra	STAT3-105 none	G*G*AGCTGCGGCAGTTTCTC	PS modified	NA	NA	ACACTCTTTCCCTACACGACGCTCTTCCGATCTGAACAGCAAGGCATGACATT	GTGACTGGAGTTCAGACGTGTGCTCTTCCGATCTCTCCATCAGCTCTACAGTGA
S3A	STAT3	Cas9	STAT3-105	CTTGGATTGAGAGTCAAGAT	Alt-R Cas9 crRNA:tra	STAT3-105 1 PAM	G*G*AGCTGCGGCAGTTTCTC	PS modified	NA	NA	ACACTCTTTCCCTACACGACGCTCTTCCGATCTGAACAGCAAGGCATGACATT	GTGACTGGAGTTCAGACGTGTGCTCTTCCGATCTCTCCATCAGCTCTACAGTGA
S3A	STAT3	Cas9	STAT3-105	CTTGGATTGAGAGTCAAGAT	Alt-R Cas9 crRNA:tra	STAT3-105 2 PAM	G*G*AGCTGCGGCAGTTTCTC	PS modified	NA	NA	ACACTCTTTCCCTACACGACGCTCTTCCGATCTGAACAGCAAGGCATGACATT	GTGACTGGAGTTCAGACGTGTGCTCTTCCGATCTCTCCATCAGCTCTACAGTGA
S3A	STAT3	Cas9	STAT3-105	CTTGGATTGAGAGTCAAGAT	Alt-R Cas9 crRNA:tra	STAT3-105 1 seed A	G*G*AGCTGCGGCAGTTTCTC	PS modified	NA	NA	ACACTCTTTCCCTACACGACGCTCTTCCGATCTGAACAGCAAGGCATGACATT	GTGACTGGAGTTCAGACGTGTGCTCTTCCGATCTCTCCATCAGCTCTACAGTGA
S3A	STAT3	Cas9	STAT3-105	CTTGGATTGAGAGTCAAGAT	Alt-R Cas9 crRNA:tra	STAT3-105 1 seed B	G*G*AGCTGCGGCAGTTTCTC	PS modified	NA	NA	ACACTCTTTCCCTACACGACGCTCTTCCGATCTGAACAGCAAGGCATGACATT	GTGACTGGAGTTCAGACGTGTGCTCTTCCGATCTCTCCATCAGCTCTACAGTGA
S3A	STAT3	Cas9	STAT3-105	CTTGGATTGAGAGTCAAGAT	Alt-R Cas9 crRNA:tra	STAT3-105 2 seed	G*G*AGCTGCGGCAGTTTCTC	PS modified	NA	NA	ACACTCTTTCCCTACACGACGCTCTTCCGATCTGAACAGCAAGGCATGACATT	GTGACTGGAGTTCAGACGTGTGCTCTTCCGATCTCTCCATCAGCTCTACAGTGA
S3A	STAT3	Cas9	STAT3-105	CTTGGATTGAGAGTCAAGAT	Alt-R Cas9 crRNA:tra	STAT3-105 1 PAM+1 seed A	G*G*AGCTGCGGCAGTTTCTC	PS modified	NA	NA	ACACTCTTTCCCTACACGACGCTCTTCCGATCTGAACAGCAAGGCATGACATT	GTGACTGGAGTTCAGACGTGTGCTCTTCCGATCTCTCCATCAGCTCTACAGTGA
S3A	STAT3	Cas9	STAT3-105	CTTGGATTGAGAGTCAAGAT	Alt-R Cas9 crRNA:tra	STAT3-105 1 PAM+1 seed B	G*G*AGCTGCGGCAGTTTCTC	PS modified	NA	NA	ACACTCTTTCCCTACACGACGCTCTTCCGATCTGAACAGCAAGGCATGACATT	GTGACTGGAGTTCAGACGTGTGCTCTTCCGATCTCTCCATCAGCTCTACAGTGA
S3A	STAT3	Cas9	STAT3-105	CTTGGATTGAGAGTCAAGAT	Alt-R Cas9 crRNA:tra	STAT3-105 2 PAM+1 seed A	G*G*AGCTGCGGCAGTTTCTC	PS modified	NA	NA	ACACTCTTTCCCTACACGACGCTCTTCCGATCTGAACAGCAAGGCATGACATT	GTGACTGGAGTTCAGACGTGTGCTCTTCCGATCTCTCCATCAGCTCTACAGTGA
S3A	STAT3	Cas9	STAT3-105	CTTGGATTGAGAGTCAAGAT	Alt-R Cas9 crRNA:tra	STAT3-105 1 PAM+2 seed	G*G*AGCTGCGGCAGTTTCTC	PS modified	NA	NA	ACACTCTTTCCCTACACGACGCTCTTCCGATCTGAACAGCAAGGCATGACATT	GTGACTGGAGTTCAGACGTGTGCTCTTCCGATCTCTCCATCAGCTCTACAGTGA
S3A	STAT3	Cas9	STAT3-105	CTTGGATTGAGAGTCAAGAT	Alt-R Cas9 crRNA:tra	STAT3-105 2 PAM+2 seed	G*G*AGCTGCGGCAGTTTCTC	PS modified	NA	NA	ACACTCTTTCCCTACACGACGCTCTTCCGATCTGAACAGCAAGGCATGACATT	GTGACTGGAGTTCAGACGTGTGCTCTTCCGATCTCTCCATCAGCTCTACAGTGA
S3A	HPRT1	Cas9	HPRT-38087	AATTATGGGGATTACTAGGA	Alt-R Cas9 crRNA:tra	HPRT 38087 1 PAM	A*A*AGACTATGAAATGGAG	PS modified	NA	NA	ACACTCTTTCCCTACACGACGCTCTTCCGATCTCAGAACTGTCCTTCAGGTTC	GTGACTGGAGTTCAGACGTGTGCTCTTCCGATCTCACTGTTTCATTTCATCCGTG
S3A	HPRT1	Cas9	HPRT-38087	AATTATGGGGATTACTAGGA	Alt-R Cas9 crRNA:tra	HPRT 38087 2 PAM	A*A*AGACTATGAAATGGAG	PS modified	NA	NA	ACACTCTTTCCCTACACGACGCTCTTCCGATCTCAGAACTGTCCTTCAGGTTC	GTGACTGGAGTTCAGACGTGTGCTCTTCCGATCTCACTGTTTCATTTCATCCGTG
S3A	HPRT1	Cas9	HPRT-38087	AATTATGGGGATTACTAGGA	Alt-R Cas9 crRNA:tra	HPRT 38087 1 seed A	A*A*AGACTATGAAATGGAG	PS modified	NA	NA	ACACTCTTTCCCTACACGACGCTCTTCCGATCTCAGAACTGTCCTTCAGGTTC	GTGACTGGAGTTCAGACGTGTGCTCTTCCGATCTCACTGTTTCATTTCATCCGTG
S3A	HPRT1	Cas9	HPRT-38087	AATTATGGGGATTACTAGGA	Alt-R Cas9 crRNA:tra	HPRT 38087 1 seed B	A*A*AGACTATGAAATGGAG	PS modified	NA	NA	ACACTCTTTCCCTACACGACGCTCTTCCGATCTCAGAACTGTCCTTCAGGTTC	GTGACTGGAGTTCAGACGTGTGCTCTTCCGATCTCACTGTTTCATTTCATCCGTG
S3A	HPRT1	Cas9	HPRT-38087	AATTATGGGGATTACTAGGA	Alt-R Cas9 crRNA:tra	HPRT 38087 2 seed	A*A*AGACTATGAAATGGAG	PS modified	NA	NA	ACACTCTTTCCCTACACGACGCTCTTCCGATCTCAGAACTGTCCTTCAGGTTC	GTGACTGGAGTTCAGACGTGTGCTCTTCCGATCTCACTGTTTCATTTCATCCGTG
S3A	HPRT1	Cas9	HPRT-38087	AATTATGGGGATTACTAGGA	Alt-R Cas9 crRNA:tra	HPRT 38087 1 PAM+1 seed A	A*A*AGACTATGAAATGGAG	PS modified	NA	NA	ACACTCTTTCCCTACACGACGCTCTTCCGATCTCAGAACTGTCCTTCAGGTTC	GTGACTGGAGTTCAGACGTGTGCTCTTCCGATCTCACTGTTTCATTTCATCCGTG
S3A	HPRT1	Cas9	HPRT-38087	AATTATGGGGATTACTAGGA	Alt-R Cas9 crRNA:tra	HPRT 38087 1 PAM+1 seed B	A*A*AGACTATGAAATGGAG	PS modified	NA	NA	ACACTCTTTCCCTACACGACGCTCTTCCGATCTCAGAACTGTCCTTCAGGTTC	GTGACTGGAGTTCAGACGTGTGCTCTTCCGATCTCACTGTTTCATTTCATCCGTG
S3A	HPRT1	Cas9	HPRT-38087	AATTATGGGGATTACTAGGA	Alt-R Cas9 crRNA:tra	HPRT 38087 2 PAM+1 seed A	A*A*AGACTATGAAATGGAG	PS modified	NA	NA	ACACTCTTTCCCTACACGACGCTCTTCCGATCTCAGAACTGTCCTTCAGGTTC	GTGACTGGAGTTCAGACGTGTGCTCTTCCGATCTCACTGTTTCATTTCATCCGTG
S3A	HPRT1	Cas9	HPRT-38087	AATTATGGGGATTACTAGGA	Alt-R Cas9 crRNA:tra	HPRT 38087 1 PAM+2 seed	A*A*AGACTATGAAATGGAG	PS modified	NA	NA	ACACTCTTTCCCTACACGACGCTCTTCCGATCTCAGAACTGTCCTTCAGGTTC	GTGACTGGAGTTCAGACGTGTGCTCTTCCGATCTCACTGTTTCATTTCATCCGTG
S3A	HPRT1	Cas9	HPRT-38087	AATTATGGGGATTACTAGGA	Alt-R Cas9 crRNA:tra	HPRT 38087 2 PAM+2 seed	A*A*AGACTATGAAATGGAG	PS modified	NA	NA	ACACTCTTTCCCTACACGACGCTCTTCCGATCTCAGAACTGTCCTTCAGGTTC	GTGACTGGAGTTCAGACGTGTGCTCTTCCGATCTCACTGTTTCATTTCATCCGTG
S3C	HPRT1	Cas9	HPRT-38087	AATTATGGGGATTACTAGGA	Alt-R Cas9 crRNA:tra	HPRT 38087 EcoRI -45 mutPAM	T*C*ATCCGTGCTGAGTGTAC	PS modified	AAGAATGTTGTGATAAAAGGTGATGCT	ACACATCCATGGGACTTCTGCCTC	NA	NA
S3C	HPRT1	Cas9	HPRT-38087	AATTATGGGGATTACTAGGA	Alt-R Cas9 crRNA:tra	HPRT 38087 EcoRI -35 mutPAM	T*G*AGTGTACCATGGTCACT	PS modified	AAGAATGTTGTGATAAAAGGTGATGCT	ACACATCCATGGGACTTCTGCCTC	NA	NA
S3C	HPRT1	Cas9	HPRT-38087	AATTATGGGGATTACTAGGA	Alt-R Cas9 crRNA:tra	HPRT 38087 EcoRI -25 mutPAM	A*T*GGTCACTTTTAACACAC	PS modified	AAGAATGTTGTGATAAAAGGTGATGCT	ACACATCCATGGGACTTCTGCCTC	NA	NA
S3C	HPRT1	Cas9	HPRT-38087	AATTATGGGGATTACTAGGA	Alt-R Cas9 crRNA:tra	HPRT 38087 EcoRI -20 mutPAM	C*A*CTTTTAACACACCCAAG	PS modified	AAGAATGTTGTGATAAAAGGTGATGCT	ACACATCCATGGGACTTCTGCCTC	NA	NA
S3C	HPRT1	Cas9	HPRT-38087	AATTATGGGGATTACTAGGA	Alt-R Cas9 crRNA:tra	HPRT 38087 EcoRI -15 mutPAM	T*T*AACACACCCAAGGAAAC	PS modified	AAGAATGTTGTGATAAAAGGTGATGCT	ACACATCCATGGGACTTCTGCCTC	NA	NA
S3C	HPRT1	Cas9	HPRT-38087	AATTATGGGGATTACTAGGA	Alt-R Cas9 crRNA:tra	HPRT 38087 EcoRI -10 mutPAM	A*C*ACCCAAGGAAAGACTA	PS modified	AAGAATGTTGTGATAAAAGGTGATGCT	ACACATCCATGGGACTTCTGCCTC	NA	NA
S3C	HPRT1	Cas9	HPRT-38087	AATTATGGGGATTACTAGGA	Alt-R Cas9 crRNA:tra	HPRT 38087 EcoRI -6 mutPAM	A*A*AGACTATGAAATGGAG	PS modified	AAGAATGTTGTGATAAAAGGTGATGCT	ACACATCCATGGGACTTCTGCCTC	NA	NA
S3C	HPRT1	Cas9	HPRT-38087	AATTATGGGGATTACTAGGA	Alt-R Cas9 crRNA:tra	HPRT 38087 EcoRI -3 mutPAM	A*A*AGACTATGAAATGGAG	PS modified	AAGAATGTTGTGATAAAAGGTGATGCT	ACACATCCATGGGACTTCTGCCTC	NA	NA
S3C	HPRT1	Cas9	HPRT-38087	AATTATGGGGATTACTAGGA	Alt-R Cas9 crRNA:tra	HPRT 38087 EcoRI +3 mutPAM	A*A*AGACTATGAAATGGAG	PS modified	AAGAATGTTGTGATAAAAGGTGATGCT	ACACATCCATGGGACTTCTGCCTC	NA	NA
S3C	HPRT1	Cas9	HPRT-38087	AATTATGGGGATTACTAGGA	Alt-R Cas9 crRNA:tra	HPRT 38087 EcoRI +6 mutPAM	A*A*AGACTATGAAATGGAG	PS modified	AAGAATGTTGTGATAAAAGGTGATGCT	ACACATCCATGGGACTTCTGCCTC	NA	NA
S3C	HPRT1	Cas9	HPRT-38087	AATTATGGGGATTACTAGGA	Alt-R Cas9 crRNA:tra	HPRT 38087 EcoRI +10 mutPAM	A*A*AGACTATGAAATGGAG	PS modified	AAGAATGTTGTGATAAAAGGTGATGCT	ACACATCCATGGGACTTCTGCCTC	NA	NA
S3C	HPRT1	Cas9	HPRT-38087	AATTATGGGGATTACTAGGA	Alt-R Cas9 crRNA:tra	HPRT 38087 EcoRI +15 mutPAM	A*A*AGACTATGAAATGGAG	PS modified	AAGAATGTTGTGATAAAAGGTGATGCT	ACACATCCATGGGACTTCTGCCTC	NA	NA
S3C	HPRT1	Cas9	HPRT-38087	AATTATGGGGATTACTAGGA	Alt-R Cas9 crRNA:tra	HPRT 38087 EcoRI +20 mutPAM	A*A*AGACTATGAAATGGAG	PS modified	AAGAATGTTGTGATAAAAGGTGATGCT	ACACATCCATGGGACTTCTGCCTC	NA	NA
S3C	HPRT1	Cas9	HPRT-38087	AATTATGGGGATTACTAGGA	Alt-R Cas9 crRNA:tra	HPRT 38087 EcoRI +25 mutPAM	A*A*AGACTATGAAATGGAG	PS modified	AAGAATGTTGTGATAAAAGGTGATGCT	ACACATCCATGGGACTTCTGCCTC	NA	NA
S3C	HPRT1	Cas9	HPRT-38087	AATTATGGGGATTACTAGGA	Alt-R Cas9 crRNA:tra	HPRT 38087 EcoRI +35 mutPAM	A*A*AGACTATGAAATGGAG	PS modified	AAGAATGTTGTGATAAAAGGTGATGCT	ACACATCCATGGGACTTCTGCCTC	NA	NA
S3C	HPRT1	Cas9	HPRT-38087	AATTATGGGGATTACTAGGA	Alt-R Cas9 crRNA:tra	HPRT 38087 EcoRI +45 mutPAM	A*A*AGACTATGAAATGGAG	PS modified	AAGAATGTTGTGATAAAAGGTGATGCT	ACACATCCATGGGACTTCTGCCTC	NA	NA
S3C	SAA1	Cas9	SAA1-88	AAGTTTTAAAAATCACTCCT	Alt-R Cas9 crRNA:tra	SAA1-88 EcoRI -45 mutPAM	T*G*TATGCCCCAGGATAACT	PS modified	TGCCCTCACTTCACACCTTC	GGTCCAGGGTGATGTTTCTCTT	NA	NA
S3C	SAA1	Cas9	SAA1-88	AAGTTTTAAAAATCACTCCT	Alt-R Cas9 crRNA:tra	SAA1-88 EcoRI -35 mutPAM	A*G*GATAACTATTTTTTTTAA	PS modified	TGCCCTCACTTCACACCTTC	GGTCCAGGGTGATGTTTCTCTT	NA	NA
S3C	SAA1	Cas9	SAA1-88	AAGTTTTAAAAATCACTCCT	Alt-R Cas9 crRNA:tra	SAA1-88 EcoRI -25 mutPAM	T*T*TTTTTTAAAGGAAATGA	PS modified	TGCCCTCACTTCACACCTTC	GGTCCAGGGTGATGTTTCTCTT	NA	NA
S3C	SAA1	Cas9	SAA1-88	AAGTTTTAAAAATCACTCCT	Alt-R Cas9 crRNA:tra	SAA1-88 EcoRI -20 mutPAM	T*T*TAAAGGAAATGATAATC	PS modified	TGCCCTCACTTCACACCTTC	GGTCCAGGGTGATGTTTCTCTT	NA	NA
S3C	SAA1	Cas9	SAA1-88	AAGTTTTAAAAATCACTCCT	Alt-R Cas9 crRNA:tra	SAA1-88 EcoRI -15 mutPAM	A*G*GAAATGATAATCTTGTT	PS modified	TGCCCTCACTTCACACCTTC	GGTCCAGGGTGATGTTTCTCTT	NA	NA
S3C	SAA1	Cas9	SAA1-88	AAGTTTTAAAAATCACTCCT	Alt-R Cas9 crRNA:tra	SAA1-88 EcoRI -10 mutPAM	A*T*GATAATCTTGTTGGGAA	PS modified	TGCCCTCACTTCACACCTTC	GGTCCAGGGTGATGTTTCTCTT	NA	NA
S3C	SAA1	Cas9	SAA1-88	AAGTTTTAAAAATCACTCCT	Alt-R Cas9 crRNA:tra	SAA1-88 EcoRI -6 mutPAM	T*G*TTGGGAAAGAGAAAAC	PS modified	TGCCCTCACTTCACACCTTC	GGTCCAGGGTGATGTTTCTCTT	NA	NA
S3C	SAA1	Cas9	SAA1-88	AAGTTTTAAAAATCACTCCT	Alt-R Cas9 crRNA:tra	SAA1-88 EcoRI -3 mutPAM	T*G*TTGGGAAAGAGAAAAC	PS modified	TGCCCTCACTTCACACCTTC	GGTCCAGGGTGATGTTTCTCTT	NA	NA
S3C	SAA1	Cas9	SAA1-88	AAGTTTTAAAAATCACTCCT	Alt-R Cas9 crRNA:tra	SAA1-88 EcoRI +3 mutPAM	T*G*TTGGGAAAGAGAAAAC	PS modified	TGCCCTCACTTCACACCTTC	GGTCCAGGGTGATGTTTCTCTT	NA	NA
S3C	SAA1	Cas9	SAA1-88	AAGTTTTAAAAATCACTCCT	Alt-R Cas9 crRNA:tra	SAA1-88 EcoRI +6 mutPAM	T*G*TTGGGAAAGAGAAAAC	PS modified	TGCCCTCACTTCACACCTTC	GGTCCAGGGTGATGTTTCTCTT	NA	NA
S3C	SAA1	Cas9	SAA1-88	AAGTTTTAAAAATCACTCCT	Alt-R Cas9 crRNA:tra	SAA1-88 EcoRI +10 mutPAM	T*G*TTGGGAAAGAGAAAAC	PS modified	TGCCCTCACTTCACACCTTC	GGTCCAGGGTGATGTTTCTCTT	NA	NA
S3C	SAA1	Cas9	SAA1-88	AAGTTTTAAAAATCACTCCT	Alt-R Cas9 crRNA:tra	SAA1-88 EcoRI +15 mutPAM	T*G*TTGGGAAAGAGAAAAC	PS modified	TGCCCTCACTTCACACCTTC	GGTCCAGGGTGATGTTTCTCTT	NA	NA
S3C	SAA1	Cas9	SAA1-88	AAGTTTTAAAAATCACTCCT	Alt-R Cas9 crRNA:tra	SAA1-88 EcoRI +20 mutPAM	T*G*TTGGGAAAGAGAAAAC	PS modified	TGCCCTCACTTCACACCTTC	GGTCCAGGGTGATGTTTCTCTT	NA	NA
S3C	SAA1	Cas9	SAA1-88	AAGTTTTAAAAATCACTCCT	Alt-R Cas9 crRNA:tra	SAA1-88 EcoRI +25 mutPAM	T*G*TTGGGAAAGAGAAAAC	PS modified	TGCCCTCACTTCACACCTTC	GGTCCAGGGTGATGTTTCTCTT	NA	NA
S3C	SAA1	Cas9	SAA1-88	AAGTTTTAAAAATCACTCCT	Alt-R Cas9 crRNA:tra	SAA1-88 EcoRI +35 mutPAM	T*G*TTGGGAAAGAGAAAAC	PS modified	TGCCCTCACTTCACACCTTC	GGTCCAGGGTGATGTTTCTCTT	NA	NA
S3C	SAA1	Cas9	SAA1-88	AAGTTTTAAAAATCACTCCT	Alt-R Cas9 crRNA:tra	SAA1-88 EcoRI +45 mutPAM	T*G*TTGGGAAAGAGAAAAC	PS modified	TGCCCTCACTTCACACCTTC	GGTCCAGGGTGATGTTTCTCTT	NA	NA
S3C	SERPINC1	Cas9	SERPINC1-72	AGTGGAGATAGTGTGATCTG	Alt-R Cas9 crRNA:tra	SERPINC1-AS-72 EcoRI -27 mutPAM	T*T*CCTATCACATTGGAATA	PS modified	CTTGTCCCTCTTTGCCTTCTCT	GGGTGGATCTGAGTGGAAGAAA	NA	NA
S3C	SERPINC1	Cas9	SERPINC1-72	AGTGGAGATAGTGTGATCTG	Alt-R Cas9 crRNA:tra	SERPINC1-AS-72 EcoRI -21 mutPAM	T*C*ACATTGGAATACATGGC	PS modified	CTTGTCCCTCTTTGCCTTCTCT	GGGTGGATCTGAGTGGAAGAAA	NA	NA
S3C	SERPINC1	Cas9	SERPINC1-72	AGTGGAGATAGTGTGATCTG	Alt-R Cas9 crRNA:tra	SERPINC1-AS-72 EcoRI -15 mutPAM	T*G*GAATACATGGCCGCTAA	PS modified	CTTGTCCCTCTTTGCCTTCTCT	GGGTGGATCTGAGTGGAAGAAA	NA	NA
S3C	SERPINC1	Cas9	SERPINC1-72	AGTGGAGATAGTGTGATCTG	Alt-R Cas9 crRNA:tra	SERPINC1-AS-72 EcoRI -10 mutPAM	T*A*CATGGCCGCTAATCTTC	PS modified	CTTGTCCCTCTTTGCCTTCTCT	GGGTGGATCTGAGTGGAAGAAA	NA	NA
S3C	SERPINC1	Cas9	SERPINC1-72	AGTGGAGATAGTGTGATCTG	Alt-R Cas9 crRNA:tra	SERPINC1-AS-72 EcoRI -6 mutPAM	C*T*AATCTTCCACAGGGCTG	PS modified	CTTGTCCCTCTTTGCCTTCTCT	GGGTGGATCTGAGTGGAAGAAA	NA	NA
S3C	SERPINC1	Cas9	SERPINC1-72	AGTGGAGATAGTGTGATCTG	Alt-R Cas9 crRNA:tra	SERPINC1-AS-72 EcoRI -3 mutPAM	C*T*AATCTTCCACAGGGCTG	PS modified	CTTGTCCCTCTTTGCCTTCTCT	GGGTGGATCTGAGTGGAAGAAA	NA	NA
S3C	SERPINC1	Cas9	SERPINC1-72	AGTGGAGATAGTGTGATCTG	Alt-R Cas9 crRNA:tra	SERPINC1-AS-72 EcoRI +3 mutPAM	C*T*AATCTTCCACAGGGCTG	PS modified	CTTGTCCCTCTTTGCCTTCTCT	GGGTGGATCTGAGTGGAAGAAA	NA	NA
S3C	SERPINC1	Cas9	SERPINC1-72	AGTGGAGATAGTGTGATCTG	Alt-R Cas9 crRNA:tra	SERPINC1-AS-72 EcoRI +6 mutPAM	C*T*AATCTTCCACAGGGCTG	PS modified	CTTGTCCCTCTTTGCCTTCTCT	GGGTGGATCTGAGTGGAAGAAA	NA	NA
S3C	SERPINC1	Cas9	SERPINC1-72	AGTGGAGATAGTGTGATCTG	Alt-R Cas9 crRNA:tra	SERPINC1-AS-72 EcoRI +10 mutPAM	C*T*AATCTTCCACAGGGCTG	PS modified	CTTGTCCCTCTTTGCCTTCTCT	GGGTGGATCTGAGTGGAAGAAA	NA	NA
S3C	SERPINC1	Cas9	SERPINC1-72	AGTGGAGATAGTGTGATCTG	Alt-R Cas9 crRNA:tra	SERPINC1-AS-72 EcoRI +15 mutPAM	C*T*AATCTTCCACAGGGCTG	PS modified	CTTGTCCCTCTTTGCCTTCTCT	GGGTGGATCTGAGTGGAAGAAA	NA	NA
S3C	SERPINC1	Cas9	SERPINC1-72	AGTGGAGATAGTGTGATCTG	Alt-R Cas9 crRNA:tra	SERPINC1-AS-72 EcoRI +21 mutPAM	C*T*AATCTTCCACAGGGCTG	PS modified	CTTGTCCCTCTTTGCCTTCTCT	GGGTGGATCTGAGTGGAAGAAA	NA	NA
S3C	SERPINC1	Cas9	SERPINC1-72	AGTGGAGATAGTGTGATCTG	Alt-R Cas9 crRNA:tra	SERPINC1-AS-72 EcoRI +27 mutPAM	C*T*AATCTTCCACAGGGCTG	PS modified	CTTGTCCCTCTTTGCCTTCTCT	GGGTGGATCTGAGTGGAAGAAA	NA	NA
S3C	SERPINC1	Cas9	SERPINC1-166	ACCTCTGGAAAAAGGTAAGA	Alt-R Cas9 crRNA:tra	SERPINC1-S-166 EcoRI -45 mutPAM	C*A*CTATCTCCACTTGCCCAC	PS modified	CTTGTCCCTCTTTGCCTTCTCT	GGGTGGATCTGAGTGGAAGAAA	NA	NA
S3C	SERPINC1	Cas9	SERPINC1-166	ACCTCTGGAAAAAGGTAAGA	Alt-R Cas9 crRNA:tra	SERPINC1-S-166 EcoRI -35 mutPAM	A*C*TTGCCCAGCCCTGTGGA	PS modified	CTTGTCCCTCTTTGCCTTCTCT	GGGTGGATCTGAGTGGAAGAAA	NA	NA
S3C	SERPINC1	Cas9	SERPINC1-166	ACCTCTGGAAAAAGGTAAGA	Alt-R Cas9 crRNA:tra	SERPINC1-S-166 EcoRI -25 mutPAM	C*C*CTGTGGAAGATTAGCGC	PS modified	CTTGTCCCTCTTTGCCTTCTCT	GGGTGGATCTGAGTGGAAGAAA	NA	NA
S3C	SERPINC1	Cas9	SERPINC1-166	ACCTCTGGAAAAAGGTAAGA	Alt-R Cas9 crRNA:tra	SERPINC1-S-166 EcoRI -20 mutPAM	T*G*GAAGATTAGCGGCCATC	PS modified	CTTGTCCCTCTTTGCCTTCTCT	GGGTGGATCTGAGTGGAAGAAA	NA	NA
S3C	SERPINC1	Cas9	SERPINC1-166	ACCTCTGGAAAAAGGTAAGA	Alt-R Cas9 crRNA:tra	SERPINC1-S-166 EcoRI -15 mutPAM	G*A*TTAGCGGCCATGTATTC	PS modified	CTTGTCCCTCTTTGCCTTCTCT	GGGTGGATCTGAGTGGAAGAAA	NA	NA
S3C	SERPINC1	Cas9	SERPINC1-166	ACCTCTGGAAAAAGGTAAGA	Alt-R Cas9 crRNA:tra	SERPINC1-S-166 EcoRI -10 mutPAM	G*C*GGCCATGTATTCCAATG	PS modified	CTTGTCCCTCTTTGCCTTCTCT	GGGTGGATCTGAGTGGAAGAAA	NA	NA
S3C	SERPINC1	Cas9	SERPINC1-166	ACCTCTGGAAAAAGGTAAGA	Alt-R Cas9 crRNA:tra	SERPINC1-S-166 EcoRI -6 mutPAM	A*T*TCCAATGTGATAGGAAC	PS modified	CTTGTCCCTCTTTGCCTTCTCT	GGGTGGATCTGAGTGGAAGAAA	NA	NA
S3C	SERPINC1	Cas9	SERPINC1-166	ACCTCTGGAAAAAGGTAAGA	Alt-R Cas9 crRNA:tra	SERPINC1-S-166 EcoRI -3 mutPAM	A*T*TCCAATGTGATAGGAAC	PS modified	CTTGTCCCTCTTTGCCTTCTCT	GGGTGGATCTGAGTGGAAGAAA	NA	NA
S3C	SERPINC1	Cas9	SERPINC1-166	ACCTCTGGAAAAAGGTAAGA	Alt-R Cas9 crRNA:tra	SERPINC1-S-166 EcoRI +3 mutPAM	A*T*TCCAATGTGATAGGAAC	PS modified	CTTGTCCCTCTTTGCCTTCTCT	GGGTGGATCTGAGTGGAAGAAA	NA	NA
S3C	SERPINC1	Cas9	SERPINC1-166	ACCTCTGGAAAAAGGTAAGA	Alt-R Cas9 crRNA:tra	SERPINC1-S-166 EcoRI +6 mutPAM	A*T*TCCAATGTGATAGGAAC	PS modified	CTTGTCCCTCTTTGCCTTCTCT	GGGTGGATCTGAGTGGAAGAAA	NA	NA
S3C	SERPINC1	Cas9	SERPINC1-166	ACCTCTGGAAAAAGGTAAGA	Alt-R Cas9 crRNA:tra	SERPINC1-S-166 EcoRI +10 mutPAM	A*T*TCCAATGTGATAGGAAC	PS modified	CTTGTCCCTCTTTGCCTTCTCT	GGGTGGATCTGAGTGGAAGAAA	NA	NA
S3C	SERPINC1	Cas9	SERPINC1-166	ACCTCTGGAAAAAGGTAAGA	Alt-R Cas9 crRNA:tra	SERPINC1-S-166 EcoRI +15 mutPAM	A*T*TCCAATGTGATAGGAAC	PS modified	CTTGTCCCTCTTTGCCTTCTCT	GGGTGGATCTGAGTGGAAGAAA	NA	NA
S3C	SERPINC1	Cas9	SERPINC1-166	ACCTCTGGAAAAAGGTAAGA	Alt-R Cas9 crRNA:tra	SERPINC1-S-166 EcoRI +20 mutPAM	A*T*TCCAATGTGATAGGAAC	PS modified	CTTGTCCCTCTTTGCCTTCTCT	GGGTGGATCTGAGTGGAAGAAA	NA	NA
S3C	SERPINC1	Cas9	SERPINC1-166	ACCTCTGGAAAAAGGTAAGA	Alt-R Cas9 crRNA:tra	SERPINC1-S-166 EcoRI +25 mutPAM	A*T*TCCAATGTGATAGGAAC	PS modified	CTTGTCCCTCTTTGCCTTCTCT	GGGTGGATCTGAGTGGAAGAAA	NA	NA
S3C	SERPINC1	Cas9	SERPINC1-166	ACCTCTGGAAAAAGGTAAGA	Alt-R Cas9 crRNA:tra	SERPINC1-S-166 EcoRI +35 mutPAM	A*T*TCCAATGTGATAGGAAC	PS modified	CTTGTCCCTCTTTGCCTTCTCT	GGGTGGATCTGAGTGGAAGAAA	NA	NA
S3C	SERPINC1	Cas9	SERPINC1-166	ACCTCTGGAAAAAGGTAAGA	Alt-R Cas9 crRNA:tra	SERPINC1-S-166 EcoRI +45 mutPAM	A*T*TCCAATGTGATAGGAAC	PS modified	CTTGTCCCTCTTTGCCTTCTCT	GGGTGGATCTGAGTGGAAGAAA	NA	NA
S3D	HPRT1	Cas9	HPRT-38087	AATTATGGGGATTACTAGGA	Alt-R Cas9 crRNA:tra	HPRT-38087 EcoRI -45	T*C*ATCCGTGCTGAGTGTAC	PS modified	AAGAATGTTGTGATAAAAGGTGATGCT	ACACATCCATGGGACTTCTGCCTC	NA	NA
S3D	HPRT1	Cas9	HPRT-38087	AATTATGGGGATTACTAGGA	Alt-R Cas9 crRNA:tra	HPRT-38087 EcoRI -35	T*G*AGTGTACCATGGTCACT	PS modified	AAGAATGTTGTGATAAAAGGTGATGCT	ACACATCCATGGGACTTCTGCCTC	NA	NA
S3D	HPRT1	Cas9	HPRT-38087	AATTATGGGGATTACTAGGA	Alt-R Cas9 crRNA:tra	HPRT-38087 EcoRI -25	A*T*GGTCACTTTTAACACAC	PS modified	AAGAATGTTGTGATAAAAGGTGATGCT	ACACATCCATGGGACTTCTGCCTC	NA	NA
S3D	HPRT1	Cas9	HPRT-38087	AATTATGGGGATTACTAGGA	Alt-R Cas9 crRNA:tra	HPRT-38087 EcoRI -20	C*A*CTTTTAACACACCCAAG	PS modified	AAGAATGTTGTGATAAAAGGTGATGCT	ACACATCCATGGGACTTCTGCCTC	NA	NA
S3D	HPRT1	Cas9	HPRT-38087	AATTATGGGGATTACTAGGA	Alt-R Cas9 crRNA:tra	HPRT-38087 EcoRI -15	T*T*AACACACCCAAGGAAAC	PS modified	AAGAATGTTGTGATAAAAGGTGATGCT	ACACATCCATGGGACTTCTGCCTC	NA	NA
S3D	HPRT1	Cas9	HPRT-38087	AATTATGGGGATTACTAGGA	Alt-R Cas9 crRNA:tra	HPRT-38087 EcoRI -10	A*C*ACCCAAGGAAAGACTA	PS modified	AAGAATGTTGTGATAAAAGGTGATGCT	ACACATCCATGGGACTTCTGCCTC	NA	NA
S3D	HPRT1	Cas9	HPRT-38087	AATTATGGGGATTACTAGGA	Alt-R Cas9 crRNA:tra	HPRT-38087 EcoRI -6	A*A*AGACTATGAAATGGAG	PS modified	AAGAATGTTGTGATAAAAGGTGATGCT	ACACATCCATGGGACTTCTGCCTC	NA	NA
S3D	HPRT1	Cas9	HPRT-38087	AATTATGGGGATTACTAGGA	Alt-R Cas9 crRNA:tra	HPRT-38087 EcoRI -3	A*A*AGACTATGAAATGGAG	PS modified	AAGAATGTTGTGATAAAAGGTGATGCT	ACACATCCATGGGACTTCTGCCTC	NA	NA
S3D	HPRT1	Cas9	HPRT-38087	AATTATGGGGATTACTAGGA	Alt-R Cas9 crRNA:tra	HPRT-38087 EcoRI +3	A*A*AGACTATGAAATGGAG	PS modified	AAGAATGTTGTGATAAAAGGTGATGCT	ACACATCCATGGGACTTCTGCCTC	NA	NA
S3D	HPRT1	Cas9	HPRT-38087	AATTATGGGGATTACTAGGA	Alt-R Cas9 crRNA:tra	HPRT-38087 EcoRI +6	A*A*AGACTATGAAATGGAG	PS modified	AAGAATGTTGTGATAAAAGGTGATGCT	ACACATCCATGGGACTTCTGCCTC	NA	NA
S3D	HPRT1	Cas9	HPRT-38087	AATTATGGGGATTACTAGGA	Alt-R Cas9 crRNA:tra	HPRT-38087 EcoRI +10	A*A*AGACTATGAAATGGAG	PS modified	AAGAATGTTGTGATAAAAGGTGATGCT	ACACATCCATGGGACTTCTGCCTC	NA	NA
S3D	HPRT1	Cas9	HPRT-38087	AATTATGGGGATTACTAGGA	Alt-R Cas9 crRNA:tra	HPRT-38087 EcoRI +15	A*A*AGACTATGAAATGGAG	PS modified	AAGAATGTTGTGATAAAAGGTGATGCT	ACACATCCATGGGACTTCTGCCTC	NA	NA
S3D	HPRT1	Cas9	HPRT-38087	AATTATGGGGATTACTAGGA	Alt-R Cas9 crRNA:tra	HPRT-38087 EcoRI +20	A*A*AGACTATGAAATGGAG	PS modified	AAGAATGTTGTGATAAAAGGTGATGCT	ACACATCCATGGGACTTCTGCCTC	NA	NA
S3D	HPRT1	Cas9	HPRT-38087	AATTATGGGGATTACTAGGA	Alt-R Cas9 crRNA:tra	HPRT-38087 EcoRI +25	A*A*AGACTATGAAATGGAG	PS modified	AAGAATGTTGTGATAAAAGGTGATGCT	ACACATCCATGGGACTTCTGCCTC	NA	NA
S3D	HPRT1	Cas9	HPRT-38087	AATTATGGGGATTACTAGGA	Alt-R Cas9 crRNA:tra	HPRT-38087 EcoRI +35	A*A*AGACTATGAAATGGAG	PS modified	AAGAATGTTGTGATAAAAGGTGATGCT	ACACATCCATGGGACTTCTGCCTC	NA	NA
S3D	HPRT1	Cas9	HPRT-38087	AATTATGGGGATTACTAGGA	Alt-R Cas9 crRNA:tra	HPRT-38087 EcoRI +45	A*A*AGACTATGAAATGGAG	PS modified	AAGAATGTTGTGATAAAAGGTGATGCT	ACACATCCATGGGACTTCTGCCTC	NA	NA
S3D	SAA1	Cas9	SAA1-88	AAGTTTTAAAAATCACTCCT	Alt-R Cas9 crRNA:tra	SAA1-88 EcoRI -45	T*G*TATGCCCCAGGATAACT	PS modified	TGCCCTCACTTCACACCTTC	GGTCCAGGGTGATGTTTCTCTT	NA	NA
S3D	SAA1	Cas9	SAA1-88	AAGTTTTAAAAATCACTCCT	Alt-R Cas9 crRNA:tra	SAA1-88 EcoRI -35	A*G*GATAACTATTTTTTTTAA	PS modified	TGCCCTCACTTCACACCTTC	GGTCCAGGGTGATGTTTCTCTT	NA	NA
S3D	SAA1	Cas9	SAA1-88	AAGTTTTAAAAATCACTCCT	Alt-R Cas9 crRNA:tra	SAA1-88 EcoRI -25	T*T*TTTTTTAAAGGAAATGA	PS modified	TGCCCTCACTTCACACCTTC	GGTCCAGGGTGATGTTTCTCTT	NA	NA
S3D	SAA1	Cas9	SAA1-88	AAGTTTTAAAAATCACTCCT	Alt-R Cas9 crRNA:tra	SAA1-88 EcoRI -20	T*T*TAAAGGAAATGATAATC	PS modified	TGCCCTCACTTCACACCTTC	GGTCCAGGGTGATGTTTCTCTT	NA	NA
S3D	SAA1	Cas9	SAA1-88	AAGTTTTAAAAATCACTCCT	Alt-R Cas9 crRNA:tra	SAA1-88 EcoRI -15	A*G*GAAATGATAATCTTGTT	PS modified	TGCCCTCACTTCACACCTTC	GGTCCAGGGTGATGTTTCTCTT	NA	NA
S3D	SAA1	Cas9	SAA1-88	AAGTTTTAAAAATCACTCCT	Alt-R Cas9 crRNA:tra	SAA1-88 EcoRI -10	A*T*GATAATCTTGTTGGGAA	PS modified	TGCCCTCACTTCACACCTTC	GGTCCAGGGTGATGTTTCTCTT	NA	NA
S3D	SAA1	Cas9	SAA1-88	AAGTTTTAAAAATCACTCCT	Alt-R Cas9 crRNA:tra	SAA1-88 EcoRI -6	T*G*TTGGGAAAGAGAAAAC	PS modified	TGCCCTCACTTCACACCTTC	GGTCCAGGGTGATGTTTCTCTT	NA	NA
S3D	SAA1	Cas9	SAA1-88	AAGTTTTAAAAATCACTCCT	Alt-R Cas9 crRNA:tra	SAA1-88 EcoRI -3	T*G*TTGGGAAAGAGAAAAC	PS modified	TGCCCTCACTTCACACCTTC	GGTCCAGGGTGATGTTTCTCTT	NA	NA
S3D	SAA1	Cas9	SAA1-88	AAGTTTTAAAAATCACTCCT	Alt-R Cas9 crRNA:tra	SAA1-88 EcoRI +3	T*G*TTGGGAAAGAGAAAAC	PS modified	TGCCCTCACTTCACACCTTC	GGTCCAGGGTGATGTTTCTCTT	NA	NA
S3D	SAA1	Cas9	SAA1-88	AAGTTTTAAAAATCACTCCT	Alt-R Cas9 crRNA:tra	SAA1-88 EcoRI +6	T*G*TTGGGAAAGAGAAAAC	PS modified	TGCCCTCACTTCACACCTTC	GGTCCAGGGTGATGTTTCTCTT	NA	NA
S3D	SAA1	Cas9	SAA1-88	AAGTTTTAAAAATCACTCCT	Alt-R Cas9 crRNA:tra	SAA1-88 EcoRI +10	T*G*TTGGGAAAGAGAAAAC	PS modified	TGCCCTCACTTCACACCTTC	GGTCCAGGGTGATGTTTCTCTT	NA	NA
S3D	SAA1	Cas9	SAA1-88	AAGTTTTAAAAATCACTCCT	Alt-R Cas9 crRNA:tra	SAA1-88 EcoRI +15	T*G*TTGGGAAAGAGAAAAC	PS modified	TGCCCTCACTTCACACCTTC	GGTCCAGGGTGATGTTTCTCTT	NA	NA
S3D	SAA1	Cas9	SAA1-88	AAGTTTTAAAAATCACTCCT	Alt-R Cas9 crRNA:tra	SAA1-88 EcoRI +20	T*G*TTGGGAAAGAGAAAAC	PS modified	TGCCCTCACTTCACACCTTC	GGTCCAGGGTGATGTTTCTCTT	NA	NA
S3D	SAA1	Cas9	SAA1-88	AAGTTTTAAAAATCACTCCT	Alt-R Cas9 crRNA:tra	SAA1-88 EcoRI +25	T*G*TTGGGAAAGAGAAAAC	PS modified	TGCCCTCACTTCACACCTTC	GGTCCAGGGTGATGTTTCTCTT	NA	NA
S3D	SAA1	Cas9	SAA1-88	AAGTTTTAAAAATCACTCCT	Alt-R Cas9 crRNA:tra	SAA1-88 EcoRI +35	T*G*TTGGGAAAGAGAAAAC	PS modified	TGCCCTCACTTCACACCTTC	GGTCCAGGGTGATGTTTCTCTT	NA	NA
S3D	SAA1	Cas9	SAA1-88	AAGTTTTAAAAATCACTCCT	Alt-R Cas9 crRNA:tra	SAA1-88 EcoRI +45	T*G*TTGGGAAAGAGAAAAC	PS modified	TGCCCTCACTTCACACCTTC	GGTCCAGGGTGATGTTTCTCTT	NA	NA
S3D	SERPINC1	Cas9	SERPINC1-72	AGTGGAGATAGTGTGATCTG	Alt-R Cas9 crRNA:tra	SERPINC1-72 EcoRI -27	T*T*CCTATCACATTGGAATA	PS modified	CTTGTCCCTCTTTGCCTTCTCT	GGGTGGATCTGAGTGGAAGAAA	NA	NA
S3D	SERPINC1	Cas9	SERPINC1-72	AGTGGAGATAGTGTGATCTG	Alt-R Cas9 crRNA:tra	SERPINC1-72 EcoRI -21	T*C*ACATTGGAATACATGGC	PS modified	CTTGTCCCTCTTTGCCTTCTCT	GGGTGGATCTGAGTGGAAGAAA	NA	NA
S3D	SERPINC1	Cas9	SERPINC1-72	AGTGGAGATAGTGTGATCTG	Alt-R Cas9 crRNA:tra	SERPINC1-72 EcoRI -15	T*G*GAATACATGGCCGCTAA	PS modified	CTTGTCCCTCTTTGCCTTCTCT	GGGTGGATCTGAGTGGAAGAAA	NA	NA
S3D	SERPINC1	Cas9	SERPINC1-72	AGTGGAGATAGTGTGATCTG	Alt-R Cas9 crRNA:tra	SERPINC1-72 EcoRI -10	T*A*CATGGCCGCTAATCTTC	PS modified	CTTGTCCCTCTTTGCCTTCTCT	GGGTGGATCTGAGTGGAAGAAA	NA	NA
S3D	SERPINC1	Cas9	SERPINC1-72	AGTGGAGATAGTGTGATCTG	Alt-R Cas9 crRNA:tra	SERPINC1-72 EcoRI -6	C*T*AATCTTCCACAGGGCTG	PS modified	CTTGTCCCTCTTTGCCTTCTCT	GGGTGGATCTGAGTGGAAGAAA	NA	NA
S3D	SERPINC1	Cas9	SERPINC1-72	AGTGGAGATAGTGTGATCTG	Alt-R Cas9 crRNA:tra	SERPINC1-72 EcoRI -3	C*T*AATCTTCCACAGGGCTG	PS modified	CTTGTCCCTCTTTGCCTTCTCT	GGGTGGATCTGAGTGGAAGAAA	NA	NA
S3D	SERPINC1	Cas9	SERPINC1-72	AGTGGAGATAGTGTGATCTG	Alt-R Cas9 crRNA:tra	SERPINC1-72 EcoRI +3	C*T*AATCTTCCACAGGGCTG	PS modified	CTTGTCCCTCTTTGCCTTCTCT	GGGTGGATCTGAGTGGAAGAAA	NA	NA
S3D	SERPINC1	Cas9	SERPINC1-72	AGTGGAGATAGTGTGATCTG	Alt-R Cas9 crRNA:tra	SERPINC1-72 EcoRI +6	C*T*AATCTTCCACAGGGCTG	PS modified	CTTGTCCCTCTTTGCCTTCTCT	GGGTGGATCTGAGTGGAAGAAA	NA	NA
S3D	SERPINC1	Cas9	SERPINC1-72	AGTGGAGATAGTGTGATCTG	Alt-R Cas9 crRNA:tra	SERPINC1-72 EcoRI +10	C*T*AATCTTCCACAGGGCTG	PS modified	CTTGTCCCTCTTTGCCTTCTCT	GGGTGGATCTGAGTGGAAGAAA	NA	NA
S3D	SERPINC1	Cas9	SERPINC1-72	AGTGGAGATAGTGTGATCTG	Alt-R Cas9 crRNA:tra	SERPINC1-72 EcoRI +15	C*T*AATCTTCCACAGGGCTG	PS modified	CTTGTCCCTCTTTGCCTTCTCT	GGGTGGATCTGAGTGGAAGAAA	NA	NA
S3D	SERPINC1	Cas9	SERPINC1-72	AGTGGAGATAGTGTGATCTG	Alt-R Cas9 crRNA:tra	SERPINC1-72 EcoRI +21	C*T*AATCTTCCACAGGGCTG	PS modified	CTTGTCCCTCTTTGCCTTCTCT	GGGTGGATCTGAGTGGAAGAAA	NA	NA
S3D	SERPINC1	Cas9	SERPINC1-72	AGTGGAGATAGTGTGATCTG	Alt-R Cas9 crRNA:tra	SERPINC1-72 EcoRI +27	C*T*AATCTTCCACAGGGCTG	PS modified	CTTGTCCCTCTTTGCCTTCTCT	GGGTGGATCTGAGTGGAAGAAA	NA	NA
S3D	SERPINC1	Cas9	SERPINC1-166	ACCTCTGGAAAAAGGTAAGA	Alt-R Cas9 crRNA:tra	SERPINC1-166 EcoRI -45	C*A*CTATCTCCACTTGCCCAC	PS modified	CTTGTCCCTCTTTGCCTTCTCT	GGGTGGATCTGAGTGGAAGAAA	NA	NA
S3D	SERPINC1	Cas9	SERPINC1-166	ACCTCTGGAAAAAGGTAAGA	Alt-R Cas9 crRNA:tra	SERPINC1-166 EcoRI -35	A*C*TTGCCCAGCCCTGTGGA	PS modified	CTTGTCCCTCTTTGCCTTCTCT	GGGTGGATCTGAGTGGAAGAAA	NA	NA
S3D	SERPINC1	Cas9	SERPINC1-166	ACCTCTGGAAAAAGGTAAGA	Alt-R Cas9 crRNA:tra	SERPINC1-166 EcoRI -25	C*C*CTGTGGAAGATTAGCGC	PS modified	CTTGTCCCTCTTTGCCTTCTCT	GGGTGGATCTGAGTGGAAGAAA	NA	NA
S3D	SERPINC1	Cas9	SERPINC1-166	ACCTCTGGAAAAAGGTAAGA	Alt-R Cas9 crRNA:tra	SERPINC1-166 EcoRI -20	T*G*GAAGATTAGCGGCCATC	PS modified	CTTGTCCCTCTTTGCCTTCTCT	GGGTGGATCTGAGTGGAAGAAA	NA	NA
S3D	SERPINC1	Cas9	SERPINC1-166	ACCTCTGGAAAAAGGTAAGA	Alt-R Cas9 crRNA:tra	SERPINC1-166 EcoRI -15	G*A*TTAGCGGCCATGTATTC	PS modified	CTTGTCCCTCTTTGCCTTCTCT	GGGTGGATCTGAGTGGAAGAAA	NA	NA
S3D	SERPINC1	Cas9	SERPINC1-166	ACCTCTGGAAAAAGGTAAGA	Alt-R Cas9 crRNA:tra	SERPINC1-166 EcoRI -10	G*C*GGCCATGTATTCCAATG	PS modified	CTTGTCCCTCTTTGCCTTCTCT	GGGTGGATCTGAGTGGAAGAAA	NA	NA
S3D	SERPINC1	Cas9	SERPINC1-166	ACCTCTGGAAAAAGGTAAGA	Alt-R Cas9 crRNA:tra	SERPINC1-166 EcoRI -6	A*T*TCCAATGTGATAGGAAC	PS modified	CTTGTCCCTCTTTGCCTTCTCT	GGGTGGATCTGAGTGGAAGAAA	NA	NA
S3D	SERPINC1	Cas9	SERPINC1-166	ACCTCTGGAAAAAGGTAAGA	Alt-R Cas9 crRNA:tra	SERPINC1-166 EcoRI -3	A*T*TCCAATGTGATAGGAAC	PS modified	CTTGTCCCTCTTTGCCTTCTCT	GGGTGGATCTGAGTGGAAGAAA	NA	NA
S3D	SERPINC1	Cas9	SERPINC1-166	ACCTCTGGAAAAAGGTAAGA	Alt-R Cas9 crRNA:tra	SERPINC1-166 EcoRI +3	A*T*TCCAATGTGATAGGAAC	PS modified	CTTGTCCCTCTTTGCCTTCTCT	GGGTGGATCTGAGTGGAAGAAA	NA	NA
S3D	SERPINC1	Cas9	SERPINC1-166	ACCTCTGGAAAAAGGTAAGA	Alt-R Cas9 crRNA:tra	SERPINC1-166 EcoRI +6	A*T*TCCAATGTGATAGGAAC	PS modified	CTTGTCCCTCTTTGCCTTCTCT	GGGTGGATCTGAGTGGAAGAAA	NA	NA
S3D	SERPINC1	Cas9	SERPINC1-166	ACCTCTGGAAAAAGGTAAGA	Alt-R Cas9 crRNA:tra	SERPINC1-166 EcoRI +10	A*T*TCCAATGTGATAGGAAC	PS modified	CTTGTCCCTCTTTGCCTTCTCT	GGGTGGATCTGAGTGGAAGAAA	NA	NA
S3D	SERPINC1	Cas9	SERPINC1-166	ACCTCTGGAAAAAGGTAAGA	Alt-R Cas9 crRNA:tra	SERPINC1-166 EcoRI +15	A*T*TCCAATGTGATAGGAAC	PS modified	CTTGTCCCTCTTTGCCTTCTCT	GGGTGGATCTGAGTGGAAGAAA	NA	NA
S3D	SERPINC1	Cas9	SERPINC1-166	ACCTCTGGAAAAAGGTAAGA	Alt-R Cas9 crRNA:tra	SERPINC1-166 EcoRI +20	A*T*TCCAATGTGATAGGAAC	PS modified	CTTGTCCCTCTTTGCCTTCTCT	GGGTGGATCTGAGTGGAAGAAA	NA	NA
S3D	SERPINC1	Cas9	SERPINC1-166	ACCTCTGGAAAAAGGTAAGA	Alt-R Cas9 crRNA:tra	SERPINC1-166 EcoRI +25	A*T*TCCAATGTGATAGGAAC	PS modified	CTTGTCCCTCTTTGCCTTCTCT	GGGTGGATCTGAGTGGAAGAAA	NA	NA
S3D	SERPINC1	Cas9	SERPINC1-166	ACCTCTGGAAAAAGGTAAGA	Alt-R Cas9 crRNA:tra	SERPINC1-166 EcoRI +35	A*T*TCCAATGTGATAGGAAC	PS modified	CTTGTCCCTCTTTGCCTTCTCT	GGGTGGATCTGAGTGGAAGAAA	NA	NA
S3D	SERPINC1	Cas9	SERPINC1-166	ACCTCTGGAAAAAGGTAAGA	Alt-R Cas9 crRNA:tra	SERPINC1-166 EcoRI +45	A*T*TCCAATGTGATAGGAAC	PS modified	CTTGTCCCTCTTTGCCTTCTCT	GGGTGGATCTGAGTGGAAGAAA	NA	NA
S5	HPRT	Cas12a	HPRT-38330-AS	GGTTAAAGATGGTTAAATGAT	Alt-R Cas12a crRNA	HPRT 38330 EcoRI +2	CATTTCACATAAAACTCTTTTA	Alt-R modified	NA	NA	ACACTCTTTCCCTACACGACGCTCTTCCGATCTCCCACGAAGTGTTGGATATAAG	GTGACTGGAGTTCAGACGTGTGCTCTTCCGATCTGGCATATCCTACAACAAACTTGT
S5	HPRT	Cas12a	HPRT-38330-AS	GGTTAAAGATGGTTAAATGAT	Alt-R Cas12a crRNA	HPRT 38330 EcoRI +4	CATTTCACATAAAACTCTTTTA	Alt-R modified	NA	NA	ACACTCTTTCCCTACACGACGCTCTTCCGATCTCCCACGAAGTGTTGGATATAAG	GTGACTGGAGTTCAGACGTGTGCTCTTCCGATCTGGCATATCCTACAACAAACTTGT
S5	HPRT	Cas12a	HPRT-38330-AS	GGTTAAAGATGGTTAAATGAT	Alt-R Cas12a crRNA	HPRT 38330 EcoRI +6	CATTTCACATAAAACTCTTTTA	Alt-R modified	NA	NA	ACACTCTTTCCCTACACGACGCTCTTCCGATCTCCCACGAAGTGTTGGATATAAG	GTGACTGGAGTTCAGACGTGTGCTCTTCCGATCTGGCATATCCTACAACAAACTTGT
S5	HPRT	Cas12a	HPRT-38330-AS	GGTTAAAGATGGTTAAATGAT	Alt-R Cas12a crRNA	HPRT 38330 EcoRI +10	CATTTCACATAAAACTCTTTTA	Alt-R modified	NA	NA	ACACTCTTTCCCTACACGACGCTCTTCCGATCTCCCACGAAGTGTTGGATATAAG	GTGACTGGAGTTCAGACGTGTGCTCTTCCGATCTGGCATATCCTACAACAAACTTGT
S5	HPRT	Cas12a	HPRT-38330-AS	GGTTAAAGATGGTTAAATGAT	Alt-R Cas12a crRNA	HPRT 38330 EcoRI +15	CATTTCACATAAAACTCTTTTA	Alt-R modified	NA	NA	ACACTCTTTCCCTACACGACGCTCTTCCGATCTCCCACGAAGTGTTGGATATAAG	GTGACTGGAGTTCAGACGTGTGCTCTTCCGATCTGGCATATCCTACAACAAACTTGT
S5	HPRT	Cas12a	HPRT-38330-AS	GGTTAAAGATGGTTAAATGAT	Alt-R Cas12a crRNA	HPRT 38330 EcoRI +21	CATTTCACATAAAACTCTTTTA	Alt-R modified	NA	NA	ACACTCTTTCCCTACACGACGCTCTTCCGATCTCCCACGAAGTGTTGGATATAAG	GTGACTGGAGTTCAGACGTGTGCTCTTCCGATCTGGCATATCCTACAACAAACTTGT
S5	HPRT	Cas12a	HPRT-38330-AS	GGTTAAAGATGGTTAAATGAT	Alt-R Cas12a crRNA	HPRT 38330 EcoRI +27	CATTTCACATAAAACTCTTTTA	Alt-R modified	NA	NA	ACACTCTTTCCCTACACGACGCTCTTCCGATCTCCCACGAAGTGTTGGATATAAG	GTGACTGGAGTTCAGACGTGTGCTCTTCCGATCTGGCATATCCTACAACAAACTTGT
S5	HPRT	Cas12a	HPRT-38330-AS	GGTTAAAGATGGTTAAATGAT	Alt-R Cas12a crRNA	HPRT 38330 EcoRI -2	CATTTCACATAAAACTCTTTTA	Alt-R modified	NA	NA	ACACTCTTTCCCTACACGACGCTCTTCCGATCTCCCACGAAGTGTTGGATATAAG	GTGACTGGAGTTCAGACGTGTGCTCTTCCGATCTGGCATATCCTACAACAAACTTGT
S5	HPRT	Cas12a	HPRT-38330-AS	GGTTAAAGATGGTTAAATGAT	Alt-R Cas12a crRNA	HPRT 38330 EcoRI -4	CATTTCACATAAAACTCTTTTA	Alt-R modified	NA	NA	ACACTCTTTCCCTACACGACGCTCTTCCGATCTCCCACGAAGTGTTGGATATAAG	GTGACTGGAGTTCAGACGTGTGCTCTTCCGATCTGGCATATCCTACAACAAACTTGT
S5	HPRT	Cas12a	HPRT-38330-AS	GGTTAAAGATGGTTAAATGAT	Alt-R Cas12a crRNA	HPRT 38330 EcoRI -6	CATTTCACATAAAACTCTTTTA	Alt-R modified	NA	NA	ACACTCTTTCCCTACACGACGCTCTTCCGATCTCCCACGAAGTGTTGGATATAAG	GTGACTGGAGTTCAGACGTGTGCTCTTCCGATCTGGCATATCCTACAACAAACTTGT
S5	HPRT	Cas12a	HPRT-38330-AS	GGTTAAAGATGGTTAAATGAT	Alt-R Cas12a crRNA	HPRT 38330 EcoRI -10	AATTATAAGCCATTTCACATA	Alt-R modified	NA	NA	ACACTCTTTCCCTACACGACGCTCTTCCGATCTCCCACGAAGTGTTGGATATAAG	GTGACTGGAGTTCAGACGTGTGCTCTTCCGATCTGGCATATCCTACAACAAACTTGT
S5	HPRT	Cas12a	HPRT-38330-AS	GGTTAAAGATGGTTAAATGAT	Alt-R Cas12a crRNA	HPRT 38330 EcoRI -15	TAAGCAATTATAAGCCATTTC	Alt-R modified	NA	NA	ACACTCTTTCCCTACACGACGCTCTTCCGATCTCCCACGAAGTGTTGGATATAAG	GTGACTGGAGTTCAGACGTGTGCTCTTCCGATCTGGCATATCCTACAACAAACTTGT
S5	HPRT	Cas12a	HPRT-38330-AS	GGTTAAAGATGGTTAAATGAT	Alt-R Cas12a crRNA	HPRT 38330 EcoRI -21	ATTCTCTAAGCAATTATAAGC	Alt-R modified	NA	NA	ACACTCTTTCCCTACACGACGCTCTTCCGATCTCCCACGAAGTGTTGGATATAAG	GTGACTGGAGTTCAGACGTGTGCTCTTCCGATCTGGCATATCCTACAACAAACTTGT
S5	HPRT	Cas12a	HPRT-38330-AS	GGTTAAAGATGGTTAAATGAT	Alt-R Cas12a crRNA	HPRT 38330 EcoRI -27	ACAAATATTCTCTAAGCAATT	Alt-R modified	NA	NA	ACACTCTTTCCCTACACGACGCTCTTCCGATCTCCCACGAAGTGTTGGATATAAG	GTGACTGGAGTTCAGACGTGTGCTCTTCCGATCTGGCATATCCTACAACAAACTTGT
